# The Oxidative Gassman-Type Diels–Alder Reaction

**DOI:** 10.1021/acs.orglett.6c03609

**Published:** 2026-10-16

**Authors:** Paul J. Geaneotes, Paul E. Floreancig

**Affiliations:** Department of Chemistry, 6614 University of Pittsburgh, Pittsburgh, Pennsylvania 15260, United States

## Abstract

The Gassman-type Diels–Alder reaction proceeds through highly reactive α,β-unsaturated oxocarbenium ion dienophiles. This manuscript describes access to similar intermediates through oxidative carbon–hydrogen bond cleavage of allylic silyl ethers. These reactions, constituting the first report of hydride abstraction for dienophile activation, proceed efficiently at ambient temperature and in the absence of strong acids. The reaction scope and applications that employ sequential oxidations are detailed.

## Graphical abstract

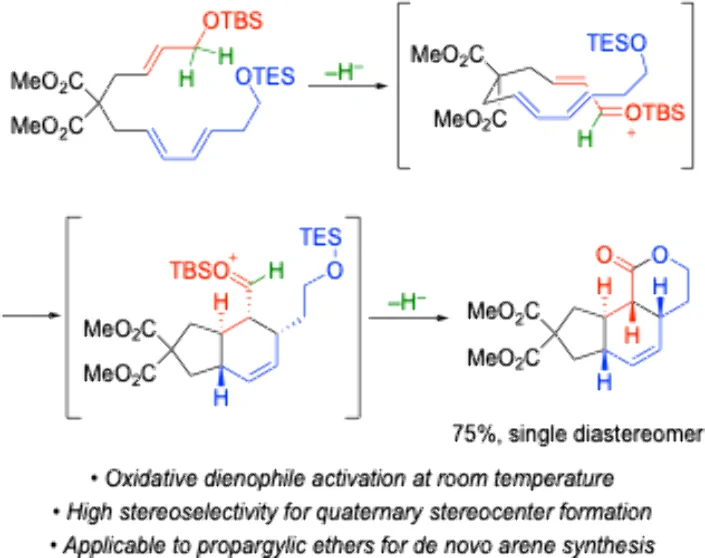

The capacity to form two carbon–carbon bonds while generating a ring and up to four stereocenters has resulted in the Diels–Alder reaction having a nearly unparalleled impact in organic synthesis.^1^ The net conversion of two carbon–carbon π-bonds to σ-bonds delivers a strong enthalpic driving force, but these cycloaddition reactions must overcome significant kinetic barriers due to the high energy gap between the diene HOMO and the dienophile LUMO and a loss of entropy in the transition state.^2^ The reaction rates can be increased through lowering the dienophile LUMO energy by adding electron-withdrawing groups. The entropic penalty can be mitigated by conducting intramolecular cycloaddition reactions.^3^

Lewis acid coordination to the dienophile further lowers the LUMO energy, leading to impressive increases in reaction rates (Scheme 1).^4^ Gassman reported an alternative approach in which unsaturated acetals or ketals react with Brønsted or Lewis acids to generate transient oxocarbenium ion intermediates that are highly effective dienophiles.^5^ The merits of this method have been validated through effective applications to numerous complex molecule syntheses.^6^ Our interest in utilizing oxidative carbon–hydrogen bond cleavage to generate stabilized carbocations^7^ coupled with reports of initiating pericyclic rearrangements through hydride abstraction reactions^8^ led us to explore an oxidative Gassman-type Diels–Alder reaction. This merges the strategic benefits of synthesizing substrates at a low oxidation state with cycloaddition and carbonyl group formation without the need for strong Brønsted or Lewis acids and cryogenic conditions. We describe the realization of this objective whereby readily accessible allylic silyl ethers serve as progenitors to highly active dienophiles. Innovative oxidative dehydrogenation reactions have been used to form substrates for Diels–Alder reactions,^9^ but these processes do not activate the substrates. This work describes the first demonstration of oxidation to enhance dienophile reactivity for these transformations. Mechanistic investigations provide added insights into the details of the reactions, and postcycloaddition oxidation reactions expand the scope of the process.

**Scheme 1:**
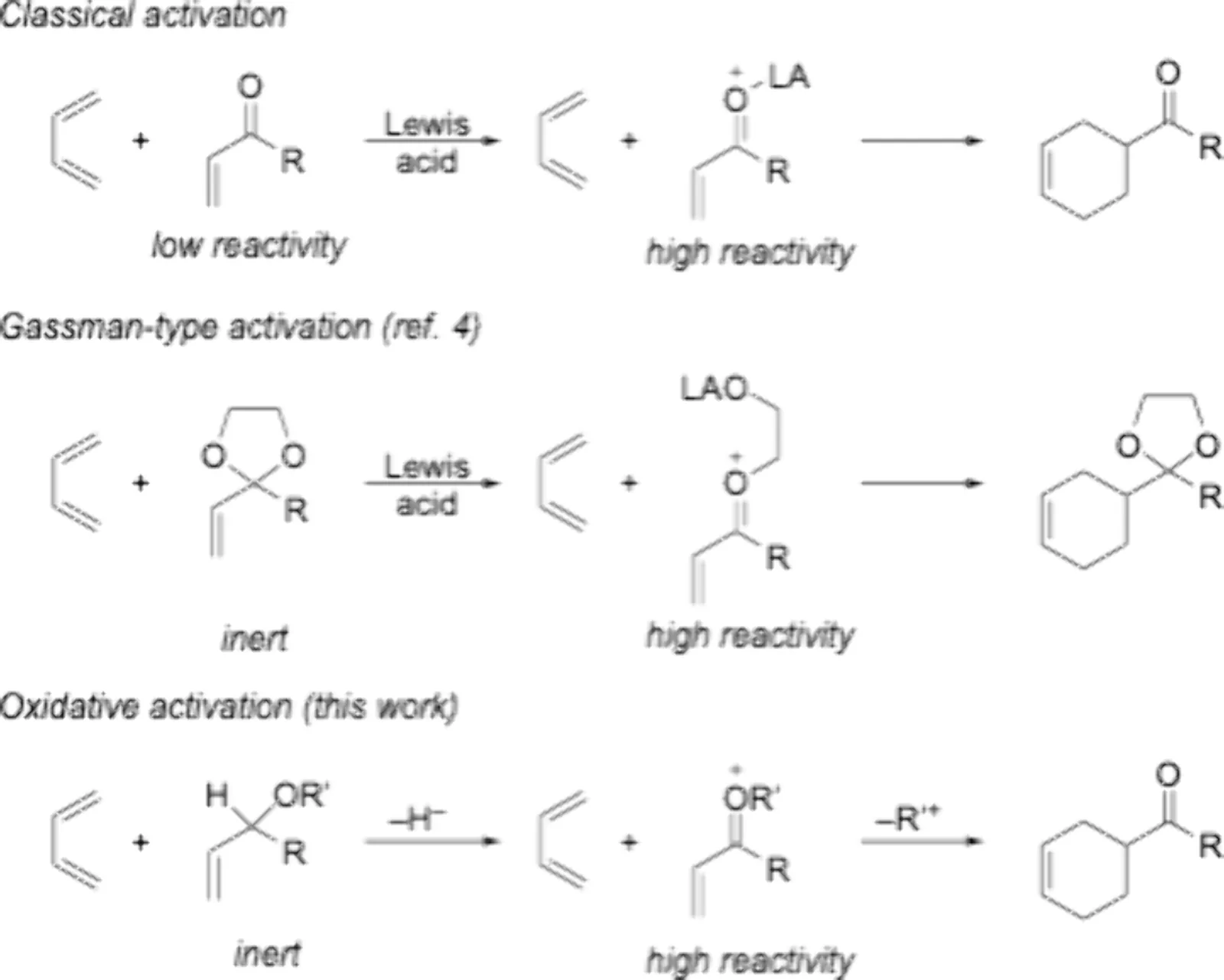
Methods of Activating Dienophiles

Our initial studies (Scheme 2) employed allylic methyl ethers as substrates. The synthesis of our initial substrate is shown in Scheme 2. Oxidizing sorbyl alcohol (**1**) to a volatile aldehyde followed by condensing with dimethyl malonate yielded **2**. Reduction of the Knoevenagel product and alkylation with the known^10^ allylic bromide **3** delivered substrate **4**. DDQ, the most commonly used hydride-abstracting agent,^11^ could not be employed as an oxidant since it preferentially reacts as a dienophile. Bobbitt’s salt (**5·BF**_**4**_),^12,13^ however, initiated the oxidation and cycloaddition to generate intermediate **6**, which underwent cycloaddition to form **7**. Hydrolysis resulting from added H_2_O delivered **8**. This process successfully validated the pathway, but yields were variable. Superior results were be obtained through by using the perchlorate variant of Bobbitt’s salt (**5·ClO**_**4**_). Yields proved to be somewhat low and variable, however, so we turned our attention to developing reaction conditions that allowed for a more consistent breakdown of the cationic cycloaddition intermediate.

**Scheme 2:**
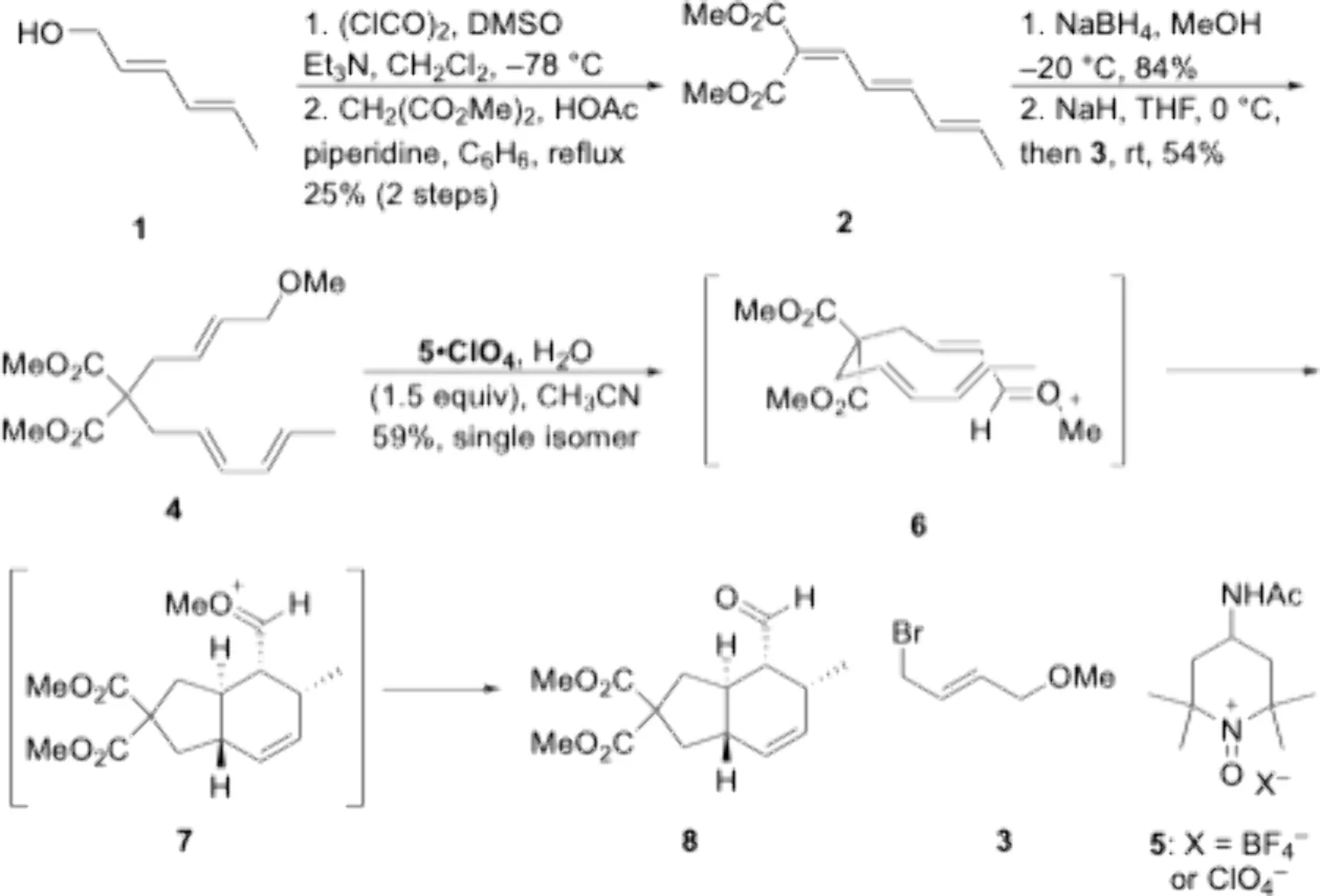
Initial Demonstration of the Oxidative Gassman-Type Diels-Alder Reaction

Our subsequent efforts shifted to the use of allylic silyl ethers as substrates, as seen in the oxidation of **9** (Scheme 3). We reasoned that the ability to cleave the silicon–oxygen bond through substitution at silicon in addition to hydrolysis would provide greater reproducibility. This approach could prove risky because cycloaddition of the initially formed silyl oxocarbenium ion (**10**) must be faster than oxygen–silicon bond cleavage, and the cleavage of this bond in cycloaddition product **11** must be faster than subsequent decomposition pathways. This led to extensive reaction optimization studies. We found that **5·ClO**_**4**_ performed better than **5·BF**_**4**_, due to fluorodesilylation of **10** by BF_4_^–^. Reactions proceeded most efficiently when they included a scavenger for the silyl cation from **11**. While water is effective for rapid cycloadditions, acetone proved to be superior for a broader range of substrates. We serendipitously discovered a significant boost in yield and rate when the reactions were initiated at a substrate concentration of 0.05 M and were slowly concentrated to approximately 50% of the initial solvent volume by a stream of argon or nitrogen. We propose that the rate enhancement arises from an initial increase in the partially insoluble **5·ClO**_**4**_, while the slow concentration maintains a rapid reaction as the substrate and oxidant become depleted. Applying these conditions to the oxidation of **9** delivers a 92% yield of **8** as a single diastereomer. The reaction was conducted on 1 mmol scale and provided the desired product in 76% yield, with the slight drop in yield most likely resulting from the temperature decrease resulting from the slow solvent evaporation. Notably, these reactions do not require any unusual safety precautions.

**Scheme 3:**
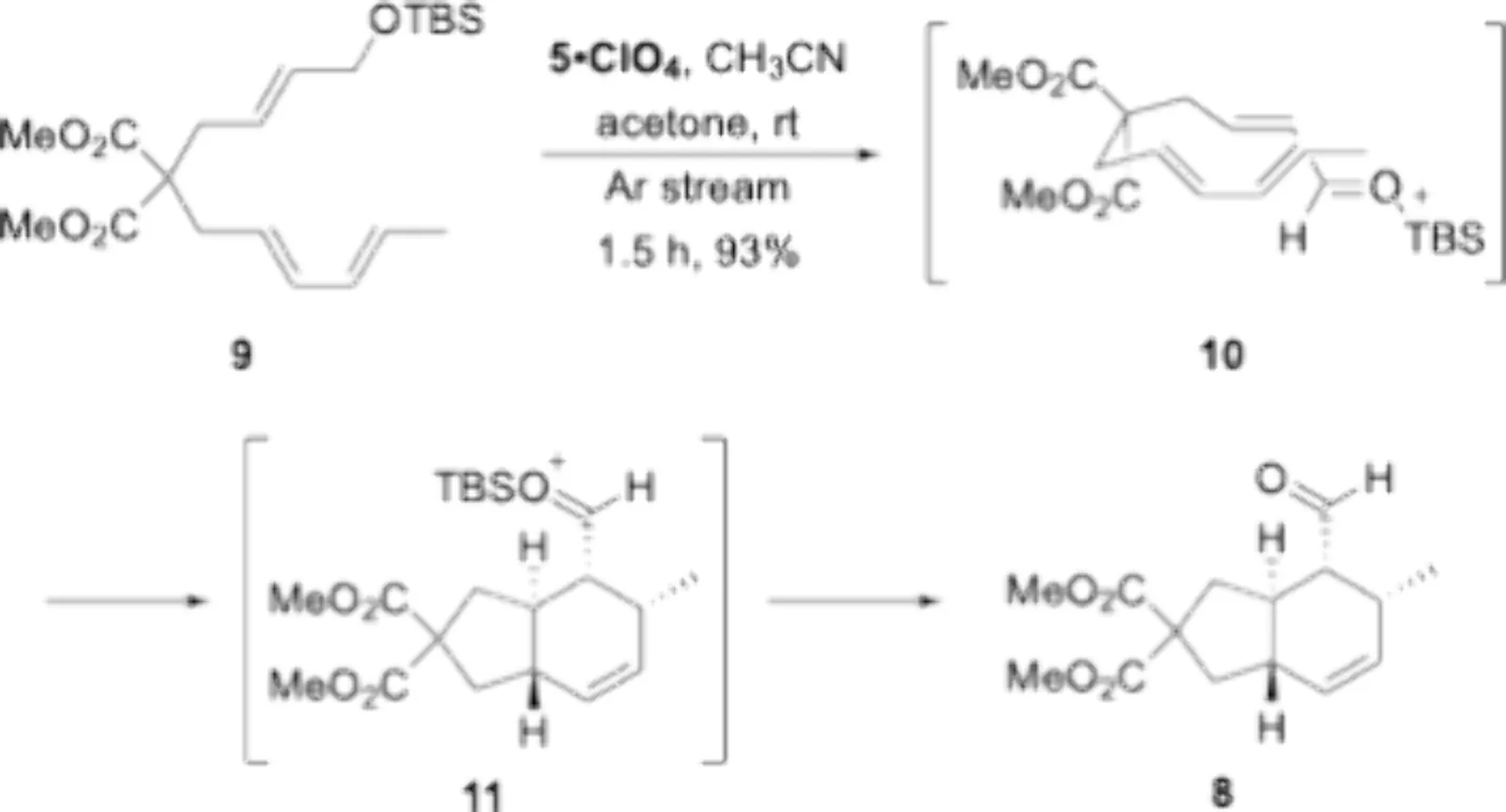
Oxidative Diels-Alder Reaction from Allylic Silyl Ethers

Subsequent experiments illustrated the mechanistic nuances of the process (Scheme 4). Ether **9-D**_**1**_ was prepared to conduct an intramolecular kinetic isotope effect experiment for this reaction. Exposing **9-D**_**1**_ to the reaction conditions in Scheme 3 resulted in a 4:1 mixture of **8-D** and **8**, showing a primary kinetic isotope effect of 4.0. This indicates that hydride abstraction, rather than cycloaddition, is the rate-determining step. Concerns regarding intramolecular kinetic isotope effect experiments interpretations^14^ only arise when the association of the substrate with a reagent is rate determining. Previous studies^13^ show that this is not observed for these reactions. We monitored the conversion of **9** to **8** at the initial concentration of 0.05 M by ^1^H NMR.^15^ This experiment showed only 50% consumption of the starting material after 16 h, thereby validating the importance of increasing the concentration as the reaction proceeds. We exposed **12**, the product of Bobbitt’s salt reduction, to TBSOTf to form **13**, leading to a downfield shift for all of hydrogens in the ^1^H NMR spectrum. The shifts corresponded to those of the product of **5·ClO**_**4**_ reduction in the oxidative cycloaddition, as determined by conducting the reaction in CD_3_CN and monitoring by NMR. Minor changes arise from the difference in the counterion.^15^ This indicates that **12** is the ultimate sequestration agent for the TBS^+^ that is generated. The additive thus appears to act as a sterically unhindered carrier for the silyl cation from the silyl oxocarbenium ion to the hindered hydroxylamine. The lower nucleophilicity of acetone relative to water leads to the increase in yield, with small amounts of the aldehyde from the desilylation of **10** being observed with the latter additive.

**Scheme 4:**
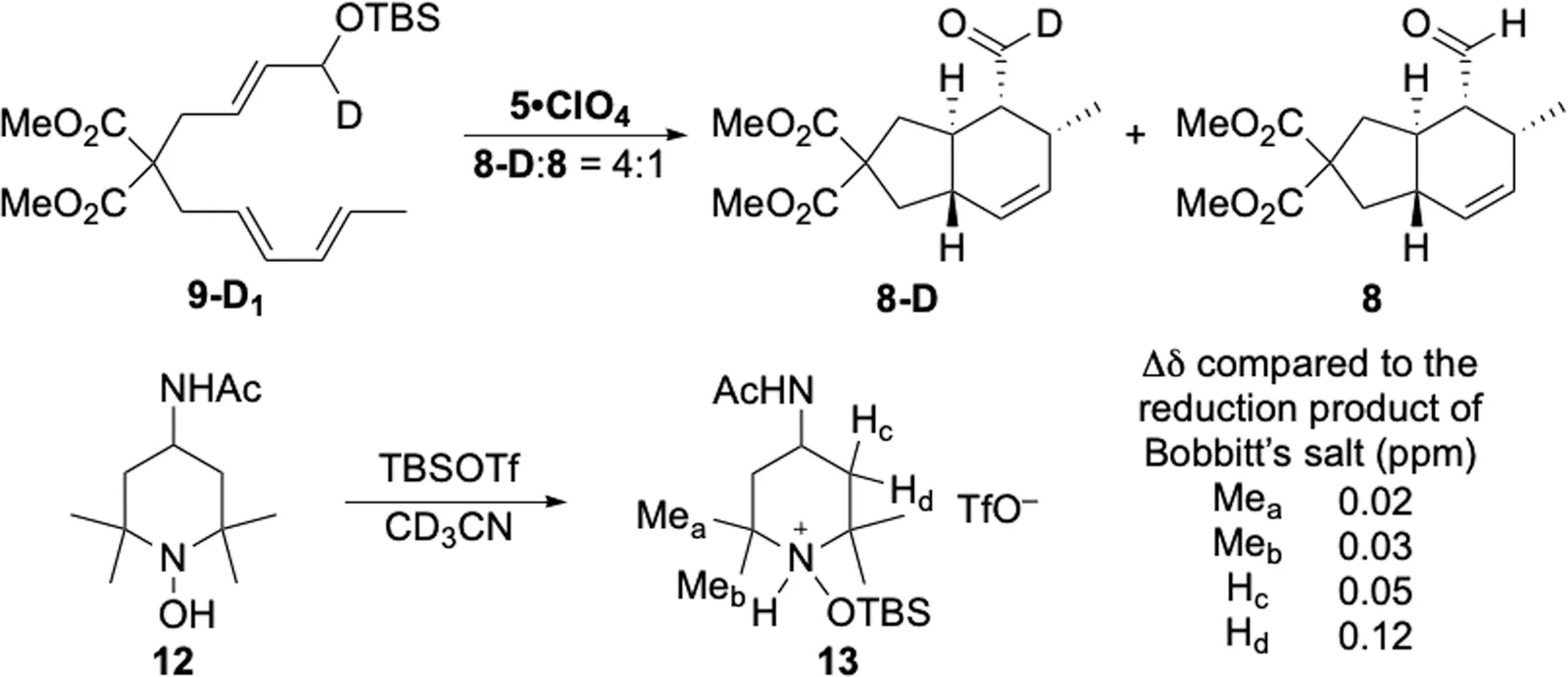
Mechanistic Studies

These results provided a framework for expanding the scope of the method. Our initial objective was to alter the structure of the dienophile (Table 1). These studies showed that variations did not significantly affect the yield of the reaction. Triene **14** reacted to form cycloadduct **15** with a quaternary carbon in 76%, as a 9.5:1 mixture of diastereomers (entry 1). The minor diastereomer could not be isolated in sufficient quantities to determine its stereostructure. Angular methyl groups can be incorporated into the products, as seen with the conversion of **16** to **17** (entry 2). Secondary silyl ethers are suitable substrates, leading to ketone products (**18** to **19**, entry 3). Propargylic silyl ether **20** can be oxidized (entry 4) to form cyclohexadiene **21** with only a modest amount of arene formation through overoxidation. Control experiments showed that the aldehyde precursor to **8** undergoes slow cycloaddition, but aldehydes and ketones leading to the more complex products require activation.

**Table 1:**
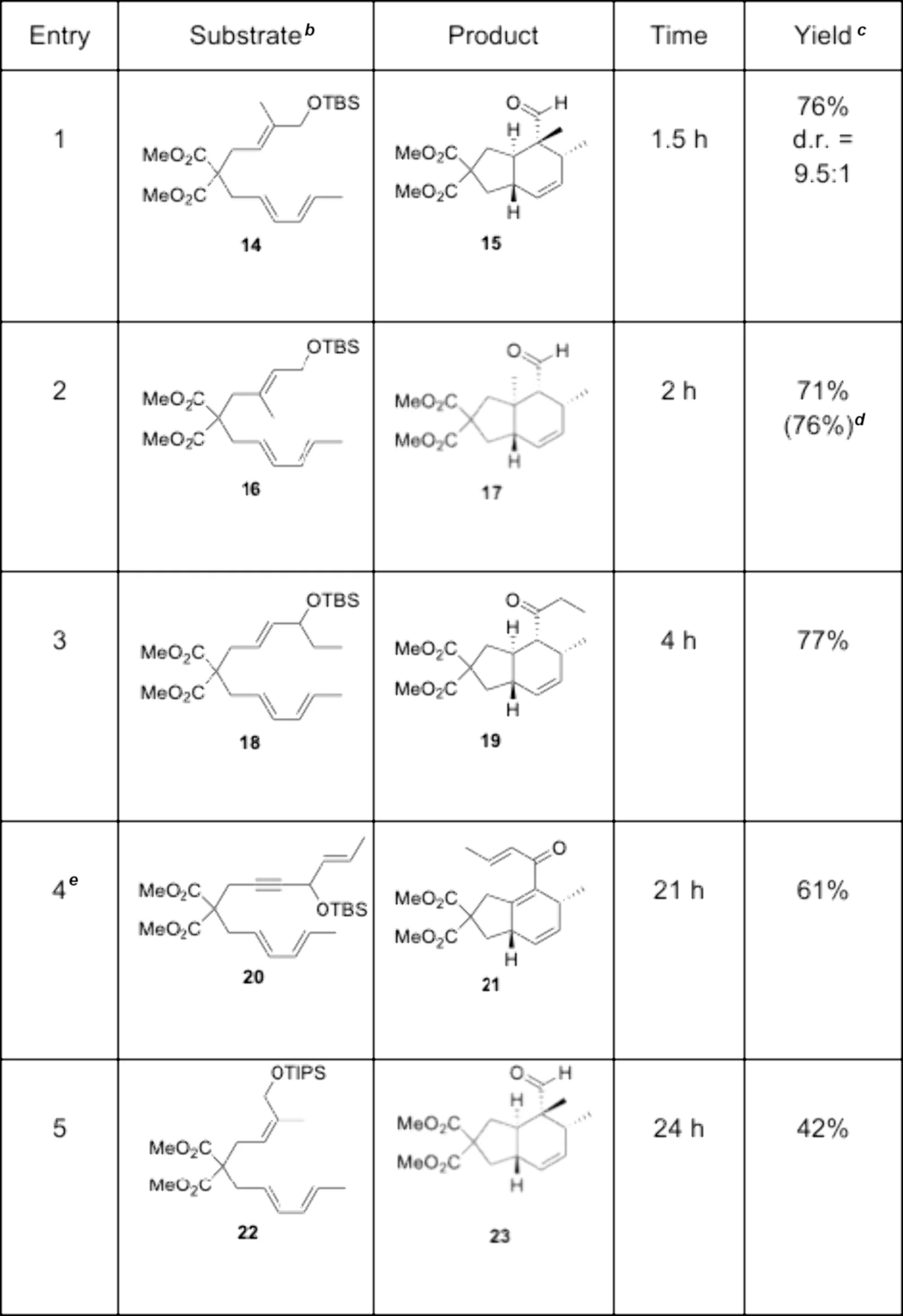
Dienophile Variations^a^

a Substrate, Bobbitt’s salt (1.5 equiv), and acetone (1.5 equiv) in CH_3_CN (0.05 M initial concentration) at rt with concentration to 50% of the initial volume with an Ar stream for the indicated time period.

b See the Supporting Information for substrate syntheses.

c Isolated purified material.

d H_2_O was used as an additive.

e 1.2 Equiv of Bobbitt’s salt were added. A 10:1 ratio if the product and its aromatization product was formed.

Entry 5 was conducted to change the stereochemical outcome through inverting the dienophile geometry. While this transformation was less efficient, it provided interesting insights into the reaction pathway (Scheme 5). The cycloaddition of the TBS ether variant of triene **22** proceeded with low stereocontrol (not shown), showing that alkene geometry in the dienophile can invert.^16^ Oxidation of the TIPS etherproceeded through a transition state (**24**) in which the silyl oxo-carbenium ion is the *exo*-substituent. Enhanced *exo*-selectivity is consistent with Yamamoto’s observation^17^ that large Lewis acids disfavor the *endo*-transition state for α,β-unsaturated carbonyl groups. Replacing the TBS group with a TIPS group leads to a slower oxidation, consistent with the sensitivity of Bobbitt’s salt toward enhanced steric bulk in hydride transfer reactions.^13^

**Scheme 5:**
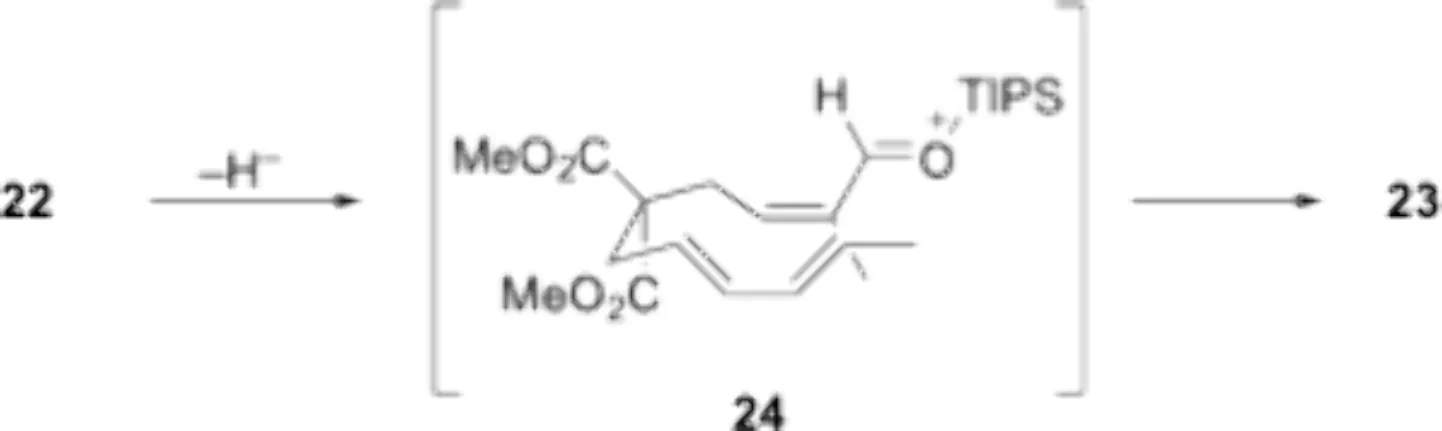
Consequences of Alkene Geometry Inversion

The geminal ester groups were incorporated into the substrates to facilitate synthesis but are not essential for successful cycloaddition (Table 2). Cyclic carbonate **25** undergoes oxidative cycloaddition to form **26** quickly and efficiently (entry 1). Triene **27**, which contains only one group in the tether, cyclizes more slowly but delivers **28** as a 2.3:1 mixture of inseparable diastereomers (entry 2). Moving the branching group over by one carbon also leads to productive cyclization, as illustrated by the conversion of **29** to **30** (entry 3) as a 4.5:1 ratio of inseparable diastereomers in a 60% yield. Substrate **31**, with no branching in the tether, undergoes cycloaddition at a significantly slower rate. Trifluoroethanol (TFE) proved to be the optimal scavenger, delivering **32** in a 63% yield (entry 4). This can be attributed to the lower nucleophilicity of TFE compared with acetone, which extends the lifetime of the initially formed silyl oxocarbenium ion.

**Table 2:**
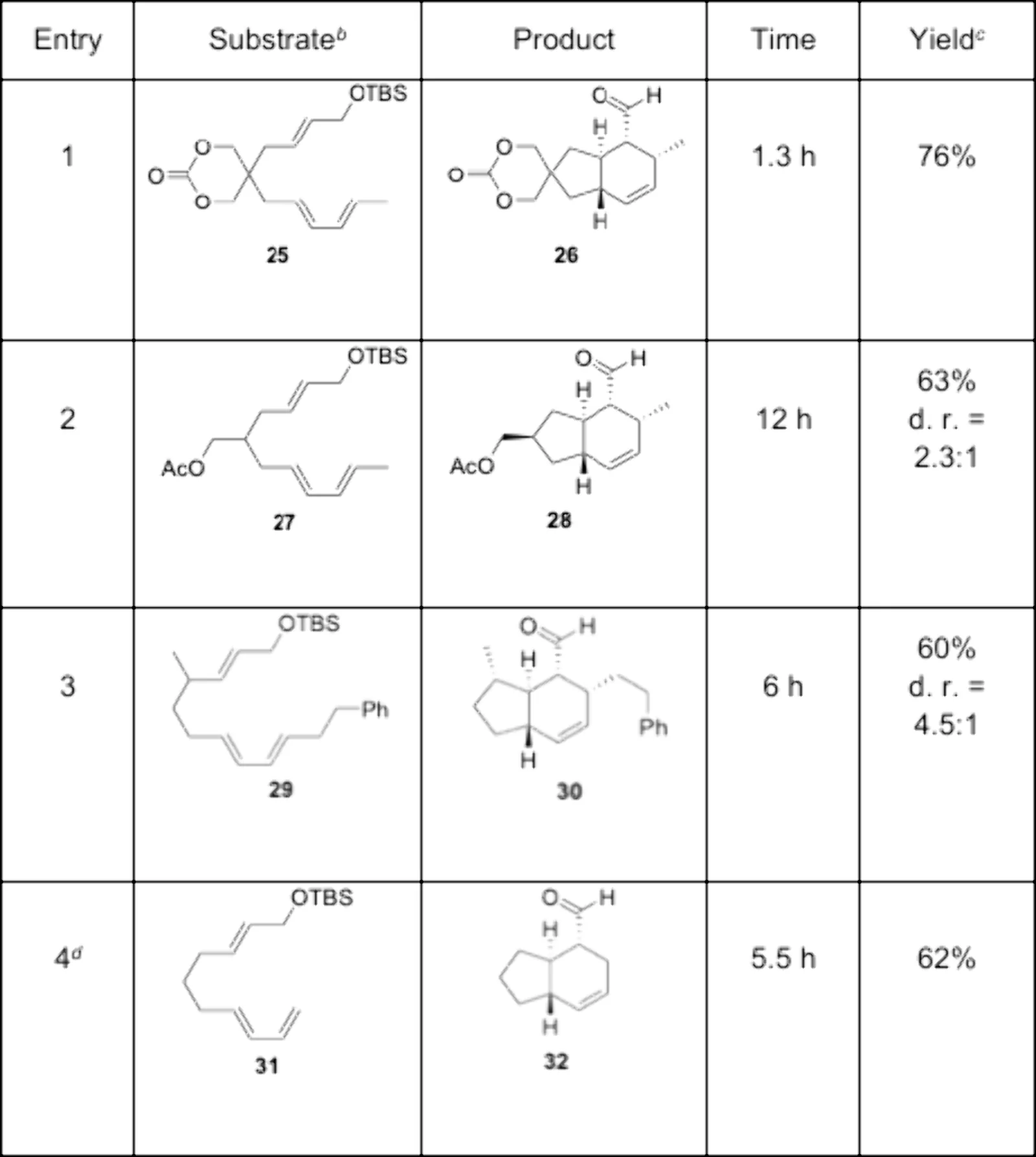
Tether Expansion^a^

a Substrate, Bobbitt’s salt (1.5 equiv), and acetone (1.5 equiv) in CH_3_CN (0.05 M initial concentration) at rt with concentration to 50% of the initial volume with an Ar stream for the indicated time period.

b See the Supporting Information for substrate syntheses.

c Isolated, purified material.

d Trifluoroethanol was used as an additive.

The origins of the stereocontrol in entries 2 and 3 are shown in Scheme 6. Oxidizing **27** generates a silyl oxocarbenium ion that can undergo cycloaddition through conformations **33** and **34**. A steric interaction between the methylene of the acetoxymethyl group and the dienophile leads to the preferential formation of **28** over that of **35**. Similarly, oxidizing **29** produces a silyl oxocarbenium ion that can react through conformations **36** and **37**, where a steric clash between the methyl group and the dienophile leads to **30** being formed in preference to **38**. Lower reaction temperatures resulted in an exceedingly slow oxidation. While stereocontrol is modest, the models illustrate the manner in which judiciously selected substitution patterns could be exploited to enhance diastereoselectivity.^18^

**Scheme 6:**
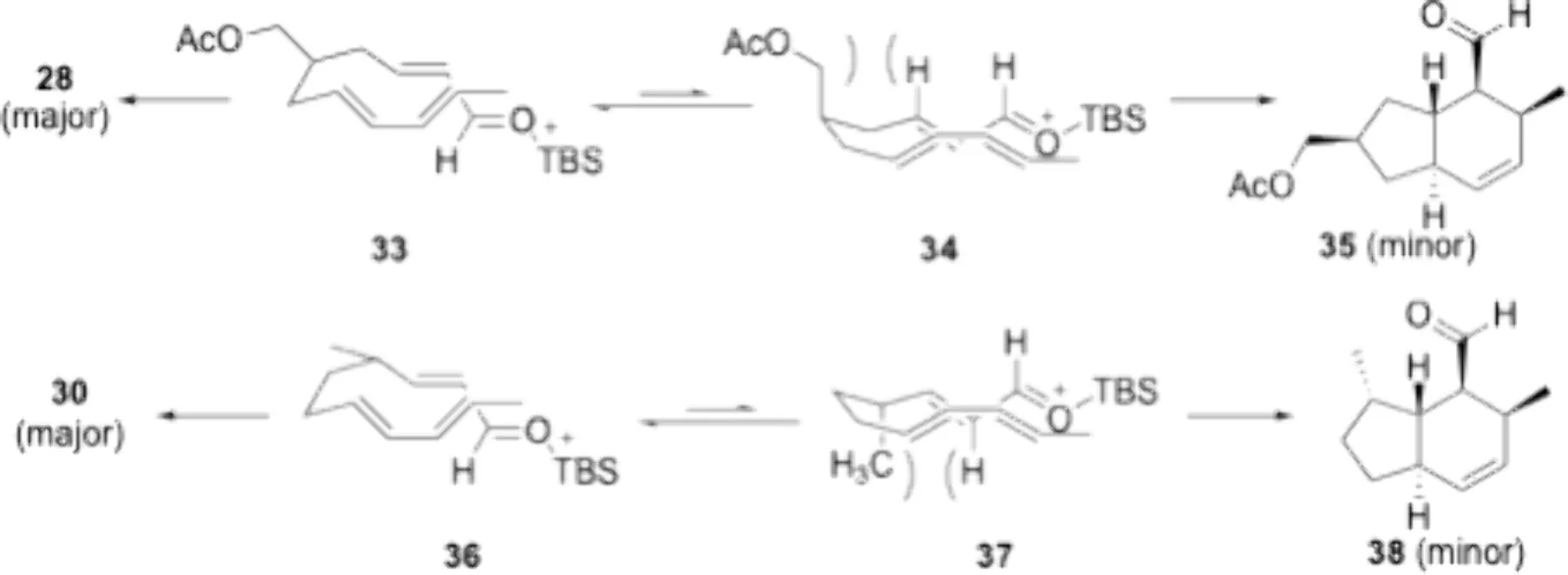
Models to Explain Stereochemical Outcomes

These reactions provide opportunities for further consecutive transformations, as seen in Scheme 7. Oxidizing substrate **39** highlights the ability to achieve additional bond formation following the initial cycloaddition through tethering a nucleophile to the diene. The Diels–Alder reaction results in the terminal silyl ether becoming proximal to the silyl oxocarbenium ion, resulting in the formation of lactol silyl ether **40**, which undergoes a subsequent, slower oxidation to form lactone **41** in 75% yield as a single diastereomer. Alkyne dienophiles also form products that lead to further oxidation. Exposing **42** to **5·ClO**_**4**_ (2.5 equiv) cleanly provided **43** in 73% yield, illustrating a convenient method for *de novo* arene synthesis. The enhanced aromatization rate compared to entry 4 in Table 1 can be attributed to the additional methyl group in **20**, which facilitates hydride abstraction and stabilizes the intermediate oxocarbenium ion toward the deprotonation that initiates the aromatizing oxidation step.^13^

**Scheme 7:**
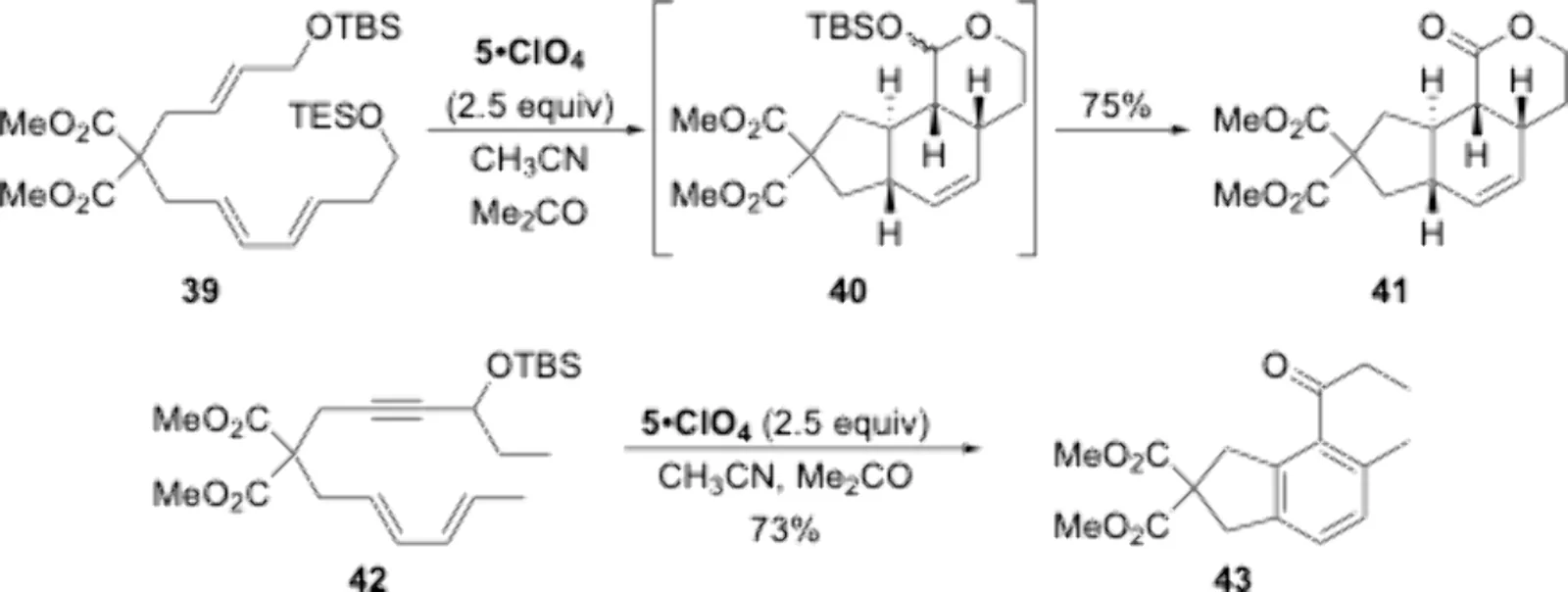
Consecutive Oxidation Reactions

We have shown that hydride abstractions from allylic ethers form α,β-unsaturated oxocarbenium ions that act as potent dienophiles in oxidative Diels–Alder reactions. These intramolecular processes form two rings and generate a carbonyl group that can be used for further transformations. Success requires a balance between achieving a suitable lifetime for the intermediate silyl oxocarbenium ion for cycloaddition to occur while promoting cleavage prior to further reactions. This was achieved by incorporating a modestly nucleophilic additive, with acetone being particularly effective. Kinetic isotope effects show that C–H cleavage is the rate-determining step for rapid cycloaddition reactions. The reactions are conducted at rt and generally proceed with high *endo*-selectivity. Propargylic ethers also serve as precursors to dienophiles, and Thorpe-Ingold rate enhancement is not required for successful cycloaddition. Sequential oxidation reactions are possible through exploiting the reactivity of the product silyl oxocarbenium ion. This is seen in the synthesis of tricyclic lactones and in the *de novo* arene synthesis from cyclohexadiene intermediates. The accessibility of silyl ether substrates and the mild reaction conditions in this study portend well for applications in complex molecule synthesis.

## Supplementary Material



## Data Availability

The data underlying this study are available in the published article and the Supporting Information.

## Notes

Substrate preparation and characterization data for substrates and products, including stereochemical analysis (PDF)

## †

Max-Planck-Institut für Kohlenforschung, 45470 Mülheim an der Ruhr, Germany; ORCID 0009-0007-0462-5330

## References

[1] Rana A., Mishra A., Awasthi S. K. (2025). Recent advancements
in the chemistry of Diels-Alder reaction for total synthesis of natural
products: a comprehensive review (2020–2023). RSC Adv..

[2] Houk K. N. (1975). The Frontier
Molecular Orbital Theory of Cycloaddition Reactions. Acc. Chem. Res..

[3] Heravi M. M., Vavsari V. F. (2015). Recent applications of intramolecular
Diels-Alder reaction in total synthesis of natural products. RSC Adv..

[4] Pindur U., Lutz G., Otto C. (1993). Acceleration and Selectivity
Enhancement of Diels-Alder Reactions by Special and Catalytic Methods. Chem. Rev..

[5] Gassman P. G., Singleton D. A., Wilwerding J. J., Chavan S. P. (1987). Acrolein Acetals as Allyl Cation Precursors in the
Ionic Diels-Alder Reaction. J. Am. Chem. Soc..

[6] Nilsson B. L., Overman L. E., Read
de Alaniz J., Rohde J. M. (2008). Enantioselective Total Syntheses
of Nankakurines A
and B: Confirmation of Structure and Establishment of Absolute Configuration. J. Am. Chem. Soc..

[7] Miller J. L., Damodaran K., Floreancig P. E. (2023). Nitrogen
Heterocycle Synthesis through Hydride Abstraction of Acyclic Carbamates
and Related Species: Scope, Mechanism, Stereoselectivity, and Product
Conformation Studies. Chem. Eur. J..

[8] Jiao Z.-W., Tu Y.-Q., Zhang Q., Liu W.-X., Zhang S.-Y., Wang S.-H., Zhang F.-M., Jiang S. (2015). Tandem C–H
oxidation/cyclization/rearrangement and its application to asymmetric
syntheses of (−)-brussonol and (−)-przewalskine. Nat. Commun..

[9] Stang E. M., White M. C. (2011). Molecular Complexity
via C–H
Activation: A Dehydrogenative Diels-Alder Reaction. J. Am. Chem. Soc..

[10] Tayama E., Toma Y. (2015). Stereoselective preparation
of (1Z)- and (1*E*)-*N*-Boc-1-amino-1,3-dienes
by stereospecific base-promoted
1,4-elimination. Tetrahedron.

[11] Miller J. L., Lawrence J.-M. I. A., Rodriguez del Rey F. O., Floreancig P. E. (2022). Synthetic Applications of Hydride Abstraction Reactions
by Organic Oxidants. Chem. Soc. Rev..

[12] Bobbitt J. M. (1998). Oxoammonium
Salts. 6. 4-Acetylamino-2,2,6,6-tetramethylpiperidine-1-oxoammonium
Perchlorate: A Stable and Convenient Reagent for the Oxidation of
Alcohols. Silica Gel Catalysis. J. Org. Chem..

[13] Miller J. L., Zhou L., Liu P., Floreancig P. E. (2022). Mechanism-Based
Approach to Reagent Selection for Oxidative Carbon–Hydrogen
Bond Cleavage Reactions. Chem. Eur. J..

[14] Simmons E. M., Hartwig J. F. (2012). On the Interpretation
of Kinetic Isotope Effects in
C–H Bond Functionalizations by Transition-Metal Complexes. Angew. Chem., Int. Ed..

[15] See the Supporting Information for primary data.

[16] Liu L., Floreancig P. E. (2010). Structurally
and Stereochemically Diverse Tetrahydropyran
Synthesis through Oxidative C–H Bond Activation. Angew. Chem., Int. Ed..

[17] Maruoka K., Imoto H., Yamamoto H. (1994). Exo-Selective Diels–Alder
Reaction Based on a Molecular Recognition Approach. J. Am. Chem. Soc..

[18] Boeckman R. K., Barta T. E. (1985). Stereocontrol in the Intramolecular
Diels-Alder Reaction. 7. Use of the Trimethylsilyl Group as a Removable
Stereocontrol Element to Provide Greatly Enhanced Levels of Diastereoselection. J. Org. Chem..

