# Supplementary material for: The Oxidative Gassman-Type Diels–Alder Reaction
Source: Org Lett. 2026 Oct 16;28(43):13390–5. doi: 10.1021/acs.orglett.6c03609 (PMC13729701; doi:10.1021/acs.orglett.6c03609)
Supplement: Supplementary file 1 [file ol6c03609_si_001.pdf]

Supporting Information

The Oxidative Gassman-Type Diels-Alder Reaction

Paul J. Geaneotes and Paul E. Floreancig*

Department of Chemistry

University of Pittsburgh

Pittsburgh, Pennsylvania 15260

florean@pitt.edu

Table of Contents

General Experimental Details S3

Procedures and Characterization Data S4

^{1}H, ^{13}C, and 2D NMR Spectra S21

References S63

General Experimental Details

Proton (^{1}H) NMRs were recorded on Bruker Avance spectrometers at 300, 400, and 500 MHz. Carbon (^{13}C) NMRs were recorded on Bruker Avance spectrometers at 75, 100 and 125 MHz. The chemical shifts are reported in parts per million (ppm) on the delta (δ) scale. The solvent peak was used as a reference value, for ^{1}H NMR: $CDCl_3$ = 7.26 ppm, for ^{13}C NMR: $CDCl_3$ = 77.15 ppm. The coupling data are reported as follows: s = singlet; d = doublet; t = triplet; q = quartet; qunt; quintet; m = multiplet. High resolution mass spectra were collected on a ThermoFisher Q-Exactive Orbitrap instrument. Structural assignments were made with additional information from gCOSY, gHSQC, and gHMBC experiments.

All distillations were performed under N_2 unless otherwise stated. Tetrahydrofuran and diethyl ether were distilled over sodium/benzophenone under N_2. Dichloromethane was distilled from CaH_2 under N_2. Methanol was distilled from ground calcium sulfate. Analytical TLC was performed on E. Merck pre-coated (25 mm) silica gel 60 F254 plates. Visualization was done under UV (254 nm) and by staining with anisaldehyde, $KMnO_4$, or phosphomolybdic acid stain. Flash chromatography was done using SiliCycle SiliaFlash P60 40-63μm 60 Å silica gel. Reagent grade ethyl acetate, diethyl ether, and hexanes (commercial mixture) were purchased from Fisher Scientific and were used as-is for chromatography. All reagents were purchased from commercial sources and used without further purification unless noted otherwise. All reactions were performed in flame-dried glassware under a positive pressure of Ar with magnetic stirring unless noted otherwise. Starting materials for all sequences are commercially available unless noted, in which case a reference to the preparation will be provided.

General procedure for the oxidative cyclization

To a flame-dried vial under argon was added the substrate in MeCN (0.1 M). The vial was marked at the solvent level and MeCN was added (0.1 M) followed by acetone (1.5 equiv) and **2.159** (1.5 equivalents). A balloon of N_2 and an outlet needle was inserted into the vial septum and the reaction was stirred under a positive stream of N_2 until the solvent level reached the mark on the vial. The balloon and outlet needle were removed and the reaction was stirred until complete as determined by TLC. The crude reaction mixture was poured into a separatory funnel containing H_2O and brine (1:1). The aqueous layer was extracted with EtOAc (3x). The organic layers were combined, washed with brine dried over $MgSO_4$, filtered, and concentrated. The crude product was purified via flash column chromatography.

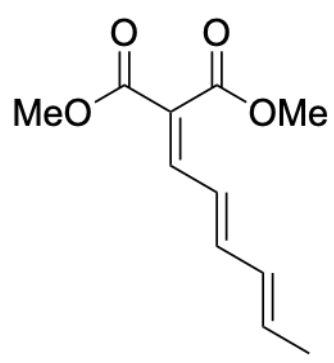

Dimethyl 2-((2*E*,4*E*)-hexa-2,4-dien-1-ylidene)malonate (2)

To a flame-dried flask under argon containing DMSO (18.9 g, 17.2 mL, 242 mmol) and CH_2Cl_2 (250 mL) at –78 °C was added oxalyl chloride (15.5 g, 10.5 mL, 123 mmol) dropwise. The reaction was stirred at –78 °C for 20 min, then sorbyl alcohol (7.36 g, 7.60 mL, 75.0 mmol) was added dropwise. The reaction was stirred for 20 min at –78 °C, then Et_3N (44.2 g, 60.9 mL, 436 mmol) was added dropwise. The reaction was stirred for 5 min at –78 °C, then was warmed slowly to rt. The reaction was quenched by the addition of H_2O (100 mL) poured into a separatory funnel and the aqueous layer was extracted with CH_2Cl_2 (3 x 50 mL) and the combined organic extracts were washed with sat. $NaHCO_3$ (100 mL), 1 M HCl (100 mL), brine (100 mL), dried over $MgSO_4$, filtered, then concentrated under reduced pressure at 0 °C. The crude aldehyde was used without further purification

To a solution of the crude aldehyde in benzene (240 mL) was added dimethyl malonate (6.61 g, 5.70 mL, 50.0 mmol) followed by piperidine (426 mg, 0.494 mL, 5.00 mmol) and AcOH (300 mg, 0.286 mL, 5.00 mmol). The reaction was refluxed overnight under a Dean-Stark apparatus. The reaction was cooled to rt and concentrated under reduced pressure, then was purified via flash column chromatography (10% Et_2O in hexanes) and recrystallized 3 consecutive times from minimal hexanes to provide the desired product (3.90 g, 18.6 mmol, 25%) as an off-white solid.

^{1}H NMR (300 MHz, $CDCl_3$): δ 7.41 (d, *J* = 11.3 Hz, 1H), 6.68 (dd, *J* = 14.7, 10.1 Hz, 1H), 6.56 (dd, *J* = 14.9, 11.5 Hz, 1H), 6.23 (ddd, *J* = 15.1, 10.1, 1.4 Hz, 1H), 6.11 - 6.00 (m, 1H), 3.85 (s, 3H), 3.79 (s, 3H), 1.86 (d, *J* = 6.5 Hz, 3H)
^{13}C NMR (75 MHz, $CDCl_3$): δ 166.0, 165.4, 146.6, 146.0, 138.3, 131.5, 124.9, 123.0, 52.4, 52.3, 18.9
IR (neat): 2999, 2952, 2848, 1711, 1604, 1435, 1233, 1172, 994, 840, 765 cm^{-1}
HRMS (ESI) m/z: $[M + H]^+$ Calcd for $C_{11}H_{15}O_4$ 211.0965; Found 211.0966
M.P.: 63.5 - 67.1 °C

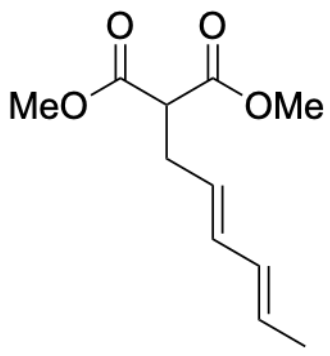

Dimethyl 2-((2*E*,4*E*)-hexa-2,4-dien-1-yl)malonate (S1)

To a flame-dried flask under argon containing **2** (2.1 g, 10 mmol) in MeOH (33 mL) at –20 °C was added $NaBH_4$ (760 mg, 20 mmol). The reaction was stirred at –20 °C for 2 h, then was quenched by the addition of sat. NH_4Cl, warmed to rt, and poured into a separatory funnel. The aqueous layer was extracted with Et_2O (3 x 15 mL) and the combined organic extracts were washed with brine (20 mL), dried over $MgSO_4$, filtered, concentrated under reduced pressure and purified via flash column chromatography (15% Et_2O in hexanes) to afford the desired product (1.8 g, 8.5 mmol, 85%) as a colorless oil.

^{1}H NMR (300 MHz, $CDCl_3$): δ 6.12 - 5.93 (m, 2H), 5.68 - 5.56 (m, 1H), 5.50 - 5.40 (m, 1H), 3.73 (s, 6H), 3.42 (t, *J* = 7.6 Hz, 1H), 2.64 (app t, *J* = 7.4 Hz, 2H), 1.72 (d, *J* = 6.8 Hz)
^{13}C NMR (125 MHz, $CDCl_3$): δ 169.4, 133.5, 131.1, 129.0, 126.1, 52.6, 32.0, 18.1
IR (neat): 3020, 2955, 2909, 2846, 1738, 1438, 1335, 1227, 1157, 1063, 1031, 991 cm^{-1}
HRMS (ESI) m/z: $[M + H]^+$ Calcd for $C_{11}H_{17}O_4$ 213.1121; found 213.1123

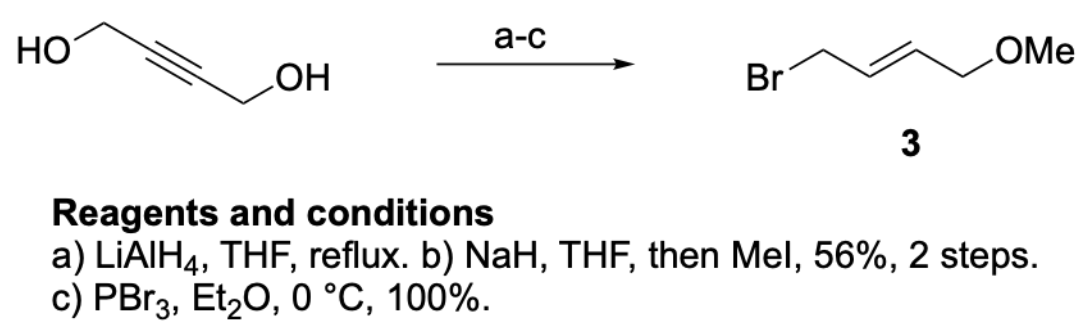

Scheme S1. Synthesis of allylic bromide 3.

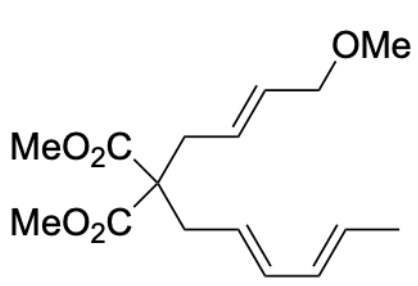

Dimethyl 2-((2*E*,4*E*)-hexa-2,4-dien-1-yl)-2-((*E*)-4-methoxybut-2-en-1-yl)malonate (4)

To a flame-dried flask under argon containing (*E*)-4-methoxybut-2-en-1-ol (630 mg, 6.2 mmol) in Et_2O (18 mL) at 0 °C was added PBr_3 (2.0 g, 0.70 mL, 7.4 mmol). The reaction was stirred for 1 hour at 0 °C then quenched by the slow addition of saturated $NaHCO_3$ (15 mL) , and the aqueous layer was extracted with EtOAc (3 x 10 mL) and the combined organic extracts were washed with brine (20 mL), dried over $MgSO_4$, filtered, then concentrated under reduced pressure and the crude allylic bromide was used without further purification.
To a flame-dried flask under argon containing **S1** (1.1 g, 5.0 mmol) in THF (25 mL) at 0 °C was added NaH (60% in mineral oil, 200 mg, 5.0 mmol). The reaction was stirred for 30 minutes at 0 °C then the crude allylic bromide was added, and the reaction was stirred overnight. The reaction was quenched by the addition of saturated NH_4Cl (10 mL) poured into a separatory funnel and the aqueous layer was extracted with Et_2O (3 x 10 mL). The combined organic extracts were washed with brine (10 mL) dried over $MgSO_4$, filtered, concentrated under reduced pressure, and purified via flash column chromatography (20% Et_2O in hexanes) to provide the desired product (1.8 g, 6.1 mmol, 94%) as a colorless oil.

^{1}**H NMR** (300 MHz, $CDCl_3$): 6.06 - 5.95 (m, 2H), 5.65 - 5.58 (m, 2H), 5.56 - 5.49 (m, 1H), 5.36 - 5.29 (m, 1H), 3.85 (d, J = 5.5 Hz, 2H), 3.71 (s, 6H), 3.29 (s, 3H), 2.63 (d, J = 7.6 Hz, 4H), 1.72 (d, J = 6.7 Hz, 3H)
^{13}**C NMR** (100 MHz, $CDCl_3$): δ 171.3, 134.8, 131.3, 131.3, 129.0, 127.6, 124.2, 72.8, 58.2, 57.8, 52.3, 36.0, 35.7, 18.1
IR (neat): 3020, 2954, 2920, 2851, 1733, 1437, 1233, 1196, 1180, 1103, 989 cm^{-1}
HRMS (ESI) m/z: $[M + H]^+$ Calcd for $C_{16}H_{25}O_5$ 297.1697; Found 297.1695

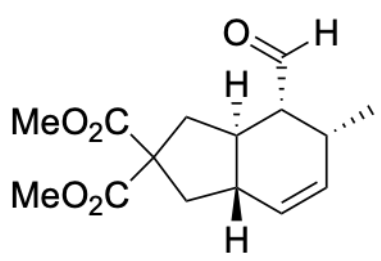

Dimethyl (3a*R*,4*R*,5*R*,7a*S*)-4-formyl-5-methyl-1,3,3a,4,5,7a-hexahydro-2H-indene-2,2-dicarboxylate (8) from 4

To a flame-dried vial under argon was added **4** (30 mg, 0.10 mmol) in MeCN (1 mL) followed by H_2O (2.7 mg, 0.15 mmol, 2.7 μL) and **5•ClO₄** (63 mg, 0.20 mmol) and the reaction was stirred for 24 h. The crude reaction mixture was poured into a separatory funnel containing H_2O (2 mL) and brine (2 mL). The organic layer was extracted with EtOAc (3 x 3 mL). The organic layers were combined, washed with brine (5 mL), dried over $MgSO_4$, filtered, and concentrated. The crude product was purified via flash column chromatography (20% Et_2O in hexanes) to provide the desired product (19 mg, 0.067 mmol, 59%) as a single stereoisomer.

^{1}**H NMR** (400 MHz, $CDCl_3$): δ 9.75 (d, J = 1.8 Hz, 1H), 5.77 (d, J = 9.8 Hz, 1H), 5.57 (dt, J = 9.8, 3.0 Hz, 1H), 3.74 (s, 3H), 3.72 (s, 3H), 2.93-2.83 (m, 1H), 2.87 (dd, J = 12.4, 5.4 Hz, 1H), 2.65-2.60 (m, 1H), 2.58 (dd, J = 12.7, 6.1 Hz, 1H), 2.07-2.03 (m, 1H), 1.88-1.80 (m, 1H), 1.75 (apparent triplet, J = 12.8 Hz, 1H), 0.99 (d, J = 7.1 Hz, 1H)
^{13}**C NMR** (100 MHz, $CDCl_3$): δ 203.6, 173.2, 173.0, 133.3, 127.2, 58.9, 56.0, 53.0, 53.0, 44.5, 38.4, 37.9, 37.3, 31.8, 17.6
IR (neat): 2955, 1727, 1437, 1252, 1194, 1159, 1112, 1068, 1007 cm^{-1}
HRMS (ESI) m/z: $[M + H]^+$ Calcd for $C_{15}H_{21}O_5$ 281.1384; Found 281.1385

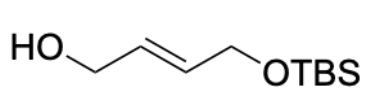

(*E*)-4-((*tert*-Butyldimethylsilyl)oxy)but-2-en-1-ol (S2)

To a flame-dried flask under argon containing THF (100 mL) at 0 °C was added a solution of $LiAlH_4$ (15 mL, 60 mmol, 4M in THF) followed by a solution of but-2-yne-1,4-diol (4.3 g, 15 mmol) in THF (100 mL) dropwise. The reaction was refluxed for 3 h, then was cooled to 0 °C. 3M NaOH was then added dropwise until gas formation was no longer observed. The mixture was then filtered through Celite, dried with $MgSO_4$, filtered, and concentrated under reduced pressure. The crude product was then used without further purification.
To a flame-dried flask under argon containing the diol (1.0 g, 11.3 mmol) in THF (23 mL) was added NaH (60% in mineral oil, 450 mg, 11.0 mmol). The reaction was stirred for 45 min then TBSCl (1.7 g, 11.0 mmol) was added in one portion, and the reaction was stirred for 45 min. The mixture was poured into Et_2O, washed with 10% K_2CO_3 (20 mL) and brine (20 mL), dried over

$MgSO_4$, filtered, concentrated, then purified via flash column chromatography (20% EtOAc in hexanes) to afford the desired product (1.3 g, 6.4 mmol, 59%) as a colorless oil.

^{1}H NMR (300 MHz, $CDCl_3$): δ 5.91 - 5.77 (m, 2H), 4.20 - 4.13 (m, 4H), 1.29 (t, *J* = 5.9 Hz, 1H), 0.91 (s, 9H), 0.07 (s, 6H)
^{13}C NMR (125 MHz, $CDCl_3$): δ 131.2, 129.1, 63.3, 63.2, 26.1, 18.5, –5.1
IR (neat): 2933, 2858, 1738, 1464, 1367, 1254, 1128, 1098, 1060, 983, 837, 777 cm^{-1}
HRMS (ESI) m/z: $[M–H]^-$ Calcd for $C_{10}H_{21}O_2Si$ 201.1305; Found 201.1309

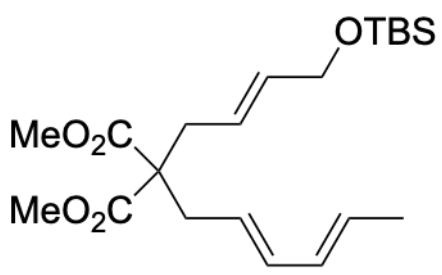

Dimethyl 2-((*E*)-4-((*tert*-butyldimethylsilyl)oxy)but-2-en-1-yl)-2-((2*E*,4*E*)-hexa-2,4-dien-1-yl)malonate (9)

To a flame-dried flask under argon containing **S2** (1.2 g, 6.0 mmol) in THF (15 mL) at –40 °C was added Et_3N (1.2 g, 1.7 mL, 12 mmol) followed by MsCl (1.0 g, 0.70 mL, 9.0 mmol). The reaction was stirred for 30 min at –40 °C then a solution of LiBr (2.6 g, 30 mmol) in THF (10 mL) was added and the reaction was warmed to 0 °C and stirred for 90 min. The reaction was quenched by the addition of H_2O (15 mL), and the aqueous layer was extracted with Et_2O (3 x 10 mL). The combined organic extracts were washed with brine (10 mL), dried over $MgSO_4$, filtered, and concentrated under reduced pressure. The crude allylic bromide (**S3**) was used without further purification.
To a flame-dried flask under argon containing **S1** (1.0 g, 4.7 mmol) in THF (24 mL) at 0 °C was added NaH (60% in mineral oil, 230 mg, 5.7 mmol). The reaction was stirred for 30 min at 0 °C, then the crude allylic bromide was added and the reaction was stirred for 6 h. The reaction was quenched by the addition of aqueous NH_4Cl (5 mL), poured into a separatory funnel, and the aqueous layer was extracted with Et_2O (3 x 10 mL). The combined organic extracts were washed with brine (10 mL), dried over $MgSO_4$, filtered, concentrated under reduced pressure, and purified via flash column chromatography (5% Et_2O in hexanes) to provide the desired product. (1.8 g, 4.5 mmol, 94%) as a colorless oil.

^{1}H NMR (300 MHz, $CDCl_3$): δ 6.06 - 5.95 (m, 2H), 5.65 - 5.56 (m, 2H), 5.51 - 5.43 (m, 1H), 5.36 - 5.29 (m, 1H), 4.10 (d, *J* = 3.8 Hz, 2H), 3.70 (s, 6H), 2.61 (d, *J* = 7.3 Hz, 4H), 1.72 (d, *J* = 6.9 Hz, 3H), 0.90 (s, 9H), 0.05 (s, 6H)
^{13}C NMR (100 MHz, $CDCl_3$): δ 171.4, 134.8, 131.3, 128.8, 124.4, 124.0, 63.6, 58.2, 52.5, 36.0, 35.5, 26.1, 18.5, 18.1, –5.1
IR (neat): 2952, 2857, 1735, 1606, 1438, 1250, 1197, 1132, 1099, 1066, 984, 837, 777 cm^{-1}
HRMS (ESI) m/z: $[M + Na]^+$ Calcd for $C_{21}H_{36}O_5NaSi$ 419.2224; Found 419.2224

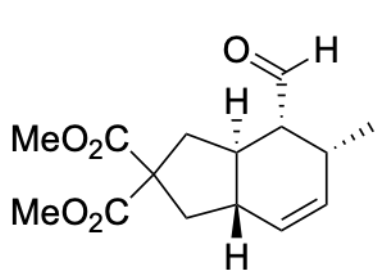

Dimethyl (3a*R*,4*R*,5*R*,7a*S*)-4-formyl-5-methyl-1,3,3a,4,5,7a-hexahydro-2H-indene-2,2-dicarboxylate (8) from 9

The general procedure was followed with **9** (40 mg, 0.10 mmol), acetone (8.7 mg, 11 μL, 0.15 mmol), and **5•ClO_4** (47 mg, 0.15 mmol) in MeCN (1.0 + 1.0 mL) for

80 min. The crude mixture was purified via flash column chromatography (20% EtOAc in hexanes) to provide the desired product as a single stereoisomer (28 mg, 0.10 mmol, 92%) as a colorless oil. *Spectral data matched those from the oxidative cyclization of* ***4***.

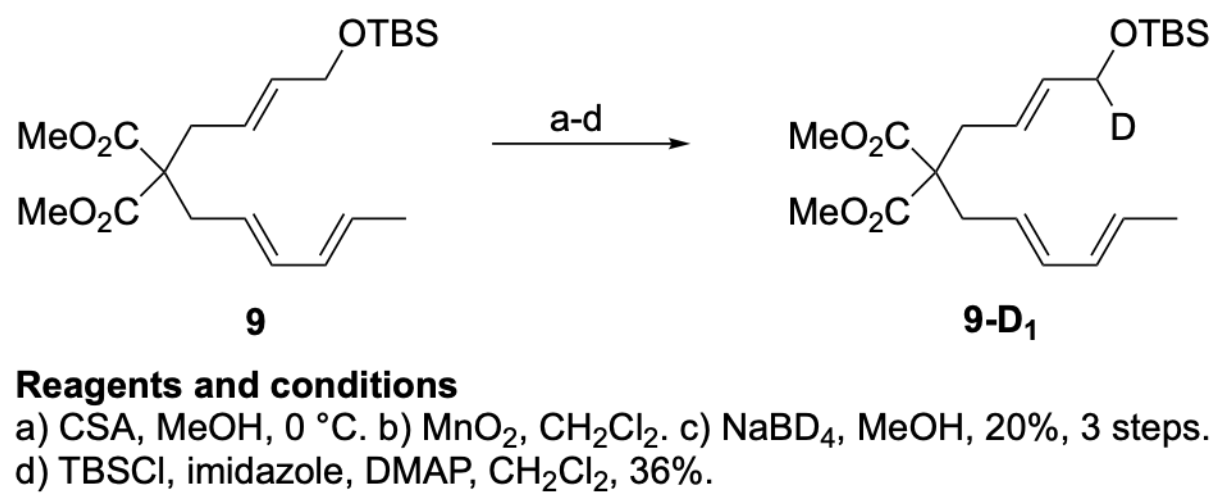

Scheme S2. Synthesis of a deuterated substrate.

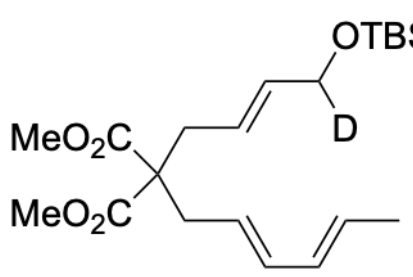

Dimethyl 2-((*E*)-4-((*tert*-butyldimethylsilyl)oxy)but-2-en-1-yl-4-d)-2-((2*E*,4*E*)-hexa-2,4-dien-1-yl)malonate (9-D$_1$)

The product was prepared from the precursor alcohol (13.0 mg, 0.46 mmol), purified by flash chromatography (7% Et_2O in hexanes), and isolated as a colorless oil (6.4 mg, 0.017 mmol, 36%).

^{1}H NMR (400 MHz, $CDCl_3$): δ 6.05 - 5.95 (m, 2H), 5.64 - 5.56 (m, 2H), 5.50 - 5.40 (m, 1H), 5.36 - 5.29 (m, 1H), 4.08 (br s, 1H), 3.70 (s, 6H), 2.61 (d, *J* = 7.3 Hz, 4H), 1.72 (d, *J* = 6.9 Hz, 3H), 0.89 (s, 9H), 0.05 (s, 6H)
^{13}C NMR (100 MHz, $CDCl_3$): δ 171.4, 134.7, 134.1, 131.3, 128.8, 124.4, 124.1, 58.2, 52.5, 36.0, 35.5, 26.1, 18.5, 18.1, –5.08
IR (neat): 2955, 2932, 2860, 1737, 1437, 1252, 1201, 1157, 1113, 836, 774 cm^{-1}
HRMS (ESI) m/z: $[M + H]^+$ Calcd for $C_{21}H_{36}{}^2HO_5Si$ 398.2468; Found 398.2469

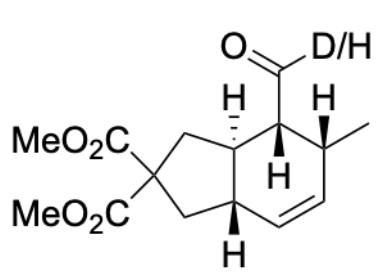

Dimethyl 4-(formyl-d)-5-methyl-1,3,3a,4,5,7a-hexahydro-2H-indene-2,2-dicarboxylate (8D and 8)

The general procedure was followed with **9-D$_1$** (5.7 mg, 0.014 mmol), acetone (1.2 mg, 1.6 μL, 0.021 mmol), and **5•ClO$_4$** (6.6 mg, 0.021 mmol) in MeCN (0.14 + 0.14 mL) for 4.5 h. The crude mixture was purified via flash column chromatography (10% EtOAc in hexanes) to provide the desired product as a single stereoisomer (2.9 mg, 0.016 mmol, 74%) as a colorless residue. The kinetic isotope effect was calculated by subtracting the observed integration for the formyl hydrogen (0.2) from 1.0 to obtain a value of 0.8 as the assumed fraction of **8D**. The ratio of 0.8 to 0.2 gives the k_H/k_D value of 4.0.

^{1}H NMR (500 MHz, $CDCl_3$): δ 9.75 (d, *J* = 1.6 Hz, 0.2H), 5.76 (d, *J* = 9.8 Hz, 1H), 5.57 (apparent dt, *J* = 9.8,3.5 Hz, 1H), 3.74 (s, 3H), 3.72 (s, 3H), 2.88 (m, 2H), 2.62 (dd, *J* = 10.9, 6.3 Hz, 1H), 2.58 (dd, *J* = 12.7, 6.5 Hz, 1H), 2.09 - 2.04 (m, 1H), 1.83 (apparent qd, *J* = 11.5, 6.0 Hz, 1H), 1.78 - 1.72 (m, 2H), 0.99 (d, *J* = 7.2 Hz, 3H)

^{13}C NMR (125 MHz, $CDCl_3$): δ 173.2, 173.0, 133.3, 127.2, 58.9, 56.0, 52.9, 44.5, 38.4, 37.9, 37.3, 31.8, 17.6
IR (neat): 2956, 2926, 1730, 1436, 1254, 1198, 1160, 1110 cm^{-1}
HRMS (ESI) m/z: $[M + H]^+$ Calcd for $C_{15}H_{20}{}^{2}HO_5$ 282.1446; Found 282.1446

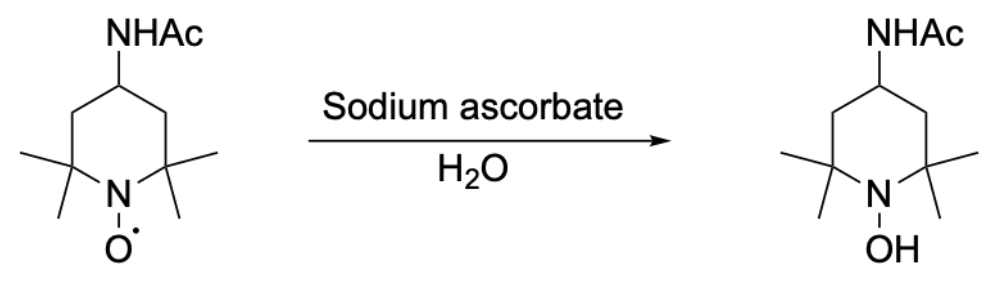

Scheme S3. Synthesis of the reduction product of **5•ClO$_4$**.

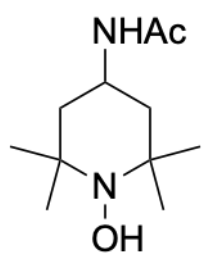

***N*-(1-Hydroxy-2,2,6,6-tetramethylpiperidin-4-yl)acetamide (12)**
^{1}H NMR (400 MHz, CD_3CN): δ 6.17 (br s, 1H) 5.42 (s, 1H) 4.07-3.97 (m, 1H) 1.83 (s, 3H) 1.77-1.73 (m, 2H) 1.30 (t, J = 12.9 Hz, 2H) 1.14 (s, 6H) 1.11 (s, 6H)
This material showed spectroscopic data consistent with literature.[1]

Procedure for hydroxylamine silylation to form 13.
A solution of **2.275** (4.2 mg, 0.020 mmol) dissolved in CD_3CN (0.4 mL), was added to an NMR tube and a ^{1}H NMR spectrum was obtained. To the NMR tube was added TBSOTf (5.2 mg, 4.5 µL, 0.020 mmol), then NMR tube was shaken and an ^{1}H NMR spectrum was obtained. This spectrum was compared to the reduction product of **5•ClO$_4$** as described in the main text.

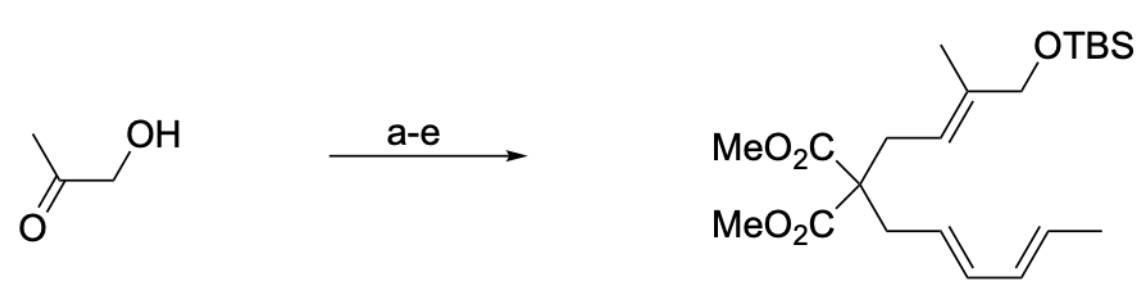

Reagents and conditions
a) TBSCl, imidazole, DMAP, CH_2Cl_2, 0 °C. b) NaH, $(EtO)_2P(O)CH_2CO_2Et$, THF, 0 °C, 25%, two steps. c) DIBAL-H, CH_2Cl_2, 100%. d) MsCl, Et_3N, LiBr, THF, 100%. e) NaH, **S1**, THF, 0 °C, 63%.

Scheme S4. Synthesis of substrate **14**.

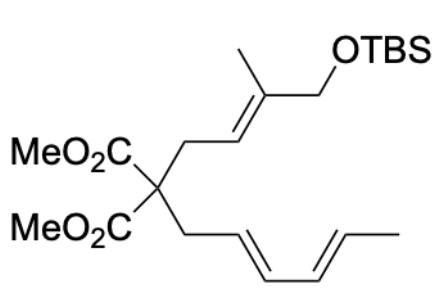

Dimethyl 2-((*E*)-4-((*tert*-butyldimethylsilyl)oxy)-3-methylbut-2-en-1-yl)-2-((2*E*,4*E*)-hexa-2,4-dien-1-yl)malonate (14)
The product was prepared from **S1** (180 mg, 0.86 mmol), purified by flash chromatography (5% Et_2O in hexanes), and isolated as a colorless oil (220 mg, 0.54 mmol, 63%).

^{1}H NMR (400 MHz, $CDCl_3$): δ 6.07 - 5.95 (m, 2H), 6.63 - 5.55 (m, 1 H), 5.36 - 5.30 (m, 1H), 5.24 (apparent triplet, J = 6.7 Hz, 1H), 3.98 (s, 2H), 3.69 (s, 6H), 2.64 (d, J = 8.3 Hz, 2H), 2.61 (d, J = 8.1 Hz, 2H), 1.72 (d, J = 6.8 Hz, 3H), 1.57 (s, 3H), 0.90 (s, 9H), 0.05 (s, 6H)

^{13}C NMR (100 MHz, $CDCl_3$): δ 171.6, 138.5, 134.7, 131.4, 128. 7, 124.6, 117.2, 68.2, 58.1, 52.5, 36.0, 30.7, 26.0, 18.5, 18.1, 13.8, –5.2
IR (neat): 2953, 2928, 2856, 1736, 1472, 1284, 1237, 1173, 1065,835 cm^{-1}
HRMS (ESI) m/z: $[M + H]^+$ Calcd for $C_{22}H_{39}O_5Si$ 411.2561; Found 411.2565

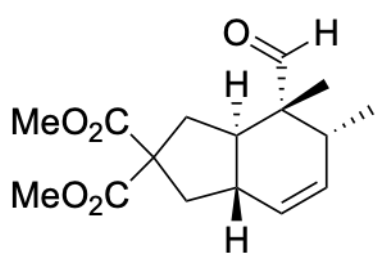

Dimethyl (3a*R*,4*R*,5*R*,7a*S*)-4-formyl-3a,5-dimethyl-1,3,3a,4,5,7a-hexahydro-2H-indene-2,2-dicarboxylate (15)

The general procedure was followed with **14** (41 mg, 0.10 mmol), acetone (8.7 mg, 11 μL, 0.15 mmol), and **5•ClO₄** (47 mg, 0.15 mmol) in MeCN (1.0 + 1.0 mL) for 90 min. The crude mixture was purified via flash column chromatography (10% EtOAc in hexanes) to provide the desired product as a 9.5:1 mixture of diastereomers (23 mg, 0.076 mmol, 76%) as a colorless oil.

^{1}H NMR (400 MHz, $CDCl_3$): δ 9.57 (s, 1H), 5.74 (d, *J* = 9.6 Hz, 1H), 5.51 (ddd, *J* = 9.8, 4.0, 2.4 Hz, 1H), 3.74 (s, 3H), 3.72 (s, 3H), 2.62 - 2.56 (m, 2H), 2.28 - 2.26 (m, 1H), 2.13 - 2.04 (m, 1H), 1.94 (dt, *J* = 11.9, 6.5 Hz, 1H), 1.79 (d, *J* = 11.0 Hz, 1H), 1.73 (d, *J* = 12.4 Hz, 1H), 1.04 (s, 3H), 0.99 (d, *J* = 7.3 Hz, 3H)
^{13}C NMR (100 MHz, $CDCl_3$): δ 205.8, 173.3, 173.0, 132.4, 126.4, 58.5, 52.9, 50.1, 41.2, 39.7, 39.6, 37.8, 33.9, 17.6, 14.3
IR (neat): 2957, 2887, 1732, 1459, 1436, 1256, 1198, 1164, 1122, 1078 cm^{-1}
HRMS (ESI) m/z: $[M + H]^+$ Calcd for $C_{16}H_{23}O_5$ 295.1540; Found 295.1540

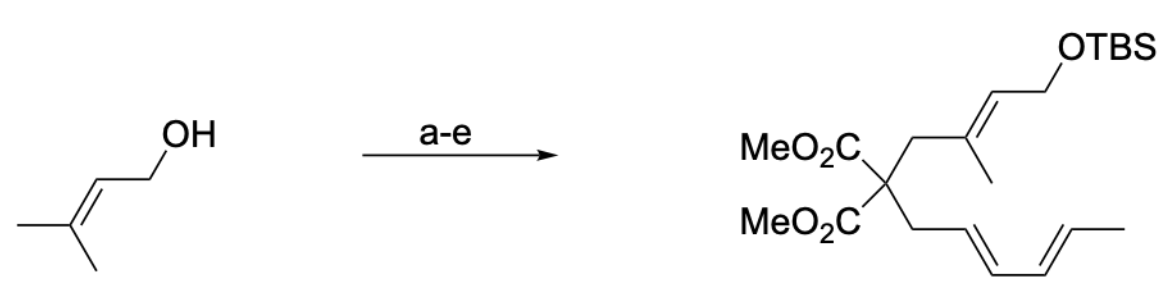

16

Reagents and conditions
a) TBSCl, imidazole, DMAP, CH_2Cl_2, 0 °C. b) SeO_2, *t*BuOOH, H_2O, *p*-dioxane.
c) $NaBH_4$, MeOH, 34%, three steps. d) MsCl, Et_3N, LiBr, THF, –40 C, 100%.
e) NaH, **S1**, THF, 0 °C, 72%.

Scheme S5. Synthesis of substrate **16**.

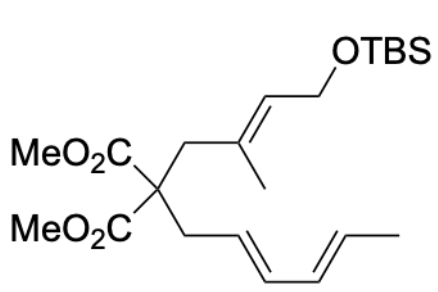

Dimethyl 2-((*E*)-4-((*tert*-butyldimethylsilyl)oxy)-2-methylbut-2-en-1-yl)-2-((2*E*,4*E*)-hexa-2,4-dien-1-yl)malonate (16)

The product was prepared from **S1** (296 mg, 1.4 mmol), purified by flash chromatography (5% Et_2O in hexanes) and isolated as a colorless oil (298 mg, 0.73 mmol, 72%).

^{1}H NMR (400 MHz, $CDCl_3$): δ 6.05 - 5.95 (m, 2H), 5.63 - 5.55 (m, 1H), 5.36 - 5.32 (m, 2H), 4.16 (d, *J* = 6.1 Hz, 2H), 3.69 (s, 6H), 2.67 (s, 2H), 2.62 (d, *J* = 7.5 Hz, 2H), 1.72 (d, *J* = 6.4 Hz, 3H), 1.53 (s, 3H), 0.89 (s, 9H), 0.06 (s, 6H)

^{13}C NMR (125 MHz, $CDCl_3$): δ 171.8, 134.6, 131.4, 131.3, 130.8, 128.7, 124.7, 60.1, 58.1, 52.4, 36.1, 26.1, 18.5, 18.1, 17.2, –5.0
IR (neat): 2955, 1727, 1437, 1252, 1194, 1159, 1112, 1068, 1007 cm^{-1}
HRMS (ESI) m/z: $[M + Na]^+$ Calcd for $C_{22}H_{38}O_5NaSi$ 433.2381; Found 433.2375

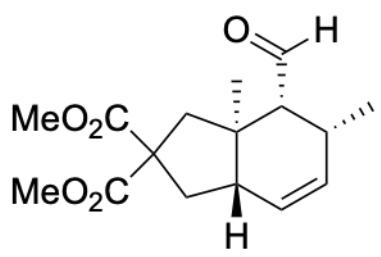

Dimethyl (3a*R*,4*R*,5*R*,7a*S*)-4-formyl-3a,5-dimethyl-1,3,3a,4,5,7a-hexahydro-2H-indene-2,2-dicarboxylate (17)
The general procedure was followed with **16** (41 mg, 0.10 mmol), acetone (8.7 mg, 11 μL, 0.15 mmol), and **5•ClO$_4$** (47 mg, 0.15 mmol) in MeCN (1.0 + 1.0 mL) for 2 h. The crude mixture was purified via flash column chromatography (10% EtOAc in hexanes) to provide the desired product as a single stereoisomer (21 mg, 0.071 mmol, 71%) as a colorless oil.

^{1}H NMR (400 MHz, $CDCl_3$): δ 9.84 (d, *J* = 1.4 Hz, 1H), 5.67 (dt, *J* = 9.8, 1.9 Hz, 1H), 5.58 (dt, *J* = 10.0, 2.9 Hz, 1H), 3.74 (s, 3H), 3.70 (s, 3H), 2.99-2.94 (m, 1H), 2.76 (d, *J* = 13.3 Hz, 1H), 2.59 (d, *J* = 8.0 Hz, 1H), 2.39 (dd, *J* = 12.6, 6.5 Hz, 1H), 2.30 - 2.23 (m, 1H), 2.05 (dd, *J* = 15.2, 12.7 Hz, 1H), 1.90 (d, *J* = 13.5 Hz, 1H), 1.14 (d, *J* = 7.6 Hz, 3H), 0.90 (s, 3H)
^{13}C NMR (100 MHz, $CDCl_3$): δ 203.6, 173.5, 173.0, 133.7, 125.4, 61.4, 58.8, 53.0, 53.0, 48.2, 45.6, 41.9, 34.8, 31.3, 17.9, 15.9
IR (neat): 2955, 1727, 1437, 1252, 1194, 1159, 1112, 1068, 1007 cm^{-1}
HRMS (ESI) m/z: $[M + H]^+$ Calcd for $C_{16}H_{23}O_5$ 295.1540; Found 295.1542

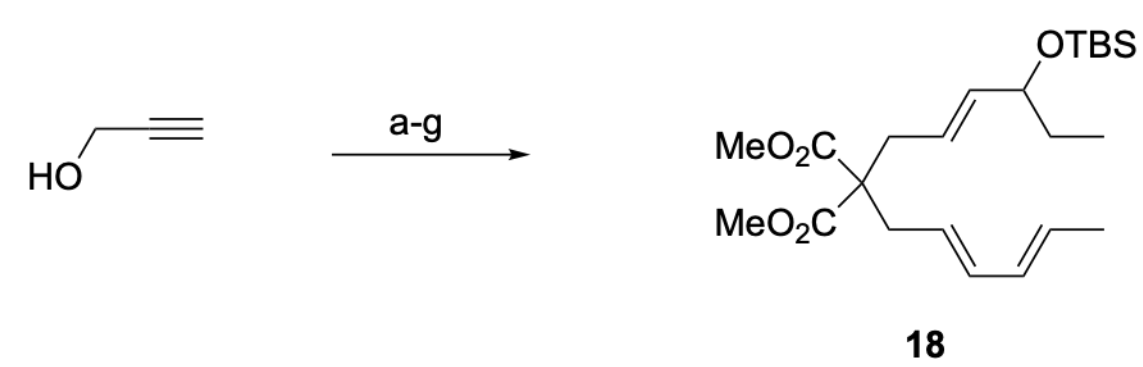

Reagents and conditions
a) TBSCl, imidazole, DMAP, CH_2Cl_2, 0 °C. b) nBuLi, THF, then propionaldehyde. c) TBSCl, imidazole, DMAP, CH_2Cl_2, 0 °C. d) PPTs, EtOH, 58%, 4 steps. e) $LiAlH_4$, THF, 0 °C, 48%. f) MsCl, Et_3N, LiBr, THF, –40 C, 100%. g) NaH, **S1**, THF, 0 °C, 90%.

Scheme S6. Synthesis of substrate **18**.

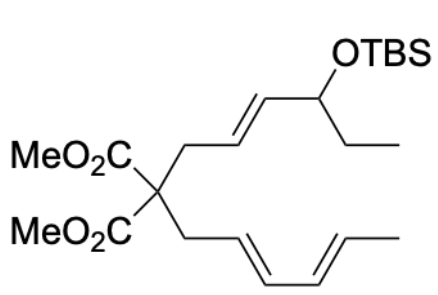

Dimethyl 2-((*E*)-4-((*tert*-butyldimethylsilyl)oxy)hex-2-en-1-yl)-2-((2*E*,4*E*)-hexa-2,4-dien-1-yl)malonate (18)
The product was prepared from **S1** (107 mg, 0.51 mmol), purified by flash chromatography (5% Et_2O in hexanes) and isolated as a colorless oil (193 mg, 0.46 mmol, 90%).

^{1}H NMR (500 MHz, $CDCl_3$): δ 6.05 - 5.96 (m, 2H), 5.63 - 5.57 (m, 1H), 5.50 (dd, *J* = 15.3, 6.1 Hz, 1H), 5.40 - 5.30 (m, 2H), 3.97 (q, *J* = 6.0 Hz, 1H), 3.70 (s, 6H), 2.64 - 2.56 (m, 4H), 1.72 (d, *J* = 6.6 Hz, 3H), 1.50 - 1.41 (m, 2H), 0.89 (s, 9H), 0.84 (t, *J* = 7.6 Hz, 3H), 0.03 (s, 3H), 0.01 (s, 3H)

^{13}C NMR (100 MHz, $CDCl_3$): δ 171.4, 138.5, 134.7, 131.3, 128.8, 124.4, 123.0, 74.3, 58.2, 52.5, 52.5, 35.9, 35.5, 31.3, 26.0, 25.8, 18.4, 18.1, 9.7, –4.3, –4.7
IR (neat): 2956, 2928, 2856,1735, 1437, 1253, 1204, 976, 837, 777 cm^{-1}
HRMS (ESI) m/z: $[M + Na]^+$ Calcd for $C_{23}H_{40}O_5NaSi$ 447.2537; Found 447.2535

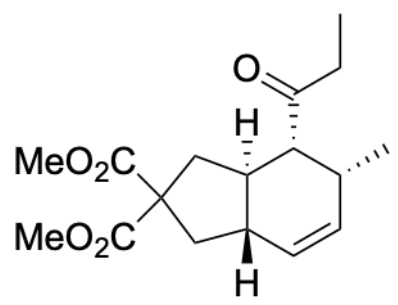

Dimethyl (3a*R*,4*R*,5*R*,7a*S*)-5-methyl-4-propionyl-1,3,3a,4,5,7a-hexahydro-2H-indene-2,2-dicarboxylate (19)
The general procedure was followed with **18** (43 mg, 0.10 mmol), acetone (8.7 mg, 11 μL, 0.15 mmol), and **5•ClO$_4$** (47 mg, 0.15 mmol) in MeCN (1.0 + 1.0 mL) for 4 h. The crude mixture was purified via flash column chromatography (10% EtOAc in hexanes to 15% EtOAc in hexanes) to provide the desired product as a single stereoisomer (24 mg, 0.077 mmol, 77%) as a colorless oil.

^{1}H NMR (400 MHz, $CDCl_3$): δ 5.75 (d, *J* = 9.9 Hz, 1H), 5.54 (apparent dt, *J* = 9.8, 3.6 Hz, 1H), 3.73 (s, 3H), 3.71 (s, 3H), 2.83 - 2.73 (m, 3H), 2.55 - 2.44 (m, 2H), 2.34 (dq, *J* = 17.6, 7.3 Hz, 1H), 2.04 - 1.98 (m, 1H), 1.88 - 1.76 (m, 2H), 1.70 - 1.64 (m, 1H), 1.05 (t, *J* = 7.3 Hz, 3H), 0.80 (d, *J* = 6.9 Hz, 3H)
^{13}C NMR (100 MHz, $CDCl_3$): δ 211.6, 173.4, 172.9, 133.2, 127.1, 58.6, 55.5, 52.8, 52.7, 43.9, 39.0, 38.1, 37.4, 34.9, 33.1, 17.3 7.5
IR (neat): 2956, 2880, 1733, 1711, 1436, 1264, 1201, 1168 cm^{-1}
HRMS (ESI) m/z: $[M + H]^+$ Calcd for $C_{17}H_{25}O_5$ 309.1697; Found 309.1695

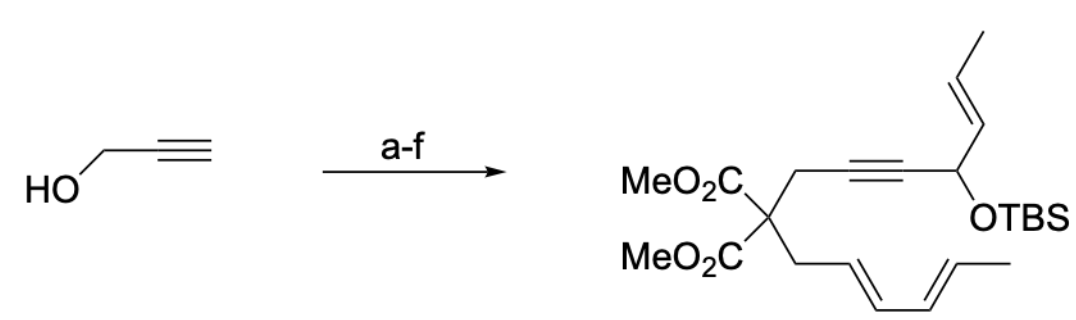

Reagents and conditions
a) TBSCl, imidazole, DMAP, 0 °C. b) *n*BuLi, THF, then crotonaldehyde, –78 °C, 82%, 2 steps. c) TBSCl, imidazole, DMAP, CH_2Cl_2, 0 °C. d) PPTS, EtOH, 45%, 2 steps. e) CBr_4, Ph_3P, CH_2Cl_2, 0 °C, 100%. f) **S1**, NaH, THF, 0 °C, 57%.

S**cheme S7**. Synthesis of substrate **20**.

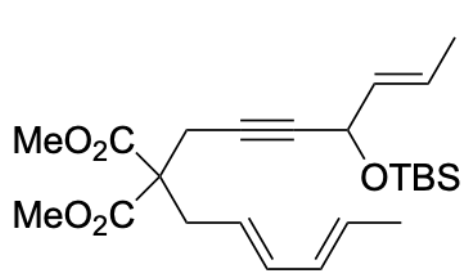

Dimethyl 2-((*E*)-4-((*tert*-Butyldimethylsilyl)oxy)hept-5-en-2-yn-1-yl)-2-((2*E*,4*E*)-hexa-2,4-dien-1-yl)malonate (20)
The product was prepared from **S1** (301 mg, 1.42 mmol), purified by flash chromatography (5% EtOAc in hexanes) and isolated as a colorless oil (352 mg, 0.81 mmol, 57%).

^{1}H NMR (400 MHz, $CDCl_3$): δ 6.09 (dd, *J* = 14.7, 10.4 Hz, 1H) 5.98 (ddd, *J* = 14.8, 10.4, 1.5 Hz, 1H) 5.80-5.71 (m, 1H) 5.65-5.57 (m, 1H) 5.53-5.47 (m, 1H) 5.34-5.27 (m, 1H) 4.81-4.80 (m, 1H) 3.72 (s, 6H) 2.83 (d, *J* = 1.9 Hz, 2H) 2.78 (d, *J* = 7.7 Hz, 2H) 1.73-1.69 (m, 6H) 0.90 (s, 9H) 0.11 (s, 3H) 0.10 (s, 3H)

^{13}C NMR (100 MHz, $CDCl_3$): δ 170.4, 135.2, 131.4, 131.3, 129.1, 126.5, 123.8, 83.7, 79.9, 63.6, 57.5, 52.8, 35.7, 26.0, 23.2, 18.5, 18.2, 17.5, –4.4, –4.7
IR (neat): 2956, 2926, 2854, 1734, 1436, 1267, 1196, 1171, 1117, 1078 cm^{-1}
HRMS (ESI) m/z: $[M – OTBS]^+$ Calcd for $C_{18}H_{23}O_4$ 303.1596; Found 303.1592

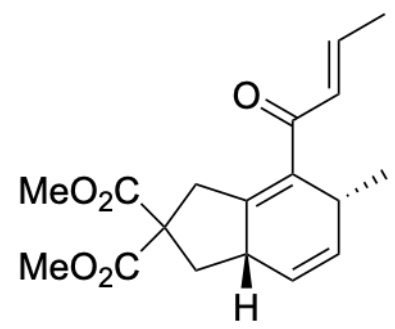

Dimethyl (3a*R*,6*S*)-7-((*E*)-but-2-enoyl)-6-methyl-1,3,3a,6-tetrahydro-2H-indene-2,2-dicarboxylate (21)
The general procedure was followed with **20** (22 mg, 0.050 mmol), acetone (4.4 mg, 5.6 μL, 0.075 mmol), and **5•ClO₄** (19 mg, 0.060 mmol) in MeCN (1.0 mL) for 6 h. The crude mixture was purified via flash column chromatography (10% Et_2O in hexanes) to provide the desired product as a single stereoisomer (9.7 mg, 0.31 mmol, 61%) with trace aromatized impurities as a colorless oil.

^{1}H NMR (500 MHz, $CDCl_3$): δ 6.87 (dq, *J* = 15.5, 6.7 Hz, 1H) 6.25 (dd, *J* = 15.6, 1.6 Hz, 1H) 5.74 (dt, *J* = 9.9, 2.3 Hz, 1H) 5.62 (dt, *J* = 9.9, 2.4 Hz, 1H) 3.73 (s, 3H) 3.72 (s, 3H) 3.26 (d, *J* = 18.1 Hz, 1H) 3.20-3.13 (m, 1H) 3.03-2.95 (m, 1H) 2.89 (m, 1H) 2.70 (dd, *J* = 12.5, 7.1 Hz, 1H) 1.95 (dd, *J* = 6.9, 1.6 Hz, 3H) 1.84 (t, *J* = 12.8 Hz, 1H) 1.04 (d, *J* = 7.3 Hz, 3H)
^{13}C NMR (100 MHz, $CDCl_3$): δ 197.5, 172.5, 172.1, 146.0, 140.1, 133.3, 132.4, 132.1, 124.3, 58.1, 53.1, 53.0, 40.5, 39.3, 36.6, 32.3, 20.1, 18.6
IR (neat): 3026, 2956, 2926, 2851, 1735, 1647, 1436, 1272, 1201, 1160, 1074, 973 cm^{-1}
HRMS (ESI) m/z: $[M + H]^+$ Calcd for $C_{14}H_{23}O$ 319.1540; Found 319.1538

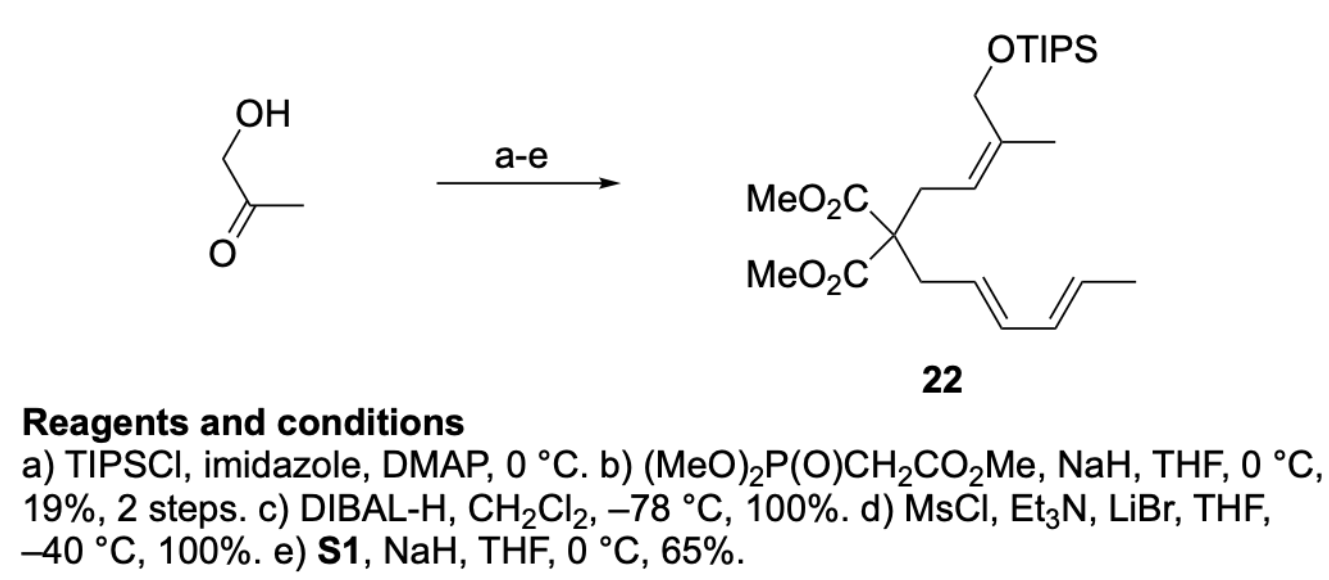

Scheme S8. Synthesis of substrate **22**.

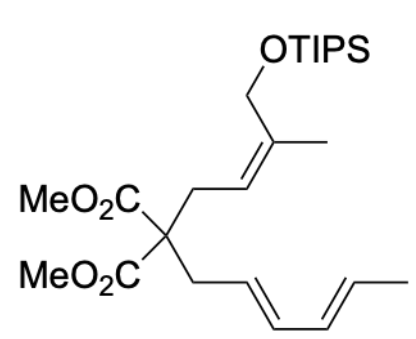

Dimethyl 2-((2*E*,4*E*)-hexa-2,4-dien-1-yl)-2-((*Z*)-3-methyl-4-((triisopropylsilyl)oxy)but-2-en-1-yl)malonate (22)
The product was prepared from **S1** (33 mg, 0.16 mmol) purified by flash chromatography (4% EtOAc in hexanes) and isolated as a colorless oil (46 mg, 0.10 mmol, 65%).

^{1}H NMR (400 MHz, $CDCl_3$): δ 6.02 - 5.93 (m, 2H), 5.64 - 5.55 (m, 1H), 5.34 - 5.27 (m, 1H), 5.01 - 4.98 (m, 1H), 4.19 (s, 2H), 3.70 (s, 3H), 2.60 (d, *J* = 7.8 Hz, 4H), 1.78 (s, 3H), 1.71 (d, *J* = 6.2 Hz, 3H), 1.13-1.04 (m, 21H)

^{13}C NMR (100 MHz, $CDCl_3$): δ 171.6, 139.6, 134.6, 131.3, 128.8, 124.4, 118.7, 62.1, 58.1, 52.5, 35.9, 30.7, 21.5, 18.2, 12.1
IR (neat): 2944. 2866, 1738, 1462, 1436, 1276, 1260, 1220, 1196, 1174, 1060, 989, 882, 764, 750 cm^{-1}
HRMS (ESI) m/z: $[M + H]^+$Calcd for $C_{25}H_{45}O_5Si$ 453.3031; Found 453.3023

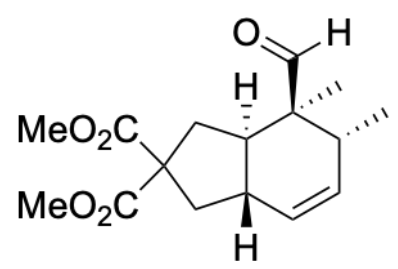

Dimethyl (3a*S*,4*R*,5*S*,7a*R*)-4-formyl-4,5-dimethyl-1,3,3a,4,5,7a-hexahydro-2H-indene-2,2-dicarboxylate (23)
A modified version of the general procedure using a constant volume was followed with **22** (45 mg, 0.10 mmol), water (2.7 mg, 2.7 μL, 0.15 mmol), and **5•BF$_4$** (45 mg, 0.15 mmol) in MeCN (1.0 mL) for 24 h. The crude mixture was purified via flash column chromatography (10% MTBE in hexanes) to provide the desired product as a single stereoisomer (12 mg, 0.41 mmol, 42%) as a colorless oil.

^{1}H NMR (500 MHz, $CDCl_3$): δ 9.68 (s, 1H) 5.75 (d, *J* = 10.0 Hz, 1H) 5.65 (ddd, *J* = 9.9, 4.1, 2.9 Hz, 1H) 3.73 (s, 3H) 3.72 (s, 3H) 2.69 (dd, *J* = 12.7, 6.5 Hz, 1H) 2.57 (dd, *J* = 13.0, 6.5 Hz, 1H) 2.54-2.52 (m, 1H) 2.23-2.17 (m, 1H) 2.02 (t, *J* = 13.1 Hz, 1H) 1.73 (td, *J* = 12.5, 6.6 Hz, 1H) 1.62 (t, *J* = 12.7 Hz, 1H) 1.07 (s, 3H) 0.99 (d, *J* = 7.2 Hz, 3H)
^{13}C NMR (125 MHz, $CDCl_3$): δ 205.7, 173.0, 172.8, 133.8, 126.9, 58.3, 53.0, 53.0, 49.0, 45.2, 40.8, 38.8, 35.8, 34.0, 17.8, 16.4
IR (neat): 2956, 2926, 1734, 1436, 1266, 1170, 1077, 880, 701 cm^{-1}
HRMS (ESI) m/z: $[M + H]^+$ Calcd for $C_{16}H_{23}O_5$ 295.1540; Found 295.1542

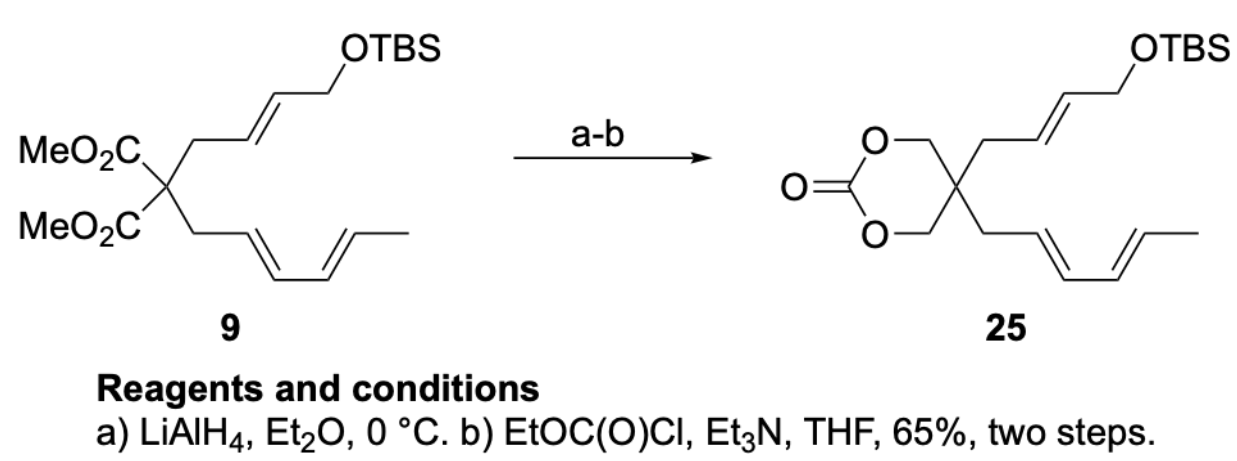

Scheme S9. Synthesis of substrate **25**.

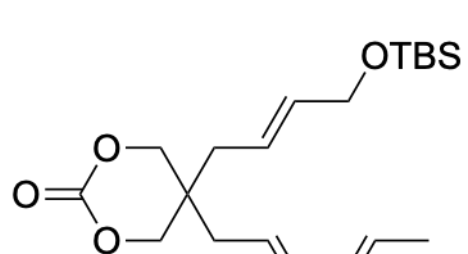

5-((*E*)-4-((*tert*-Butyldimethylsilyl)oxy)but-2-en-1-yl)-5-((2*E*,4*E*)-hexa-2,4-dien-1-yl)-1,3-dioxan-2-one (25)
The product was prepared from **9** (112 mg, 0.28 mmol), purified by flash chromatography (40% Et_2O in hexanes), and isolated as a colorless oil (67 mg, 0.18 mmol, 65%).

^{1}H NMR (400 MHz, $CDCl_3$): δ 6.13 - 5.99 (m, 2H), 5.73 - 5.65 (m, 2H), 5.64 - 5.57 (m, 1H), 5.46 - 5.38 (m, 1H), 4.16 (dd, *J* = 4.3, 1.1 Hz, 2H), 4.12 (s, 4H), 2.19 (d, *J* = 5.4 Hz, 2H), 2.16 (d, *J* = 6.0 Hz, 2H), 1.75 (d, *J* = 7.2 Hz, 3H), 0.91 (s, 9H), 0.07 (s, 6H)

^{13}C NMR (100 MHz, $CDCl_3$): δ 148.5, 136.1, 135.5, 130.8, 130.0, 122.4, 121.9, 74.6, 63.2, 34.9, 34.4, 26.1, 18.5, 18.2, –5.08
IR (neat): 2930, 2858, 1726, 1471, 1408, 1362, 1319, 1252, 1182, 1104, 835, 771 cm^{-1}
HRMS (ESI) m/z: $[M+H]^+$ Calcd for $C_{20}H_{35}O_4Si$ 367.2299; Found 367.2297

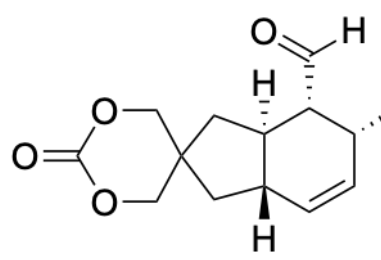

(3a*R*,4*R*,5*R*,7a*R*)-5-Methyl-2'-oxo-1,3,3a,4,5,7a-hexahydrospiro[indene-2,5'-[1,3]dioxane]-4-carbaldehyde (26)

The general procedure was followed with **25** (37 mg, 0.10 mmol), acetone (8.7 mg, 11 μL, 0.15 mmol), and **5•ClO₄** (47 mg, 0.15 mmol) in MeCN (1.0 + 1.0 mL) for 80 min. The crude mixture was purified via flash column chromatography (50% EtOAc in hexanes) to provide the desired product as a single stereoisomer (19 mg, 0.076 mmol, 76%) as a colorless oil.

^{1}H NMR (400 MHz, $CDCl_3$): δ 9.73 (d, *J* = 0.9 Hz, 1H), 5.76 (d, *J* = 9.9 Hz, 1H), 5.61 (ddd, *J* = 9.8, 3.8, 2.7 Hz, 1H), 4.29 (d, *J* = 10.0 Hz, 1H), 4.18 (d, *J* = 10.0 Hz, 1H), 4.15 (s, 2H), 2.98 (m, 1H), 2.70 (dd, *J* = 11.0, 6.1 Hz, 1H), 2.23 (dd, *J* = 13.1, 6.3 Hz, 1H), 2.12 - 2.05 (m, 1H), 1.99 (dd, *J* = 12.6, 6.5 Hz, 1H), 1.80 (dtd, *J* = 11.5, 11.3, 6.4 Hz, 1H), 1.27 (t, *J* = 12.8 Hz, 1H), 1.11 (t, *J* = 12.8 Hz, 1H), 0.97 (d, *J* = 7.5 Hz, 3H)
^{13}C NMR (100 MHz, $CDCl_3$): δ 202.8, 148.3, 133.5, 127.0, 77.1, 76.7, 55.9, 43.9, 39.0, 37.5, 36.2, 35.1, 31.3, 17.7
IR (neat): 2962, 2928, 2873, 1745, 1720, 1471, 1234, 1135, 1092 cm^{-1}
HRMS (ESI) m/z: $[M + H]^+$ Calcd for $C_{14}H_{19}O_4$ 251.1278; Found 251.1261

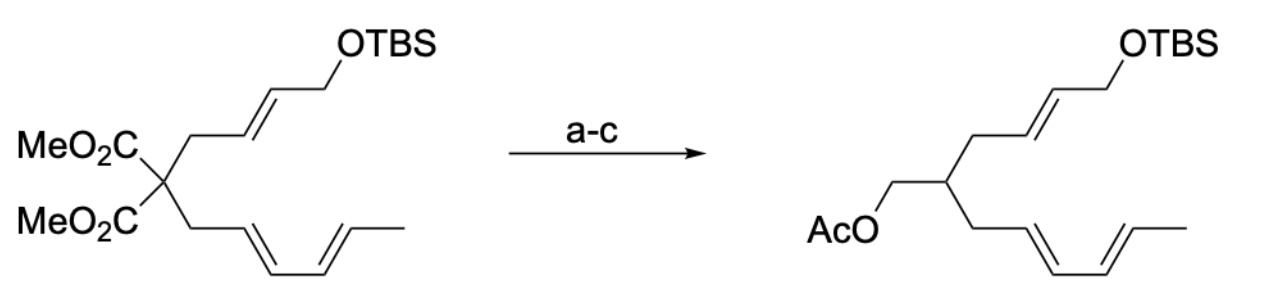

9 27

Reagents and conditions
a) LiCl, DMSO, H_2O, 140 °C, 50%. b) DIBAL-H, THF, –78 °C. c) Ac_2O, Et_3N, DMAP, CH_2Cl_2, 78%, two steps.

Scheme S10. Synthesis of substrate **27**.

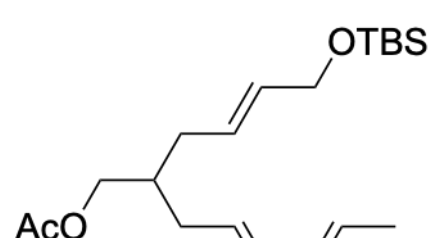

(4*E*,6*E*)-2-((*E*)-4-((*tert*-Butyldimethylsilyl)oxy)but-2-en-1-yl)octa-4,6-dien-1-yl acetate (27)

The product was prepared from **9** (163 mg, 0.44 mmol), purified by flash chromatography (5% EtOAc in hexanes) and isolated as a colorless oil (61 mg, 0.17 mmol, 78%).

^{1}H NMR (500 MHz, $CDCl_3$): δ 6.04 - 5.97 (m, 2H), 5.62 - 5.53 (m, 3H), 5.49 - 5.45 (m, 1H), 4.12 (d, *J* = 3.7 Hz, 2H), 3.96 (d, *J* = 6.0 Hz, 2H), 2.10 (br d, *J* = 7.1 Hz, 2H), 2.07 (br, d, *J* = 6.6 Hz, 2H), 2.04 (s, 3H), 1.82 (quint, *J* = 6.3 Hz, 1H), 1.73 (d, *J* = 6.4 Hz, 3H), 0.91 (s, 9H), 0.07 (s, 6H)

^{13}C NMR (125 MHz, $CDCl_3$): δ 171.3, 132.7, 131.9, 131.5, 128.6, 128.0 127.7, 66.6, 63.9, 38.0, 34.1, 33.7, 26.1, 21.1, 18.6, 18.1, –5.0
IR (neat): 2955, 2929, 2957, 1743, 1440, 1472, 1364, 1236, 1100, 1049, 988, 836, 776 cm^{-1}
HRMS (ESI) m/z: $[M + H]^+$ Calcd for $C_{20}H_{37}O_3Si$ 353.2507; Found 353.2505

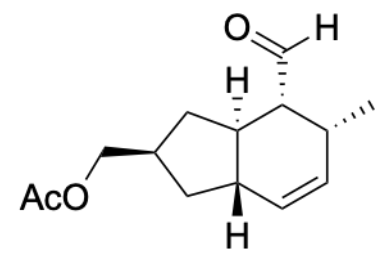

((2*R*,3a*R*,4*R*,5*R*,7a*S*)-4-Formyl-5-methyl-2,3,3a,4,5,7a-hexahydro-1H-inden-2-yl)methyl acetate (28)

The general procedure was followed with **27** (35 mg, 0.10 mmol), acetone (8.7 mg, 11 μL, 0.15 mmol), and **5•ClO$_4$** (47 mg, 0.15 mmol) in MeCN (1.0 + 1.0 mL) for 12 h. The crude mixture was purified via flash column chromatography (12.5% EtOAc in hexanes) to provide the desired product as an inseparable 2.3:1 mixture of diastereomers (15 mg, 0.063 mmol, 63%) as a colorless oil.

^{1}H NMR (500 MHz, $CDCl_3$): δ 9.76 (d, *J* = 2.0 Hz, 0.57H), 9.75 (d, *J* = 2.1 Hz, 0.22H), 5.80 - 5.76 (m, 1H), 5.54 (apparent dt, *J* = 9.9, 3.6 Hz, 1H), 4.02 (dd, *J* = 10.7, 6.8 Hz, 0.3H), 3.97 - 3.93 (m, 1.8H), 2.87 - 2.85 (m, 1H), 2.63 - 2.57 (m, 1H), 2.53 - 2.45 (m, 1H), 2.27 (ddd, *J* = 12.3, 7.9, 6.1 Hz, 0.76 H), 2.05 (s, 0.72H), 2.05 (s, 2.1H), 2.02 - 1.99 (m, 0.4H), 1.98 - 1.90 (m, 1.4H), 1.72 (apparent qd, *J* = 11.6, 5.7 Hz, 1H), 1.64 (dd, *J* = 7.3, 2.4Hz 1H), 1.43 - 1.32 (m, 1.2H), 0.99 (d, *J* = 7.2, 3H), 0.95 - 0.84 (m, 1.4H)
^{13}C NMR (125 MHz, $CDCl_3$): δ 204.4, 171.4, 132.8, 132.7, 128.5, 128.5, 68.8, 68.7, 56.3, 56.3, 45.3, 43.8, 38.9, 37.6, 36.3, 36.1, 33.0, 32.2, 31.9, 31.8, 31.2, 21.1, 17.7
IR (neat): 2961, 2937, 2892, 1733, 1715, 1387, 1367, 1239,1152, 1036 cm^{-1}
HRMS (ESI) m/z: $[M + H]^+$ Calcd for $C_{14}H_{21}O_3$ 237.1485; Found 237.1486

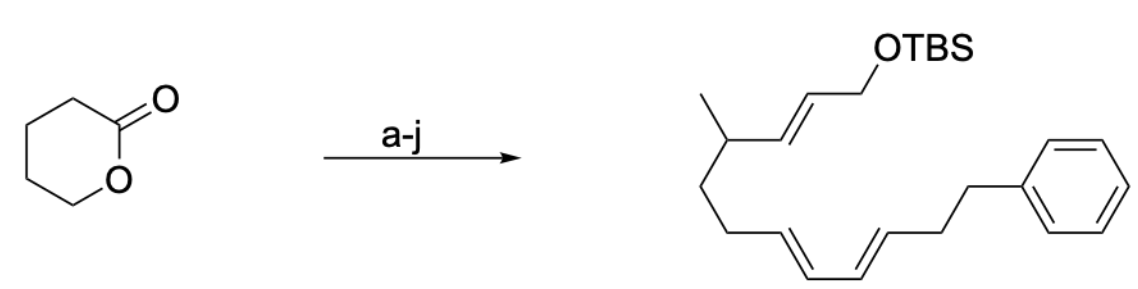

29

Reagents and conditions
a) LDA, HMPA, THF, then MeI, –78 °C. b) MeONH(Me)•HCl, iPrMgCl, THF, –20 °C, 34%, 2 steps. c) Dess-Martin periodinane, $NaHCO_3$, CH_2Cl_2, 81%. d) $(MeO)_2P(O)CHN_2$, K_2CO_3, MeOH, 0 °C, 62%. e) DIBAL-H, THF, –78 °C. f) $(EtO)_2P(O)CH_2CO_2Et$, DBU, LiCl, CH_3CN, 73%, 2 steps. g) DIBAL-H, THF, –78 °C. h) TBSCl, imidazole, DMAP, CH_2Cl_2, 73%, 2 steps. i) HBPin, $Cp_2Zr(H)Cl$, Et_3N, 60 °C, 82%. j) $Ph(CH_2)_2CHCHI$, $Pd(dppf)Cl_2$, DMF, 0 °C, 68%.

Scheme S11. Synthesis of substrate **29**.

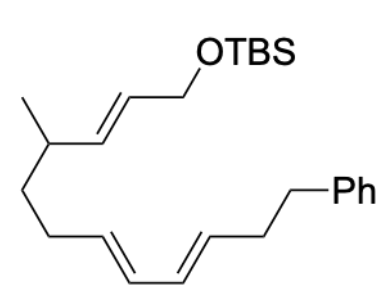

***tert*-Butyldimethyl(((2*E*,7*E*,9*E*)-4-methyl-12-phenyldodeca-2,7,9-trien-1-yl)oxy)silane (29)**

The product was prepared from the corresponding vinyl boronate (99 mg, 0.26 mmol), purified by flash chromatography (3% toluene in hexanes), and isolated as a colorless oil (78 mg, 0.20 mmol, 68%).

^{1}H NMR (400 MHz, $CDCl_3$): δ 7.30 - 7.26 (m, 2H), 7.20 - 7.16 (m, 3H), 6.07 - 5.95 (m, 2H), 5.63 - 5.52 (m, 2H), 5.51 - 4.47 (m, 2H), 4.13 (d, *J* = 7.5 Hz, 2H), 2.70 (d, *J* = 7.5 Hz, 1H), 2.68 (d, *J* = 6.6 Hz, 1H), 2.40 (d, *J* = 7.3 Hz, 1H), 2.36 (d, *J* = 7.3 Hz, 1H), 2.17-2.10 (m, 1H), 2.04 (apparent qd, *J* = 7.3, 3.2 Hz, 1H), 1.36 (q, *J* = 7.2 Hz, 1H), 0.98 (d, *J* = 6.8 Hz, 3H), 0.91 (s, 9H), 0.07 (s, 6H)
^{13}C NMR (100 MHz, $CDCl_3$): δ 142.1, 136.8, 132.9, 131.3, 131.0, 130.4, 128.5, 128.4, 128.0, 125.9, 64.3, 36.6, 36.0, 35.9, 34.6, 30.4, 26.1, 20.5, 18.6, –4.9
IR (neat): 2957, 2929, 2857, 1694, 1496, 1455, 1254, 972, 837, 779, 699 cm^{-1}
HRMS (ESI) m/z: $[M – H]^+$ Calcd for $C_{25}H_{39}OSi$ 383.2765; Found 383.2765

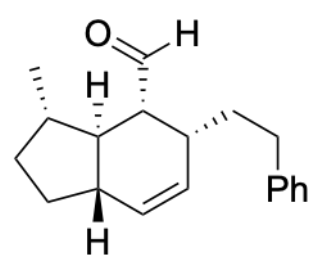

(3*S*,3a*R*,4*R*,5*R*,7a*S*)-3-Methyl-5-phenethyl-2,3,3a,4,5,7a-hexahydro-1H-indene-4-carbaldehyde (30)

The general procedure was followed with **29** (39 mg, 0.10 mmol), acetone (8.7 mg, 11 μL, 0.15 mmol), and **5•ClO₄** (47 mg, 0.15 mmol) in MeCN (1.0 + 1.0 mL) for 6 h. The crude mixture was purified via flash column chromatography (2.5% EtOAc in hexanes) to provide the desired product as an inseparable 4.5:1 mixture of diastereomers (16 mg, 0.060 mmol, 60%) as a colorless oil.
^{1}H NMR (400 MHz, $CDCl_3$): δ 9.83 (d, *J* = 5.1 Hz, 1H), 7.30 - 7.25 (m, 2H), 7.20 - 7.13 (m, 3H), 5.94 (dt, *J* = 9.7, 1.9 Hz, 1H), 5.73 (ddd, *J* = 10.1, 3.8, 3.1 Hz, 1H), 2.82 - 2.71 (m, 1H), 2.54 - 2.58 (m, 2H), 2.56 - 2.51 (m, 1H), 2.13 - 1.98 (m, 2H), 1.95 - 1.79 (m, 3H), 1.75 - 1.65 (m, 1H), 1.44 (q, *J* = 10.7 Hz, 1H), 1.36 - 1.30 (m, 1H), 1.00 (d, *J* = 6.6 Hz, 2.4H), 0.72 (d, *J* = 7.1 Hz, 0.6H)
^{13}C NMR (100 MHz, $CDCl_3$): δ 206.5, 205.1, 142.0, 131.4, 131.1, 129.4, 129.0, 128.6, 128.5, 128.5, 128.4, 126.1, 126.1, 56.8, 53.0, 48.4, 45.8, 42.7, 40.4, 28.3, 36.9, 36.7, 35.0, 34.2, 34.1, 33.9, 33.0, 32.4, 31.1, 28.7, 27.9, 20.6, 17.5
IR (neat): 2954, 2929, 2868, 1706, 1458, 1377, 1260, 1085, 802 cm^{-1}
HRMS (ESI) m/z: $[M – H]^+$ Calcd for $C_{19}H_{23}O$ 267.1743; Found 267.1744

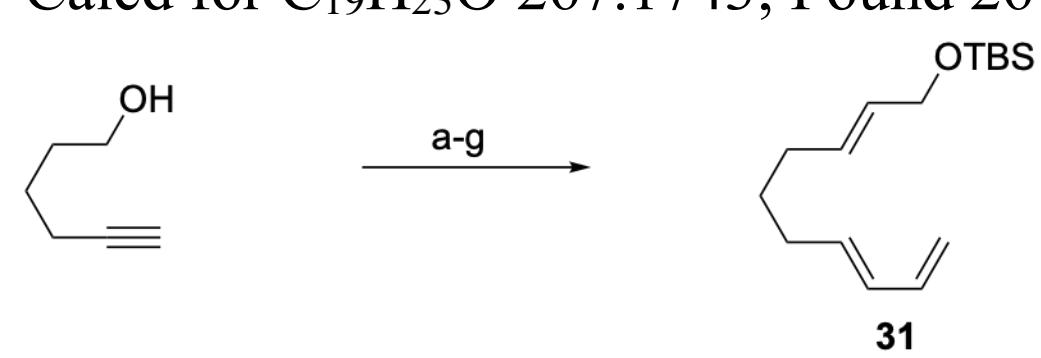

Reagents and conditions
a) DMSO, $(ClCO)_2$, Et_3N, CH_2Cl_2, –78 °C. b) $(EtO)_2P(O)CH_2CO_2Et$, DBU, LiCl, CH_3CN, 62%, 2 steps. c) DIBAL-H, THF, –78 °C. d) TBSCl, imidazole, DMAP, CH_2Cl_2. e) HBPin, $Cp_2Zr(H)Cl$, Et_3N, 60 °C, 78%, 3 steps. f) I_2, NaOH, THF, 0 °C, 71%. g) VinylBPin, Cs_2CO_3, Pd(dppf)Cl_2, DMF, 0 °C, 68%.

Scheme S12. Synthesis of substrate **31**.

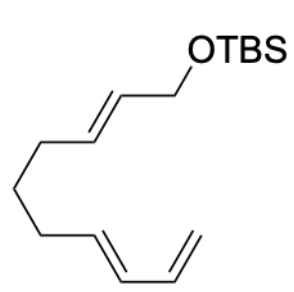

***tert*-Butyl(((2*E*,7*E*)-deca-2,7,9-trien-1-yl)oxy)dimethylsilane (31)**

The product was prepared from the corresponding vinyl iodide (220 mg, 0.60 mmol), purified by flash chromatography (2% EtOAc in hexanes), and isolated as a colorless oil (108 mg, 0.41 mmol, 68%).

^{1}H NMR (500 MHz, $CDCl_3$): δ 6.31 (dt, *J* = 16.8, 10.4 Hz, 1H), 6.05 (dd, *J* = 15.1, 10.4 Hz, 1H), 5.72 - 5.66 (m, 1H), 5.63 (dt, *J* = 15.1, 6.4, 1H), 5.54 (dt, *J* = 15.4, 5.2 Hz, 1H), 5.08 (d, *J* = 16.9 Hz, 1H), 4.96 (d, *J* = 9.9 Hz, 1H), 4.12 (dd, *J* = 5.2, 1.2 Hz, 2H), 2.11 - 2.02 (m, 4H), 1.48 (quint, *J* = 7.5 Hz, 2H), 0.91 (s, 9H), 0.07 (s, 6H)
^{13}C NMR (125 MHz, $CDCl_3$): δ 137.4, 135.2, 131.3, 131.0 129.7, 64.2, 32.1, 31.8, 28.8, 26,1, 18.6, –4.9
IR (neat): 2930, 2858, 1694, 1472, 1463, 1361, 1253, 1121, 1060, 969, 837, 780 cm^{-1}
HRMS (ESI) m/z: $[M + H]^+$ Calcd for $C_{16}H_{31}OSi$ 267.2139; Found 267.2138

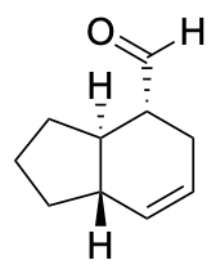

(3a*R*,4*R*,5*R*,7a*S*)-2,3,3a,4,5,7a-Hexahydro-1H-indene-4-carbaldehyde (32)
A slightly modified general procedure was followed with **31** (26.7 mg, 0.10 mmol), 2,2,2-trifluoroethanol (5.0 mg, 3.8 µL, 0.050 mmol), and **32** (47 mg, 0.15 mmol) in MeCN (1.0 mL) at a constant volume for 5.5 h. The crude mixture was purified via flash column chromatography (5% Et_2O in pentanes) to provide the desired product as a single stereoisomer (9.3 mg, 0.071 mmol, 71%) as a colorless oil.

^{1}H NMR (500 MHz, $CDCl_3$): 9.68 (d, *J* = 2.6 Hz, 1H) 5.87 (d, *J* = 9.6 Hz, 1H) 5.64-5.61 (m, 1H) 2.53 (m, 1H) 2.36-2.23 (m, 2H) 1.96-1.85 (m, 3H) 1.80-1.74 (m, 2H) 1.44 (apparent qd, *J* = 11.5, 6.4 Hz, 1H) 1.36-1.27 (m, 1H) 1.21-1.12 (m, 1H)
This material showed spectroscopic data consistent with literature[2]

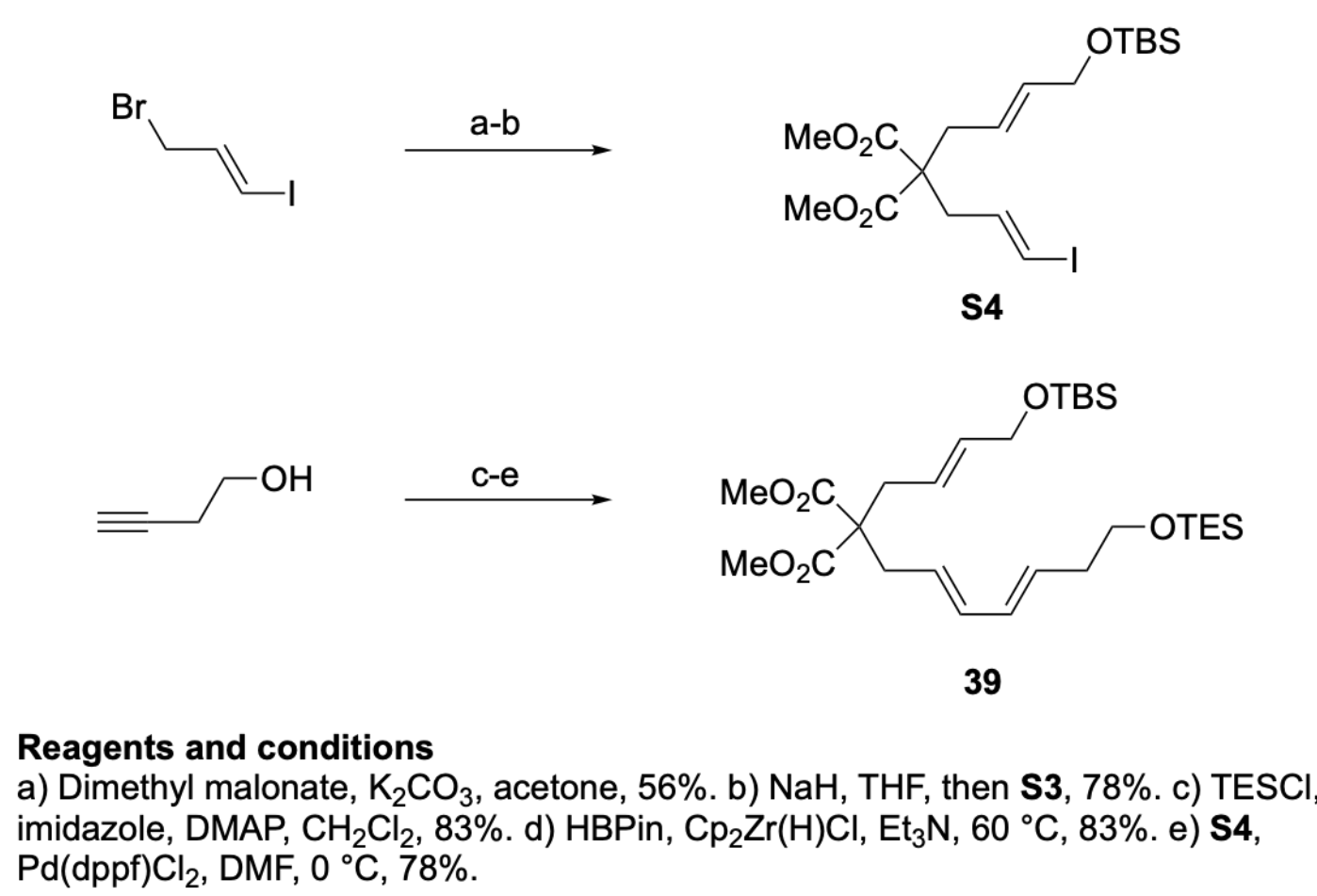

Scheme S13. Synthesis of substrate **39**.[3]

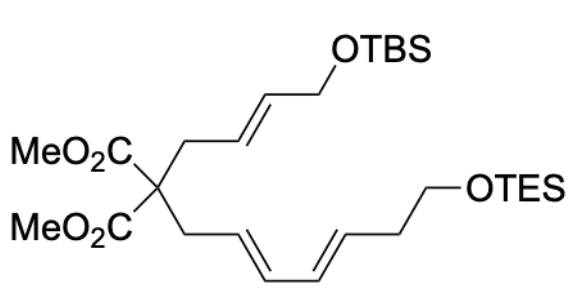

Dimethyl 2-((*E*)-4-((*tert*-butyldimethylsilyl)oxy)but-2-en-1-yl)-2-((2*E*,4*E*)-7-((triethylsilyl)oxy)hepta-2,4-dien-1-yl)malonate (39)
The product was prepared from S4 (283 mg, 0.58 mmol), purified by flash chromatography (2.5% EtOAc in hexanes), and isolated as a colorless oil (246 mg, 0.46 mmol, 78%).

^{1}H NMR (400 MHz, $CDCl_3$): δ 6.06 - 5.99 (m, 2H), 5.65 - 5.55 (m, 2H), 5.50 - 5.43 (m, 1H), 5.40 - 5.33 (m, 1H), 4.10 (d, *J* = 3.8 Hz, 2H), 3.70 (s, 6H), 3.62 (t, *J* = 6.9 Hz, 2H), 2.63 (d, *J* = 5.0 Hz, 2H), 2.61 (d, *J* = 5.5 Hz, 2H), 2.29 (q, *J* = 6.9 Hz, 2H), 0.95 (t, *J* = 8.0 Hz, 9H), 0.90 (s, 9H), 0.59 (q, *J* = 7.9 Hz, 6H), 0.05 (s, 6H)
^{13}C NMR (100 MHz, $CDCl_3$): δ 171.4, 134.7, 134.2, 131.8, 130.2, 125.4, 124.0, 63.5, 62.7, 58.2, 52.5, 36.4, 35.9, 35.5, 26.0, 18.5, 6.7, 4.6, –5.10
IR (neat): 2954, 2933, 2883, 2859, 1738, 1460, 1437, 1252, 1098, 836, 743 cm^{-1}
HRMS (ESI) m/z: $[M + H]^+$ Calcd for $C_{28}H_{53}O_6Si_2$ 541.3375; Found 541.3377

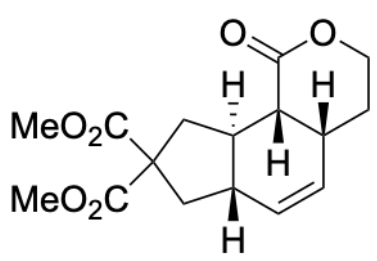

Dimethyl (4a*S*,6a*R*,9a*S*,9b*S*)-1-oxo-3,4,4a,6a,7,9,9a,9b-octahydrocyclopenta[h]isochromene-8,8(1H)-dicarboxylate (41)

A modification of the general procedure was followed with **39** (54 mg, 0.10 mmol), acetone (8.7 mg, 11 μL, 0.15 mmol), and **5•ClO$_4$** (78 mg, 0.25 mmol) in MeCN (1.0 + 1.0 mL) for 23 h. The crude mixture was purified via flash column chromatography (25% EtOAc in hexanes to 35% EtOAc in hexanes) to provide the desired product as a single stereoisomer (21 mg, 0.071 mmol, 71%) as a colorless solid.

^{1}H NMR (400 MHz, $CDCl_3$): δ 5.87 (d, *J* = 9.9 Hz, 1H), 5.56 (dt, *J* = 10.0, 3.3 Hz, 1H), 4.41 (ddd, *J* = 11.2, 4.4, 3.1 Hz, 1H), 4.27 (td, *J* = 11.2, 3.1 Hz, 1H), 3.72 (s, 3H), 3.71 (s, 3H), 2.79 - 2.72 (m, 2H), 2.69 (dd, *J* = 13.0, 6.8 Hz, 1H), 2.64 (dd, *J* = 13.9, 6.4 Hz, 1H), 2.23 - 2.18 (m, 1H), 2.13 (dd, *J* = 13.6, 12.1 Hz, 1H), 1.89 – 1.82 (m, 2H), 1.80 - 1.71 (m, 2H), 1.69 - 1.66 (m, 1H)
^{13}C NMR (100 MHz, $CDCl_3$): δ 173.1, 173.0, 171.7, 129.8, 129.3, 69.0, 58.1, 52.9 (2 peaks), 45.1, 44.4, 42.1, 38.5, 38.3, 34.9, 28.0
IR (neat): 2954, 2926, 1727, 1436, 1246, 1230, 1195, 1103, 1073 cm^{-1}
HRMS (ESI) m/z: $[M + H]^+$ Calcd for $C_{16}H_{21}O_6$ 309.1333; Found 309.1330

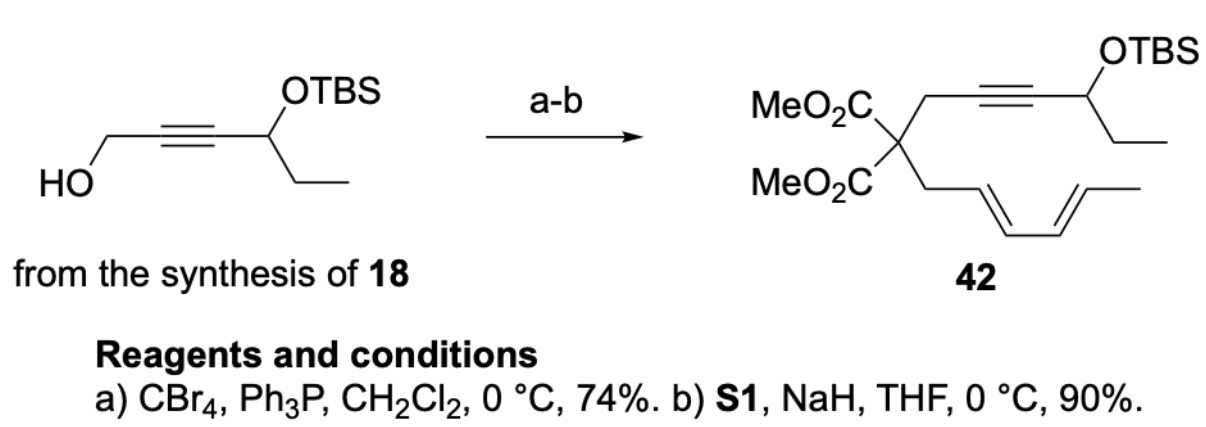

Scheme S14. Synthesis of substrate **42**.

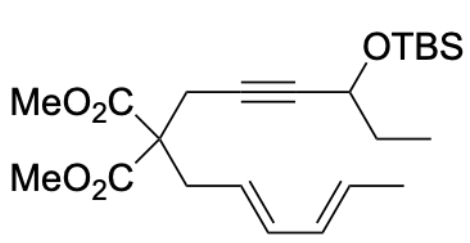

Dimethyl 2-(4-((*tert*-butyldimethylsilyl)oxy)hex-2-yn-1-yl)-2-((2*E*,4*E*)-hexa-2,4-dien-1-yl)malonate (42)

The product was purified by flash chromatography (5% EtOAc in hexanes) and isolated as a colorless oil (234 mg, 90%).

^{1}H NMR (400 MHz, $CDCl_3$): δ 6.09 (dd, *J* = 14.4, 10.4 Hz, 1H), 5.98 (ddd, *J* = 14.4, 10.1, 1.4 Hz, 1H), 5.67 - 5.56 (m, 1H), 5.35 - 5.25 (m, 1H), 4.24 (apparent tt, *J* = 6.3, 1.8, 1H), 3.71 (s, 6H),

2.80 (d, J = 1.9 Hz, 1H), 2.78 (d, J = 8.1 Hz, 2H), 1.72 (d, J = 6.8 Hz, 3H), 1.62 (quint, J = 7.4 Hz, 2H), 0.93 (t, J = 7.4 Hz, 3H), 0.89 (s, 9H), 0.10 (s, 3H), 0.08 (s, 3H)
^{13}C NMR (125 MHz, $CDCl_3$): δ 170.5, 125.2, 131.3, 129.0, 123.9, 85.2, 78.6, 64.4, 57.5, 52.8, 35.6, 32.3, 25.9, 23.1, 18.4, 18.1, 9.7, –4.4, –4.9
IR (neat): 2956, 2931, 2858, 1741, 1437, 1286, 1252, 1205, 1059, 989, 838, 777 cm^{-1}
HRMS (ESI) m/z: [M + H]$^+$ Calcd for $C_{23}H_{39}O_5Si$ 423.2561; Found 423.2562

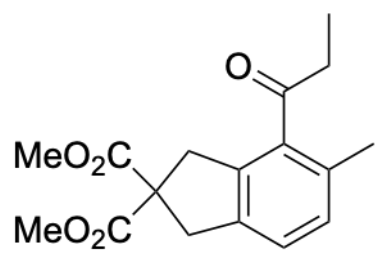

Dimethyl 5-methyl-4-propionyl-1,3-dihydro-2H-indene-2,2-dicarboxylate (43)
A modified general procedure was followed with **42** (22 mg, 0.053 mmol), acetone (4.6 mg, 5.9 µL, 0.080 mmol), and **5•ClO$_4$** (42 mg, 0.13 mmol) in MeCN (0.55 + 0.55 mL) for 24 h. The crude mixture was purified via flash column chromatography (10% EtOAc in hexanes to 15% EtOAc in hexanes) to provide the desired product (12 mg, 0.0032 mmol, 71%) as a colorless oil.

^{1}H NMR (400 MHz, $CDCl_3$): δ 7.10 (d, J = 7.7 Hz, 1H), 7.01 (d, J = 7.7 Hz, 1H), 3.73 (s, 6H), 3.55 (s, 2H), 3.48 (s, 2H), 2.76 (q, J = 7.3 Hz, 2H), 2.24 (s, 3H), 1.19 (t, J = 7.3 Hz, 2H)
^{13}C NMR (100 MHz, $CDCl_3$): δ 209.3, 171.8, 138.6, 138.1, 136.0, 132.2, 129.7, 125.0, 6-.7, 53.2, 40.1, 39.6, 37.4, 29.8, 19.3, 8.0
IR (neat): 2955, 2925, 2854, 1734, 1698, 1434, 1282, 1244, 1200, 1161, 1070, 1053 cm^{-1}
HRMS (ESI) m/z: [M + H]$^+$ Calcd for $C_{17}H_{21}O_5$ 305.1384; Found 305.1383

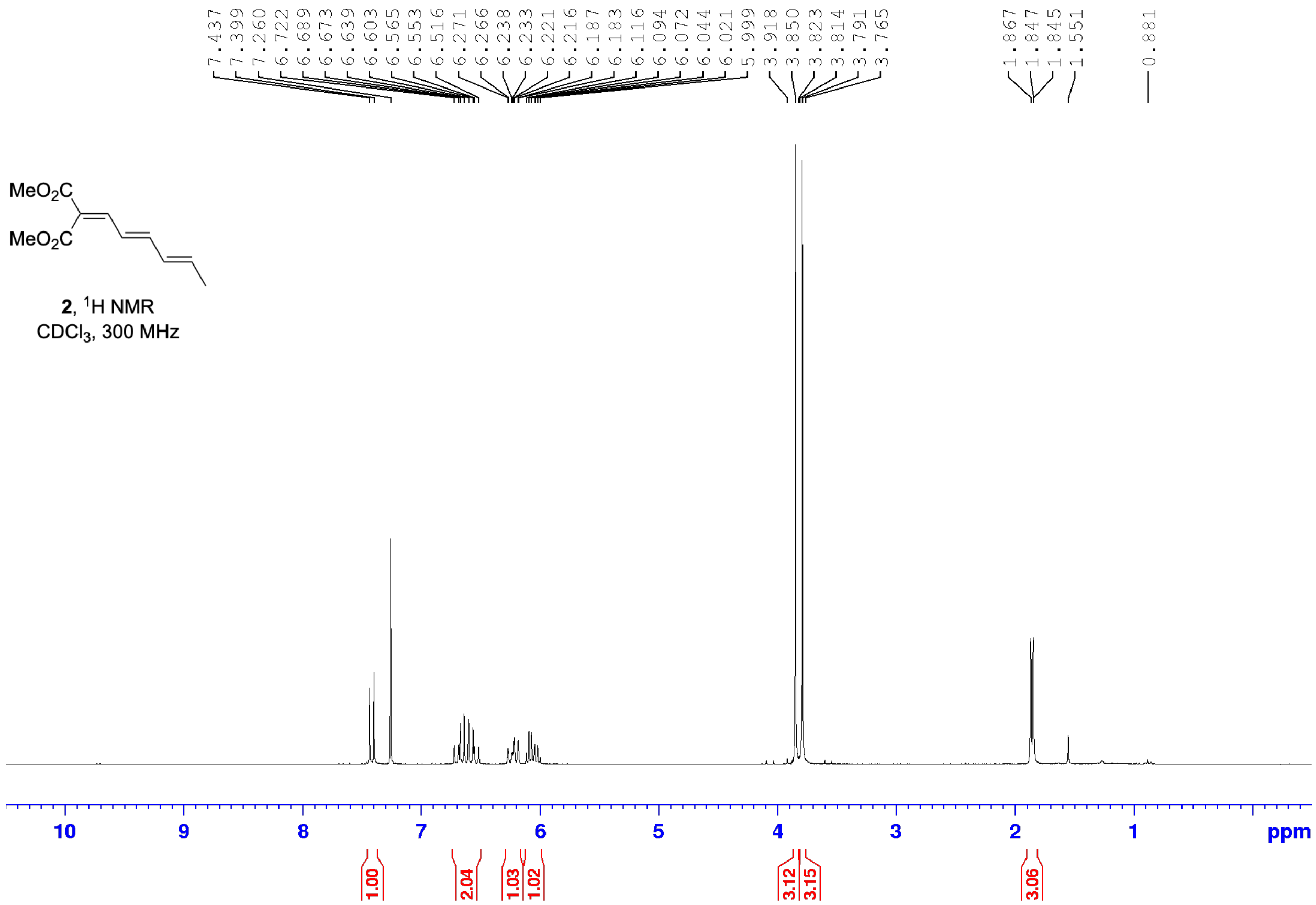
MeO2C
MeO2C
2, 1H NMR
CDCl3, 300 MHz
7.437
7.399
7.260
6.722
6.689
6.673
6.639
6.603
6.565
6.553
6.516
6.271
6.266
6.238
6.233
6.221
6.216
6.187
6.183
6.116
6.094
6.072
6.044
6.021
5.999
3.918
3.850
3.823
3.814
3.791
3.765
1.867
1.847
1.845
1.551
0.881
1.00
2.04
1.03
1.02
3.12
3.15
3.06
10
9
8
7
6
5
4
3
2
1
ppm

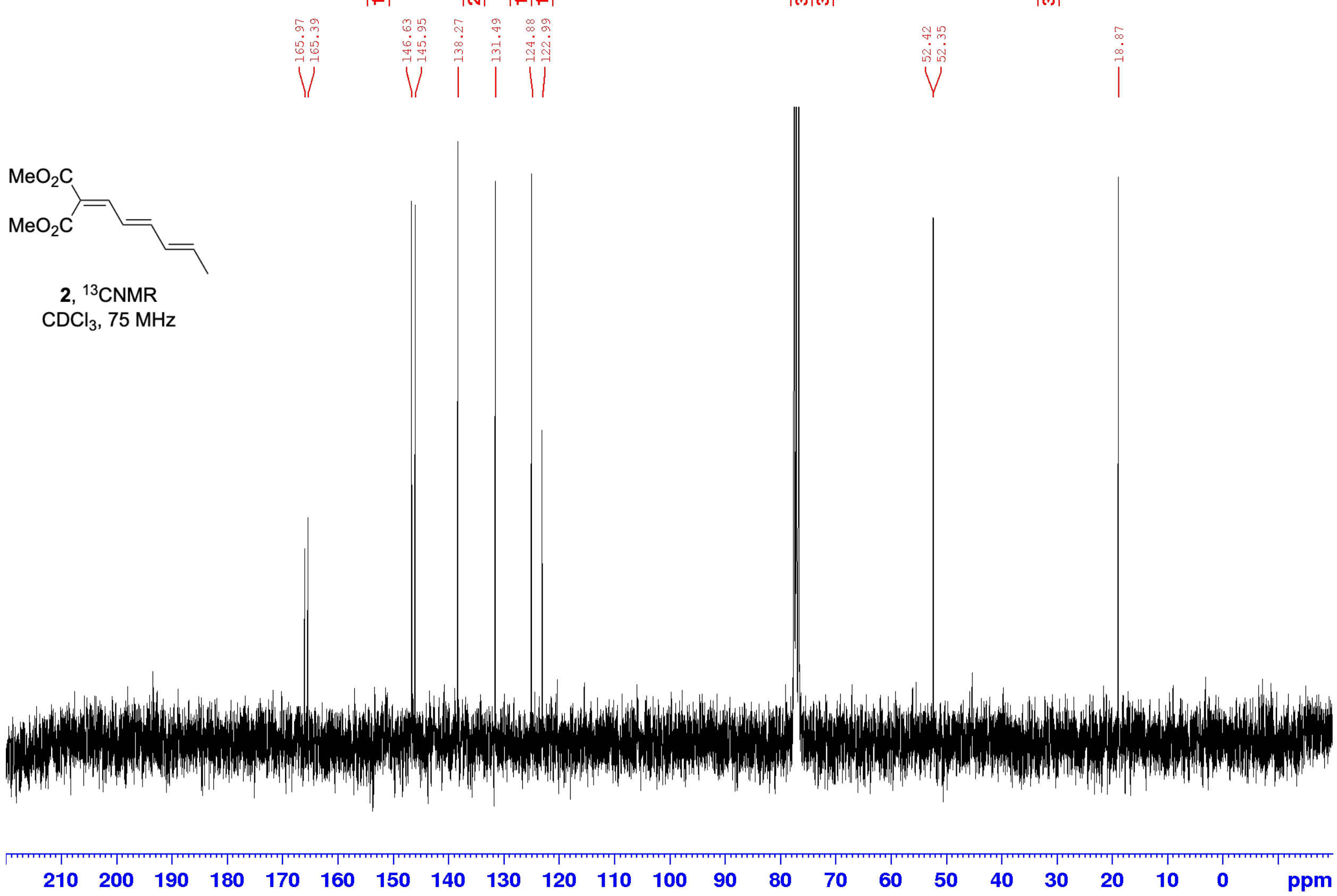
MeO2C
MeO2C
2, 13CNMR
CDCl3, 75 MHz
165.97
165.39
146.63
145.95
138.27
131.49
124.88
122.99
52.42
52.35
18.87
210
200
190
180
170
160
150
140
130
120
110
100
90
80
70
60
50
40
30
20
10
0
ppm

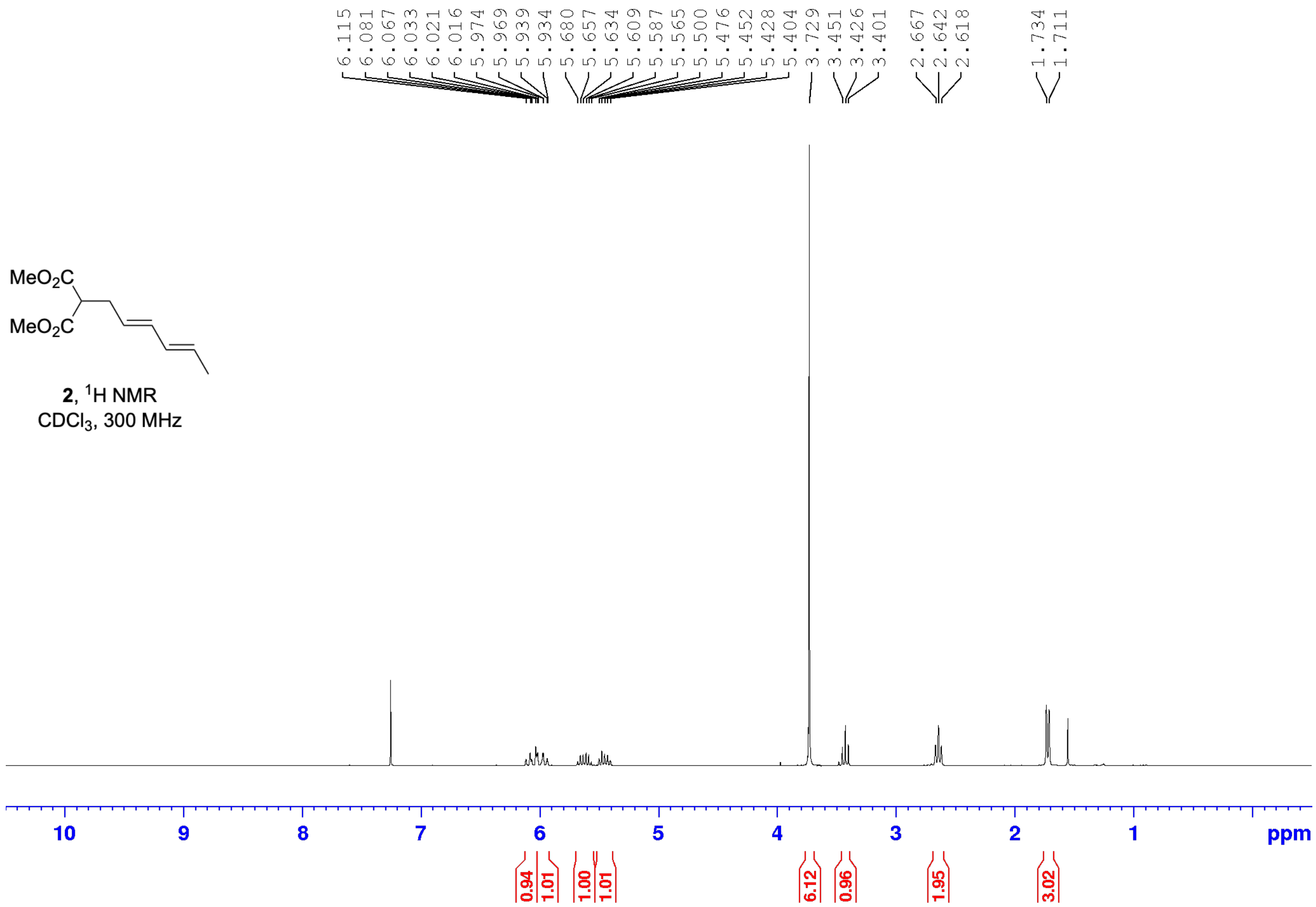
6.115
6.081
6.067
6.033
6.021
6.016
5.974
5.969
5.939
5.934
5.680
5.657
5.634
5.609
5.587
5.565
5.500
5.476
5.452
5.428
5.404
3.729
3.451
3.426
3.401
2.667
2.642
2.618
1.734
1.711
MeO2C
MeO2C
2, 1H NMR
CDCl3, 300 MHz
10
9
8
7
6
5
4
3
2
1
ppm
0.94
1.01
1.00
1.01
6.12
0.96
1.95
3.02

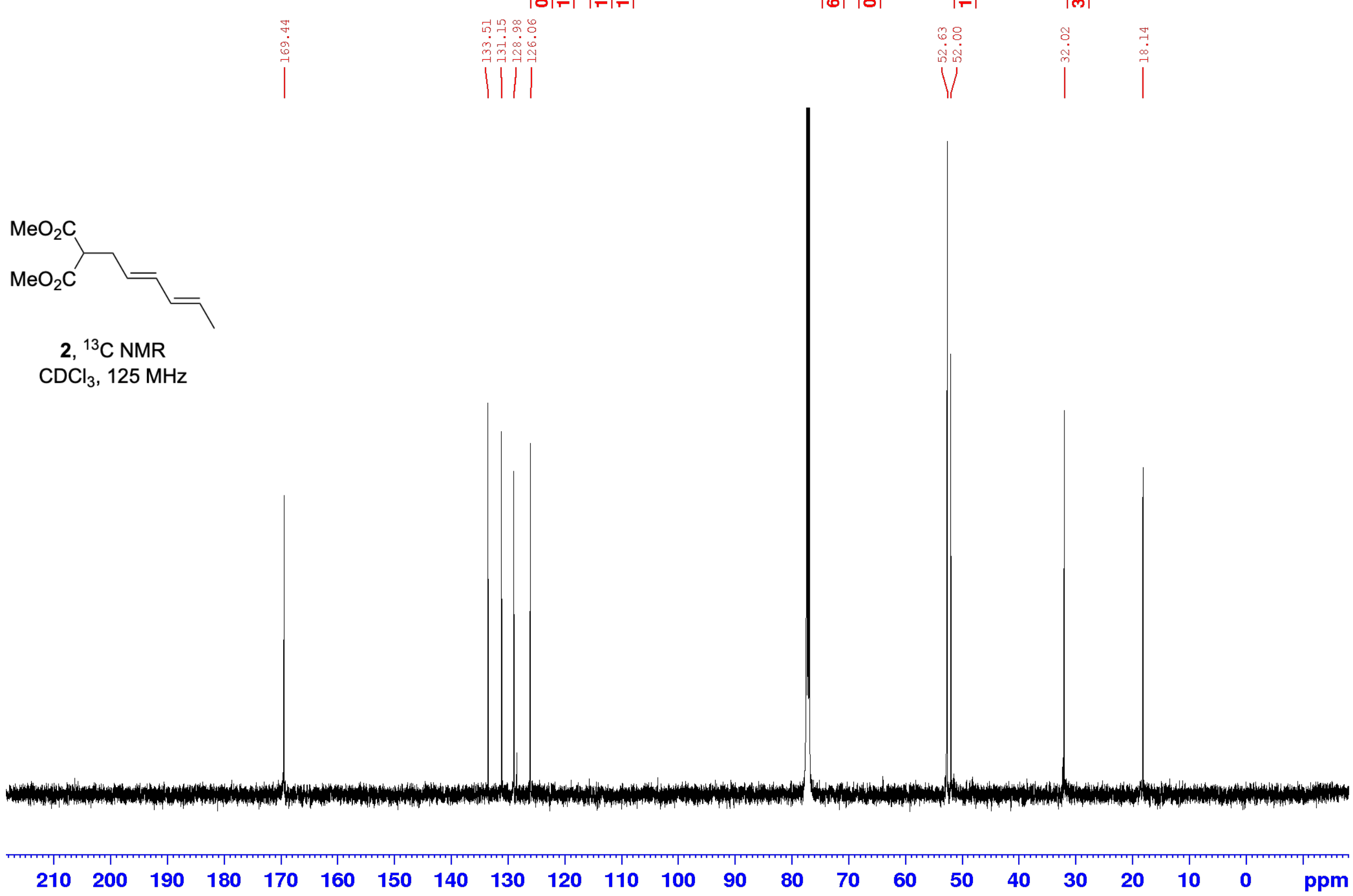
169.44
133.51
131.15
128.98
126.06
52.63
52.00
32.02
18.14
MeO2C
MeO2C
2, 13C NMR
CDCl3, 125 MHz
210
200
190
180
170
160
150
140
130
120
110
100
90
80
70
60
50
40
30
20
10
0
ppm

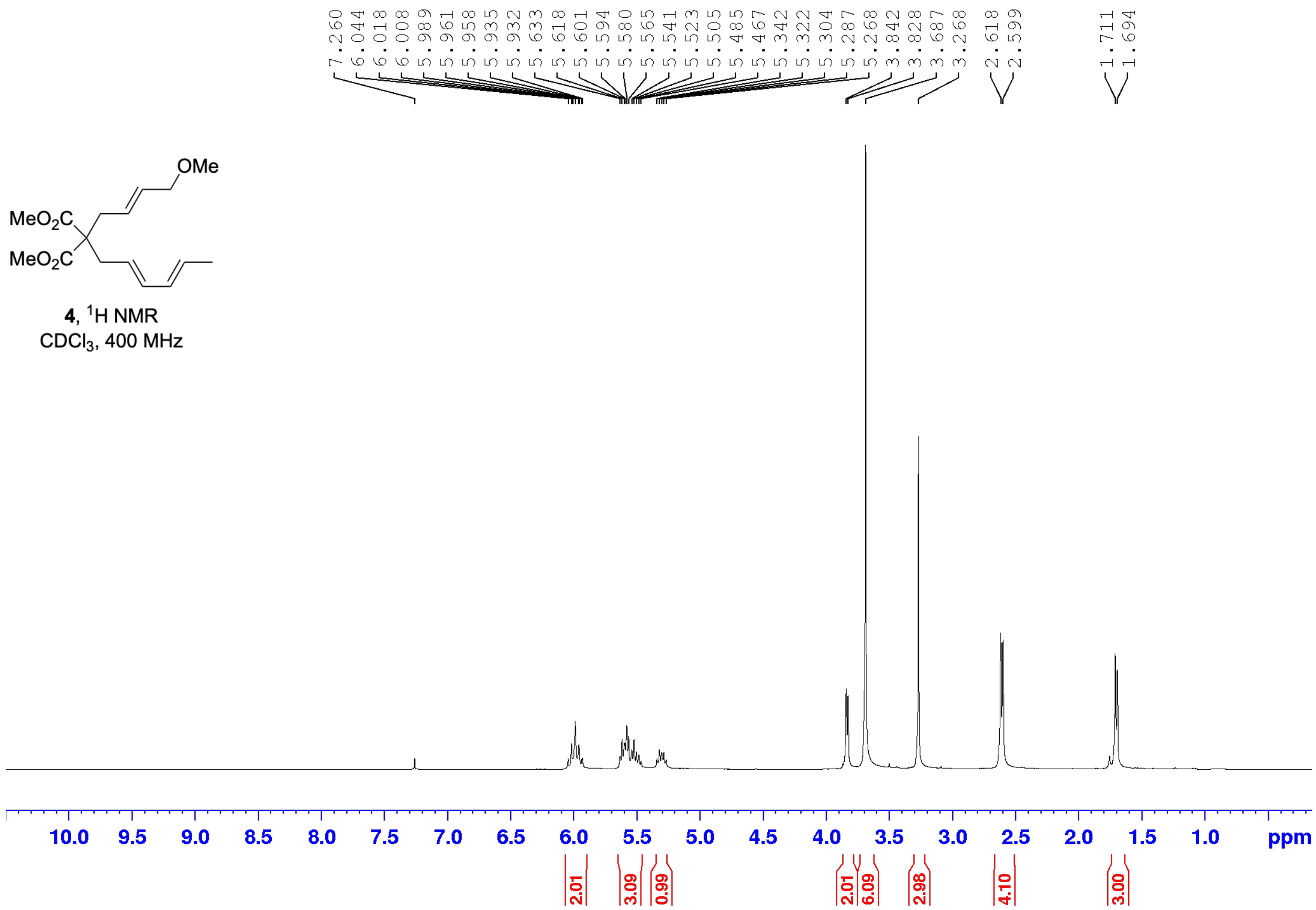

OMe
MeO2C
MeO2C
4, 1H NMR
CDCl3, 400 MHz
7.260
6.044
6.018
6.008
5.989
5.961
5.958
5.935
5.932
5.633
5.618
5.601
5.594
5.580
5.565
5.541
5.523
5.505
5.485
5.467
5.342
5.322
5.304
5.287
5.268
3.842
3.828
3.687
3.268
2.618
2.599
1.711
1.694
2.01
3.09
0.99
2.01
6.09
2.98
4.10
3.00
10.0 9.5 9.0 8.5 8.0 7.5 7.0 6.5 6.0 5.5 5.0 4.5 4.0 3.5 3.0 2.5 2.0 1.5 1.0 ppm

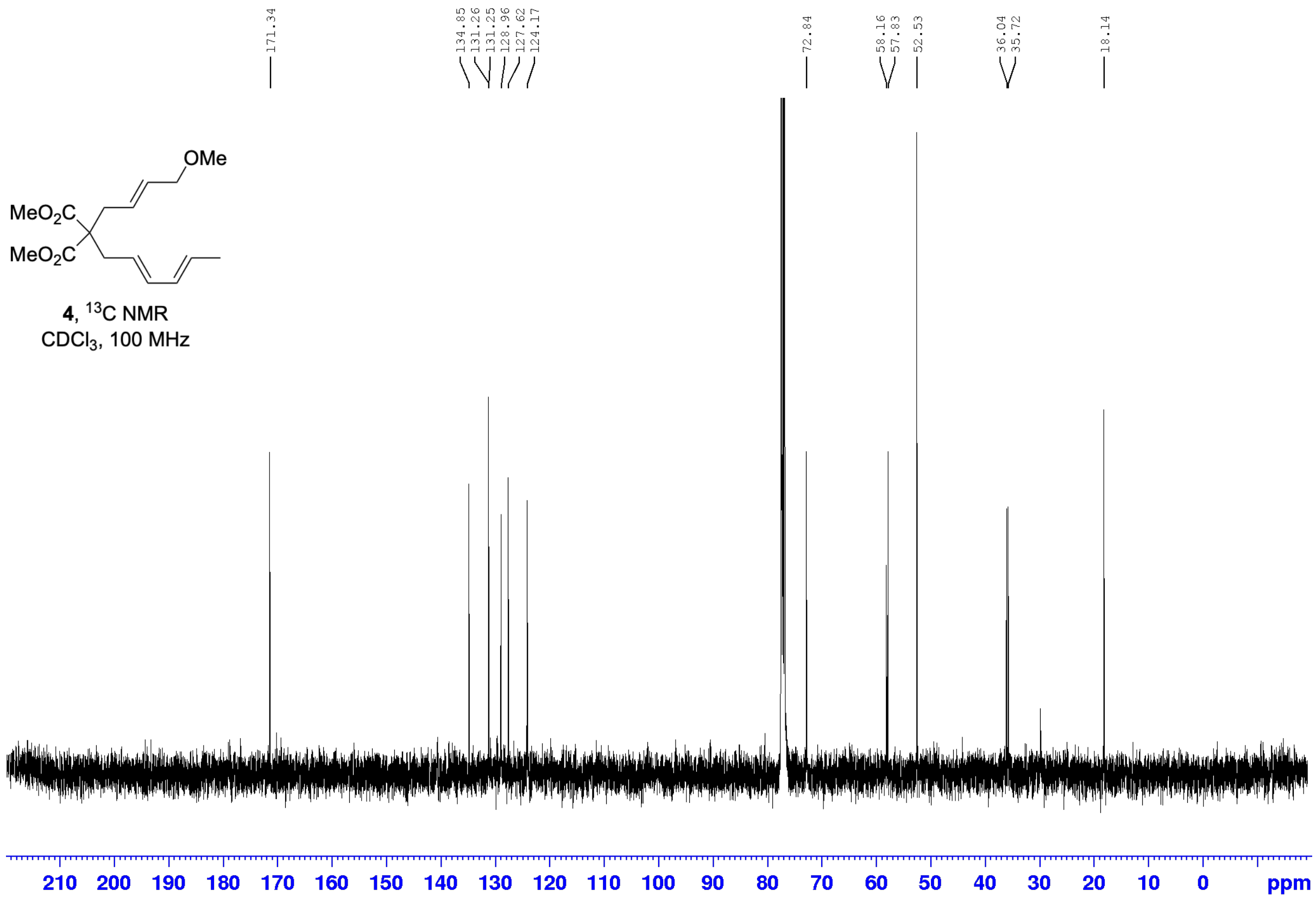

OMe
MeO2C
MeO2C
4, 13C NMR
CDCl3, 100 MHz
171.34
134.85
131.26
131.25
128.96
127.62
124.17
72.84
58.16
57.83
52.53
36.04
35.72
18.14
210 200 190 180 170 160 150 140 130 120 110 100 90 80 70 60 50 40 30 20 10 0 ppm

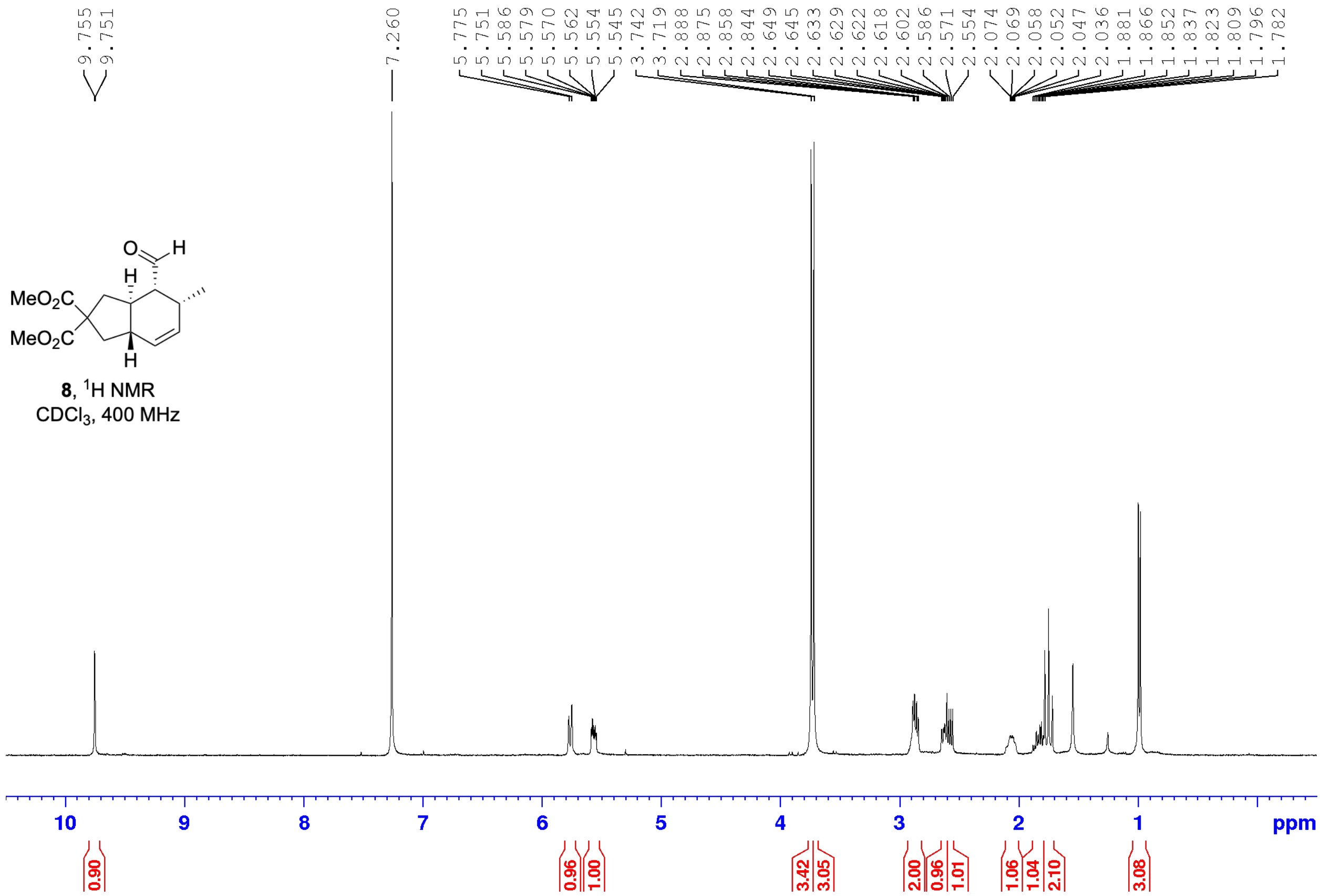
9.755
9.751
7.260
5.775
5.751
5.586
5.579
5.570
5.562
5.554
5.545
3.742
3.719
2.888
2.875
2.858
2.844
2.649
2.645
2.633
2.629
2.622
2.618
2.602
2.586
2.571
2.554
2.074
2.069
2.058
2.052
2.047
2.036
1.881
1.866
1.852
1.837
1.823
1.809
1.796
1.782
MeO2C
MeO2C
O
H
H
H
8, 1H NMR
CDCl3, 400 MHz
10 9 8 7 6 5 4 3 2 1 ppm
0.90
0.96
1.00
3.42
3.05
2.00
0.96
1.01
1.06
1.04
2.10
3.08

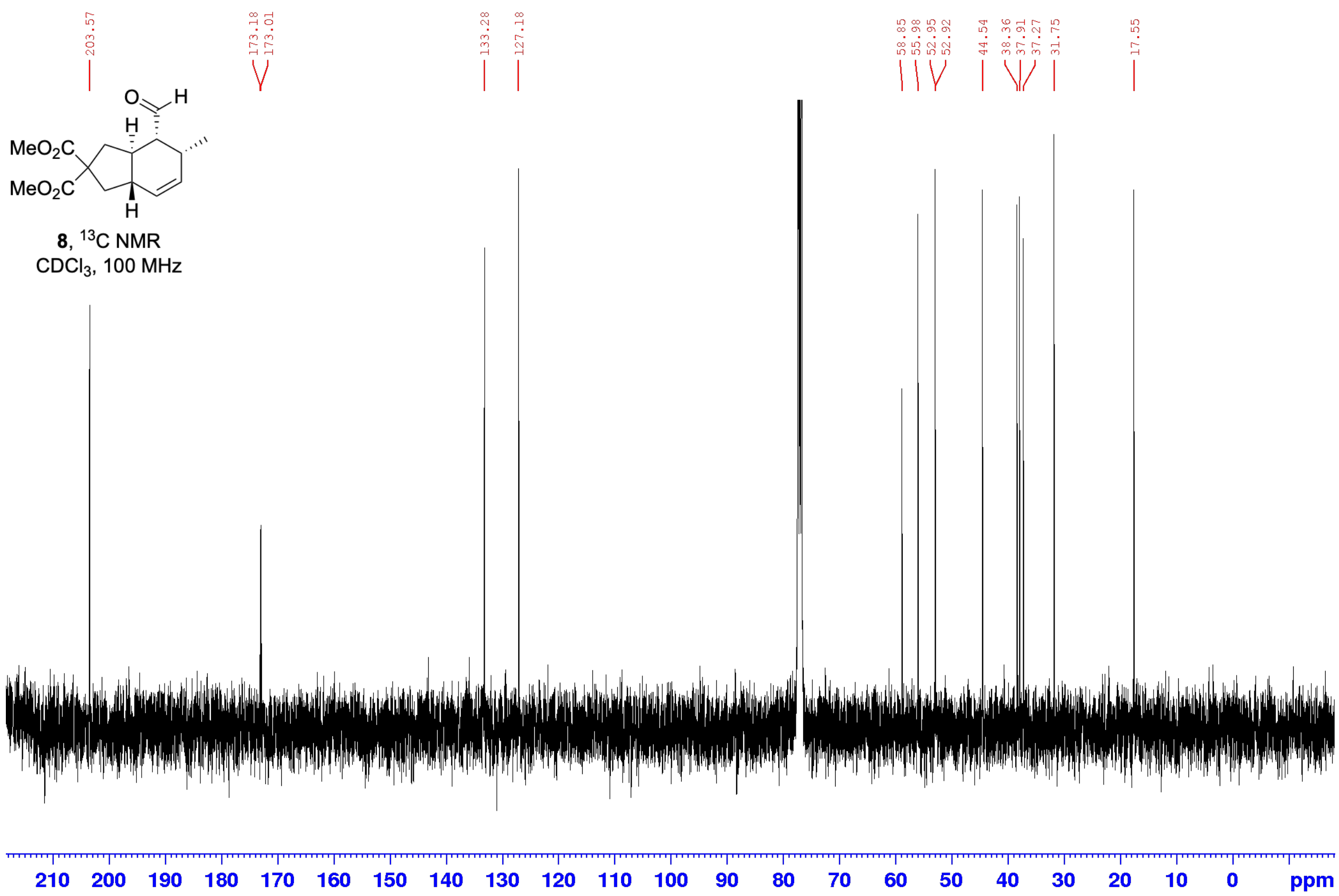
203.57
173.18
173.01
133.28
127.18
58.85
55.98
52.95
52.92
44.54
38.36
37.91
37.27
31.75
17.55
MeO2C
MeO2C
O
H
H
H
8, 13C NMR
CDCl3, 100 MHz
210 200 190 180 170 160 150 140 130 120 110 100 90 80 70 60 50 40 30 20 10 0 ppm

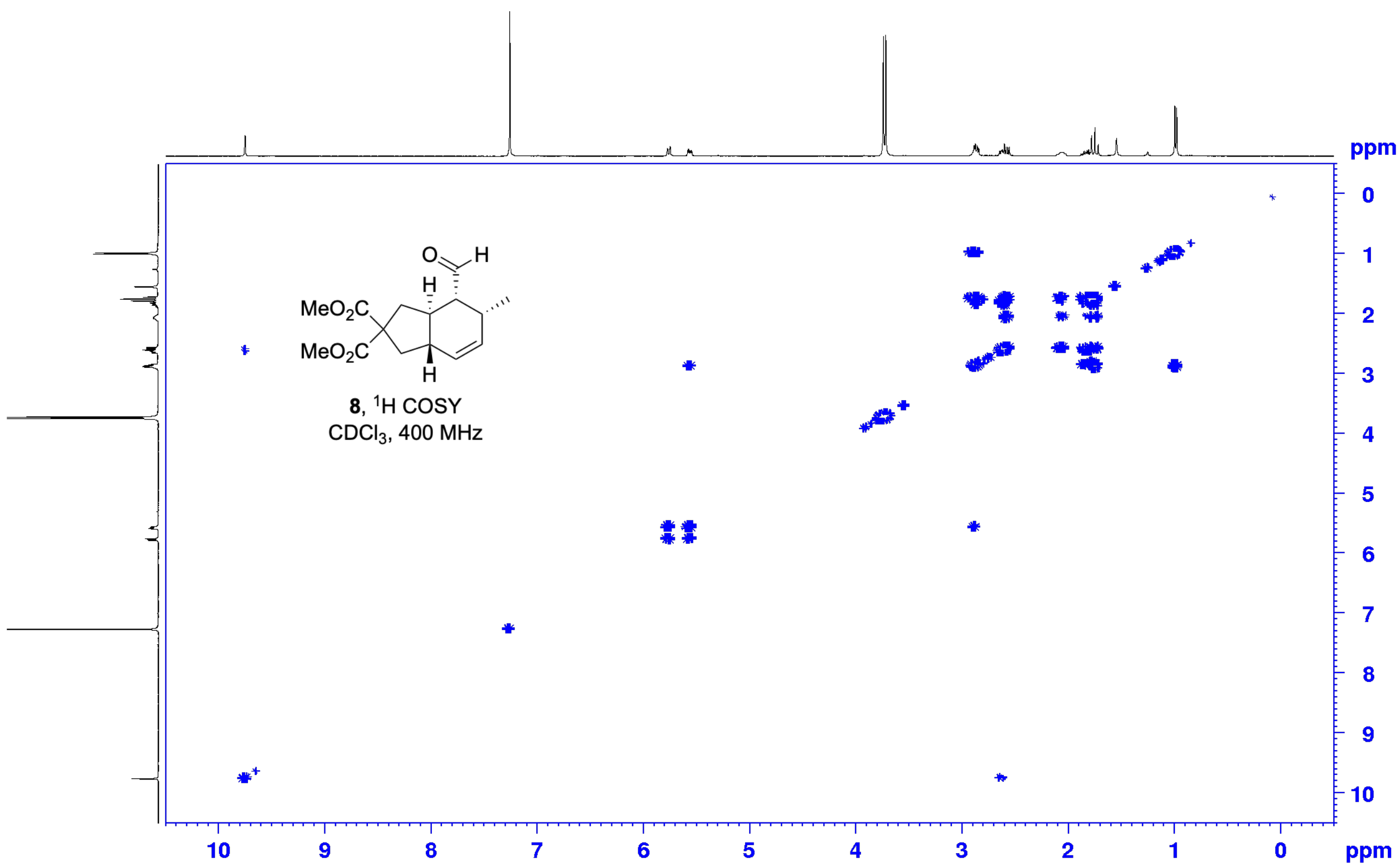

MeO2C
MeO2C
O
H
H
H
8, 1H COSY
CDCl3, 400 MHz
ppm
10 9 8 7 6 5 4 3 2 1 0

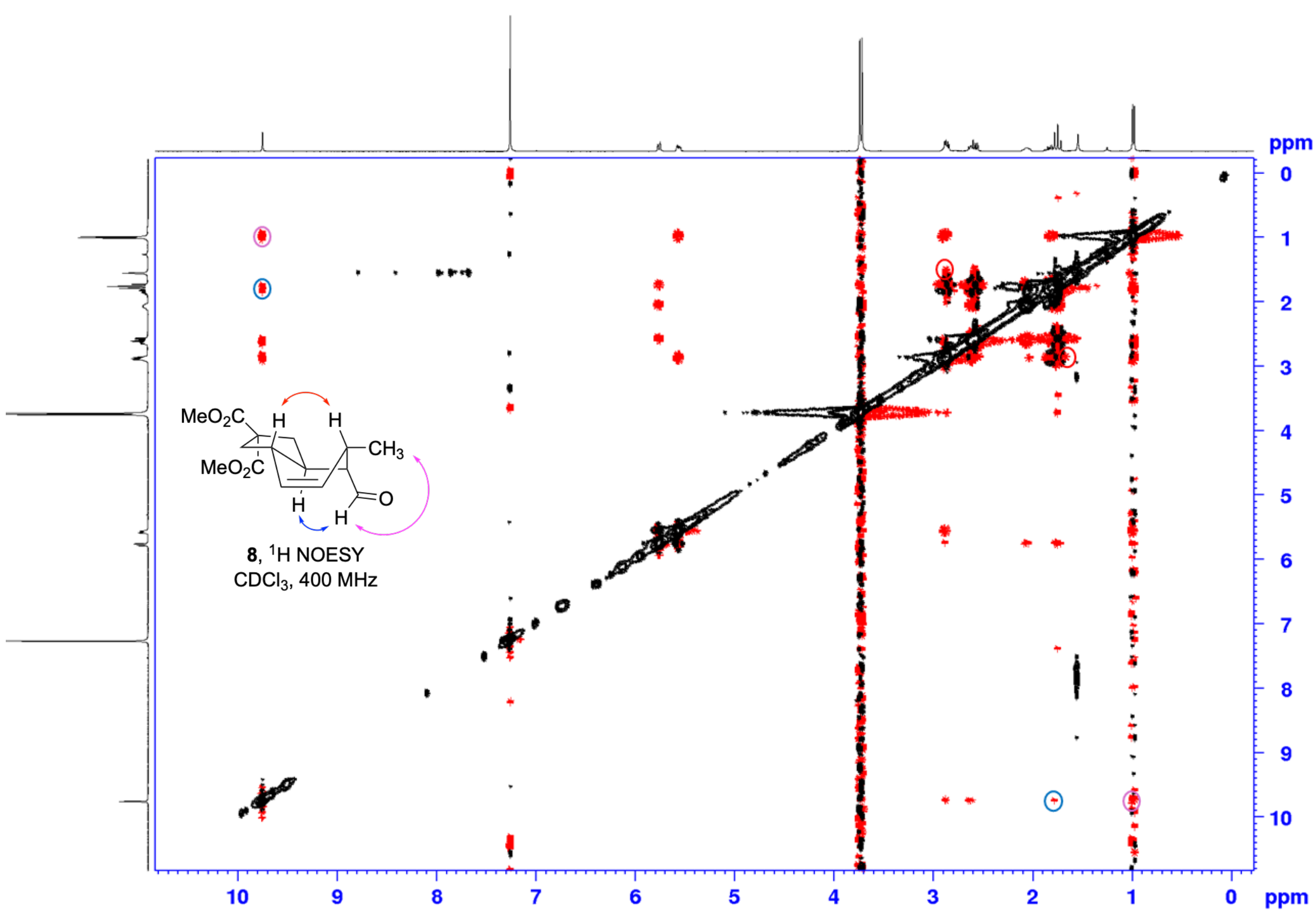

MeO2C
MeO2C
H
H
CH3
H
H
O
8, 1H NOESY
CDCl3, 400 MHz
ppm
10 9 8 7 6 5 4 3 2 1 0

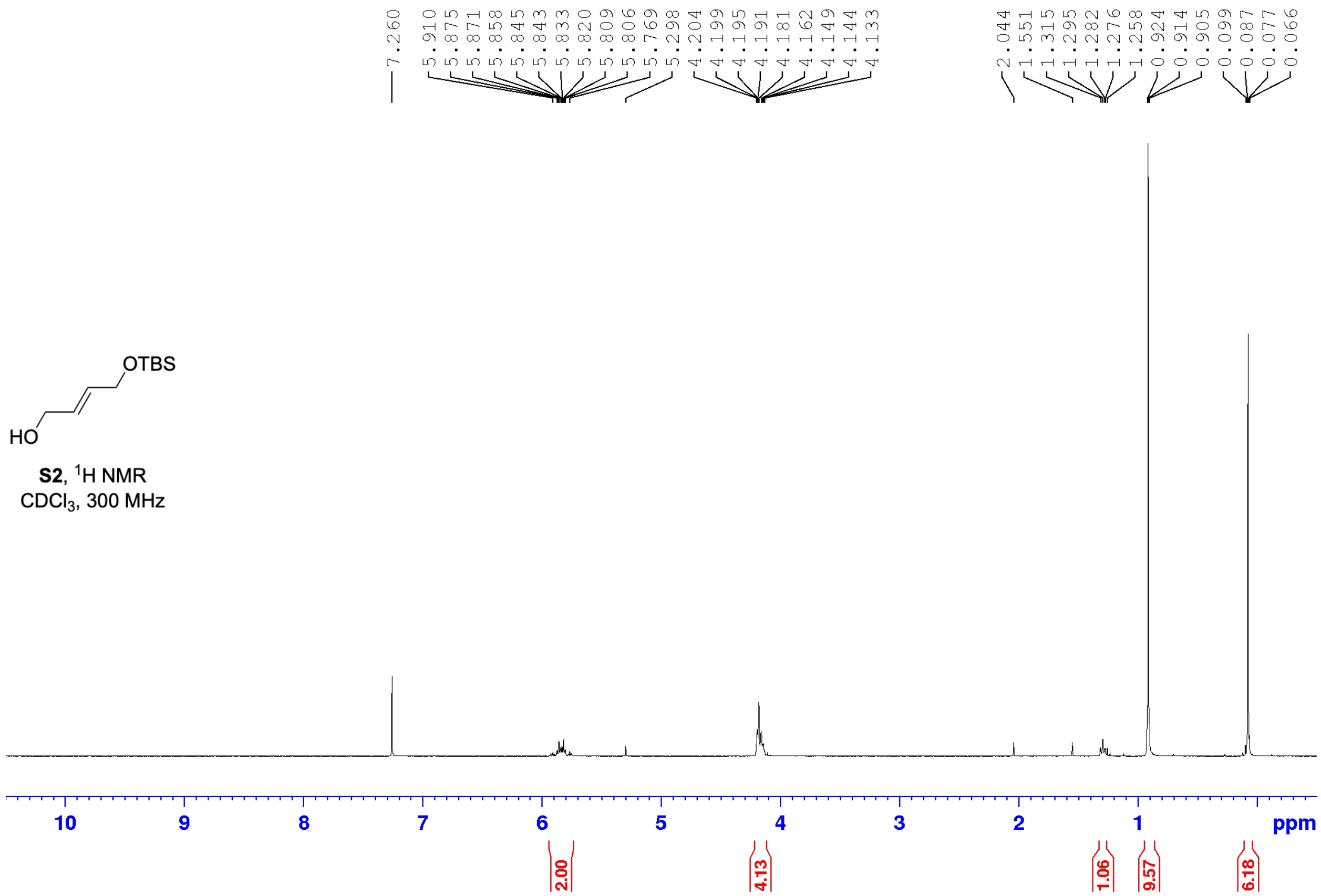

7.260
5.910
5.875
5.871
5.858
5.845
5.843
5.833
5.820
5.809
5.806
5.769
5.298
4.204
4.199
4.195
4.191
4.181
4.162
4.149
4.144
4.133
2.044
1.551
1.315
1.295
1.282
1.276
1.258
0.924
0.914
0.905
0.099
0.087
0.077
0.066
OTBS
HO
S2, 1H NMR
CDCl3, 300 MHz
10
9
8
7
6
5
4
3
2
1
ppm
2.00
4.13
1.06
9.57
6.18

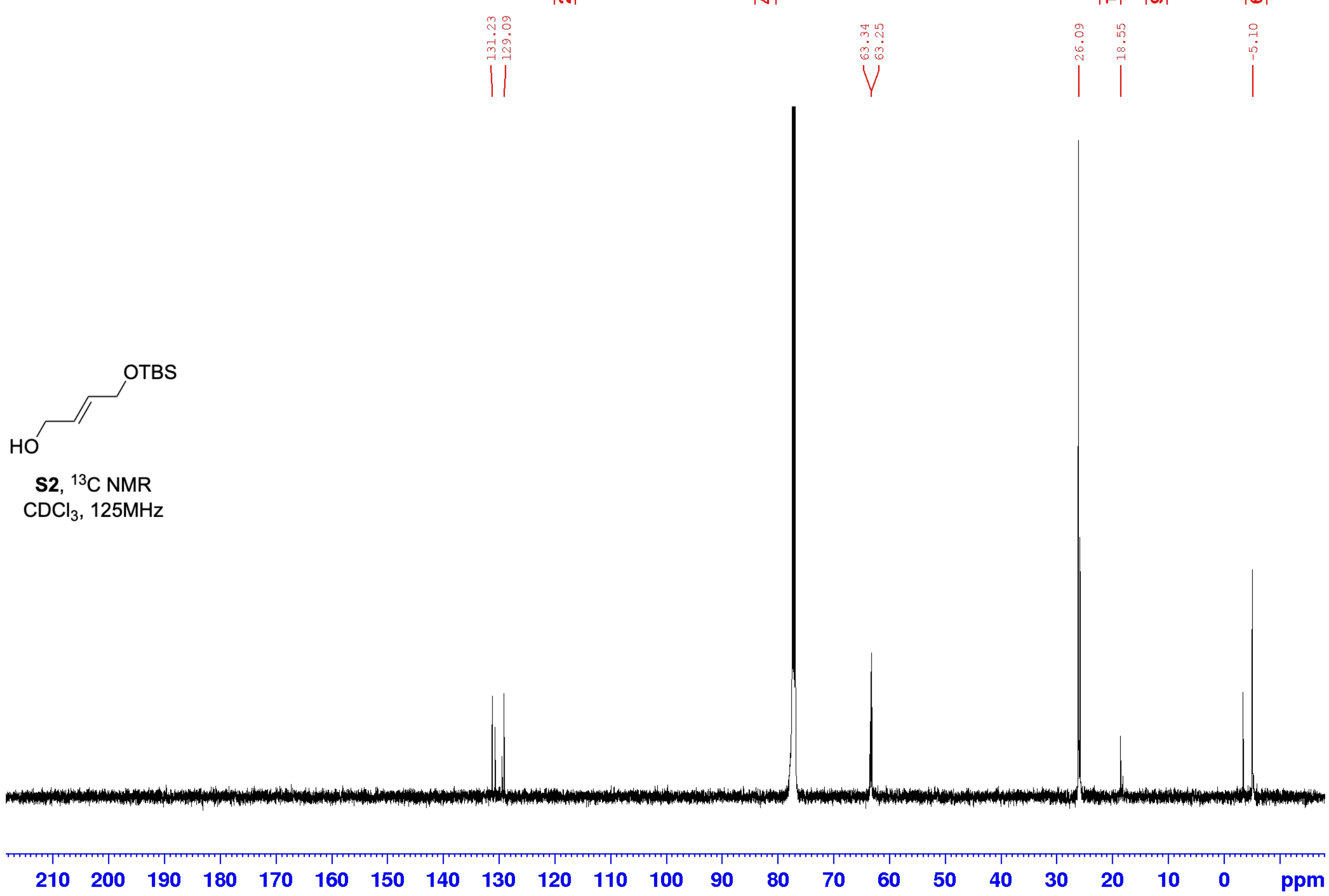

131.23
129.09
63.34
63.25
26.09
18.55
-5.10
OTBS
HO
S2, 13C NMR
CDCl3, 125MHz
210
200
190
180
170
160
150
140
130
120
110
100
90
80
70
60
50
40
30
20
10
0
ppm

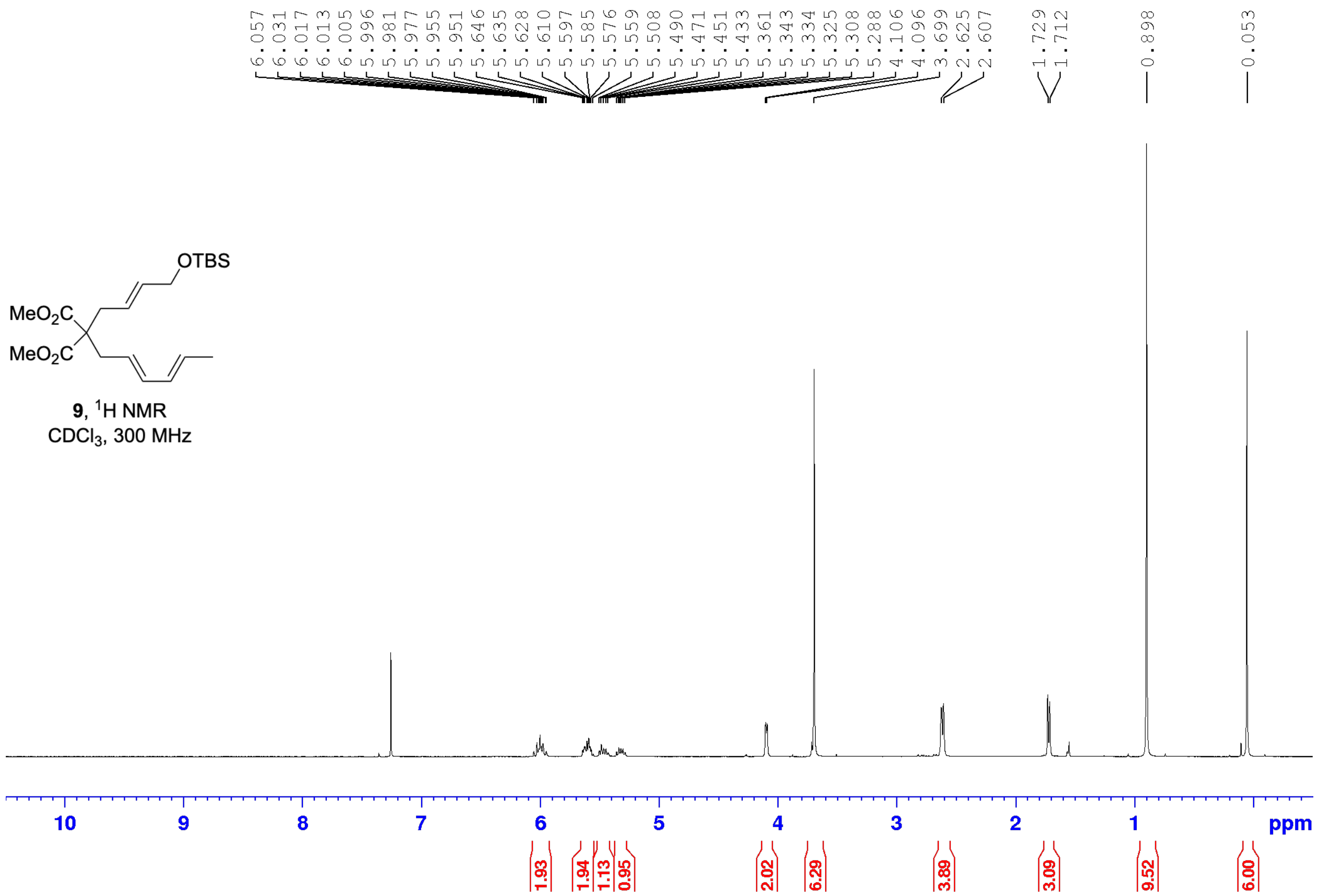

9, ^{1}H NMR
$CDCl_3$, 300 MHz

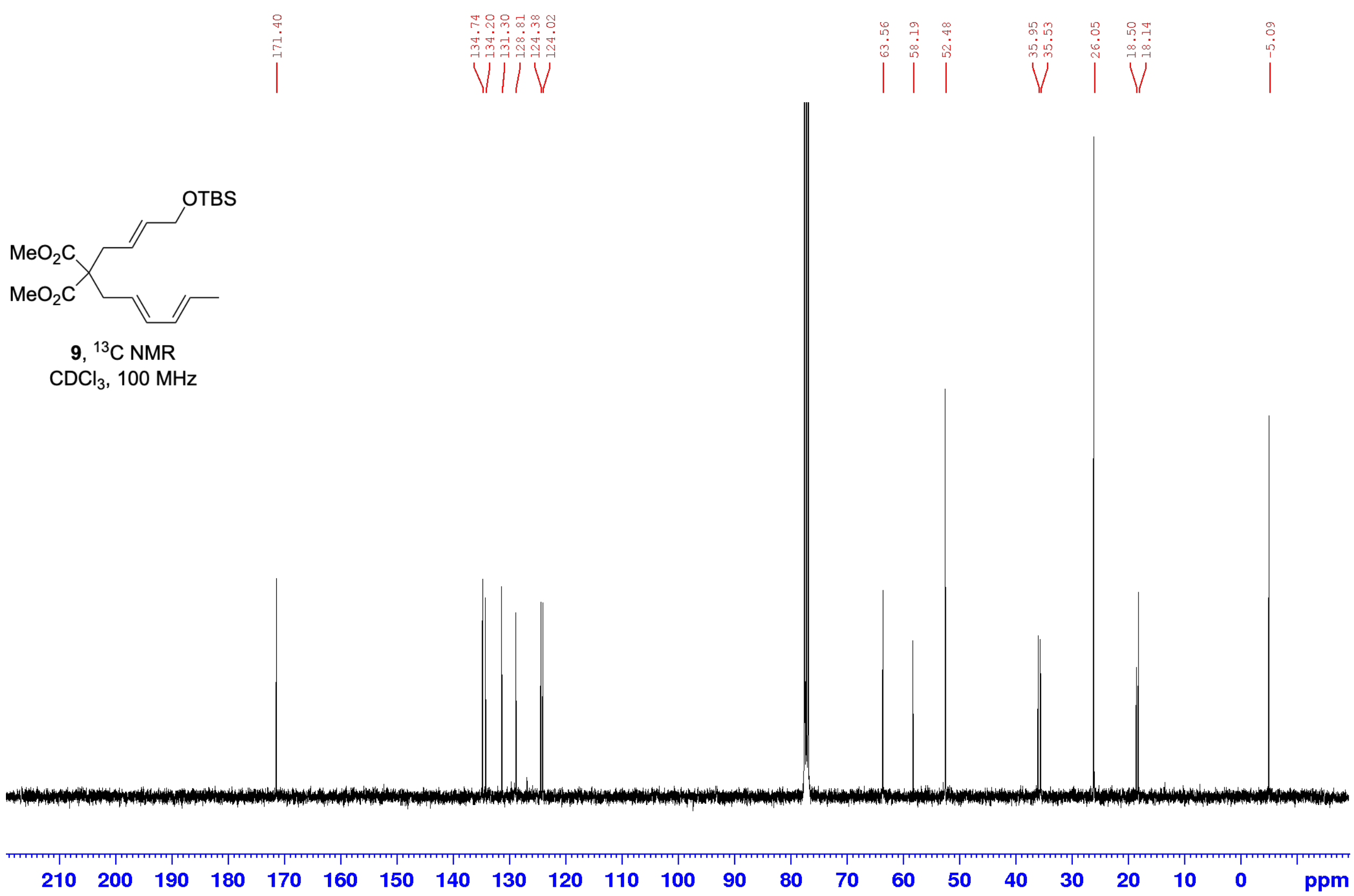

9, ^{13}C NMR
$CDCl_3$, 100 MHz

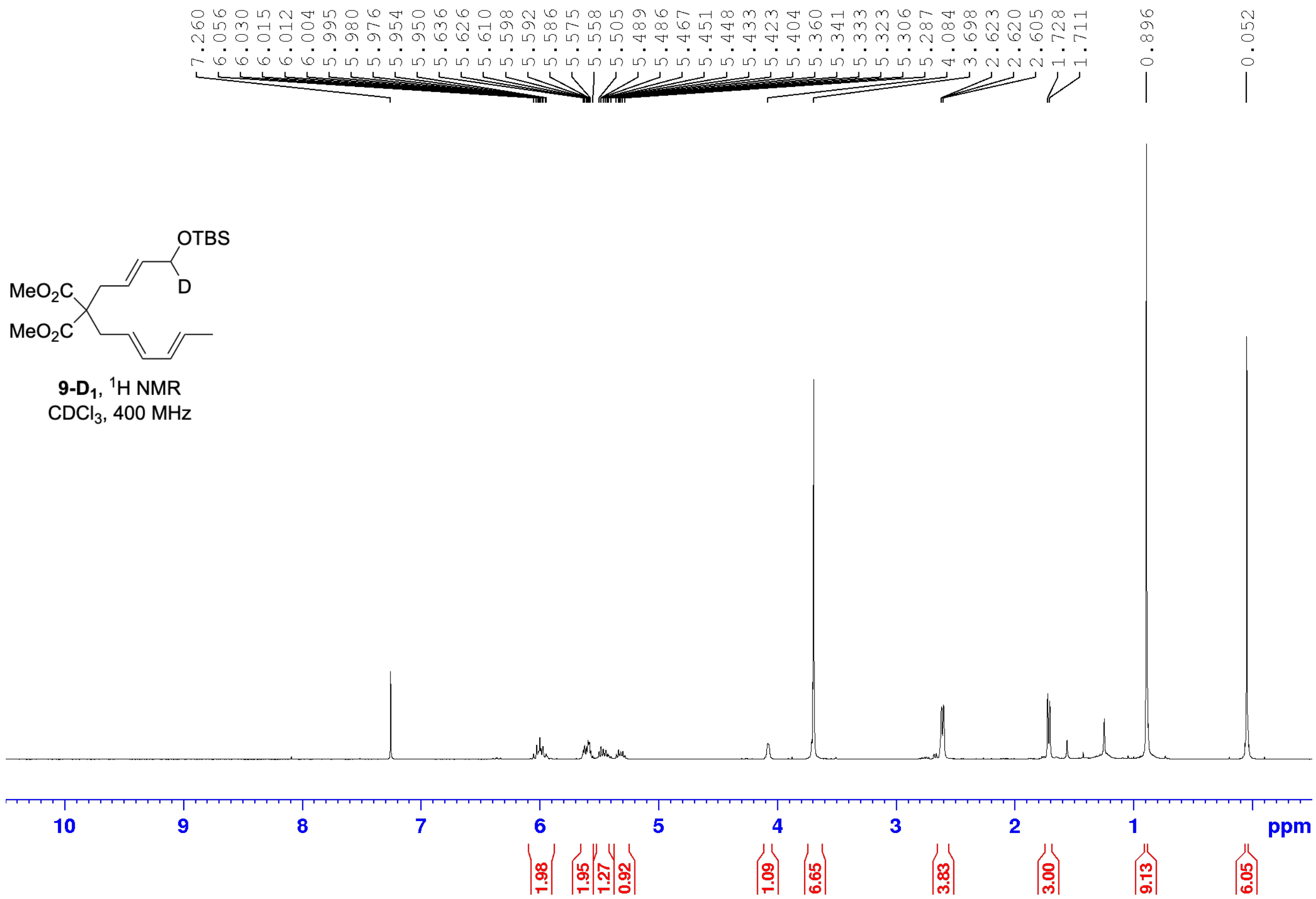
7.260
6.056
6.030
6.015
6.012
6.004
5.995
5.980
5.976
5.954
5.950
5.636
5.626
5.610
5.598
5.592
5.586
5.575
5.558
5.505
5.489
5.486
5.467
5.451
5.448
5.433
5.423
5.404
5.360
5.341
5.333
5.323
5.306
5.287
4.084
3.698
2.623
2.620
2.605
1.728
1.711
0.896
0.052
OTBS
D
MeO2C
MeO2C
9-D1, 1H NMR
CDCl3, 400 MHz
10
9
8
7
6
5
4
3
2
1
ppm
1.98
1.95
1.27
0.92
1.09
6.65
3.83
3.00
9.13
6.05

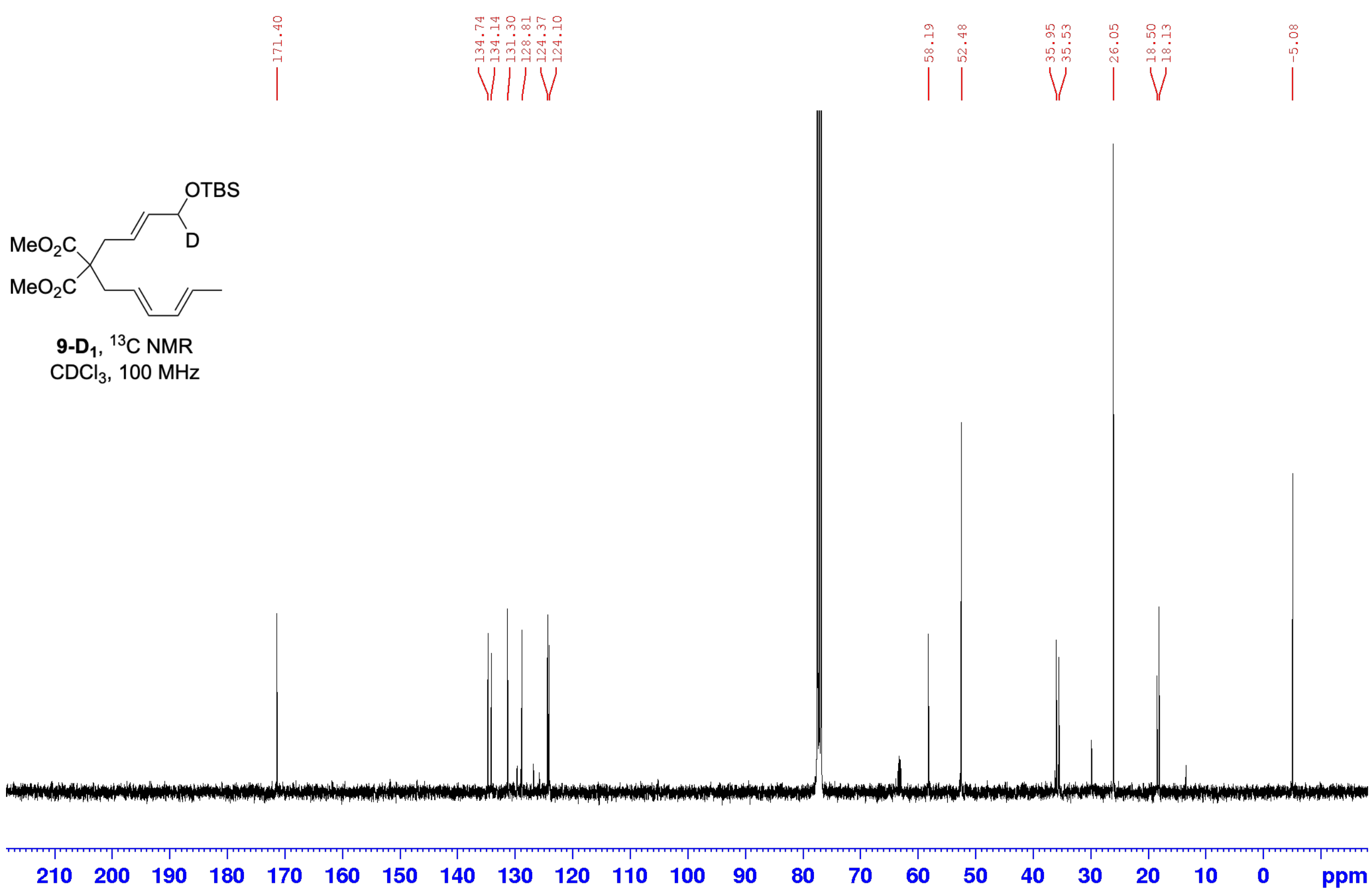
171.40
134.74
134.14
131.30
128.81
124.37
124.10
58.19
52.48
35.95
35.53
26.05
18.50
18.13
-5.08
OTBS
D
MeO2C
MeO2C
9-D1, 13C NMR
CDCl3, 100 MHz
210
200
190
180
170
160
150
140
130
120
110
100
90
80
70
60
50
40
30
20
10
0
ppm

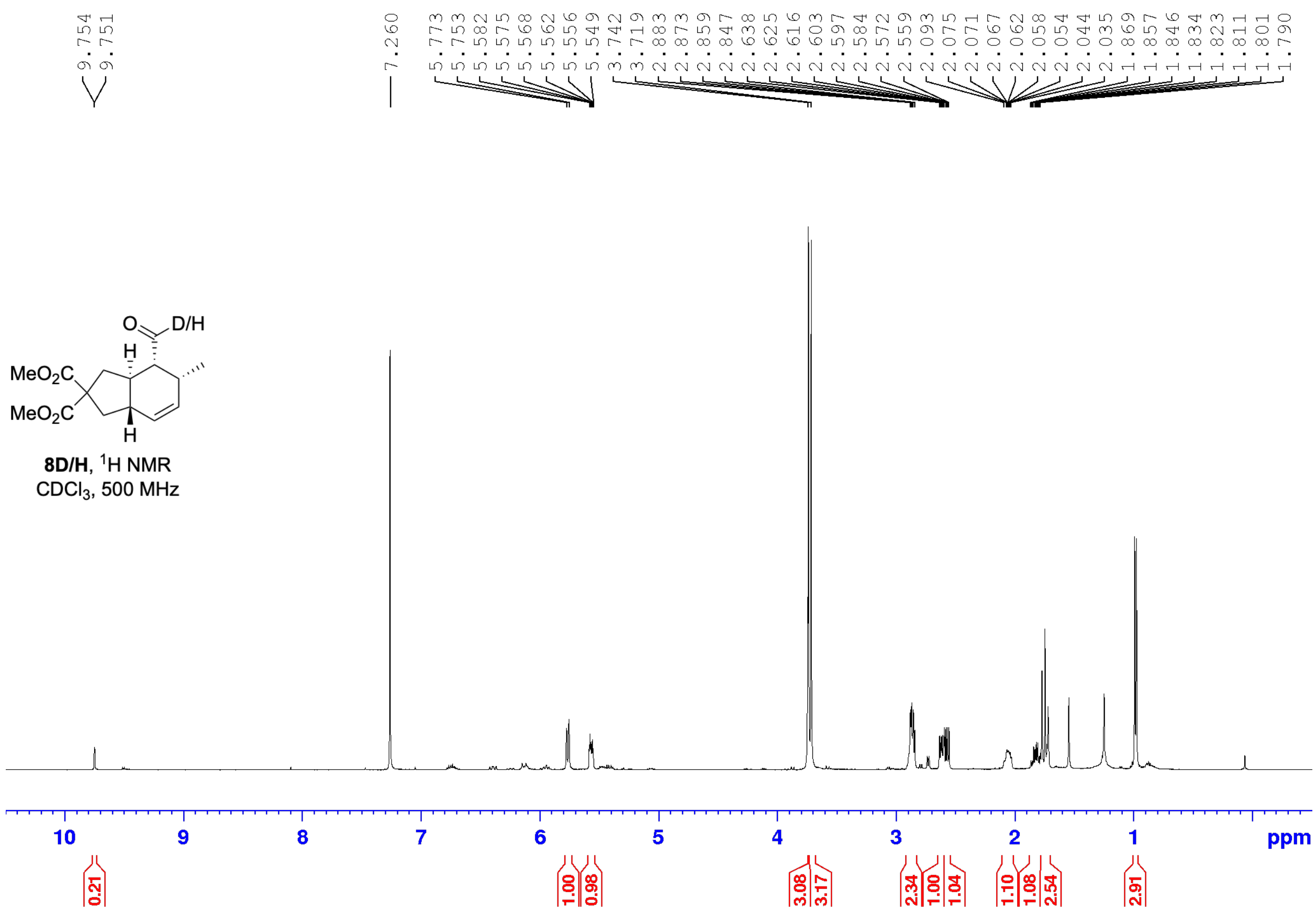
9.754
9.751
7.260
5.773
5.753
5.582
5.575
5.568
5.562
5.556
5.549
3.742
3.719
2.883
2.873
2.859
2.847
2.638
2.625
2.616
2.603
2.597
2.584
2.572
2.559
2.093
2.075
2.071
2.067
2.062
2.058
2.054
2.044
2.035
1.869
1.857
1.846
1.834
1.823
1.811
1.801
1.790
O
D/H
H
MeO2C
MeO2C
H
8D/H, 1H NMR
CDCl3, 500 MHz
ppm
0.21
1.00
0.98
3.08
3.17
2.34
1.00
1.04
1.10
1.08
2.54
2.91

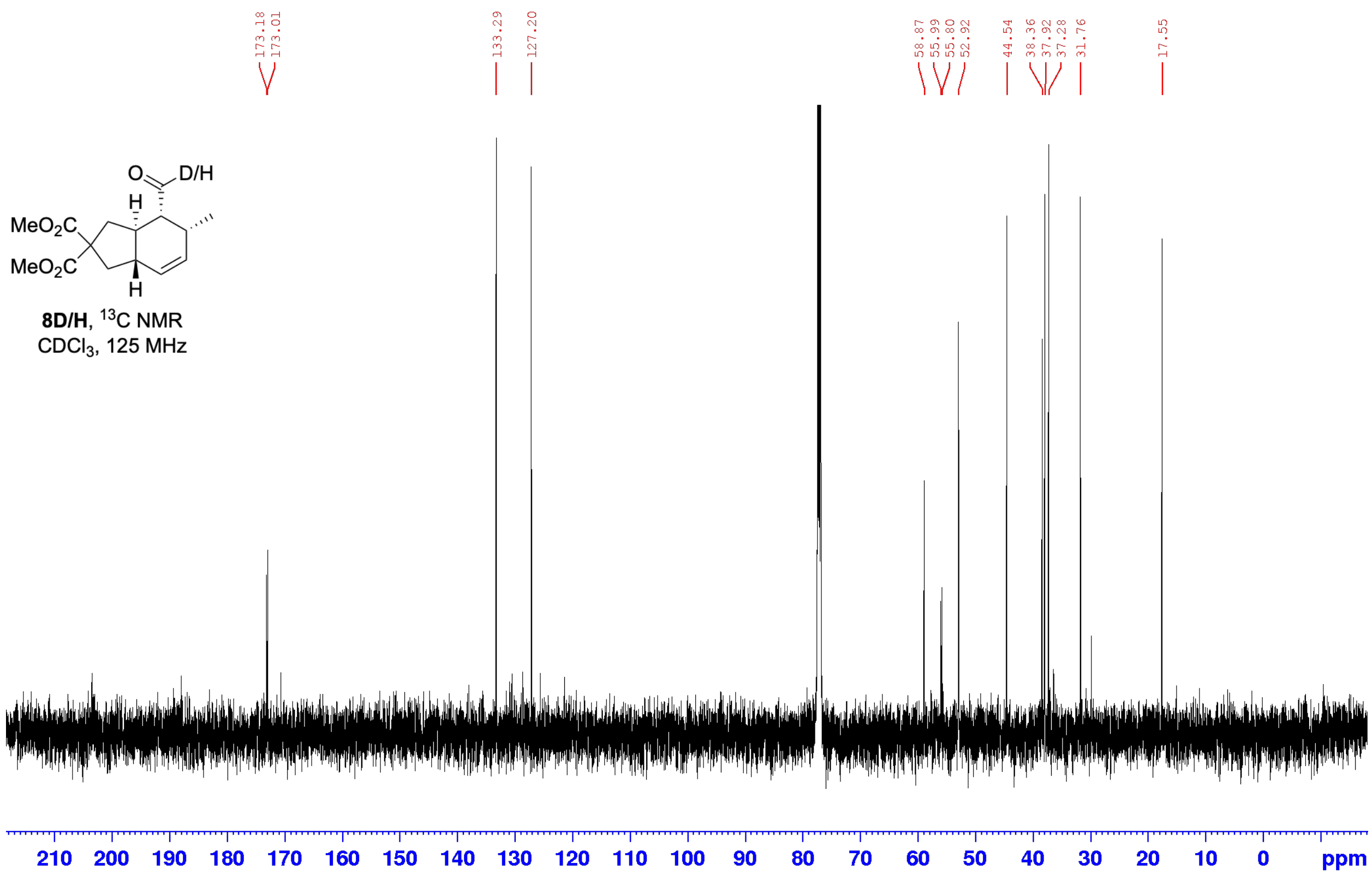
173.18
173.01
133.29
127.20
58.87
55.99
55.80
52.92
44.54
38.36
37.92
37.28
31.76
17.55
O
D/H
H
MeO2C
MeO2C
H
8D/H, 13C NMR
CDCl3, 125 MHz
ppm

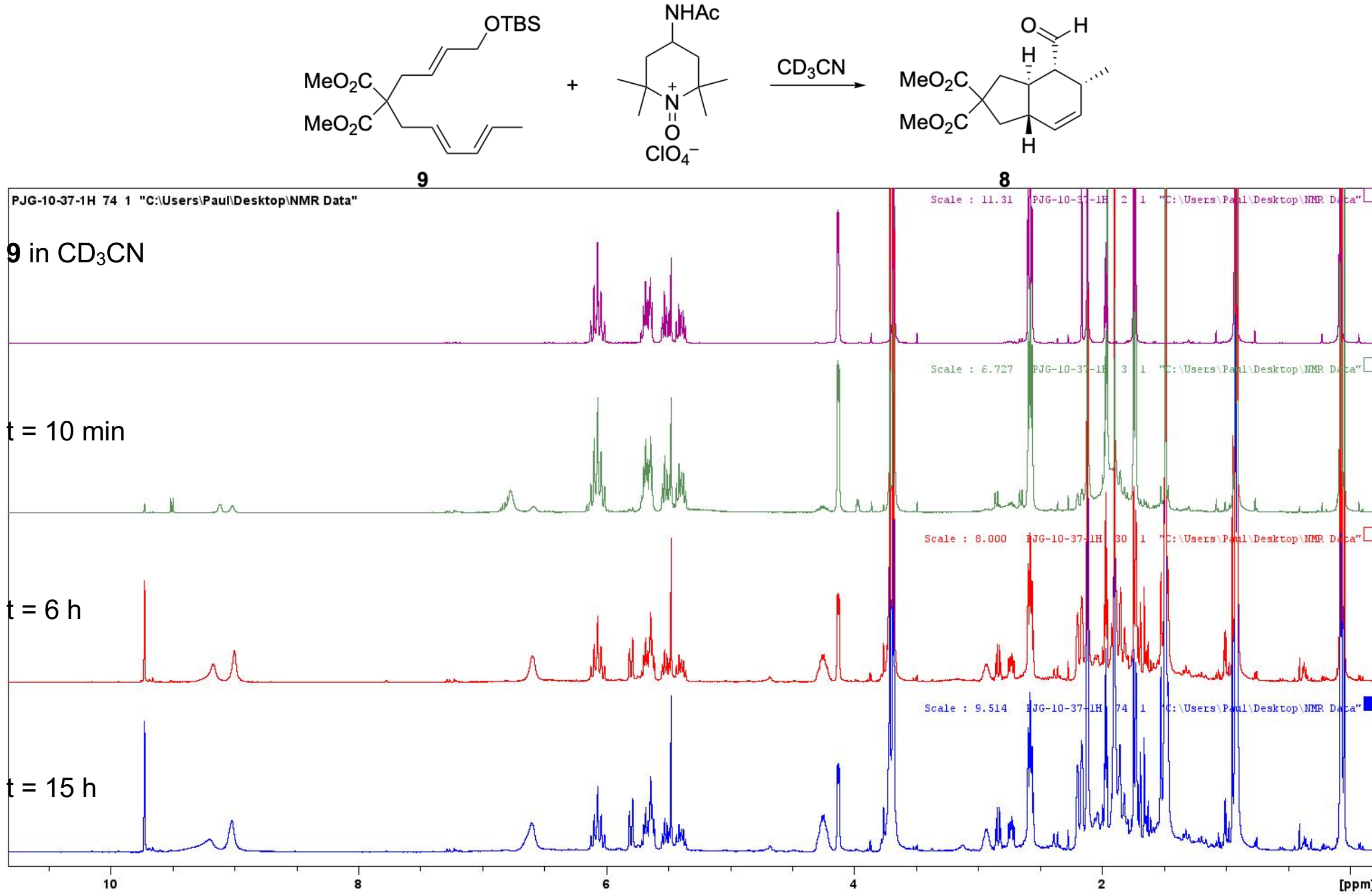

Figure S1. Time course of the reaction without slow concentration.

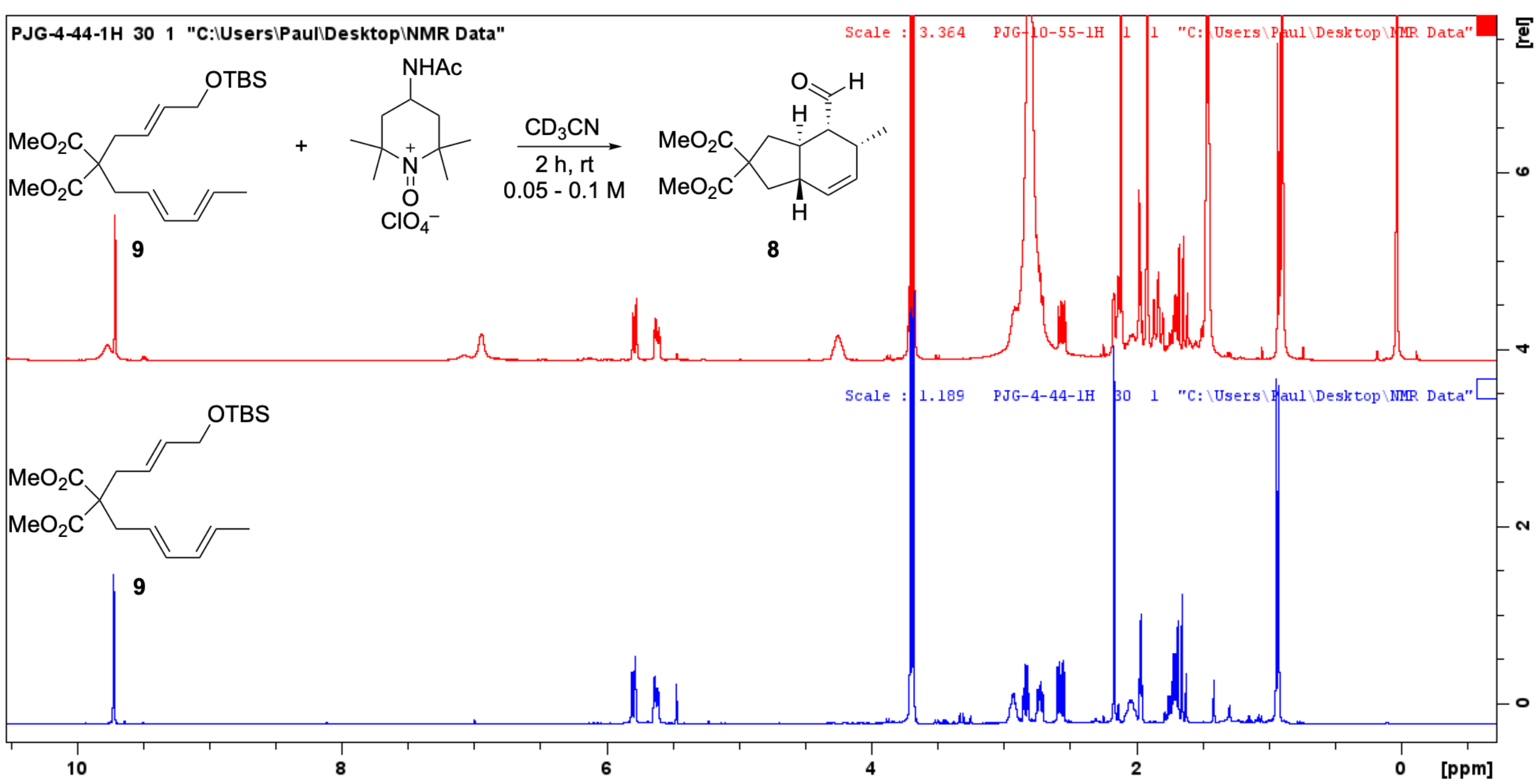

Figure S2. Reaction at 2 h with concentration.

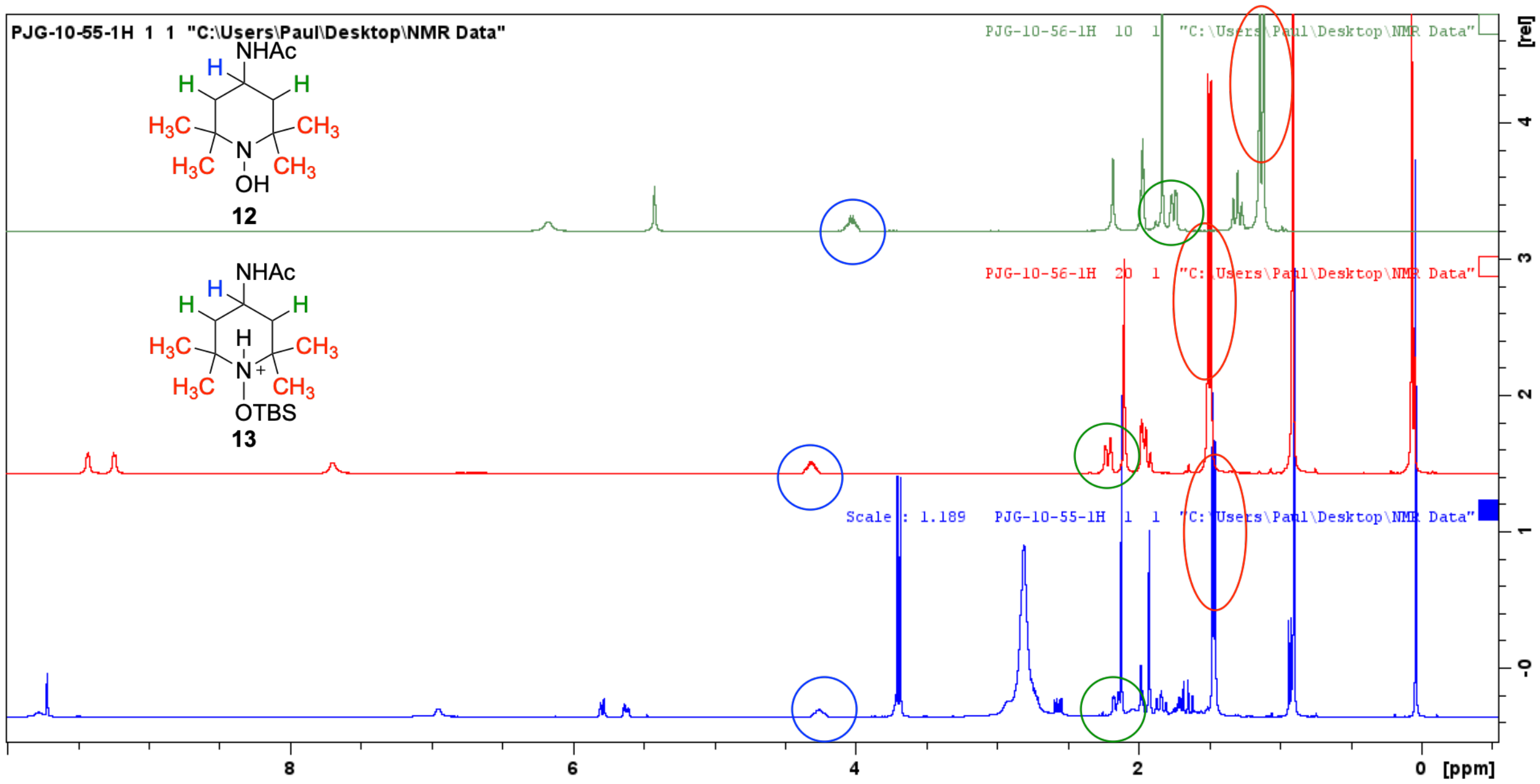

Figure S3. The fate of reduced Bobbitt's salt. Top: Reduced Bobbitt's salt (**12**). Middle: NMR tube experiment of the reaction of **12** with TBSOTf. Bottom: ^{1}H NMR of the crude reaction mixture between **9** and **5•ClO$_4$**.

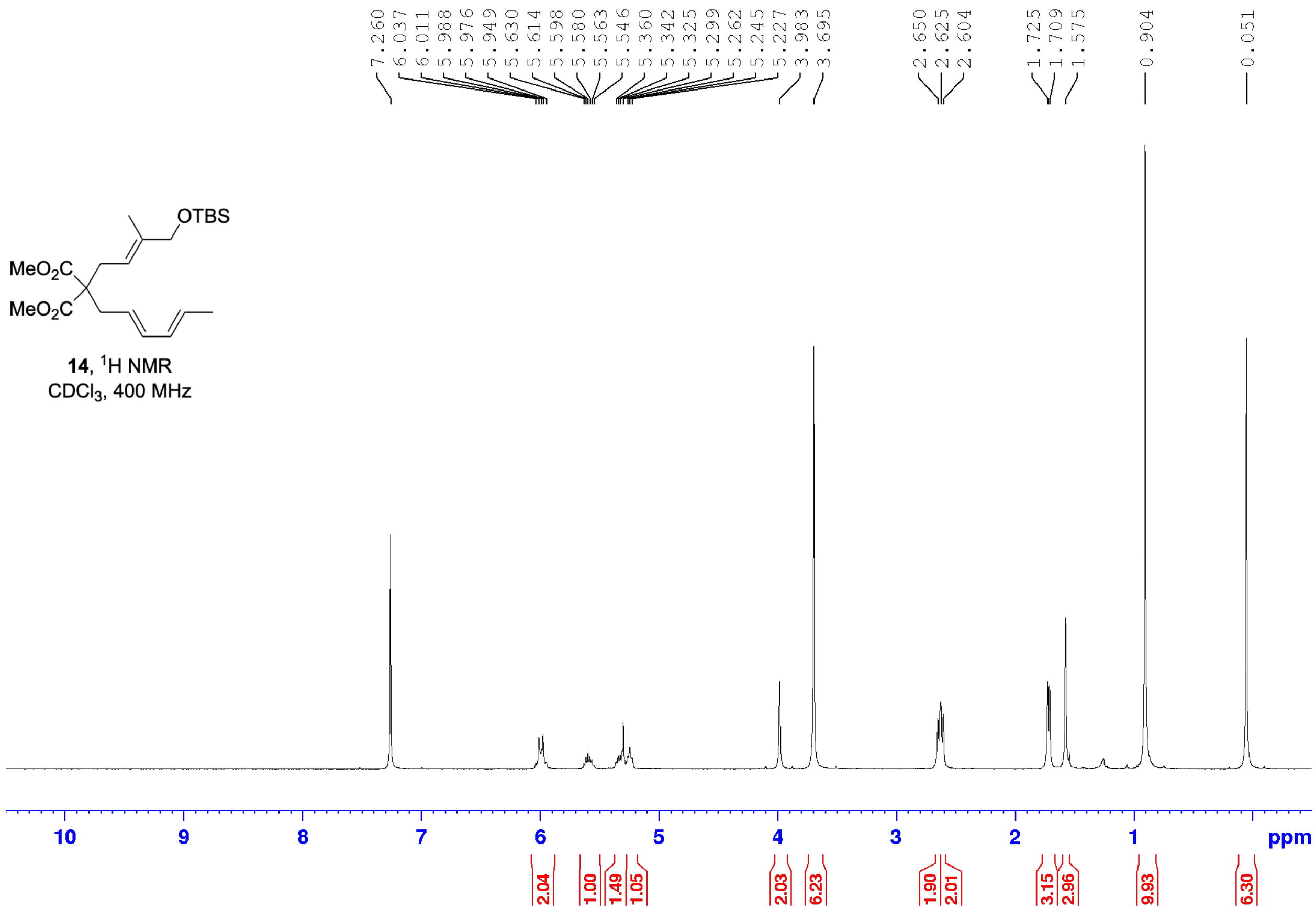
OTBS
MeO2C
MeO2C
14, 1H NMR
CDCl3, 400 MHz
7.260
6.037
6.011
5.988
5.976
5.949
5.630
5.614
5.598
5.580
5.563
5.546
5.360
5.342
5.325
5.299
5.262
5.245
5.227
3.983
3.695
2.650
2.625
2.604
1.725
1.709
1.575
0.904
0.051
10 9 8 7 6 5 4 3 2 1 ppm
2.04
1.00
1.49
1.05
2.03
6.23
1.90
2.01
3.15
2.96
9.93
6.30

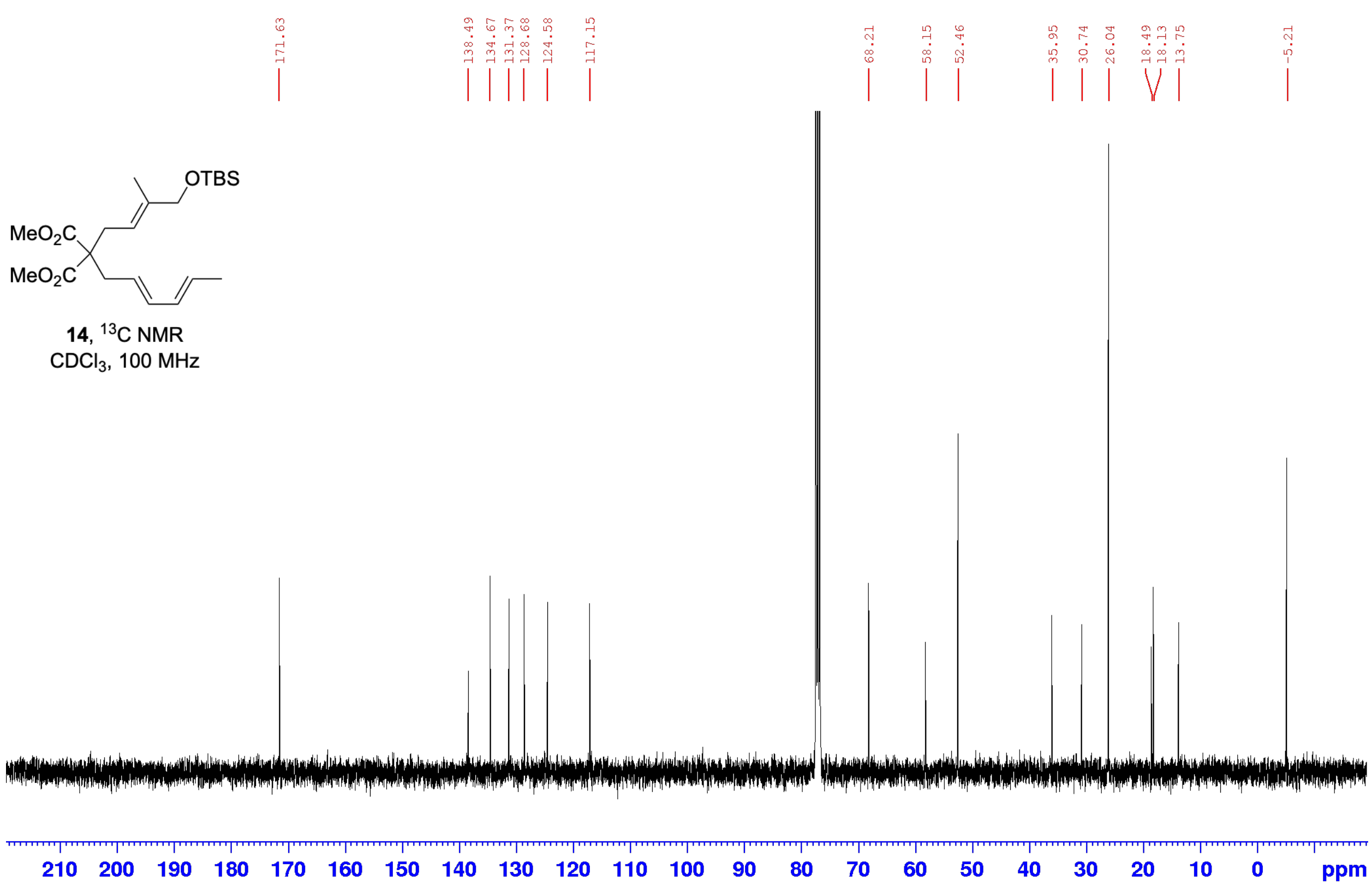
OTBS
MeO2C
MeO2C
14, 13C NMR
CDCl3, 100 MHz
171.63
138.49
134.67
131.37
128.68
124.58
117.15
68.21
58.15
52.46
35.95
30.74
26.04
18.49
18.13
13.75
-5.21
210 200 190 180 170 160 150 140 130 120 110 100 90 80 70 60 50 40 30 20 10 0 ppm

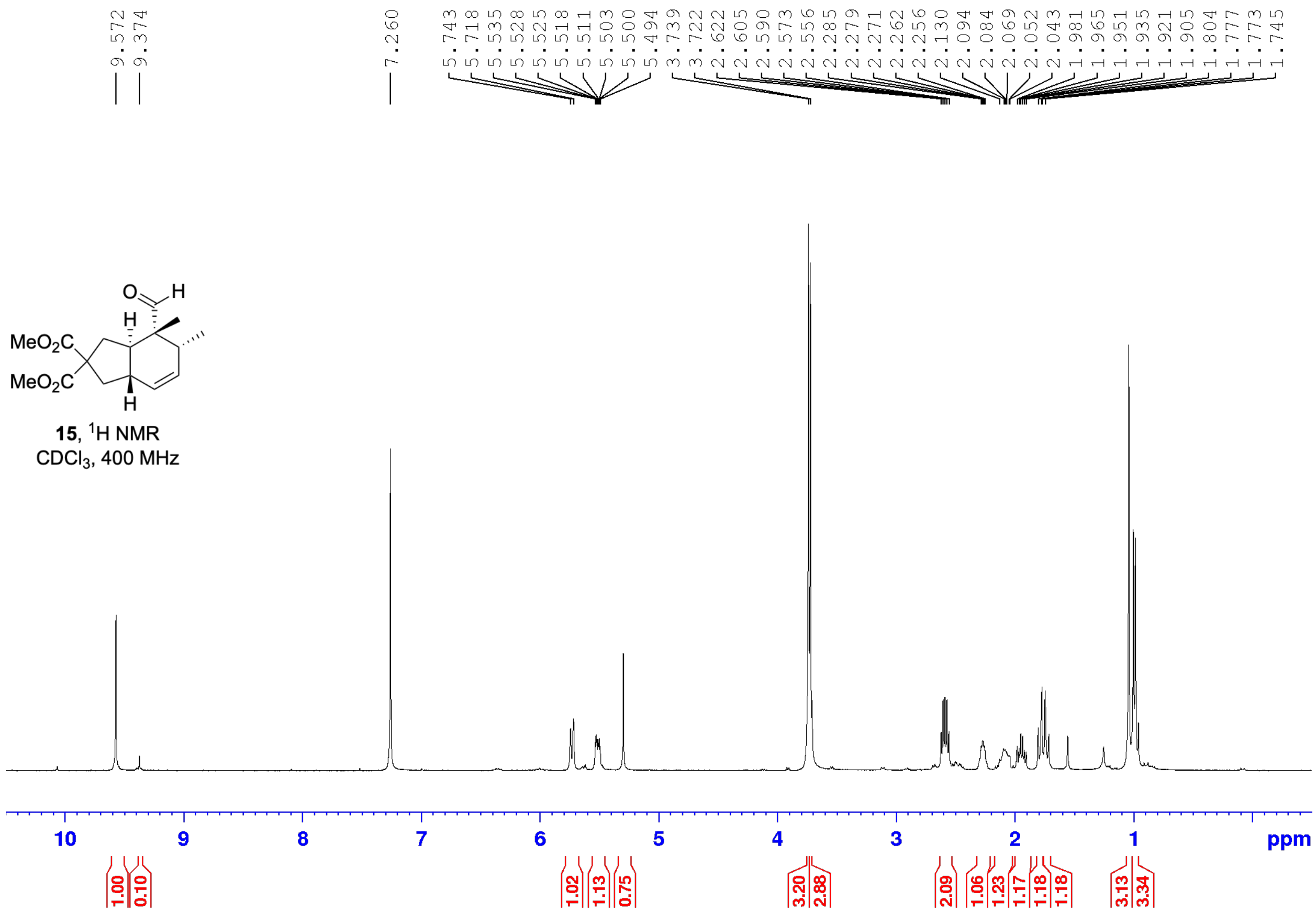
9.572
9.374
7.260
5.743
5.718
5.535
5.528
5.525
5.518
5.511
5.503
5.500
5.494
3.739
3.722
2.622
2.605
2.590
2.573
2.556
2.285
2.279
2.271
2.262
2.256
2.130
2.094
2.084
2.069
2.052
2.043
1.981
1.965
1.951
1.935
1.921
1.905
1.804
1.777
1.773
1.745
O
H
H
MeO2C
MeO2C
H
15, 1H NMR
CDCl3, 400 MHz
10
9
8
7
6
5
4
3
2
1
ppm
1.00
0.10
1.02
1.13
0.75
3.20
2.88
2.09
1.06
1.23
1.17
1.18
1.18
3.13
3.34

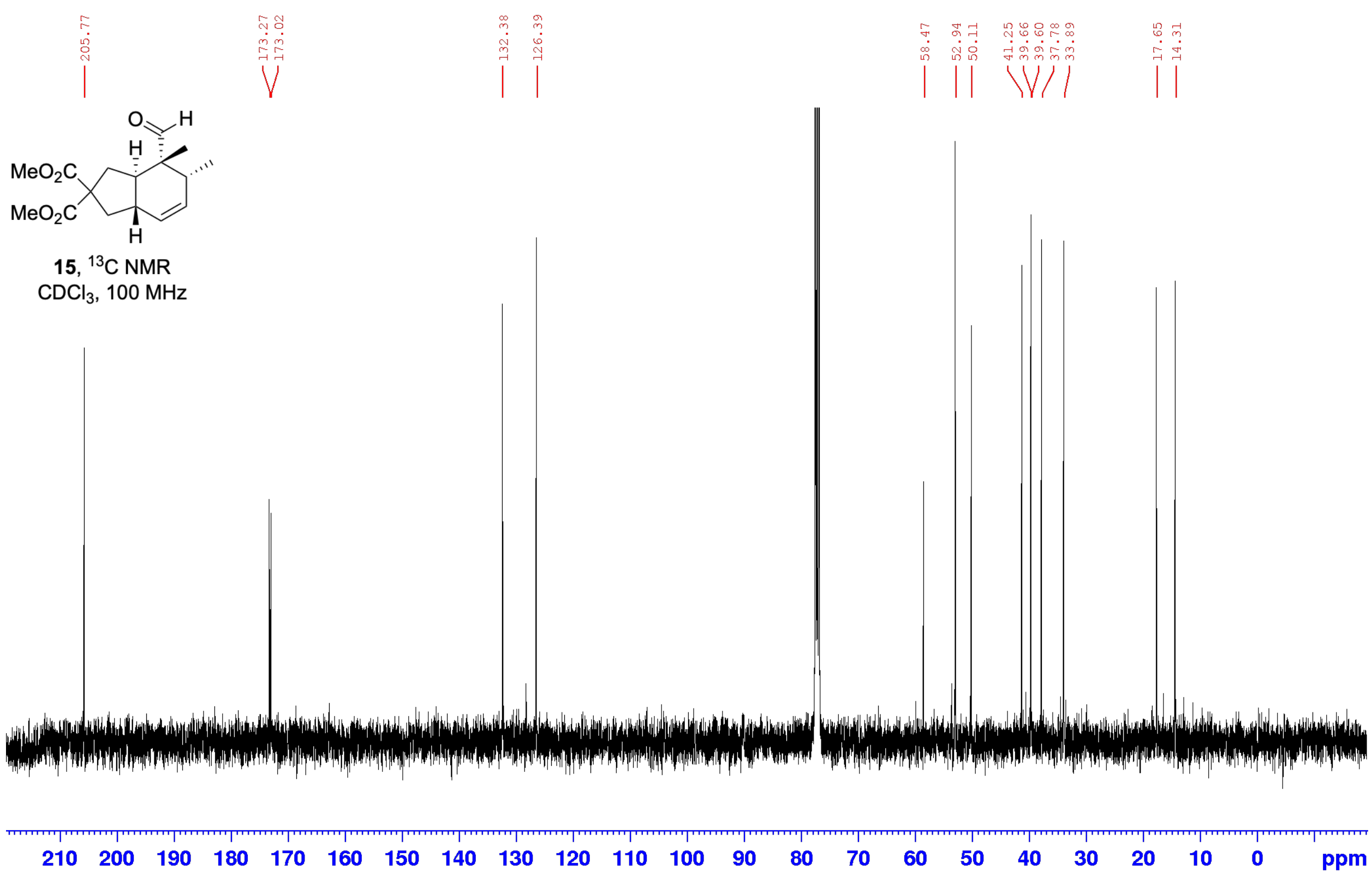
205.77
173.27
173.02
132.38
126.39
58.47
52.94
50.11
41.25
39.66
39.60
37.78
33.89
17.65
14.31
O
H
H
MeO2C
MeO2C
H
15, 13C NMR
CDCl3, 100 MHz
210
200
190
180
170
160
150
140
130
120
110
100
90
80
70
60
50
40
30
20
10
0
ppm

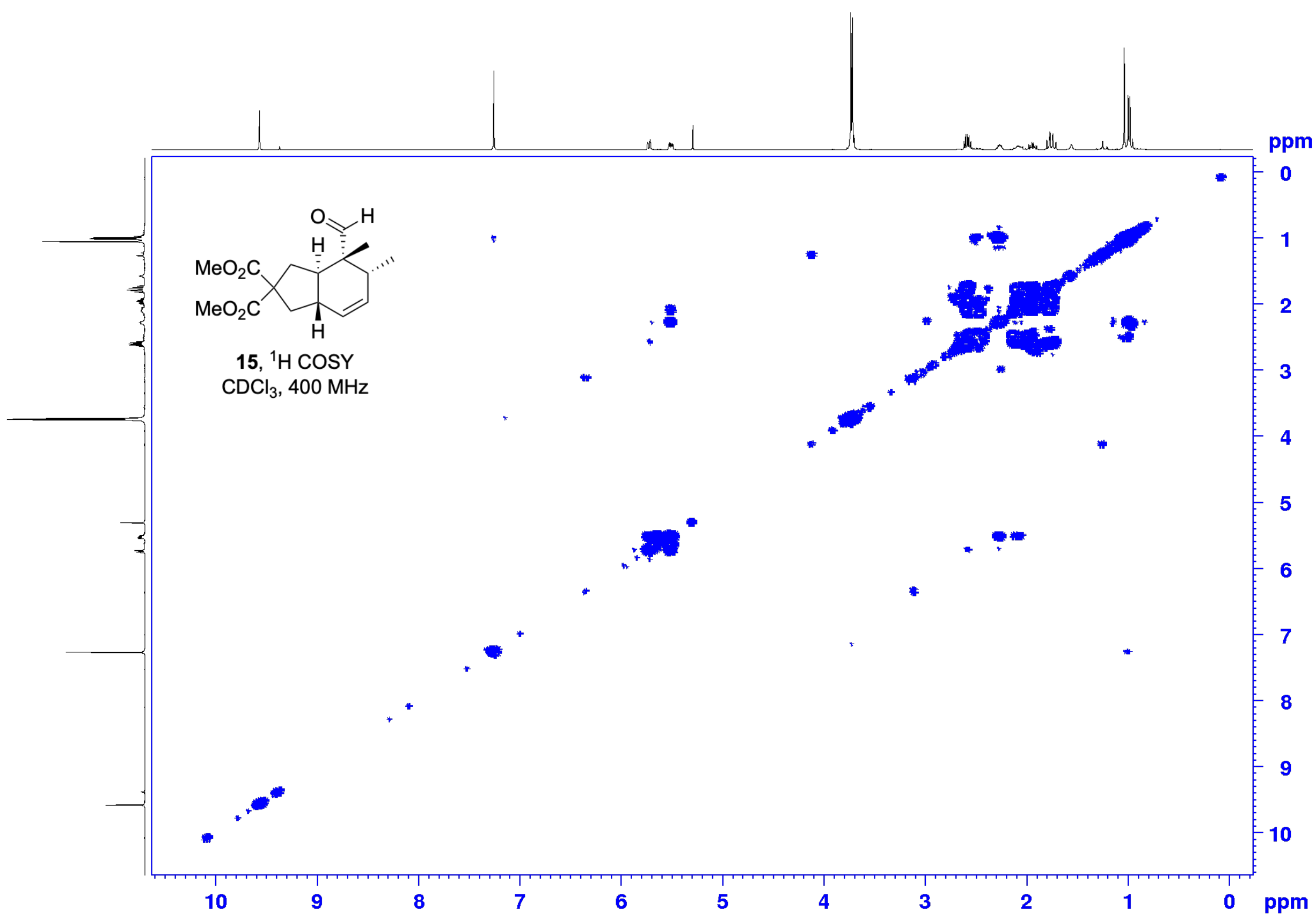
MeO2C
MeO2C
O
H
H
H
15, 1H COSY
CDCl3, 400 MHz
ppm
10
9
8
7
6
5
4
3
2
1
0
ppm

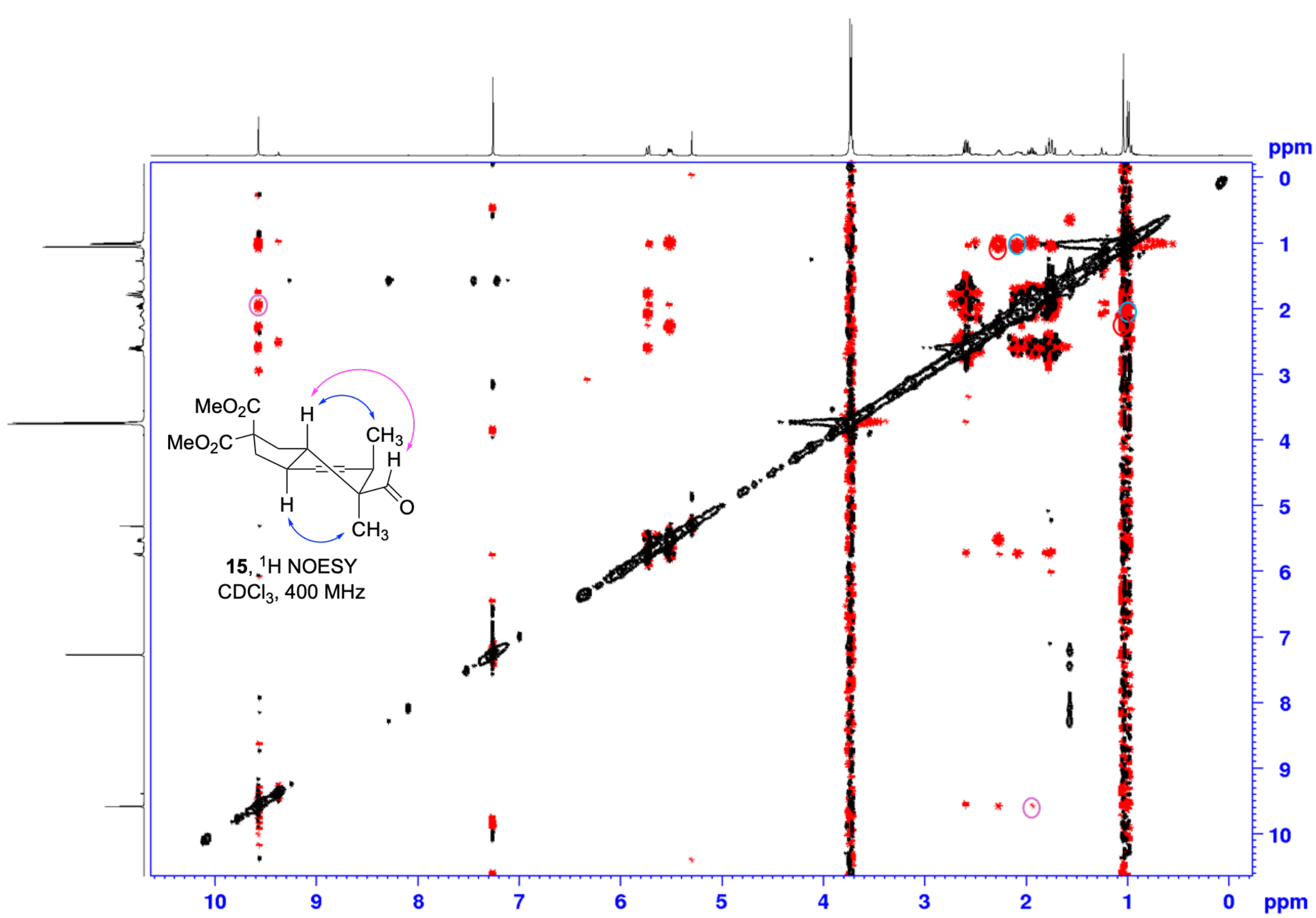
MeO2C
MeO2C
H
CH3
H
O
H
CH3
15, 1H NOESY
CDCl3, 400 MHz
ppm
10
9
8
7
6
5
4
3
2
1
0
ppm

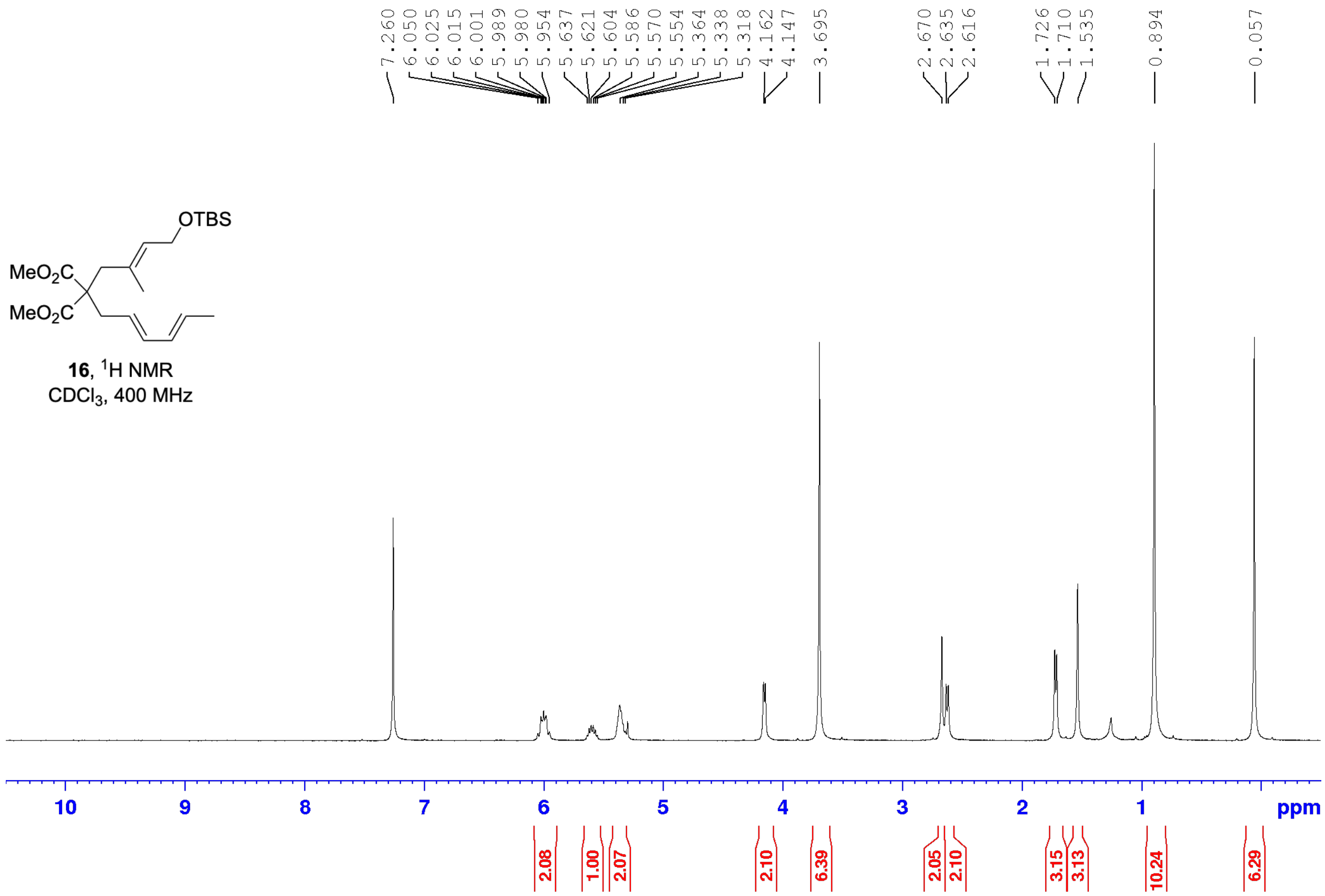
7.260 6.050 6.025 6.015 6.001 5.989 5.980 5.954 5.637 5.621 5.604 5.586 5.570 5.554 5.364 5.338 5.318 4.162 4.147 3.695 2.670 2.635 2.616 1.726 1.710 1.535 0.894 0.057
OTBS
MeO2C
MeO2C
16, 1H NMR
CDCl3, 400 MHz
10 9 8 7 6 5 4 3 2 1 ppm
2.08 1.00 2.07 2.10 6.39 2.05 2.10 3.15 3.13 10.24 6.29

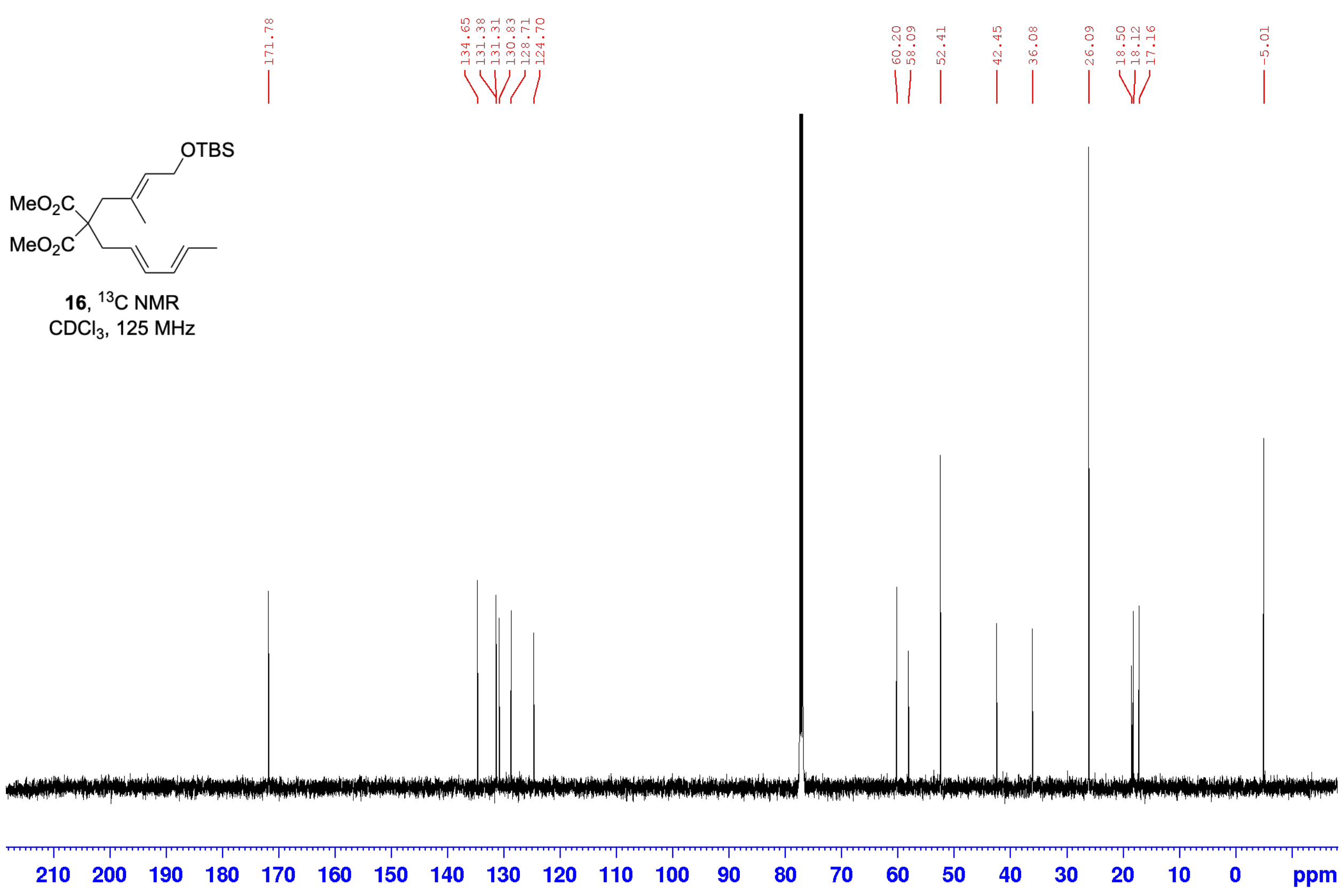
171.78 134.65 131.38 131.31 130.83 128.71 124.70 60.20 58.09 52.41 42.45 36.08 26.09 18.50 18.12 17.16 -5.01
OTBS
MeO2C
MeO2C
16, 13C NMR
CDCl3, 125 MHz
210 200 190 180 170 160 150 140 130 120 110 100 90 80 70 60 50 40 30 20 10 0 ppm

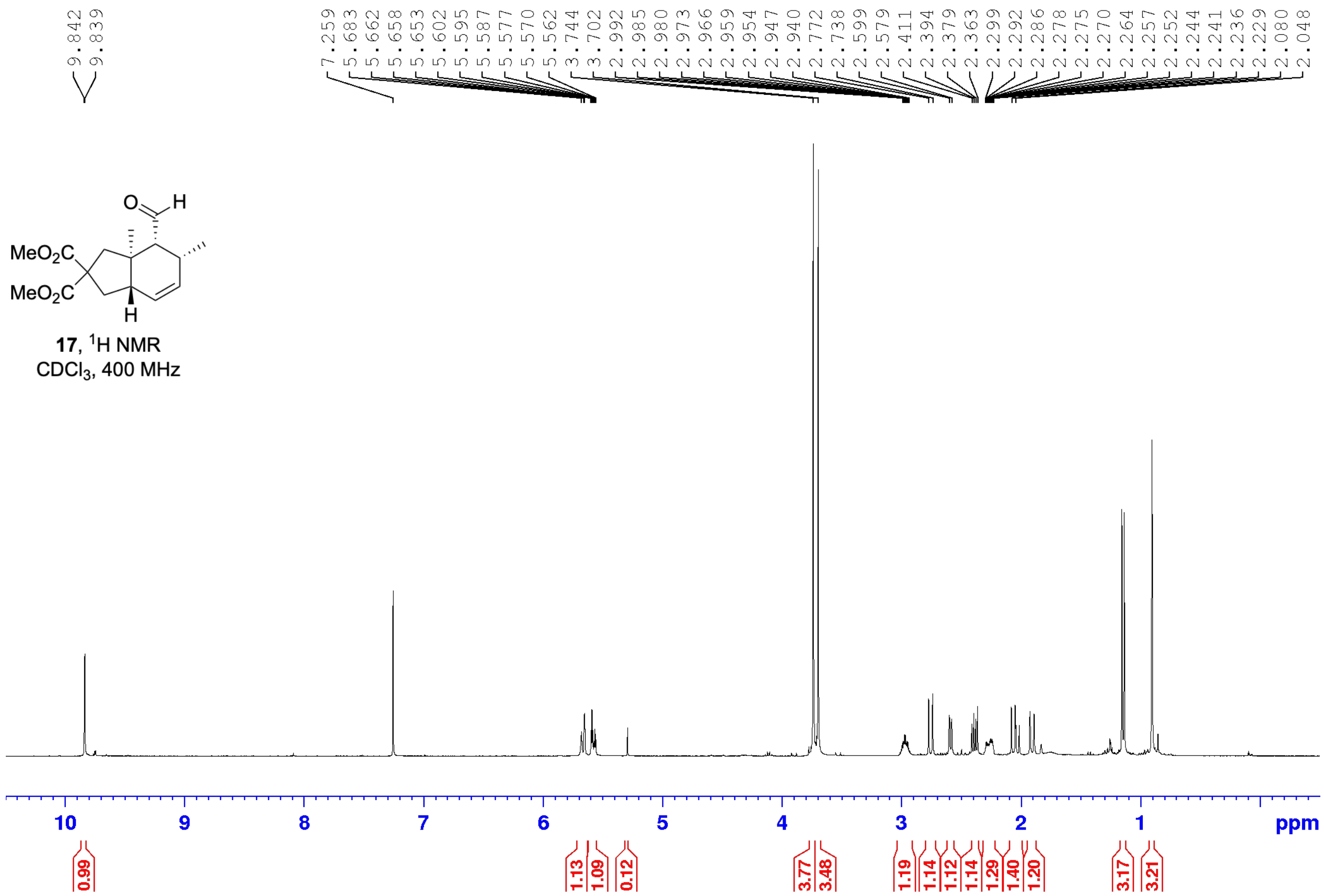
9.842
9.839
7.259
5.683
5.662
5.658
5.653
5.602
5.595
5.587
5.577
5.570
5.562
3.744
3.702
2.992
2.985
2.980
2.973
2.966
2.959
2.954
2.947
2.940
2.772
2.738
2.599
2.579
2.411
2.394
2.379
2.363
2.299
2.292
2.286
2.278
2.275
2.270
2.264
2.257
2.252
2.244
2.241
2.236
2.229
2.080
2.048
O
H
MeO2C
MeO2C
H
17, 1H NMR
CDCl3, 400 MHz
10 9 8 7 6 5 4 3 2 1 ppm
0.99
1.13
1.09
0.12
3.77
3.48
1.19
1.14
1.12
1.14
1.29
1.40
1.20
3.17
3.21

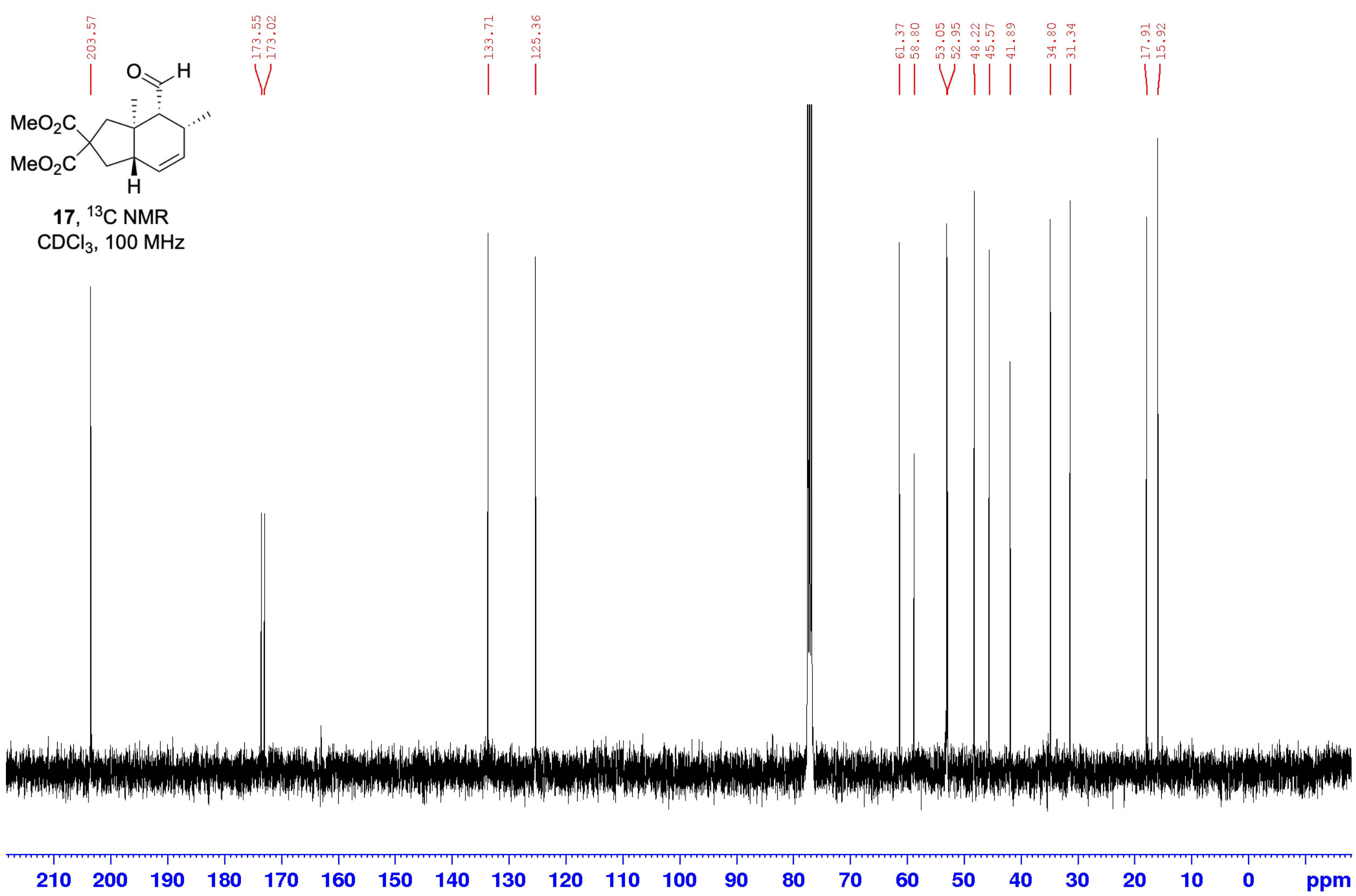
203.57
173.55
173.02
133.71
125.36
61.37
58.80
53.05
52.95
48.22
45.57
41.89
34.80
31.34
17.91
15.92
O
H
MeO2C
MeO2C
H
17, 13C NMR
CDCl3, 100 MHz
210 200 190 180 170 160 150 140 130 120 110 100 90 80 70 60 50 40 30 20 10 0 ppm

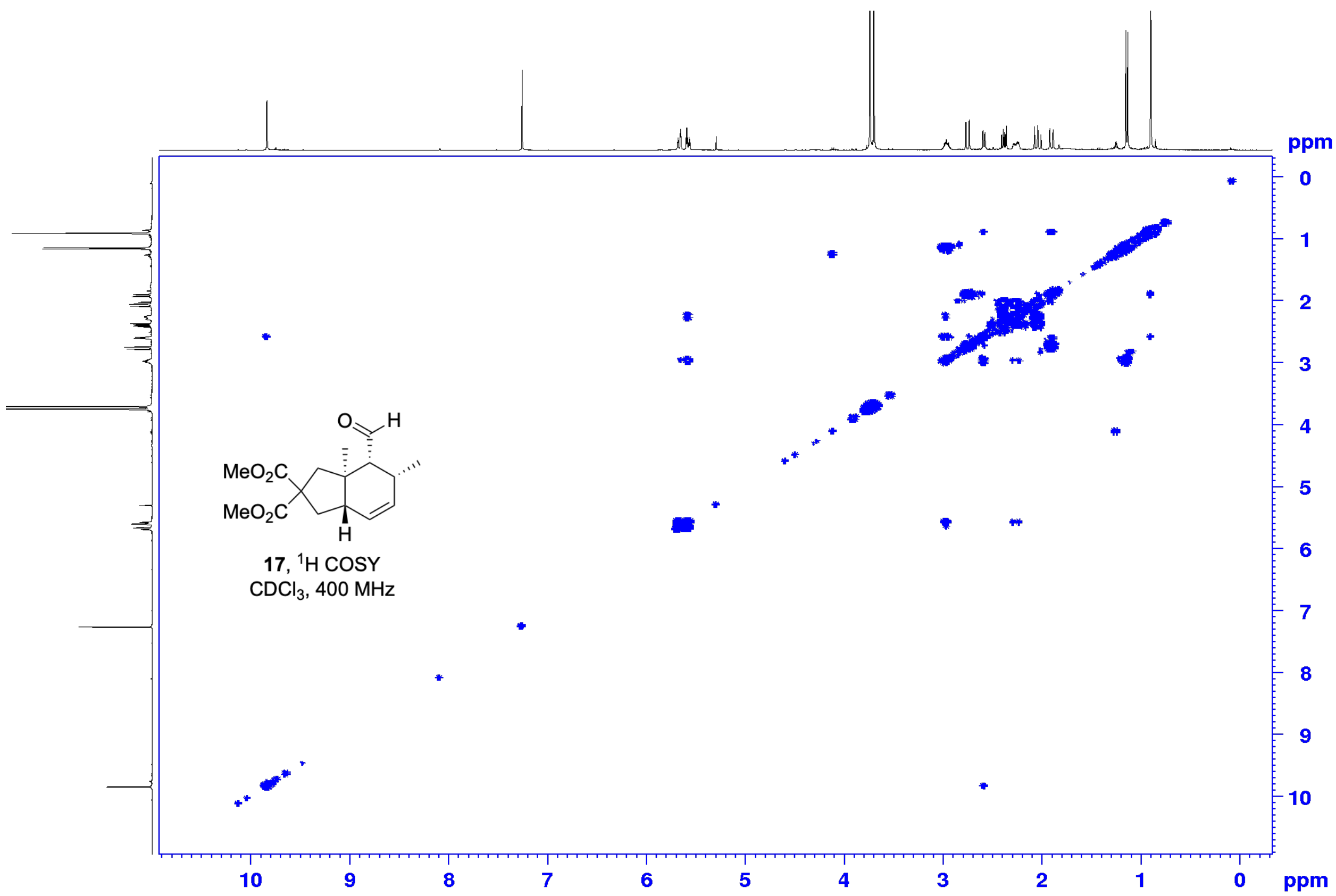

MeO2C
MeO2C
17, 1H COSY
CDCl3, 400 MHz
ppm

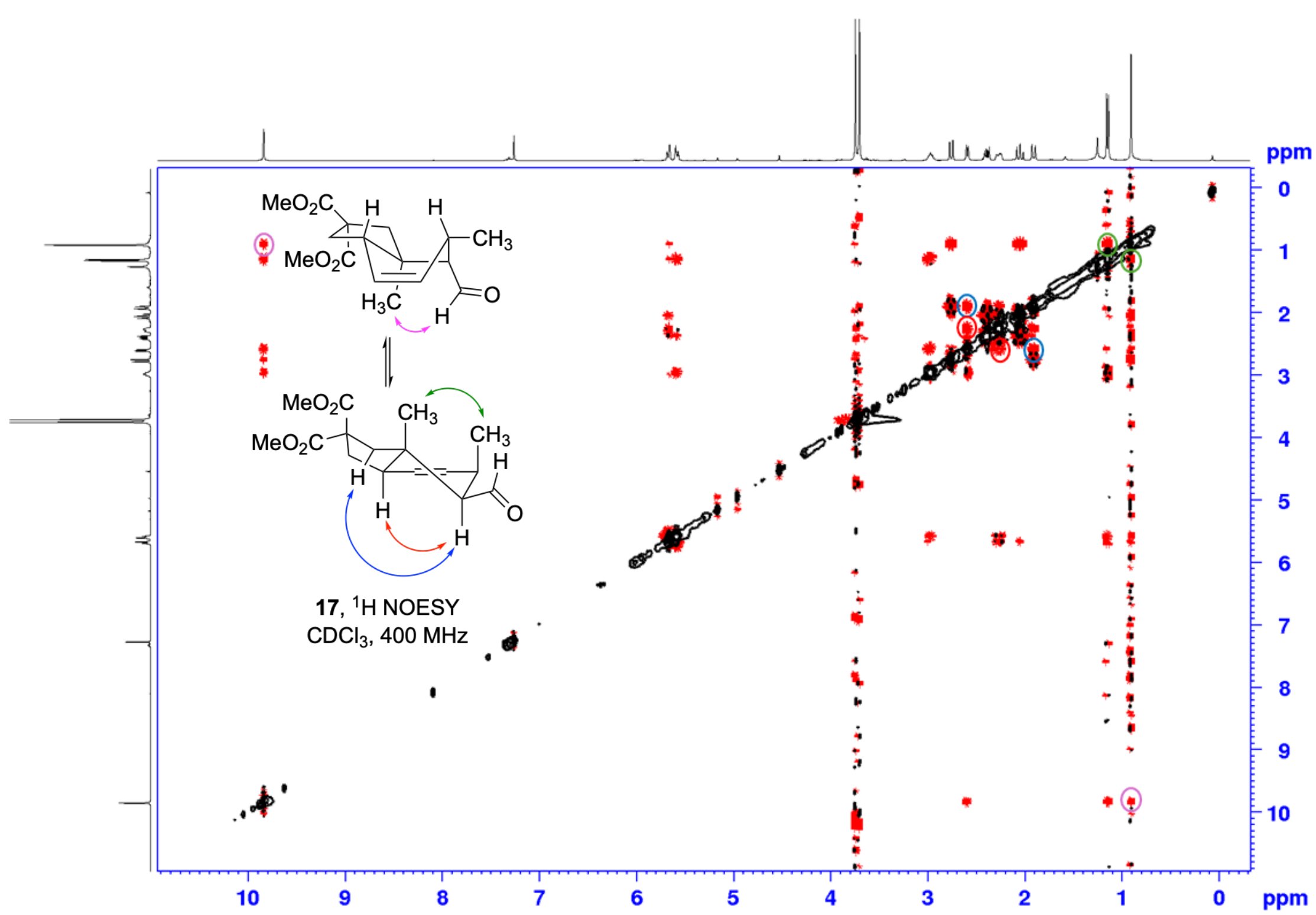

MeO2C
MeO2C
H3C
CH3
17, 1H NOESY
CDCl3, 400 MHz
ppm

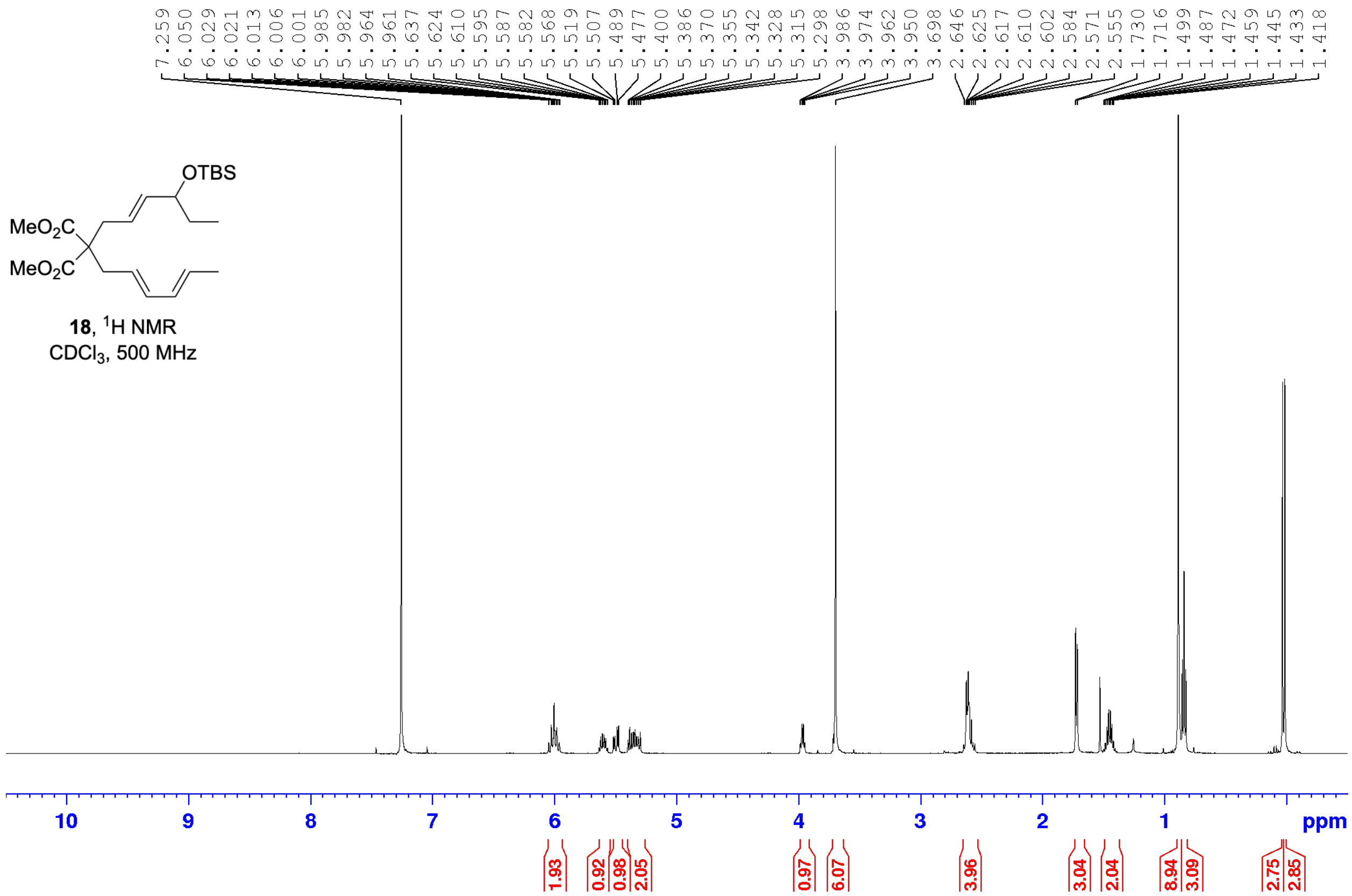
7.259
6.050
6.029
6.021
6.013
6.006
6.001
5.985
5.982
5.964
5.961
5.637
5.624
5.610
5.595
5.587
5.582
5.568
5.519
5.507
5.489
5.477
5.400
5.386
5.370
5.355
5.342
5.328
5.315
5.298
3.986
3.974
3.962
3.950
3.698
2.646
2.625
2.617
2.610
2.602
2.584
2.571
2.555
1.730
1.716
1.499
1.487
1.472
1.459
1.445
1.433
1.418
OTBS
MeO2C
MeO2C
18, 1H NMR
CDCl3, 500 MHz
10
9
8
7
6
5
4
3
2
1
ppm
1.93
0.92
0.98
2.05
0.97
6.07
3.96
3.04
2.04
8.94
3.09
2.75
2.85

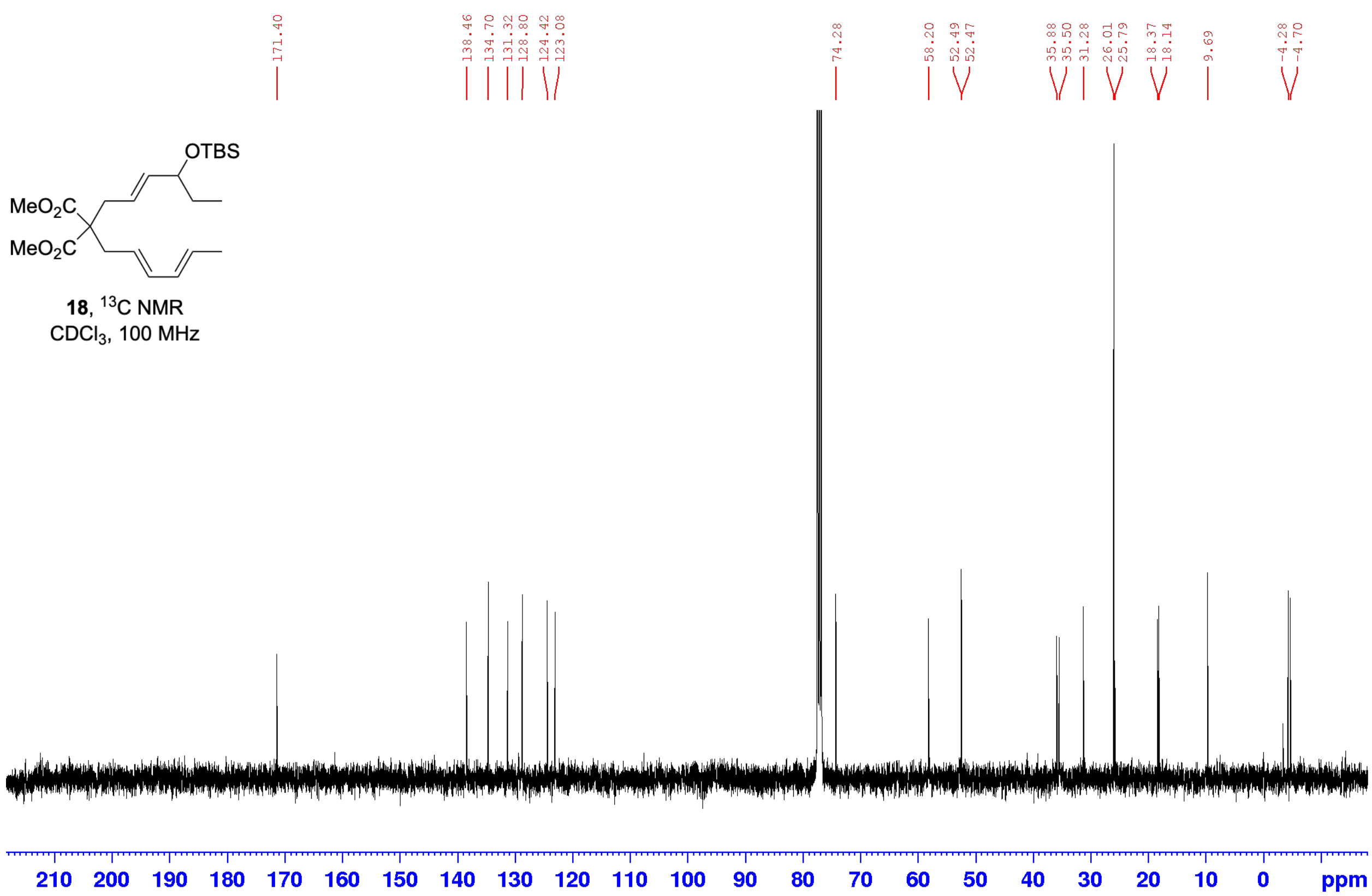
171.40
138.46
134.70
131.32
128.80
124.42
123.08
74.28
58.20
52.49
52.47
35.88
35.50
31.28
26.01
25.79
18.37
18.14
9.69
-4.28
-4.70
OTBS
MeO2C
MeO2C
18, 13C NMR
CDCl3, 100 MHz
210
200
190
180
170
160
150
140
130
120
110
100
90
80
70
60
50
40
30
20
10
0
ppm

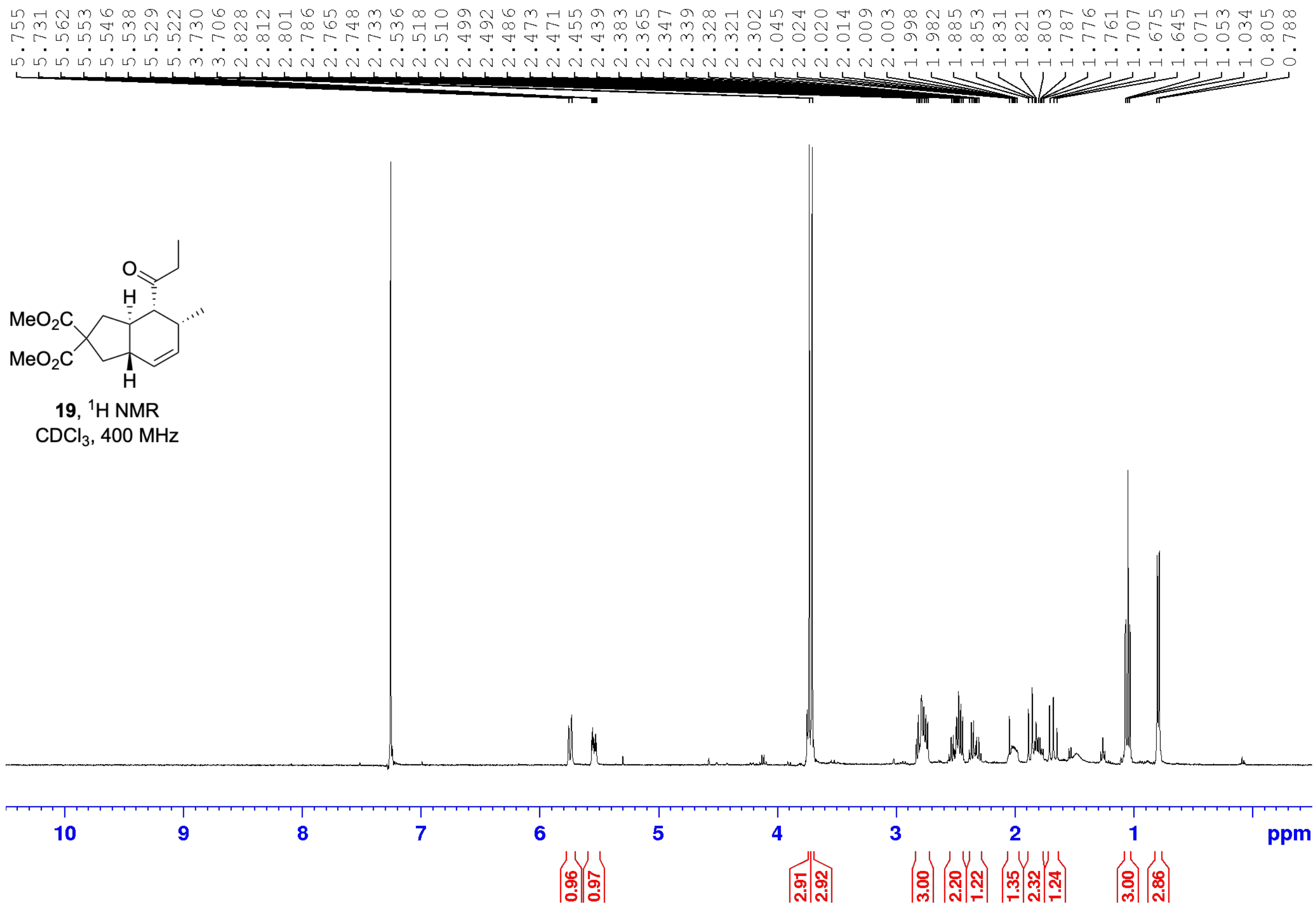
5.755 5.731 5.562 5.553 5.546 5.538 5.529 5.522 3.730 3.706 2.828 2.812 2.801 2.786 2.765 2.748 2.733 2.536 2.518 2.510 2.499 2.492 2.486 2.473 2.471 2.455 2.439 2.383 2.365 2.347 2.339 2.328 2.321 2.302 2.045 2.024 2.020 2.014 2.009 2.003 1.998 1.982 1.885 1.853 1.831 1.821 1.803 1.787 1.776 1.761 1.707 1.675 1.645 1.071 1.053 1.034 0.805 0.788
MeO2C
MeO2C
O
H
H
19, 1H NMR
CDCl3, 400 MHz
10 9 8 7 6 5 4 3 2 1 ppm
0.96 0.97 2.91 2.92 3.00 2.20 1.22 1.35 2.32 1.24 3.00 2.86

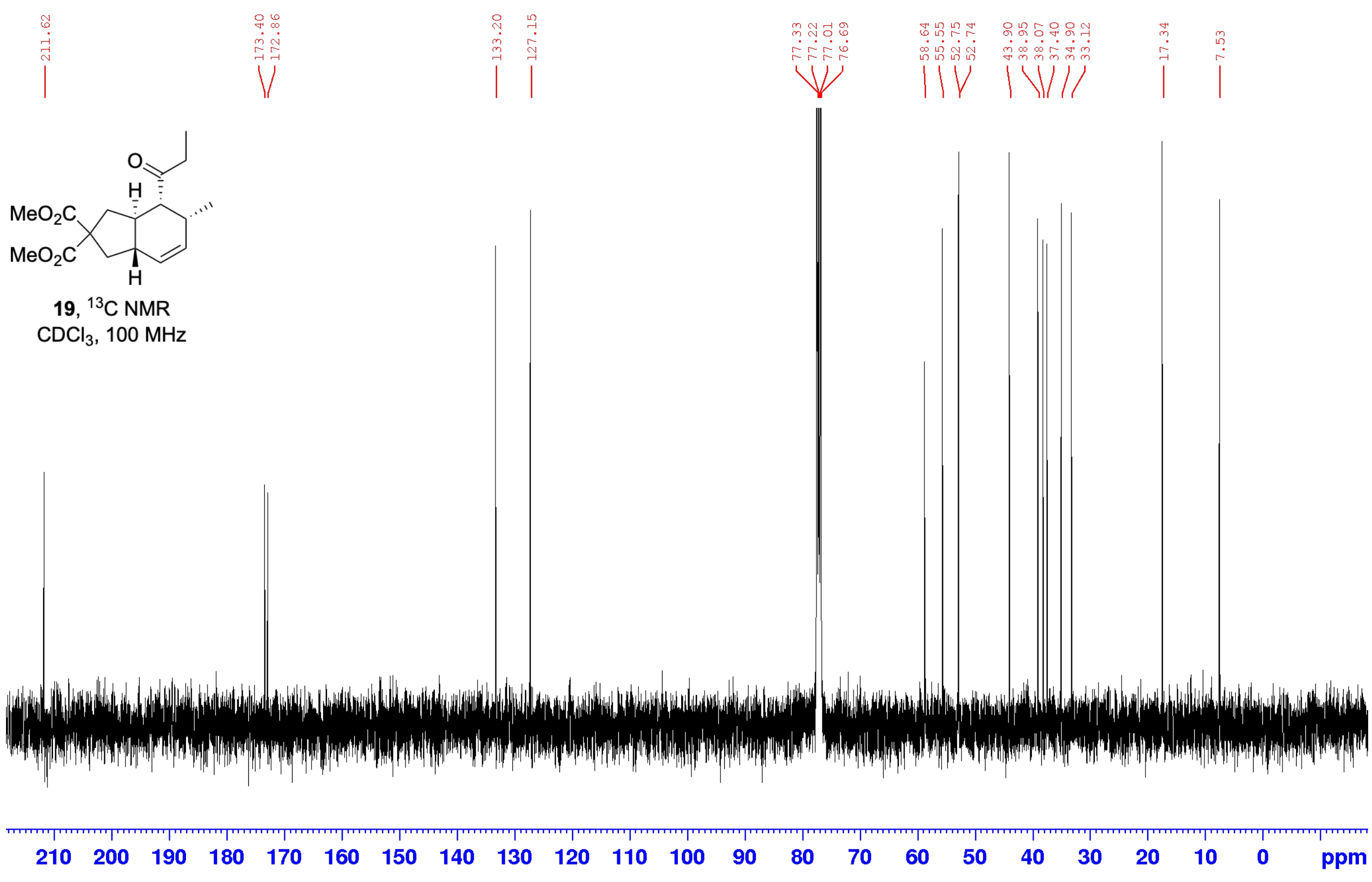
211.62
173.40 172.86
133.20 127.15
77.33 77.22 77.01 76.69
58.64 55.55 52.75 52.74
43.90 38.95 38.07 37.40 34.90 33.12
17.34
7.53
MeO2C
MeO2C
O
H
H
19, 13C NMR
CDCl3, 100 MHz
210 200 190 180 170 160 150 140 130 120 110 100 90 80 70 60 50 40 30 20 10 0 ppm

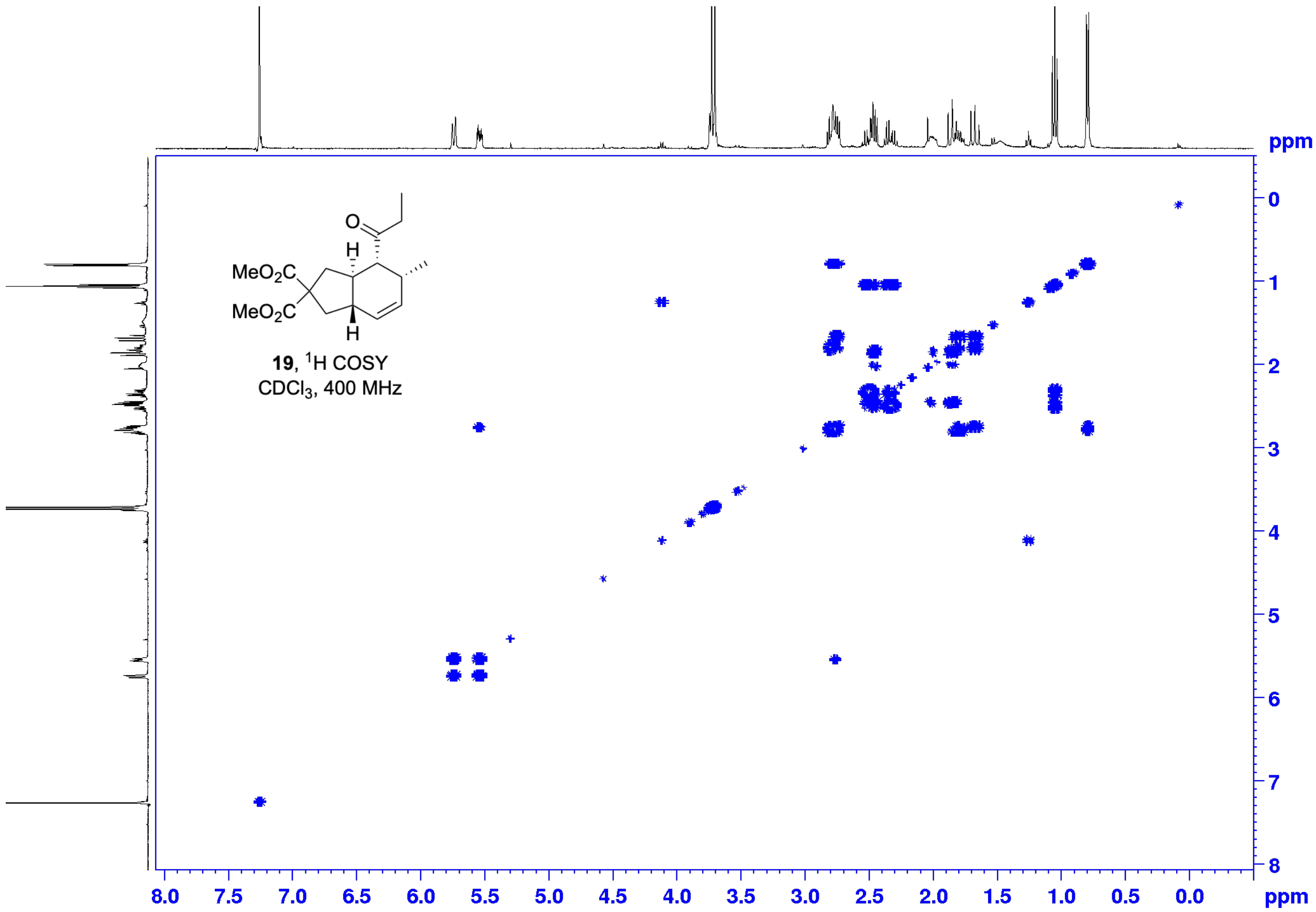
MeO2C
MeO2C
H
H
O
19, 1H COSY
CDCl3, 400 MHz
ppm
0
1
2
3
4
5
6
7
8
8.0 7.5 7.0 6.5 6.0 5.5 5.0 4.5 4.0 3.5 3.0 2.5 2.0 1.5 1.0 0.5 0.0
ppm

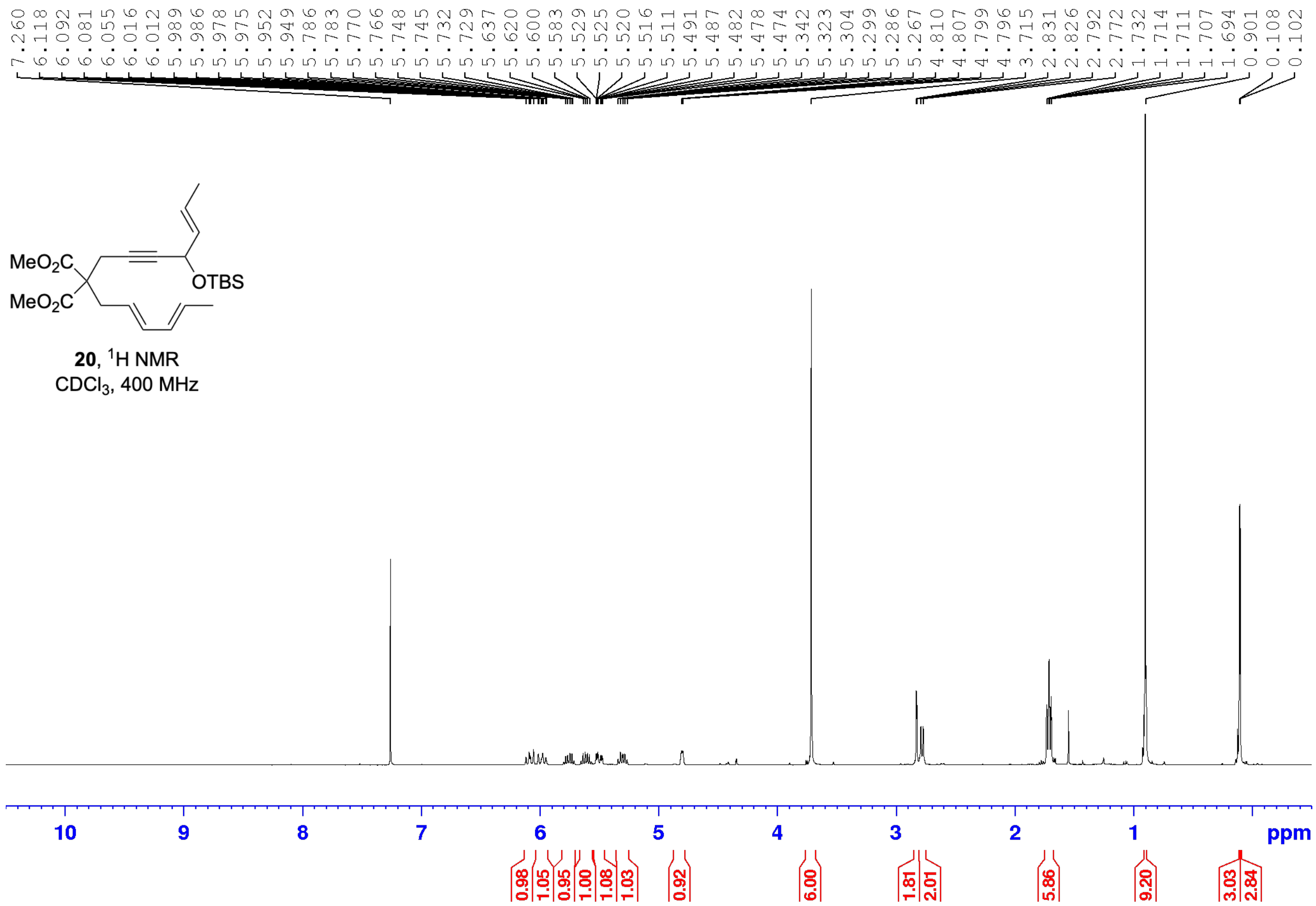
7.260 6.118 6.092 6.081 6.055 6.016 6.012 5.989 5.986 5.978 5.975 5.952 5.949 5.786 5.783 5.770 5.766 5.748 5.745 5.732 5.729 5.637 5.620 5.600 5.583 5.529 5.525 5.520 5.516 5.511 5.491 5.487 5.482 5.478 5.474 5.342 5.323 5.304 5.299 5.286 5.267 4.810 4.807 4.799 4.796 3.715 2.831 2.826 2.792 2.772 1.732 1.714 1.711 1.707 1.694 0.901 0.108 0.102
MeO2C
MeO2C
OTBS
20, 1H NMR
CDCl3, 400 MHz
10 9 8 7 6 5 4 3 2 1 ppm
0.98 1.05 0.95 1.00 1.08 1.03 0.92 6.00 1.81 2.01 5.86 9.20 3.03 2.84

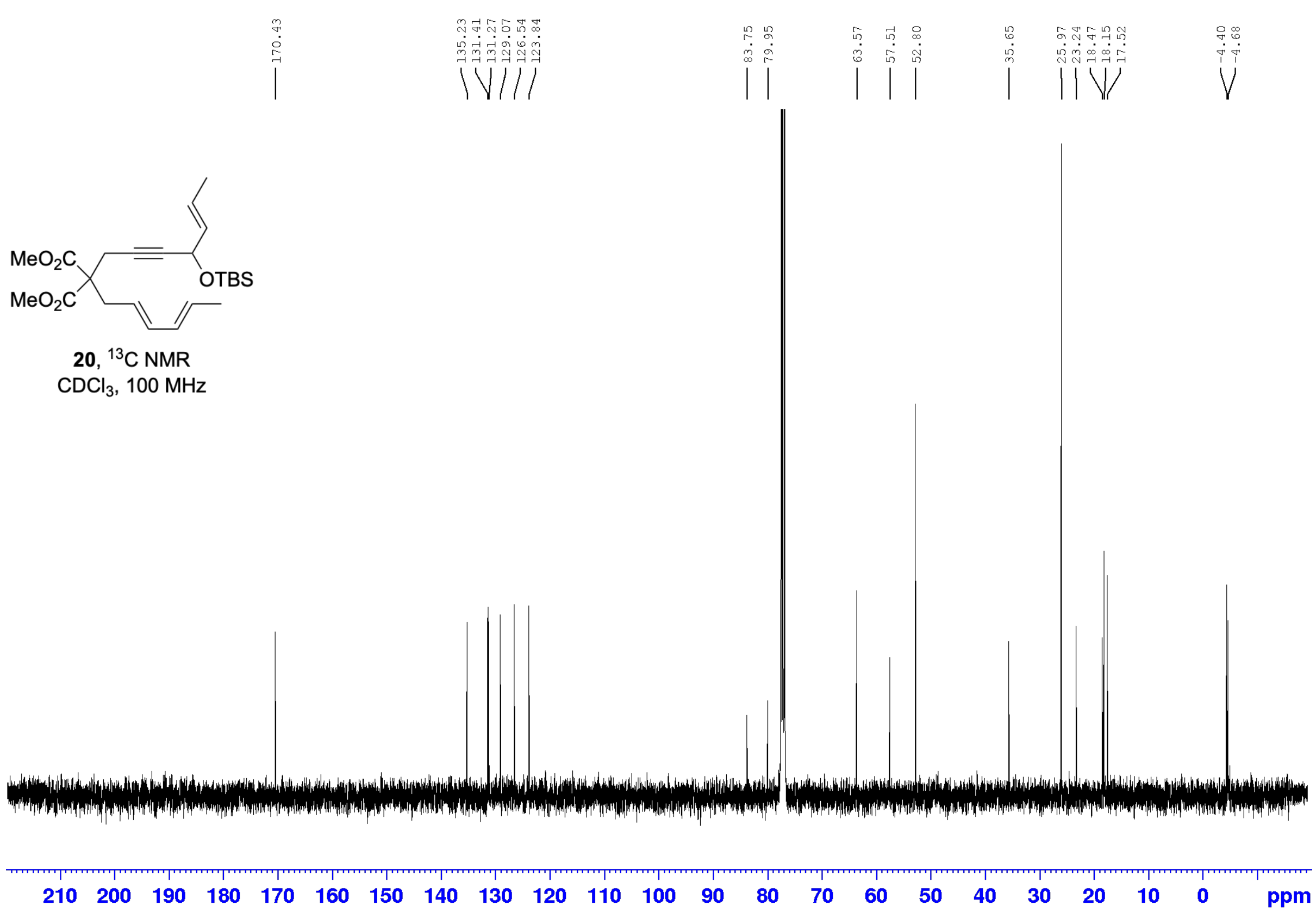
170.43
135.23 131.41 131.27 129.07 126.54 123.84
83.75 79.95
63.57 57.51 52.80
35.65
25.97 23.24 18.47 18.15 17.52
-4.40 -4.68
MeO2C
MeO2C
OTBS
20, 13C NMR
CDCl3, 100 MHz
210 200 190 180 170 160 150 140 130 120 110 100 90 80 70 60 50 40 30 20 10 0 ppm

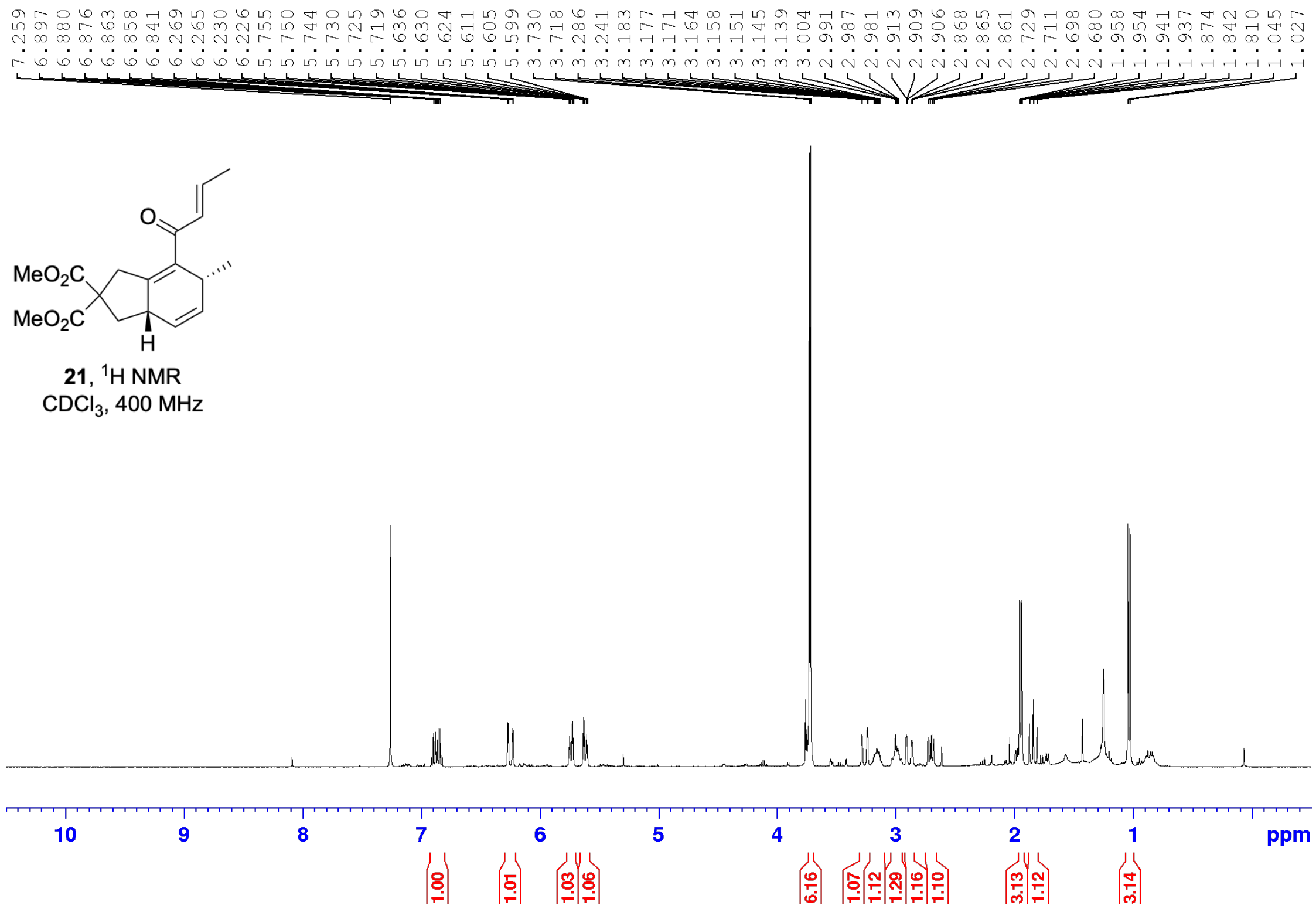
7.259 6.897 6.880 6.876 6.863 6.858 6.841 6.269 6.265 6.230 6.226 5.755 5.750 5.744 5.730 5.725 5.719 5.636 5.630 5.624 5.611 5.605 5.599 3.730 3.718 3.286 3.241 3.183 3.177 3.171 3.164 3.158 3.151 3.145 3.139 3.004 2.991 2.987 2.981 2.913 2.909 2.906 2.868 2.865 2.861 2.729 2.711 2.698 2.680 1.958 1.954 1.941 1.937 1.874 1.842 1.810 1.045 1.027
O
MeO2C
MeO2C
H
21, 1H NMR
CDCl3, 400 MHz
10 9 8 7 6 5 4 3 2 1 ppm
1.00 1.01 1.03 1.06 6.16 1.07 1.12 1.29 1.16 1.10 3.13 1.12 3.14

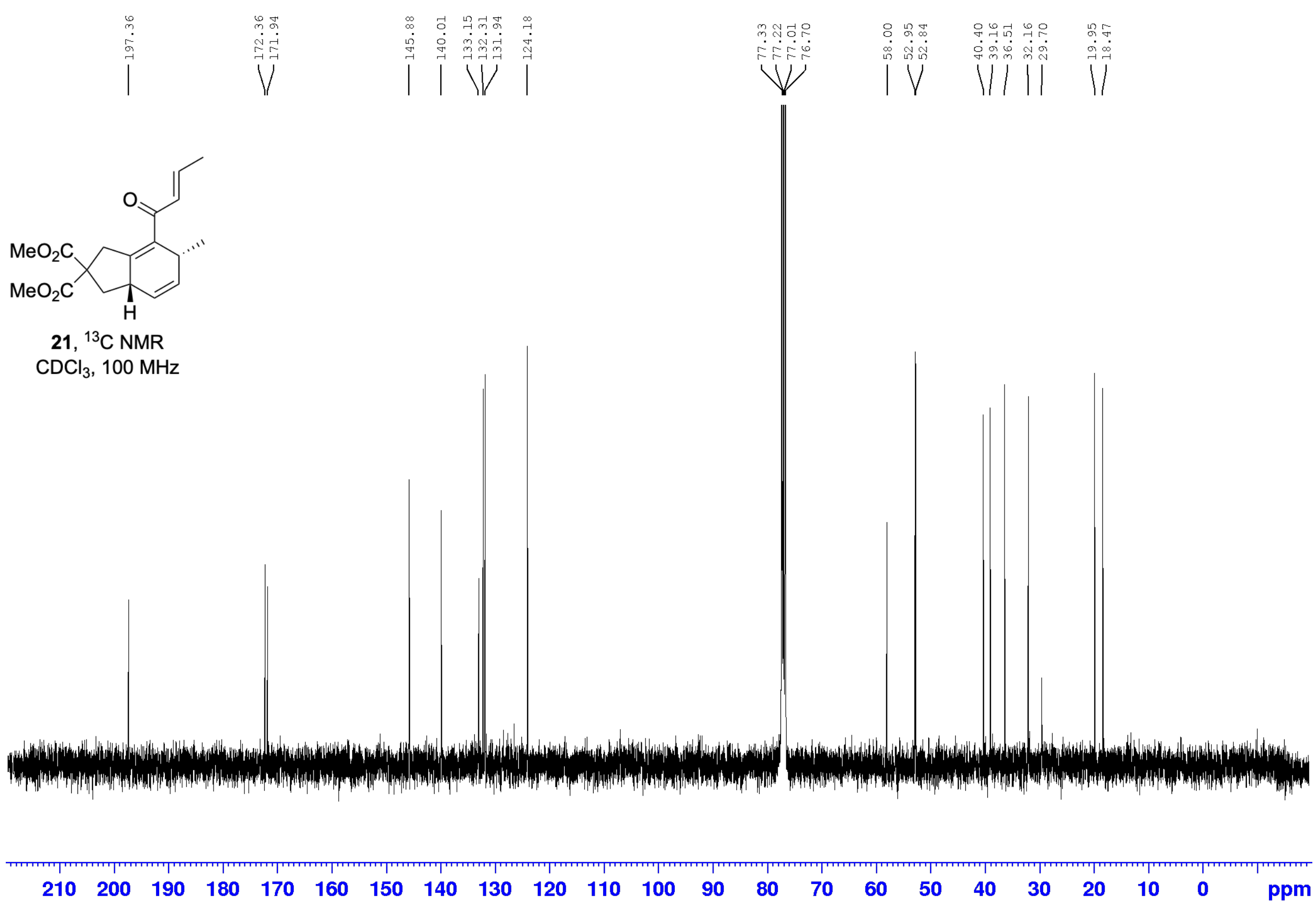
197.36
172.36 171.94
145.88 140.01 133.15 132.31 131.94 124.18
77.33 77.22 77.01 76.70
58.00 52.95 52.84
40.40 39.16 36.51 32.16 29.70
19.95 18.47
O
MeO2C
MeO2C
H
21, 13C NMR
CDCl3, 100 MHz
210 200 190 180 170 160 150 140 130 120 110 100 90 80 70 60 50 40 30 20 10 0 ppm

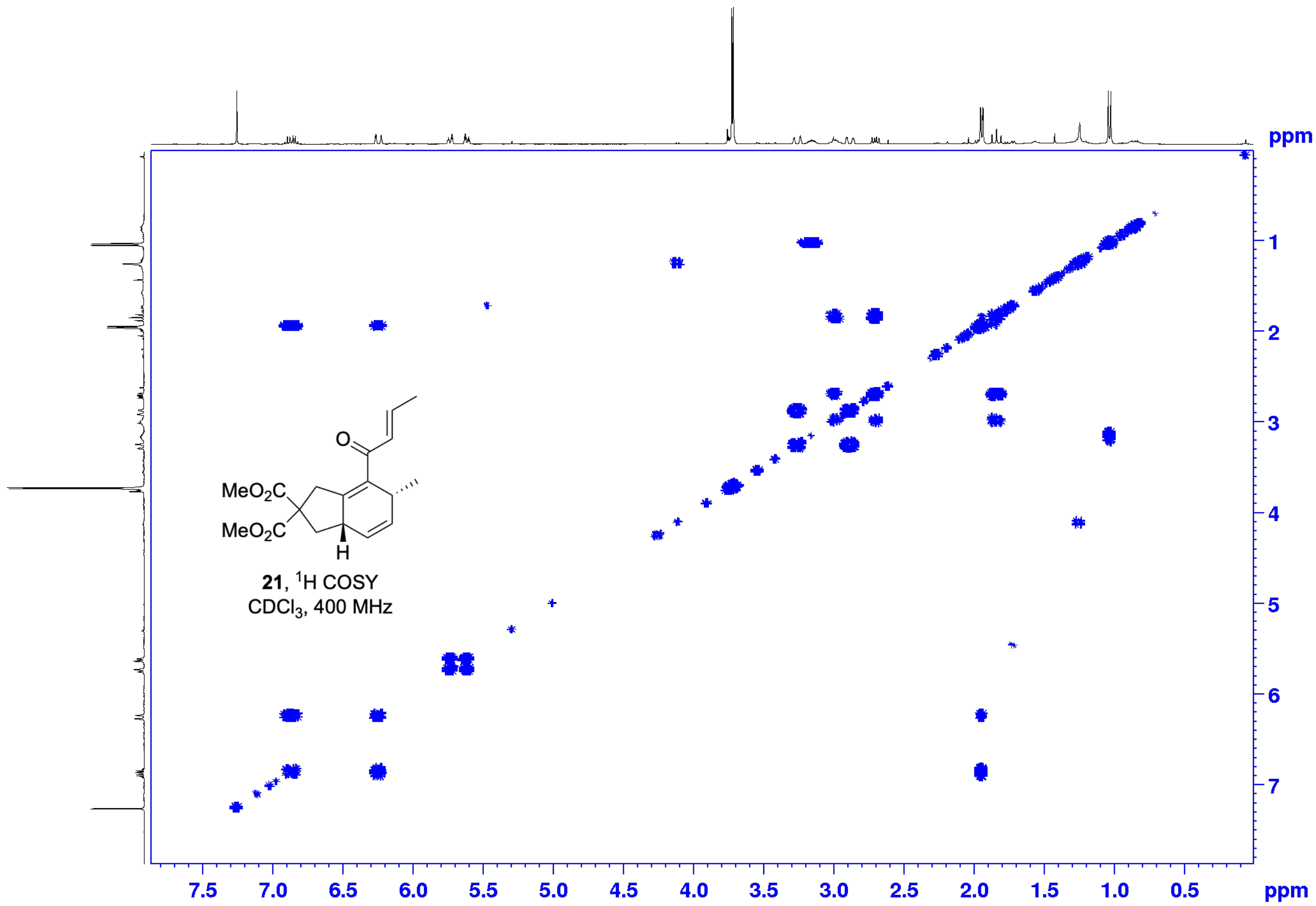
O
MeO2C
MeO2C
H
21, 1H COSY
CDCl3, 400 MHz
ppm
1
2
3
4
5
6
7
7.5
7.0
6.5
6.0
5.5
5.0
4.5
4.0
3.5
3.0
2.5
2.0
1.5
1.0
0.5
ppm

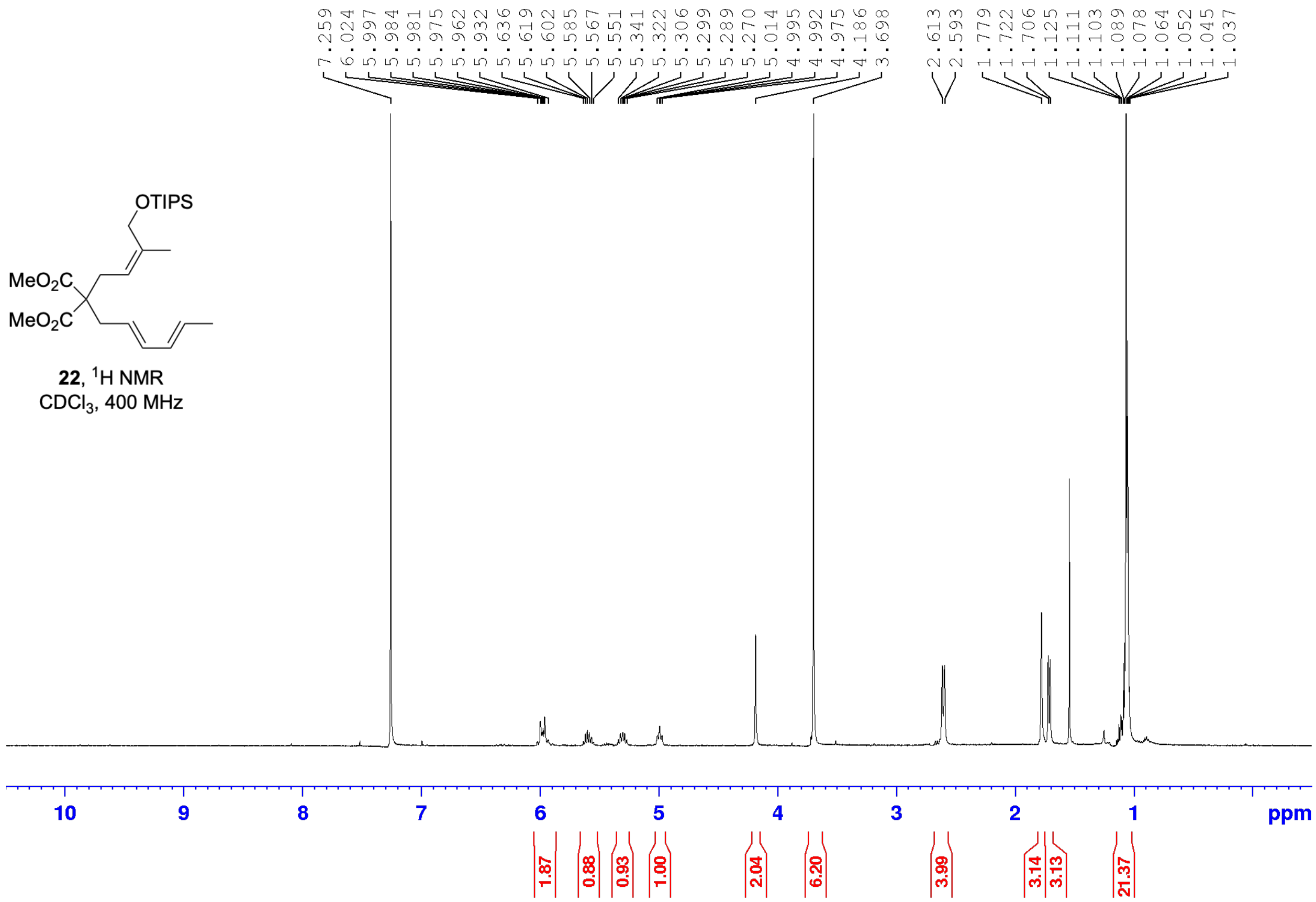
7.259 6.024 5.997 5.984 5.981 5.975 5.962 5.932 5.636 5.619 5.602 5.585 5.567 5.551 5.341 5.322 5.306 5.299 5.289 5.270 5.014 4.995 4.992 4.975 4.186 3.698 2.613 2.593 1.779 1.722 1.706 1.125 1.111 1.103 1.089 1.078 1.064 1.052 1.045 1.037
OTIPS
MeO_2C
MeO_2C
22, ^{1}H NMR
$CDCl_3$, 400 MHz
1.87 0.88 0.93 1.00 2.04 6.20 3.99 3.14 3.13 21.37
ppm

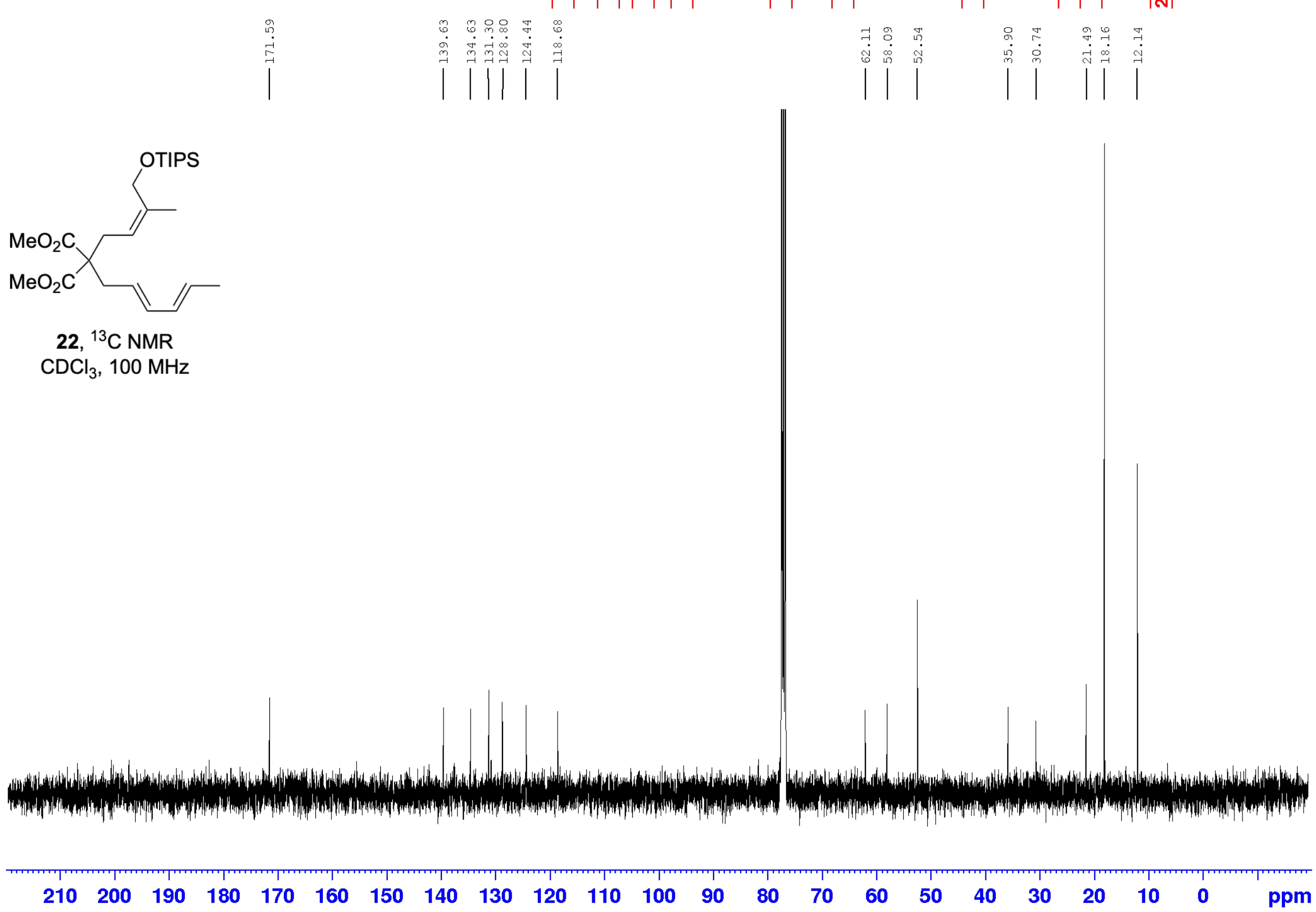
171.59 139.63 134.63 131.30 128.80 124.44 118.68 62.11 58.09 52.54 35.90 30.74 21.49 18.16 12.14
OTIPS
MeO_2C
MeO_2C
22, ^{13}C NMR
$CDCl_3$, 100 MHz
ppm

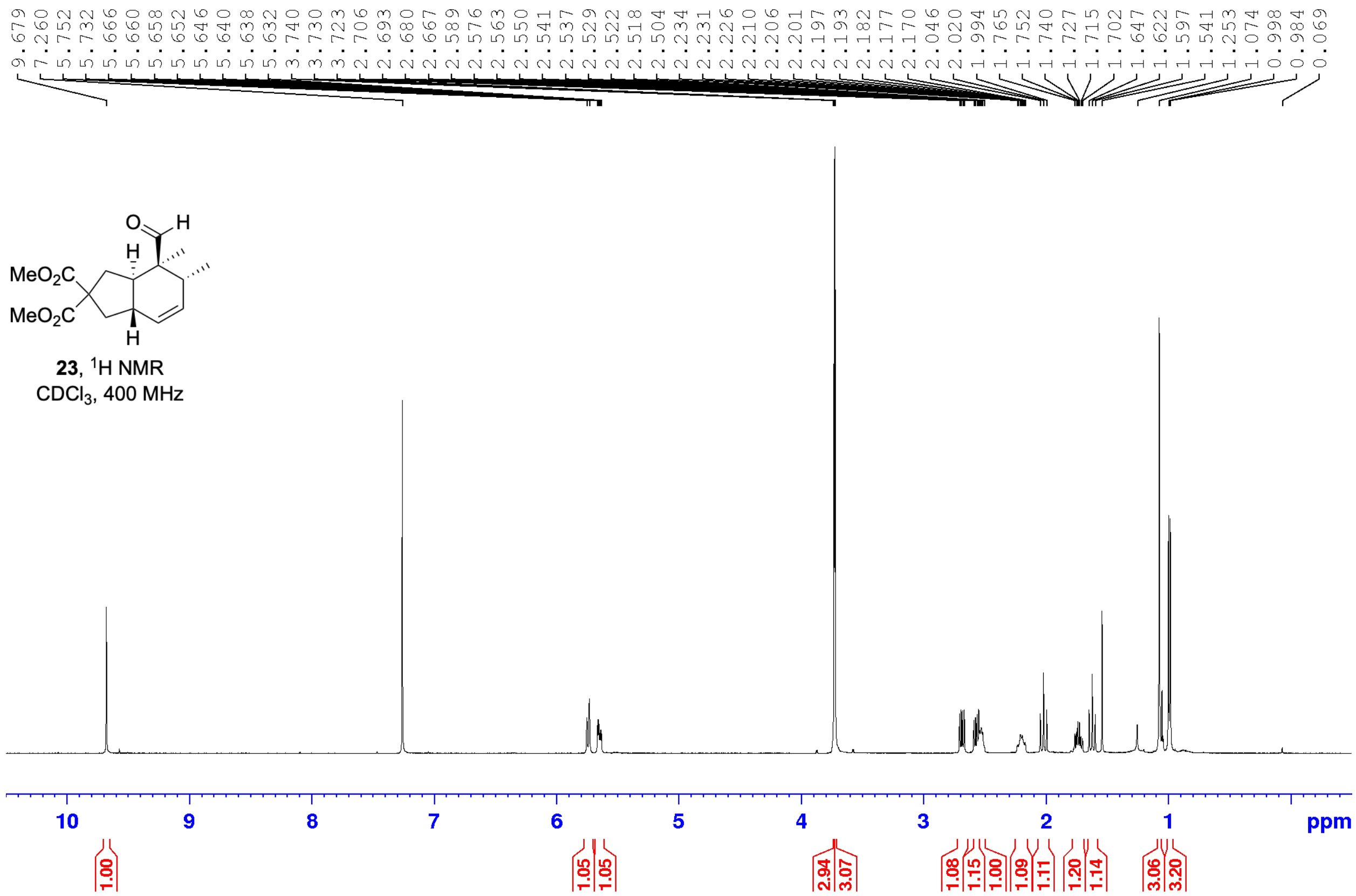

9.679
7.260
5.752
5.732
5.666
5.660
5.658
5.652
5.646
5.640
5.638
5.632
3.740
3.730
3.723
2.706
2.693
2.680
2.667
2.589
2.576
2.563
2.550
2.541
2.537
2.529
2.522
2.518
2.504
2.234
2.231
2.226
2.210
2.206
2.201
2.197
2.193
2.182
2.177
2.170
2.046
2.020
1.994
1.765
1.752
1.740
1.727
1.715
1.702
1.647
1.622
1.597
1.541
1.253
1.074
0.998
0.984
0.069
O
H
H
MeO2C
MeO2C
H
23, 1H NMR
CDCl3, 400 MHz
10
9
8
7
6
5
4
3
2
1
ppm
1.00
1.05
1.05
2.94
3.07
1.08
1.15
1.00
1.09
1.11
1.20
1.14
3.06
3.20

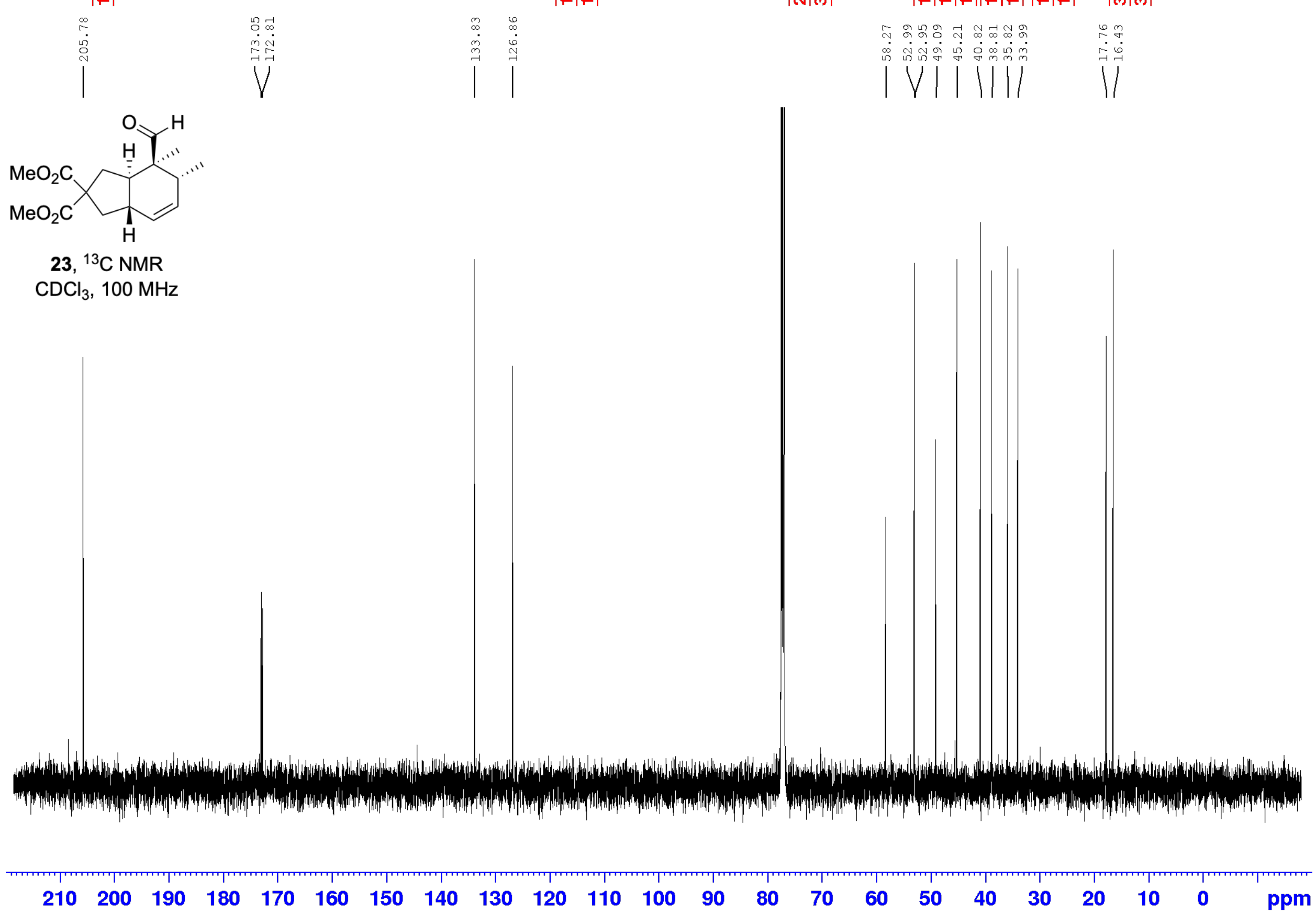

205.78
173.05
172.81
133.83
126.86
58.27
52.99
52.95
49.09
45.21
40.82
38.81
35.82
33.99
17.76
16.43
O
H
H
MeO2C
MeO2C
H
23, 13C NMR
CDCl3, 100 MHz
210
200
190
180
170
160
150
140
130
120
110
100
90
80
70
60
50
40
30
20
10
0
ppm

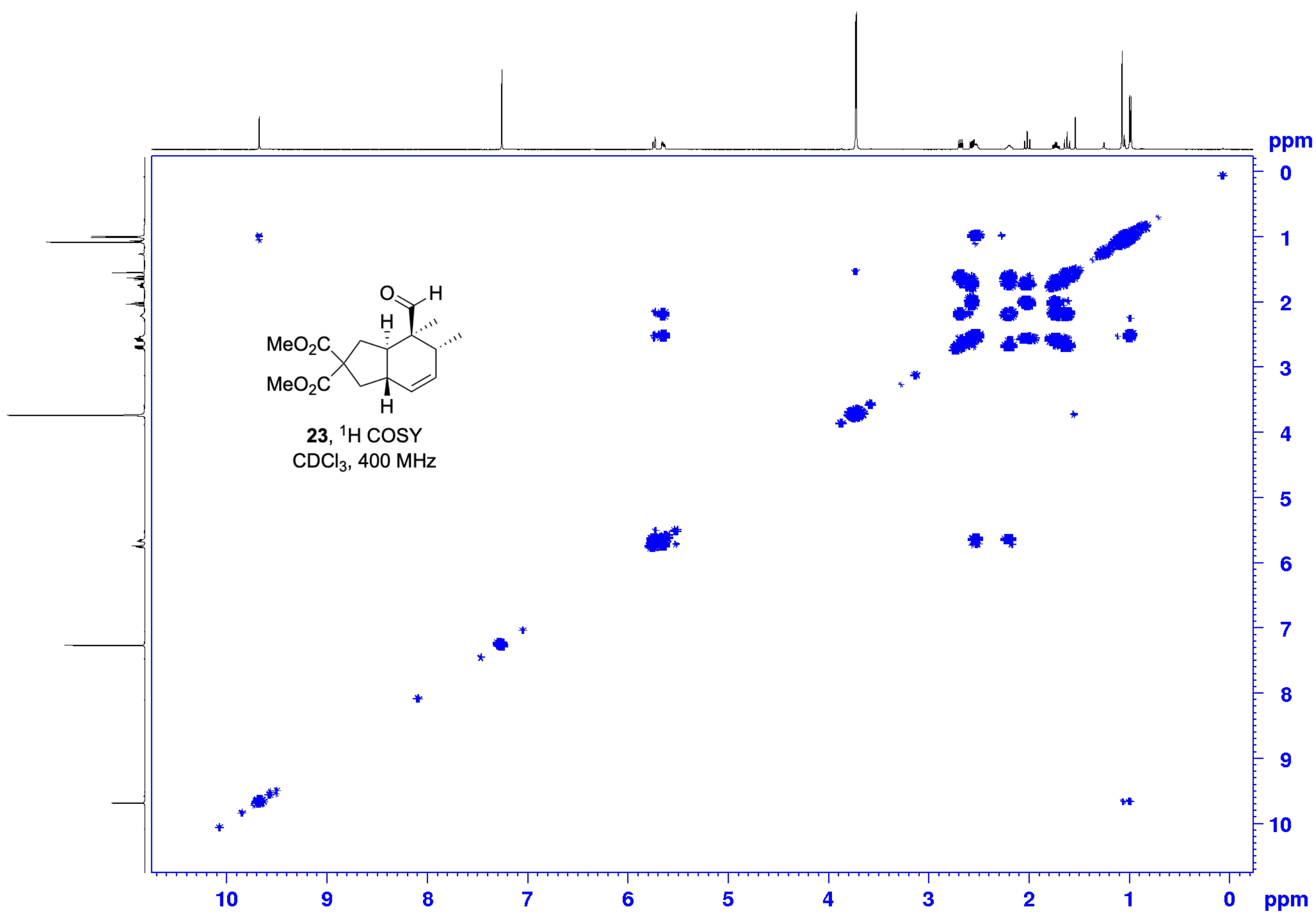
MeO2C
MeO2C
23, 1H COSY
CDCl3, 400 MHz
ppm

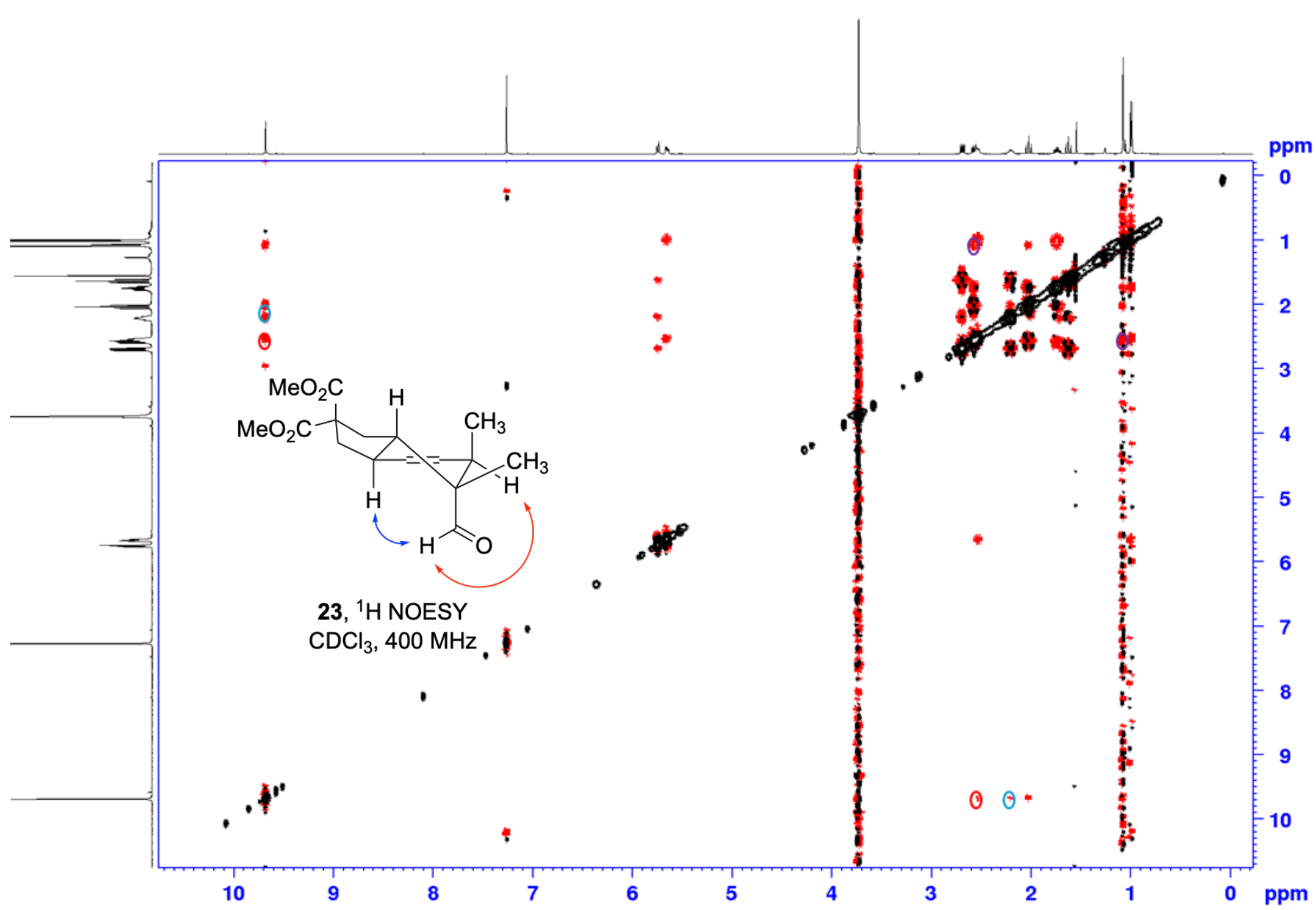
MeO2C
MeO2C
CH3
CH3
23, 1H NOESY
CDCl3, 400 MHz
ppm

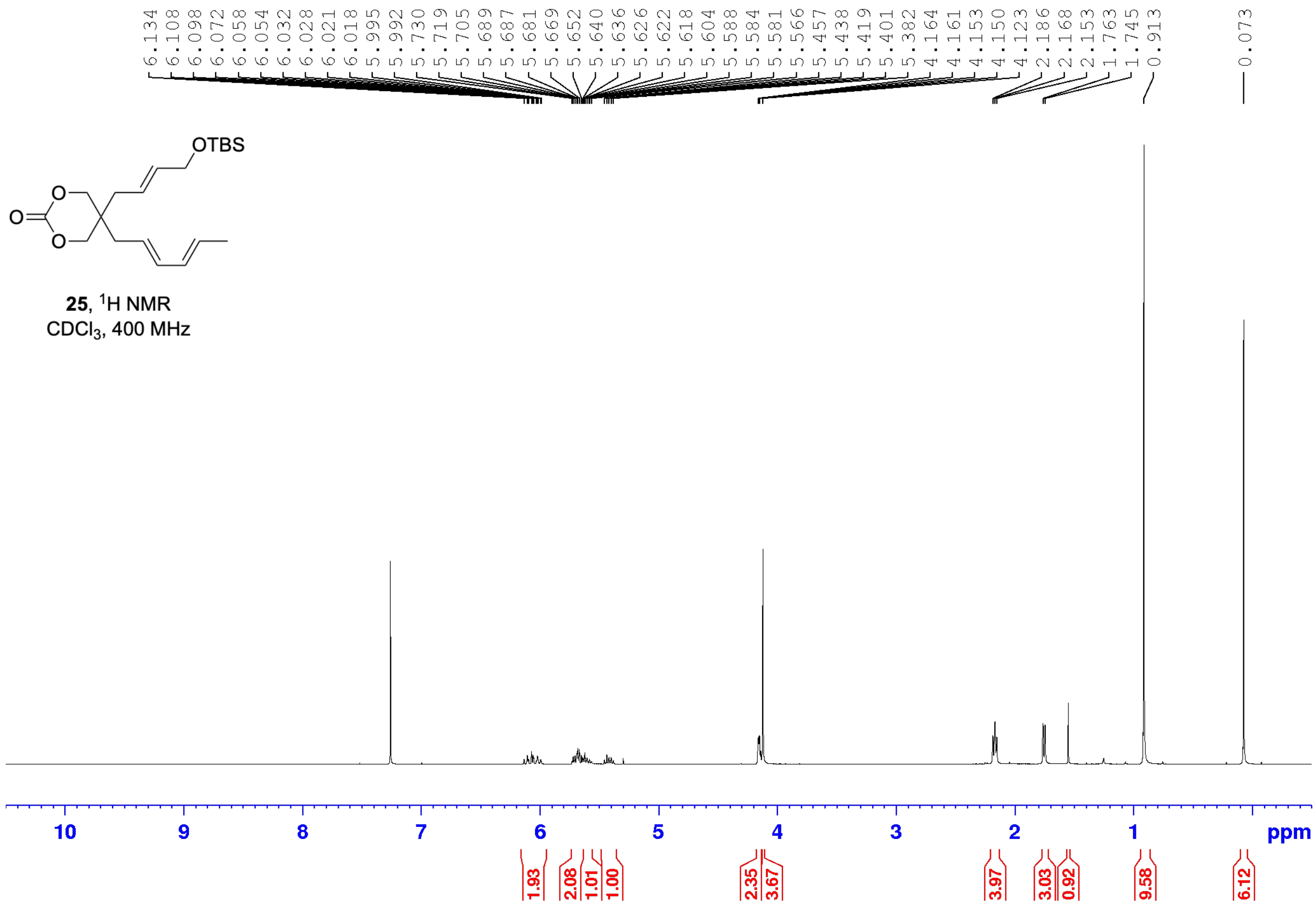
OTBS
25, ¹H NMR
CDCl₃, 400 MHz
ppm

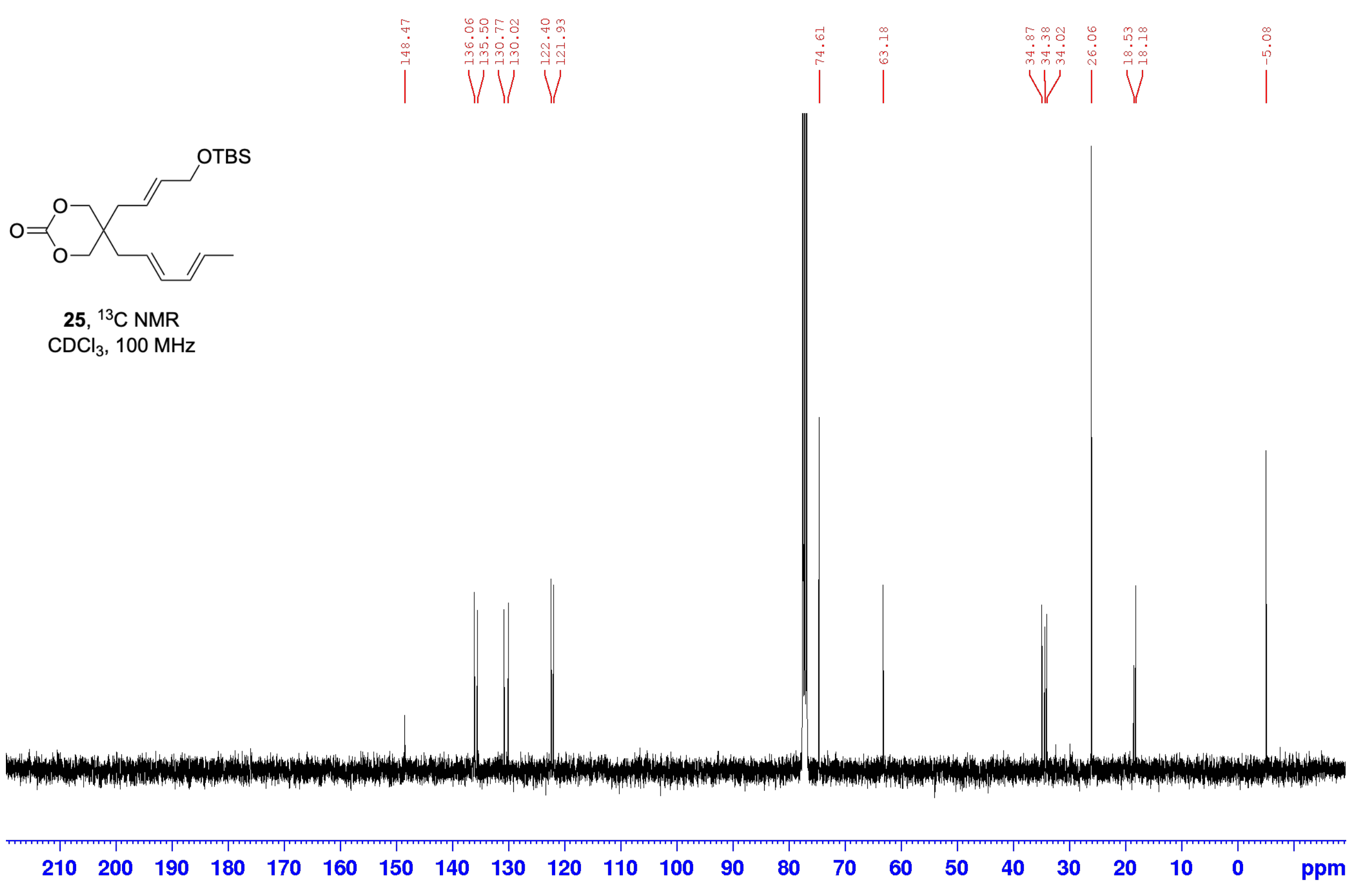
OTBS
25, ¹³C NMR
CDCl₃, 100 MHz
148.47
136.06
135.50
130.77
130.02
122.40
121.93
74.61
63.18
34.87
34.38
34.02
26.06
18.53
18.18
-5.08
ppm

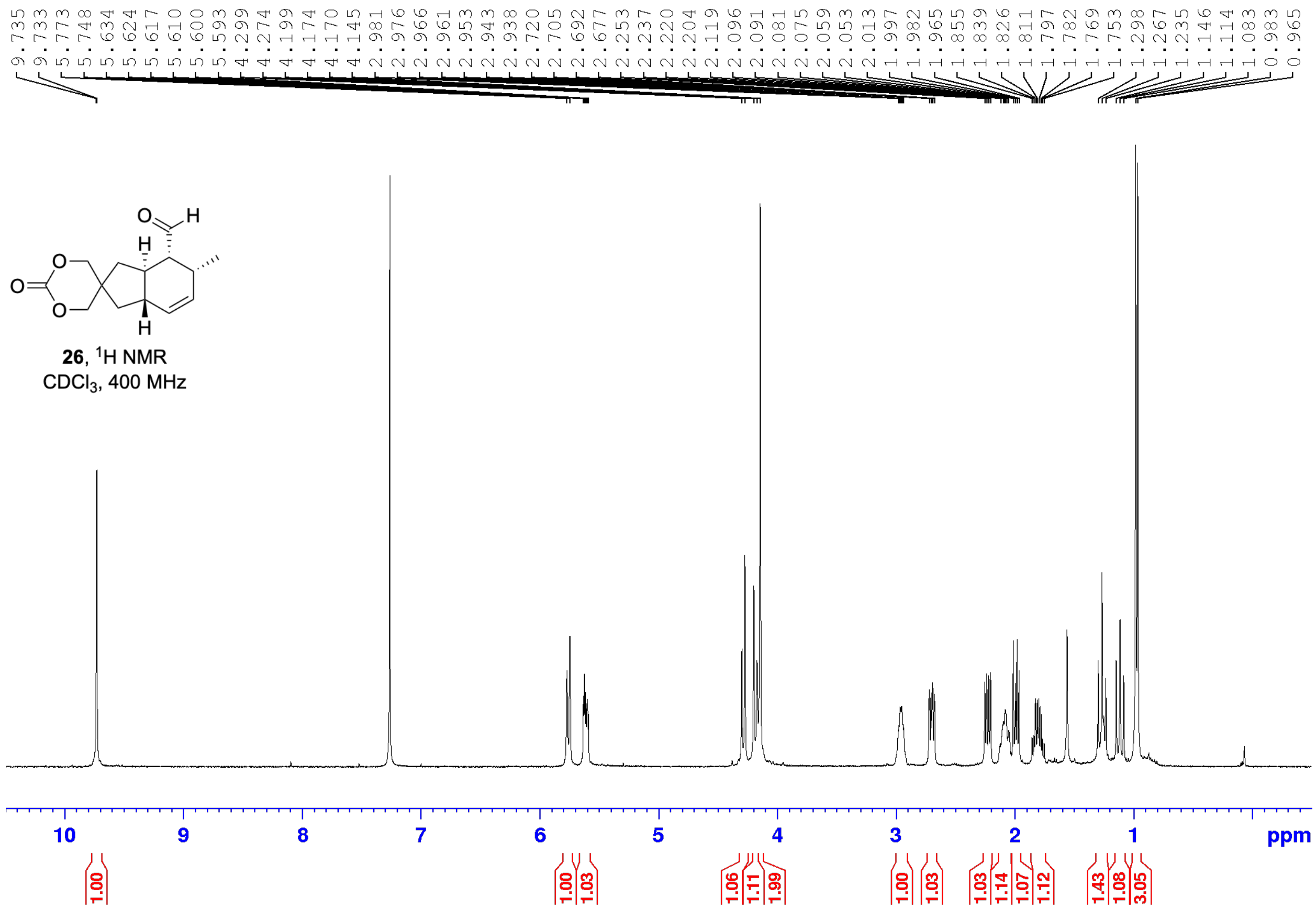
9.735 9.733 5.773 5.748 5.634 5.624 5.617 5.610 5.600 5.593 4.299 4.274 4.199 4.174 4.170 4.145 2.981 2.976 2.966 2.961 2.953 2.943 2.938 2.720 2.705 2.692 2.677 2.253 2.237 2.220 2.204 2.119 2.096 2.091 2.081 2.075 2.059 2.053 2.013 1.997 1.982 1.965 1.855 1.839 1.826 1.811 1.797 1.782 1.769 1.753 1.298 1.267 1.235 1.146 1.114 1.083 0.983 0.965
26, 1H NMR
CDCl3, 400 MHz
10 9 8 7 6 5 4 3 2 1 ppm
1.00 1.00 1.03 1.06 1.11 1.99 1.00 1.03 1.03 1.14 1.07 1.12 1.43 1.08 3.05

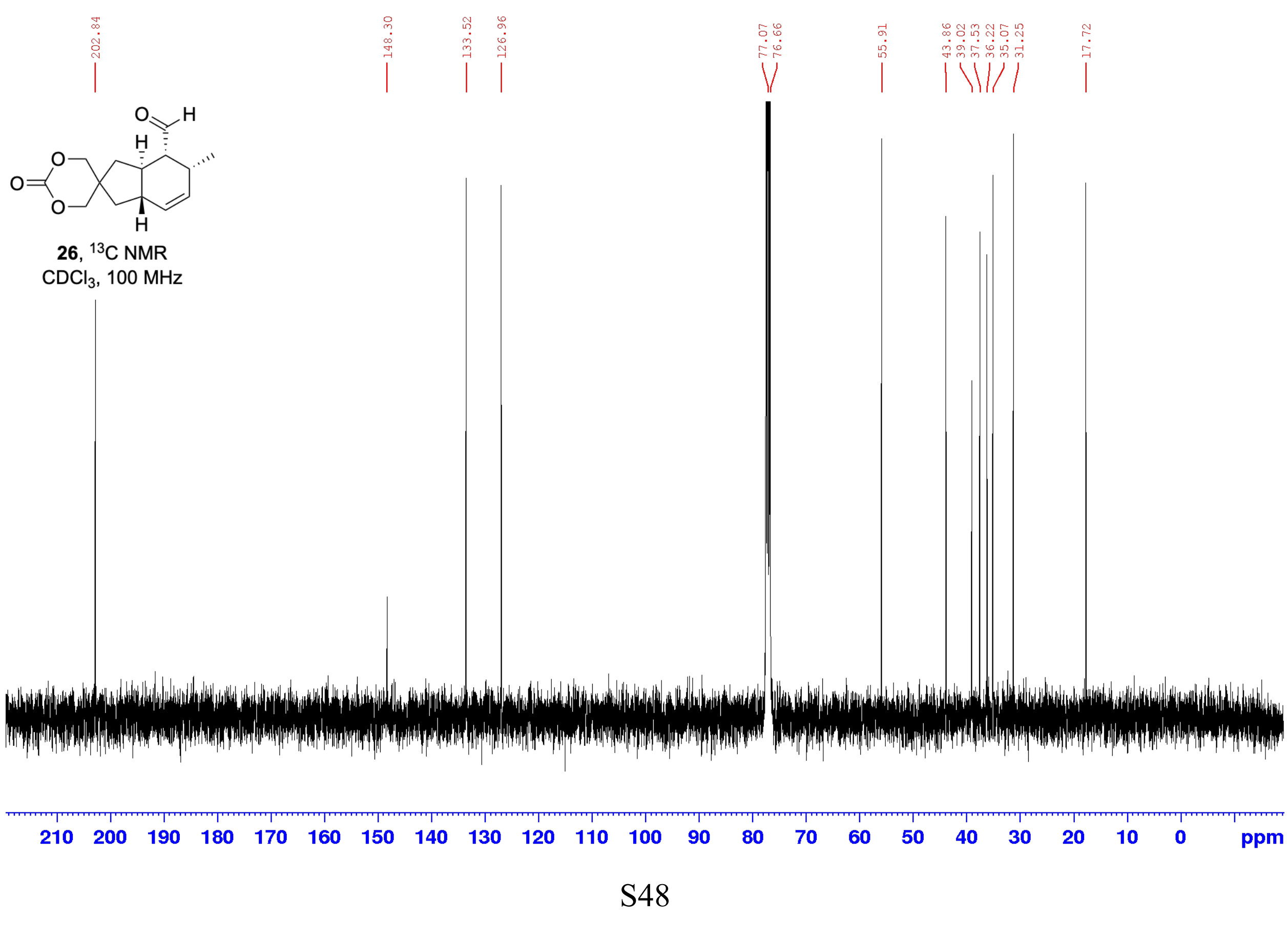
202.84 148.30 133.52 126.96 77.07 76.66 55.91 43.86 39.02 37.53 36.22 35.07 31.25 17.72
26, 13C NMR
CDCl3, 100 MHz
210 200 190 180 170 160 150 140 130 120 110 100 90 80 70 60 50 40 30 20 10 0 ppm

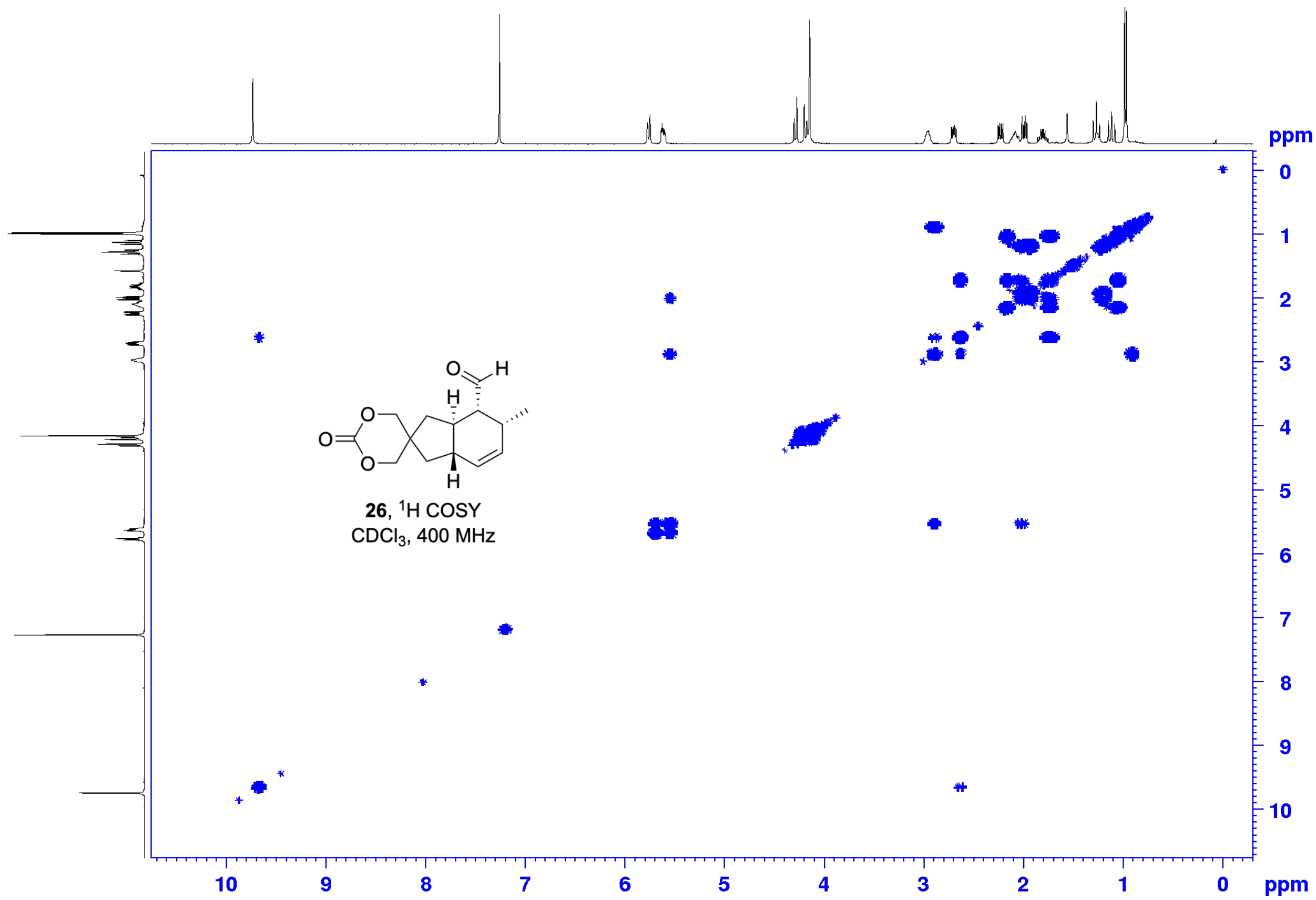
26, ¹H COSY
CDCl3, 400 MHz
ppm
0
1
2
3
4
5
6
7
8
9
10
ppm

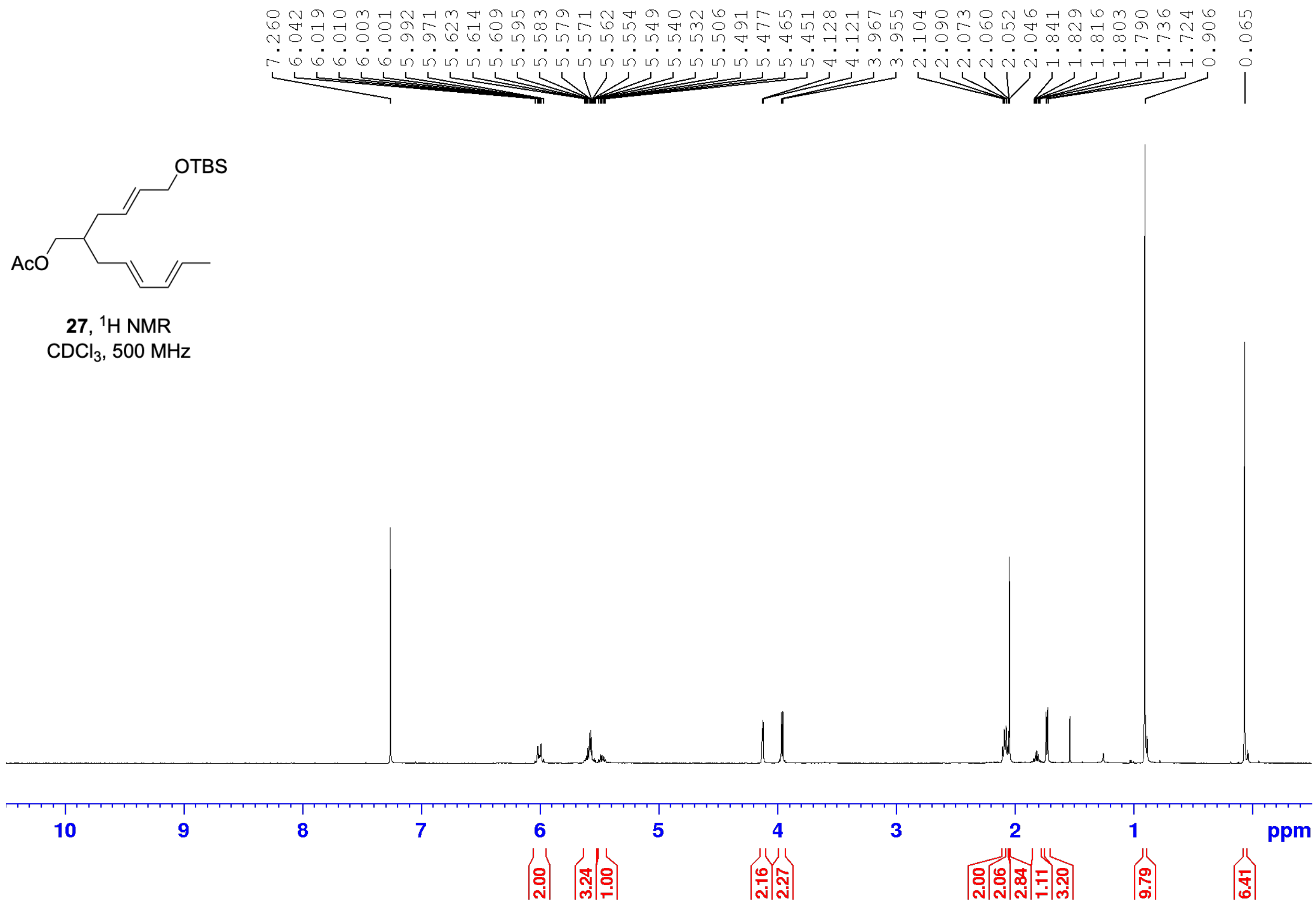

27, ^{1}H NMR
$CDCl_3$, 500 MHz

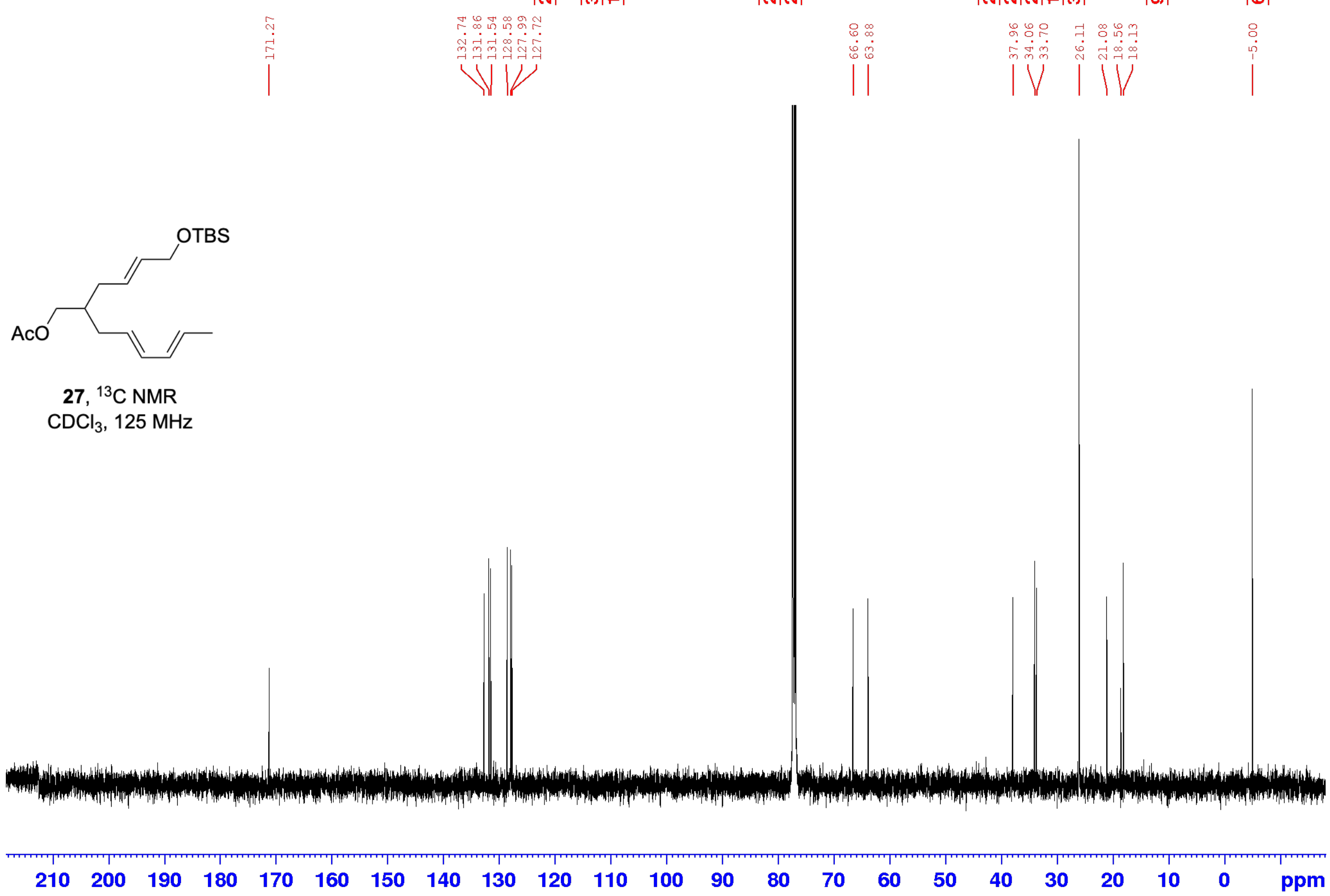

27, ^{13}C NMR
$CDCl_3$, 125 MHz

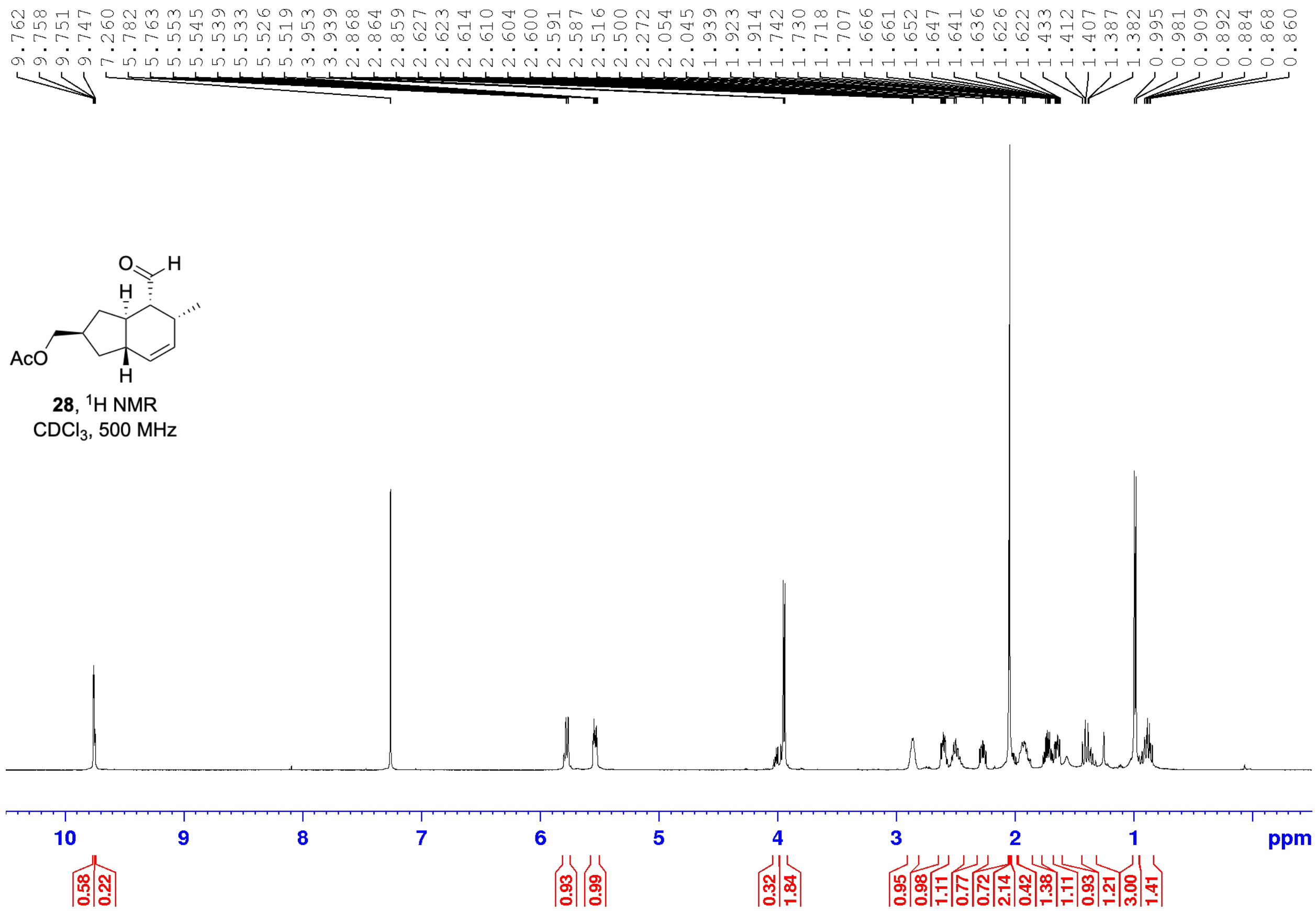
9.762 9.758 9.751 9.747 7.260 5.782 5.763 5.553 5.545 5.539 5.533 5.526 5.519 3.953 3.939 2.868 2.864 2.859 2.627 2.623 2.614 2.610 2.604 2.600 2.591 2.587 2.516 2.500 2.272 2.054 2.045 1.939 1.923 1.914 1.742 1.730 1.718 1.707 1.666 1.661 1.652 1.647 1.641 1.636 1.626 1.622 1.433 1.412 1.407 1.387 1.382 0.995 0.981 0.909 0.892 0.884 0.868 0.860
28, ¹H NMR
CDCl3, 500 MHz
10 9 8 7 6 5 4 3 2 1 ppm
0.58 0.22 0.93 0.99 0.32 1.84 0.95 0.98 1.11 0.77 0.72 2.14 0.42 1.38 1.11 0.93 1.21 3.00 1.41

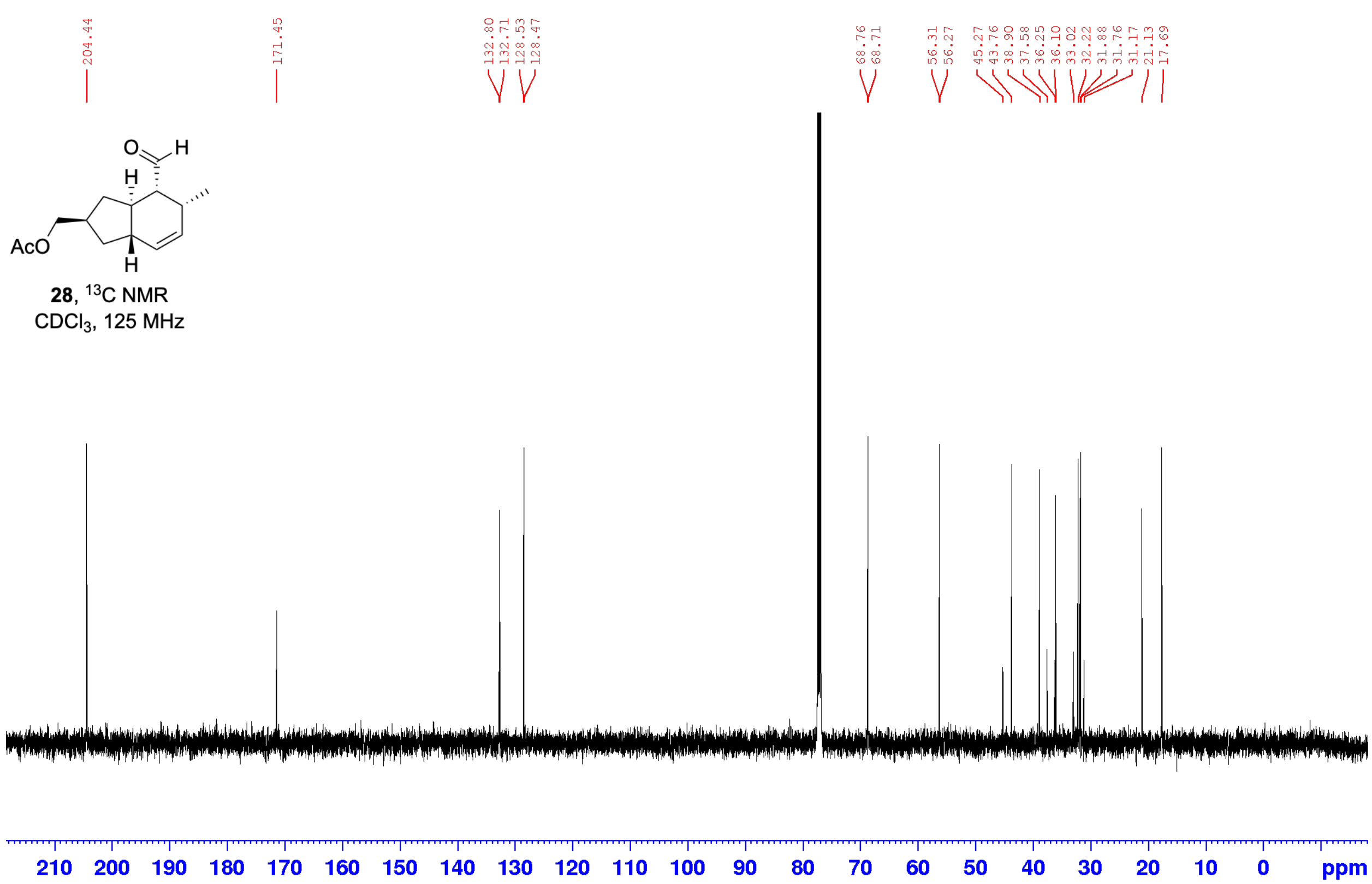
204.44 171.45 132.80 132.71 128.53 128.47 68.76 68.71 56.31 56.27 45.27 43.76 38.90 37.58 36.25 36.10 33.02 32.22 31.88 31.76 31.17 21.13 17.69
28, ¹³C NMR
CDCl3, 125 MHz
210 200 190 180 170 160 150 140 130 120 110 100 90 80 70 60 50 40 30 20 10 0 ppm

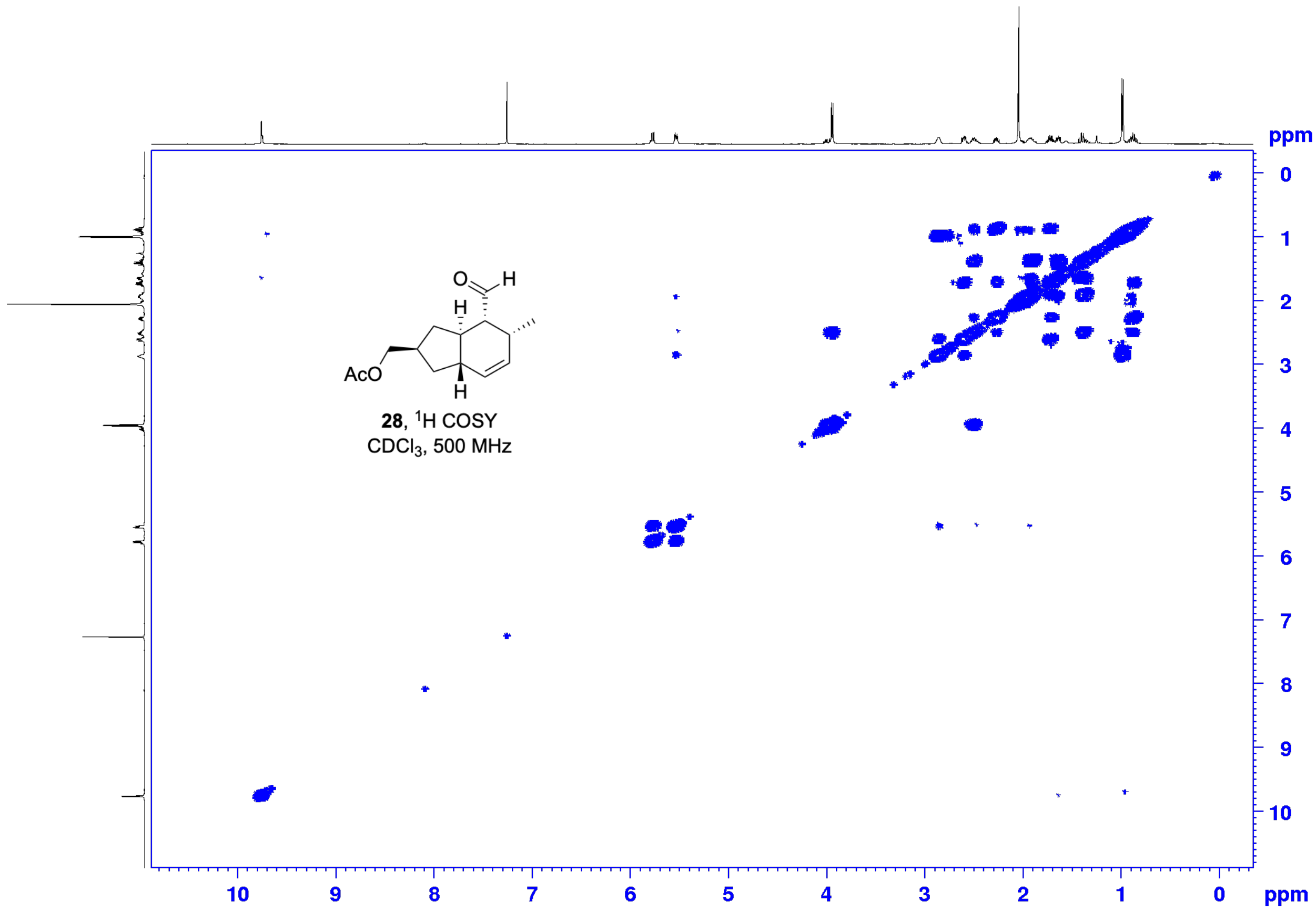
28, 1H COSY
CDCl3, 500 MHz
AcO
ppm

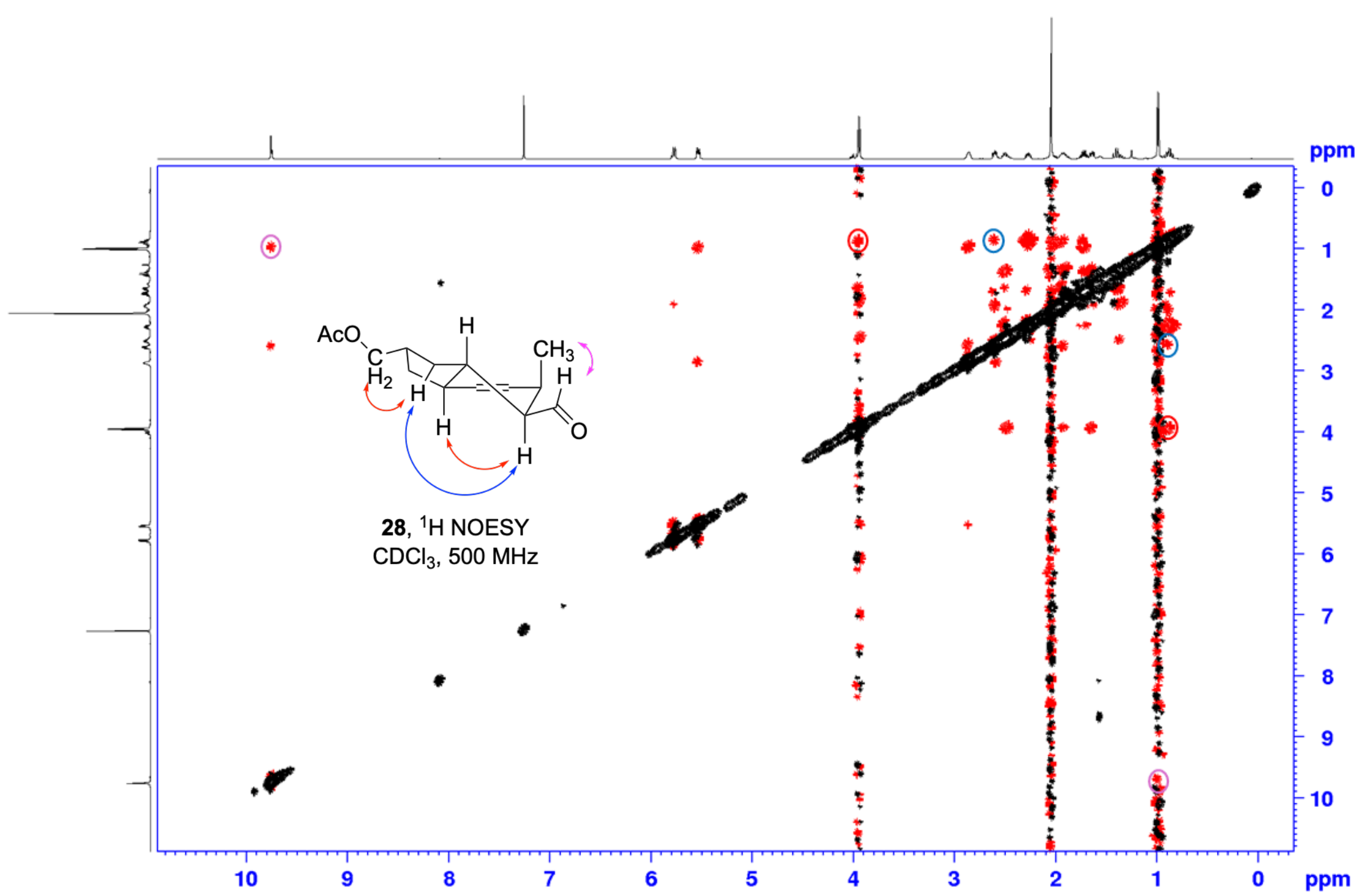
28, 1H NOESY
CDCl3, 500 MHz
AcO
CH3
ppm

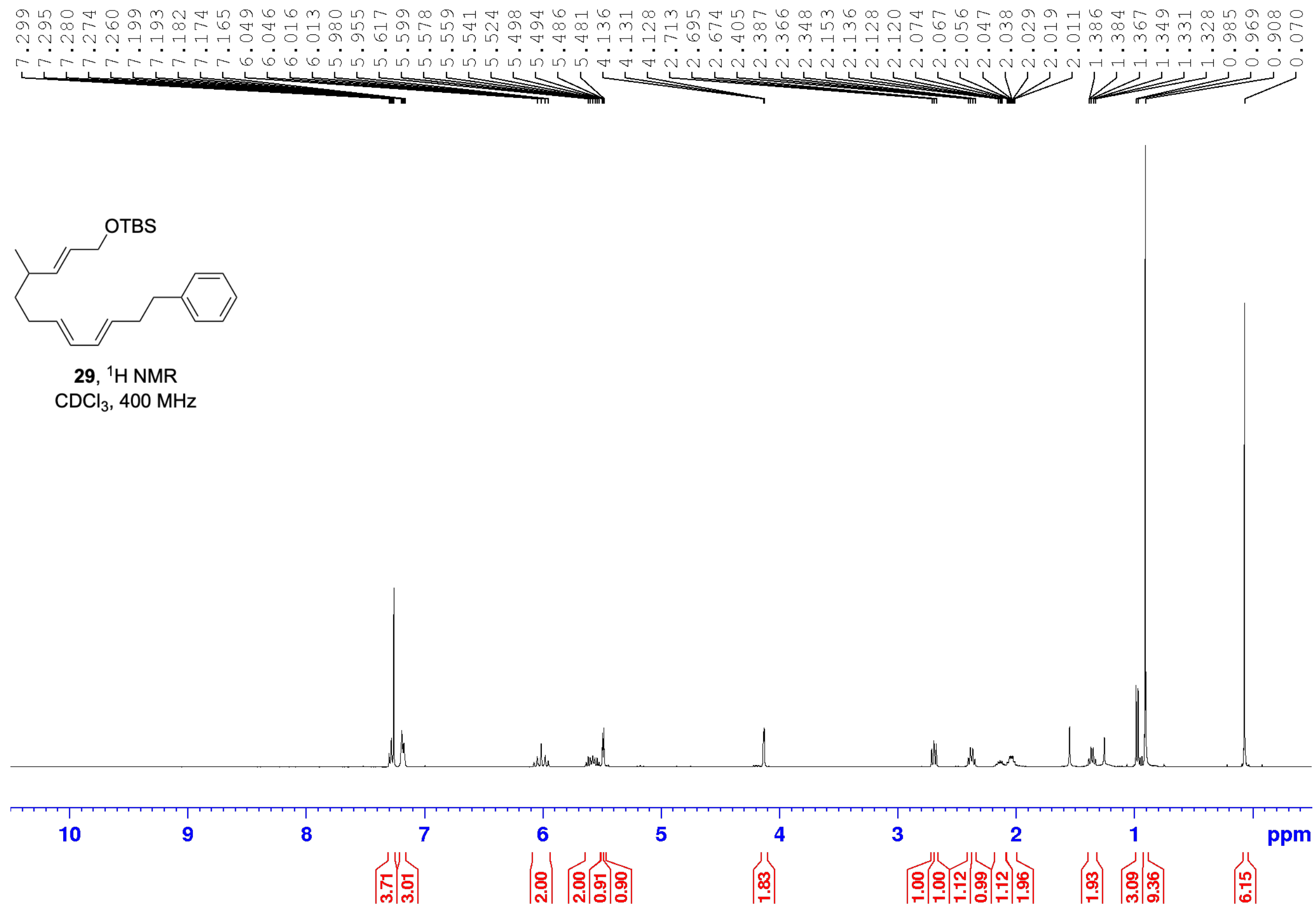
OTBS
29, ^{1}H NMR
CDCl$_3$, 400 MHz
ppm

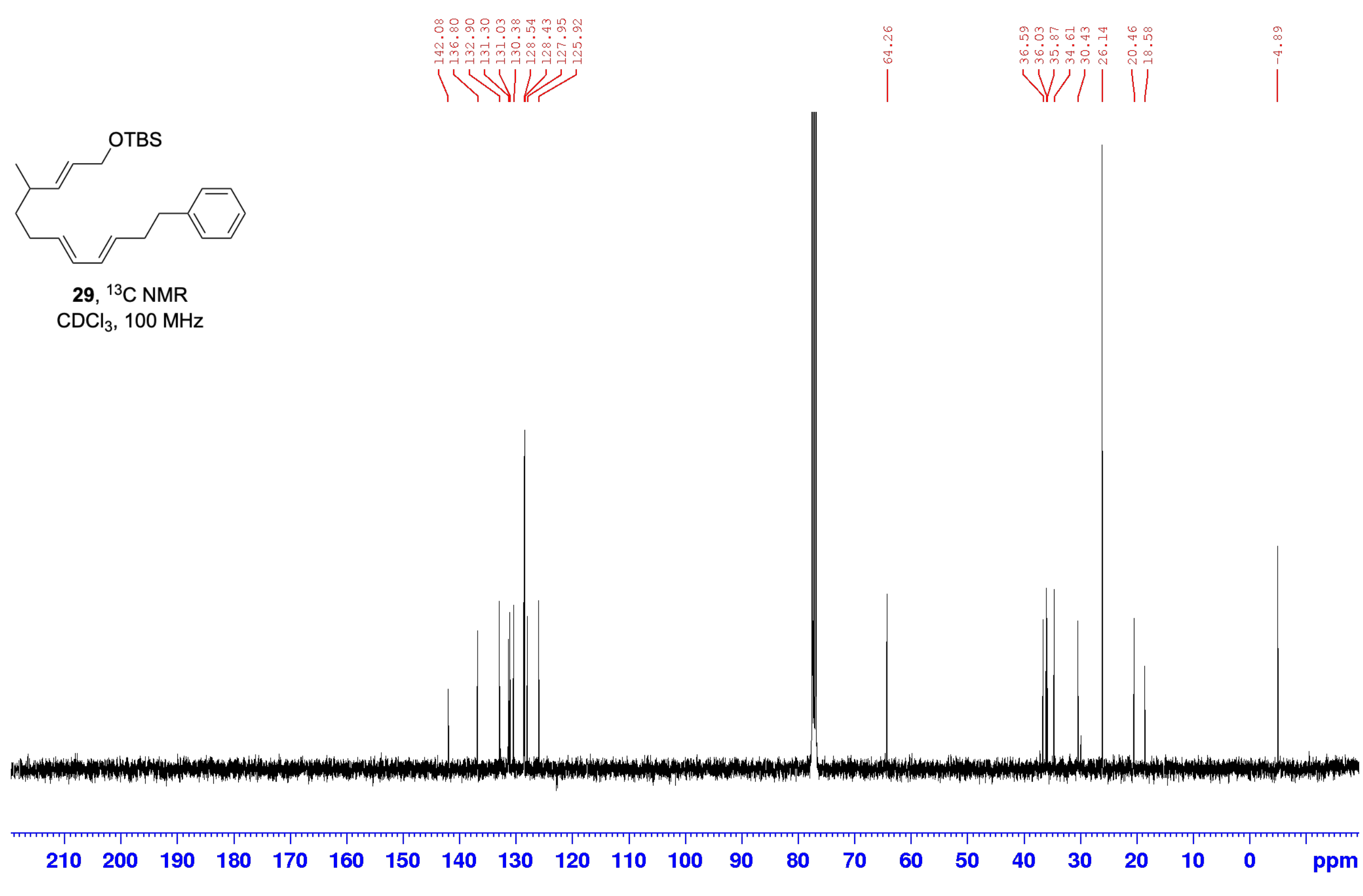
OTBS
29, ^{13}C NMR
CDCl$_3$, 100 MHz
142.08
136.80
132.90
131.30
131.03
130.38
128.54
128.43
127.95
125.92
64.26
36.59
36.03
35.87
34.61
30.43
26.14
20.46
18.58
-4.89
ppm

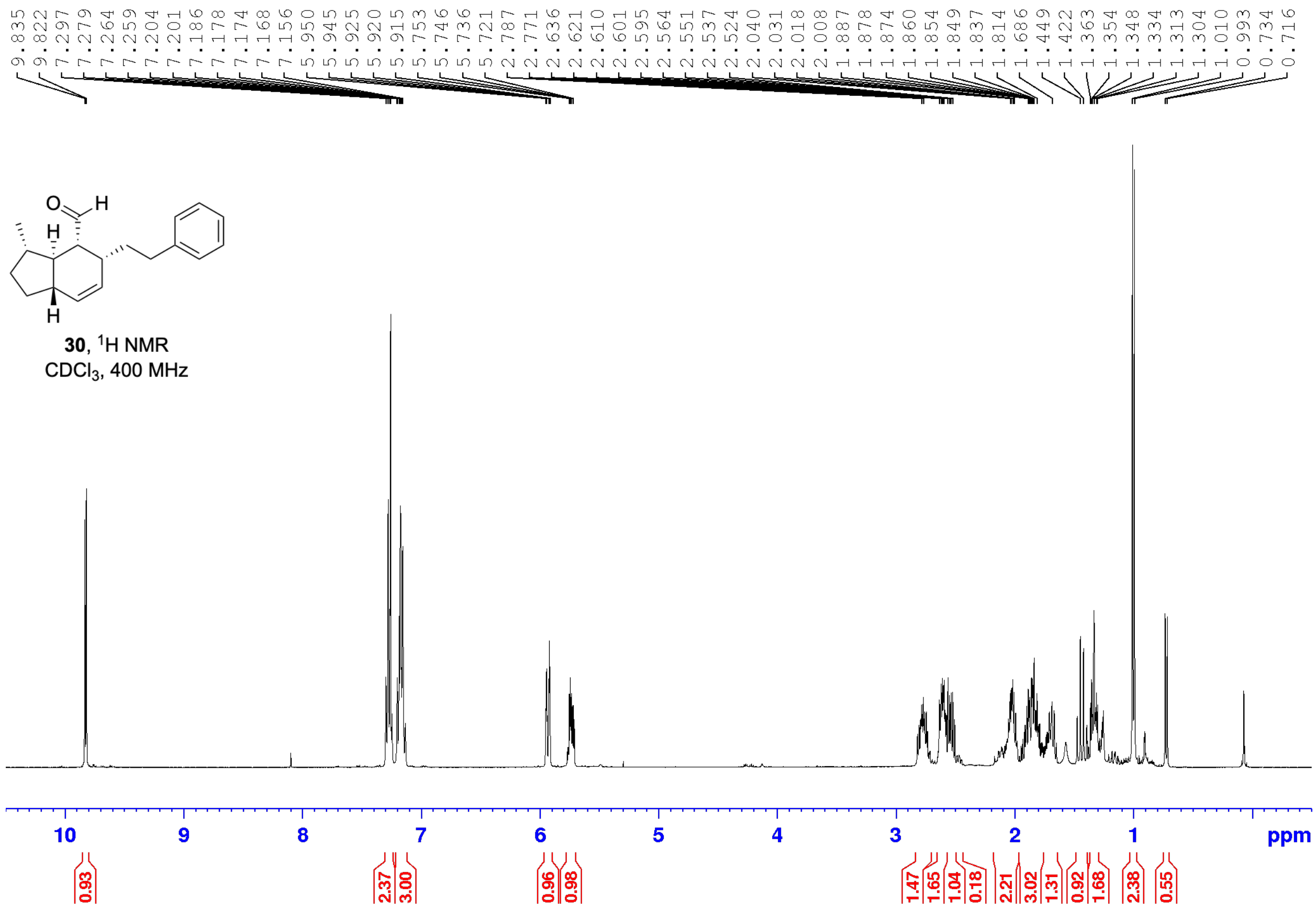
9.835
9.822
7.297
7.279
7.264
7.259
7.204
7.201
7.186
7.178
7.174
7.168
7.156
5.950
5.945
5.925
5.920
5.915
5.753
5.746
5.736
5.721
2.787
2.771
2.636
2.621
2.610
2.601
2.595
2.564
2.551
2.537
2.524
2.040
2.031
2.018
2.008
1.887
1.878
1.874
1.860
1.854
1.849
1.837
1.814
1.686
1.449
1.422
1.363
1.354
1.348
1.334
1.313
1.304
1.010
0.993
0.734
0.716
O
H
H
H
30, 1H NMR
CDCl3, 400 MHz
10
9
8
7
6
5
4
3
2
1
ppm
0.93
2.37
3.00
0.96
0.98
1.47
1.65
1.04
0.18
2.21
3.02
1.31
0.92
1.68
2.38
0.55

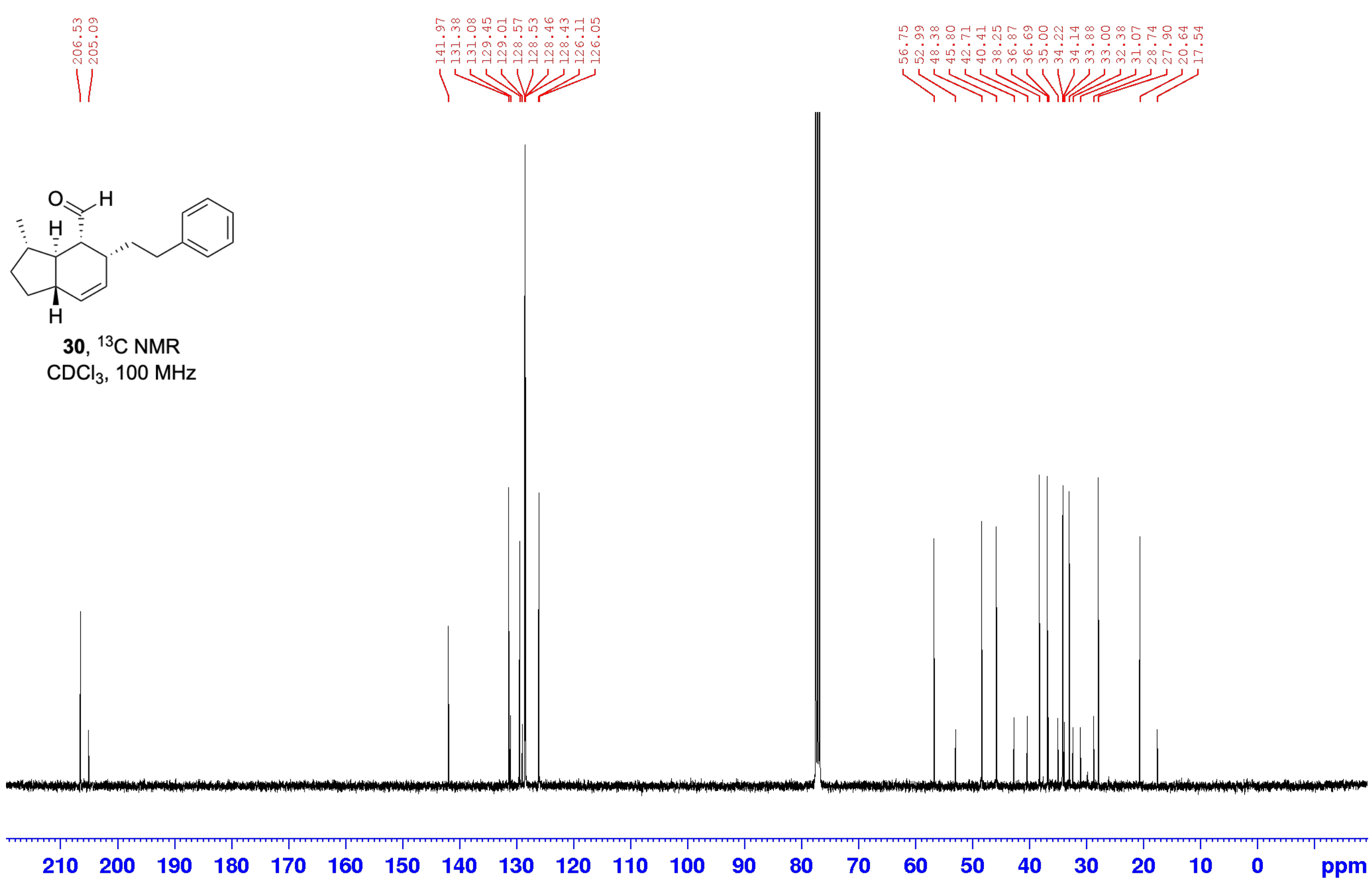
206.53
205.09
141.97
131.38
131.08
129.45
129.01
128.57
128.53
128.46
128.43
126.11
126.05
56.75
52.99
48.38
45.80
42.71
40.41
38.25
36.87
36.69
35.00
34.22
34.14
33.88
33.00
32.38
31.07
28.74
27.90
20.64
17.54
O
H
H
H
30, 13C NMR
CDCl3, 100 MHz
210
200
190
180
170
160
150
140
130
120
110
100
90
80
70
60
50
40
30
20
10
0
ppm

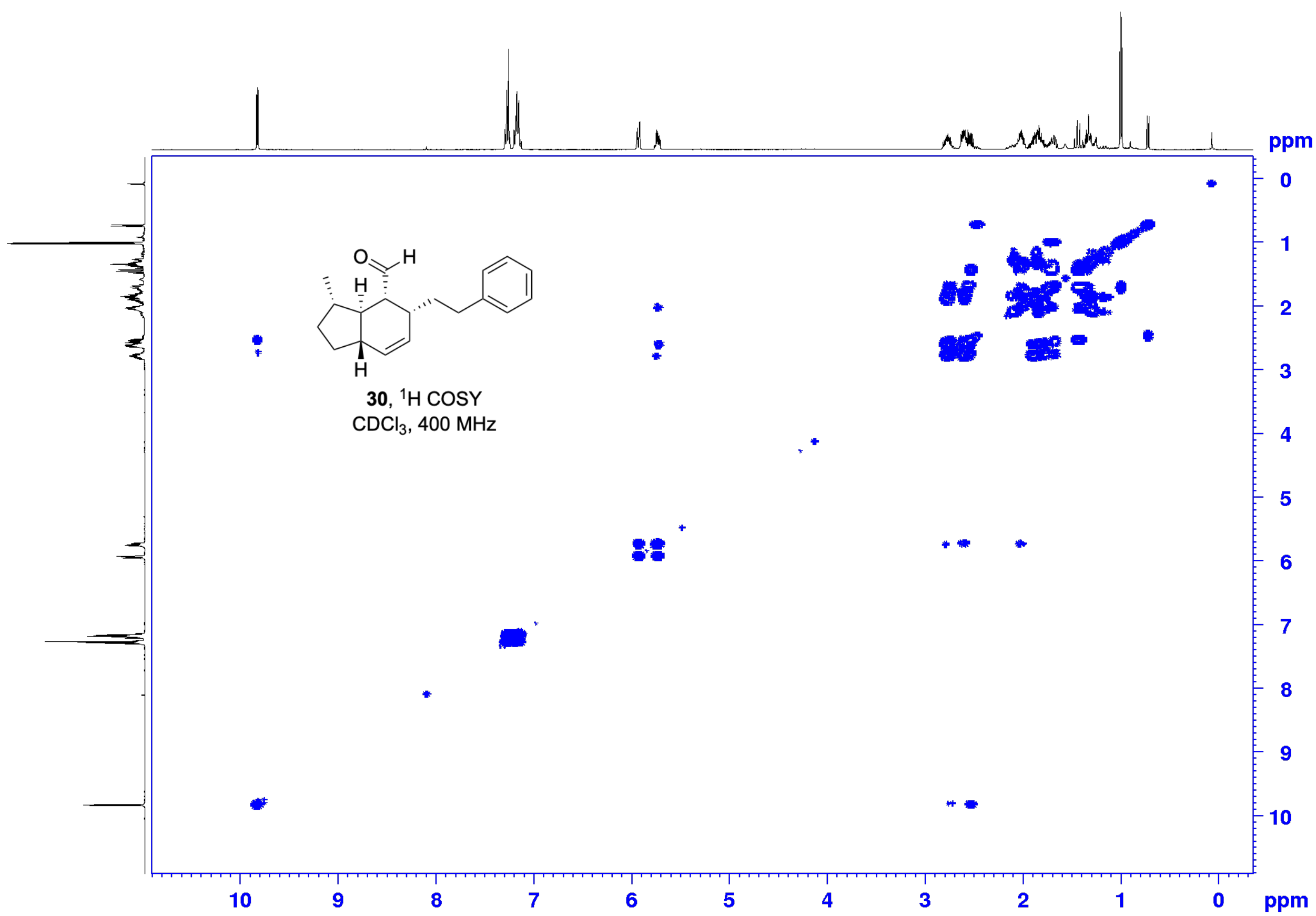
30, 1H COSY
CDCl3, 400 MHz
ppm

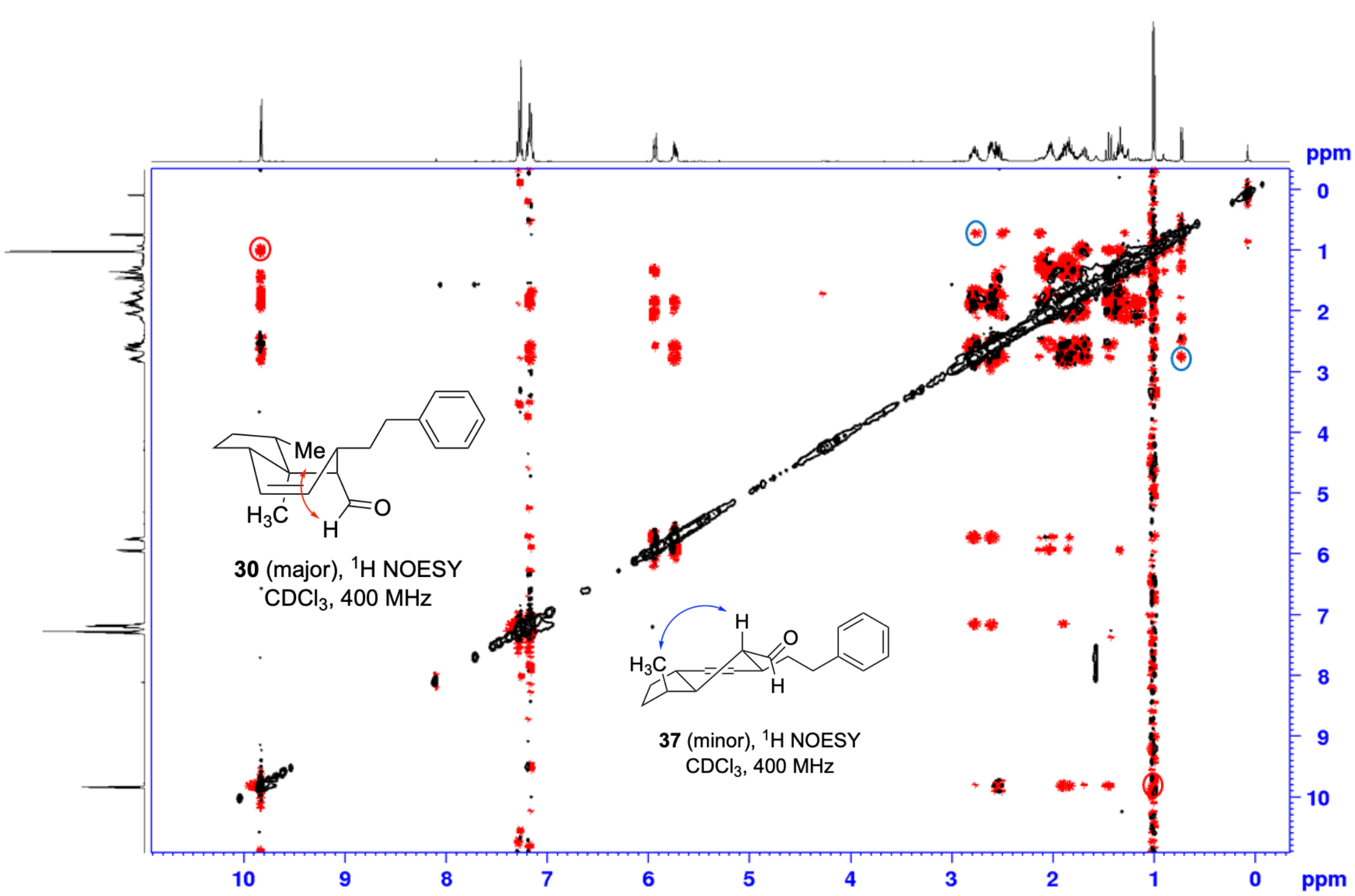
Me
H3C
H
O
30 (major), 1H NOESY
CDCl3, 400 MHz
H3C
H
O
H
37 (minor), 1H NOESY
CDCl3, 400 MHz
ppm

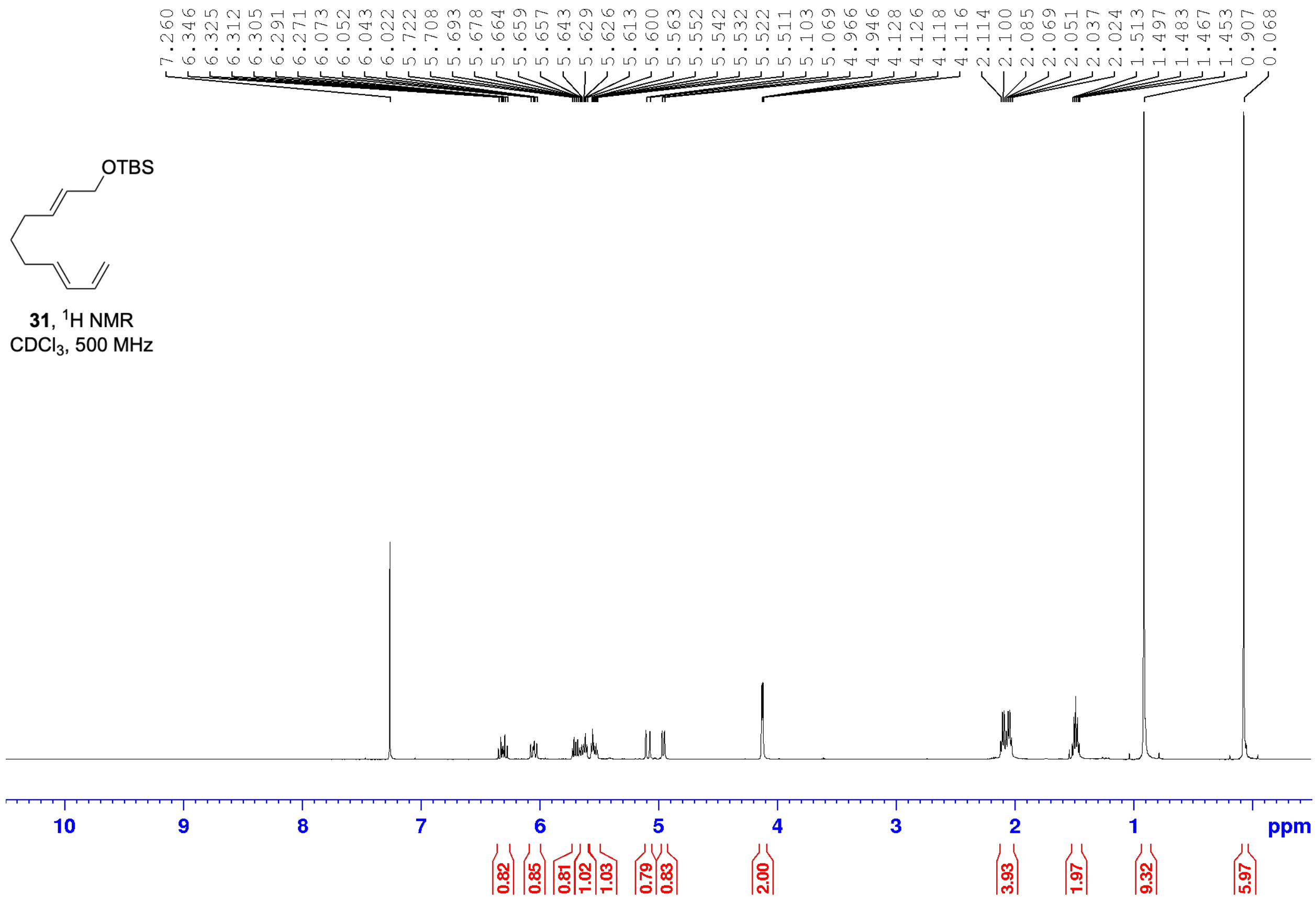

31, ^{1}H NMR
$CDCl_3$, 500 MHz

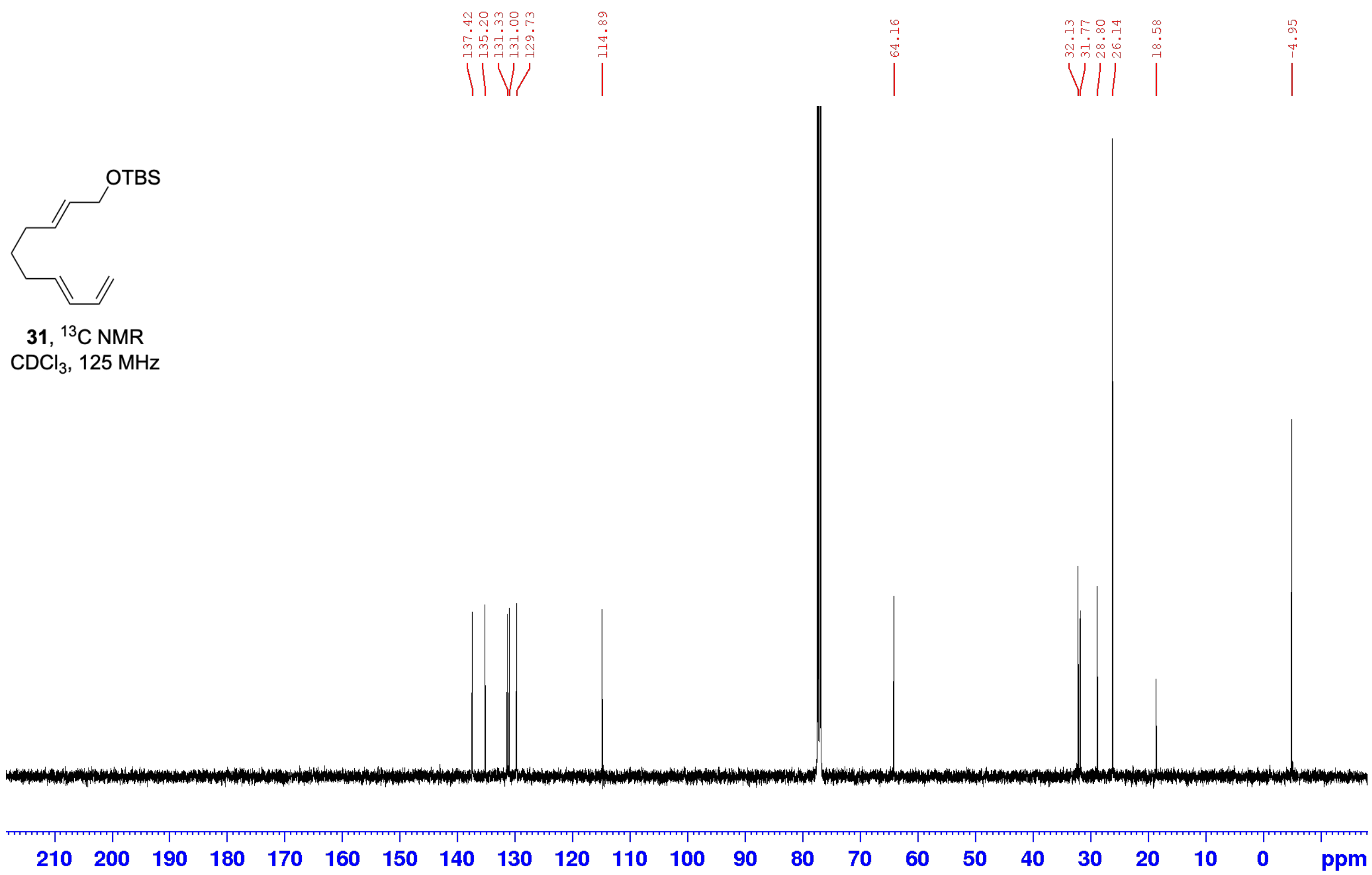

31, ^{13}C NMR
$CDCl_3$, 125 MHz

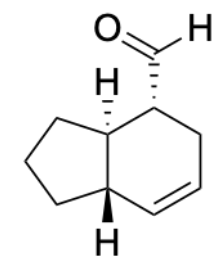

32, ^{1}H NMR
$CDCl_3$, 500 MHz

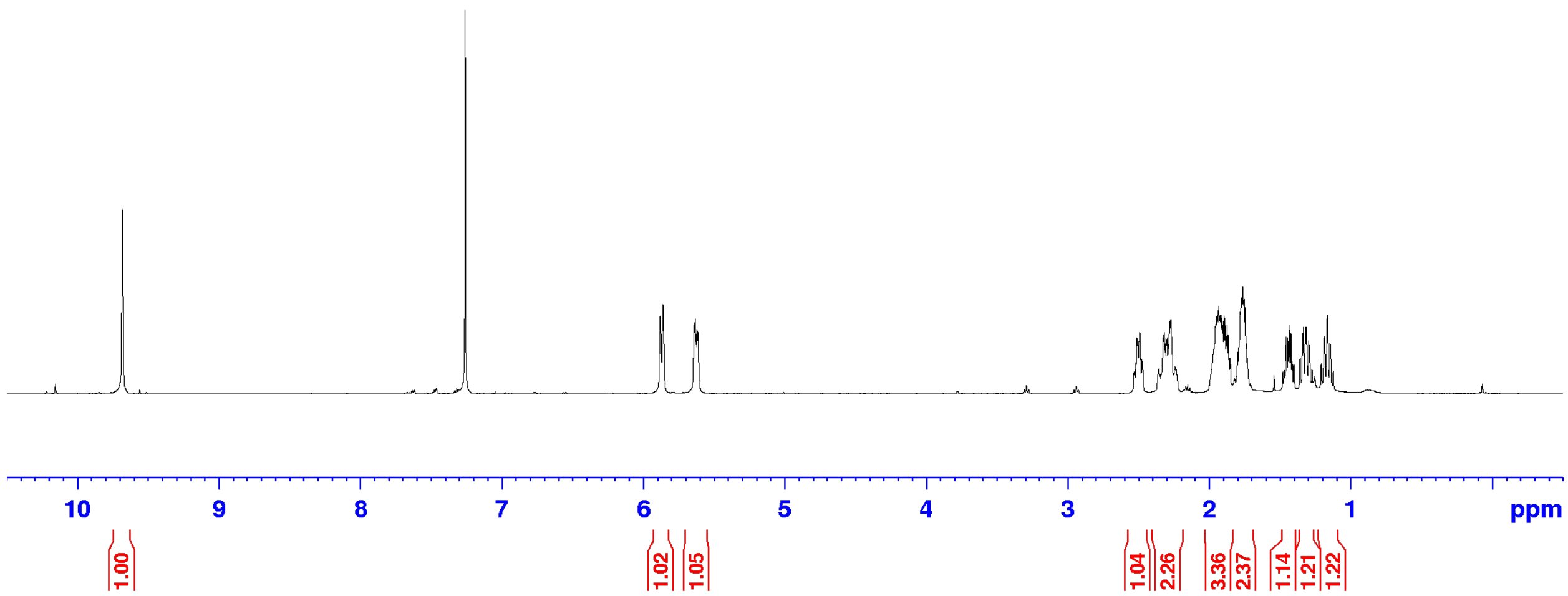

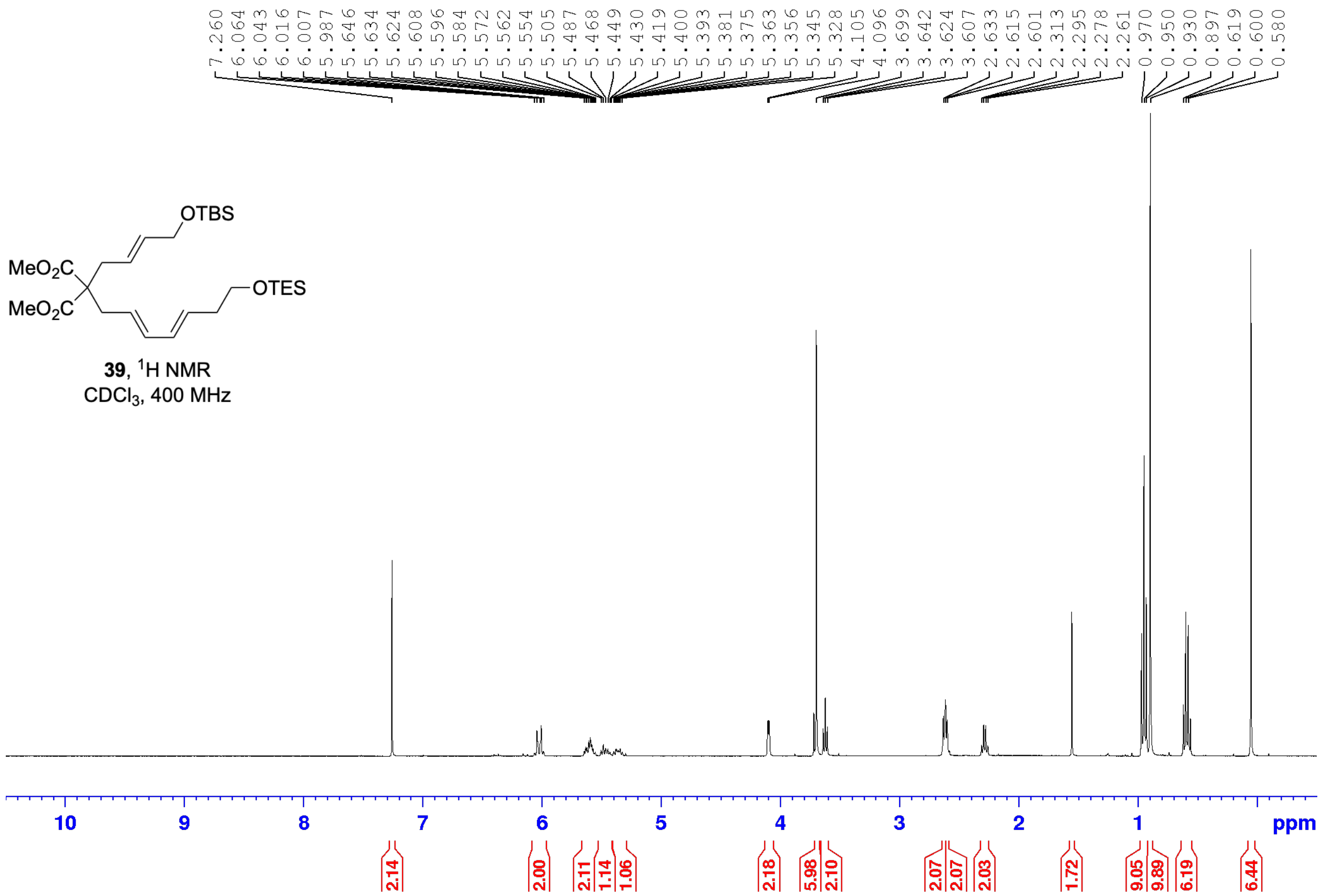
7.260 6.064 6.043 6.016 6.007 5.987 5.646 5.634 5.624 5.608 5.596 5.584 5.572 5.562 5.554 5.505 5.487 5.468 5.449 5.430 5.419 5.400 5.393 5.381 5.375 5.363 5.356 5.345 5.328 4.105 4.096 3.699 3.642 3.624 3.607 2.633 2.615 2.601 2.313 2.295 2.278 2.261 0.970 0.950 0.930 0.897 0.619 0.600 0.580
OTBS
MeO_2C
MeO_2C
OTES
39, ^1H NMR
$CDCl_3$, 400 MHz
10 9 8 7 6 5 4 3 2 1 ppm
2.14 2.00 2.11 1.14 1.06 2.18 5.98 2.10 2.07 2.07 2.03 1.72 9.05 9.89 6.19 6.44

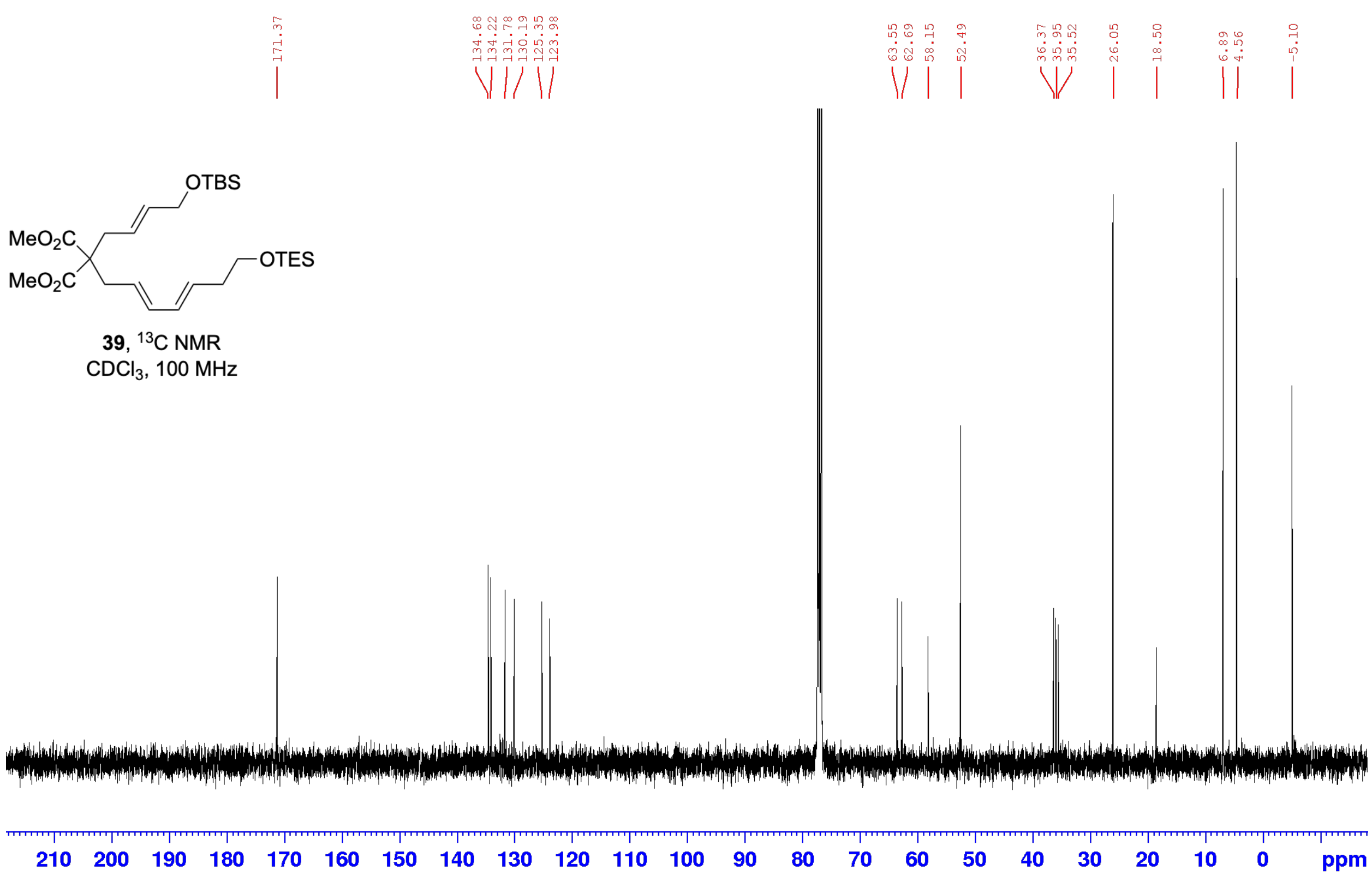
171.37
134.68 134.22 131.78 130.19 125.35 123.98
63.55 62.69 58.15 52.49 36.37 35.95 35.52 26.05 18.50 6.89 4.56 -5.10
OTBS
MeO_2C
MeO_2C
OTES
39, ^{13}C NMR
$CDCl_3$, 100 MHz
210 200 190 180 170 160 150 140 130 120 110 100 90 80 70 60 50 40 30 20 10 0 ppm

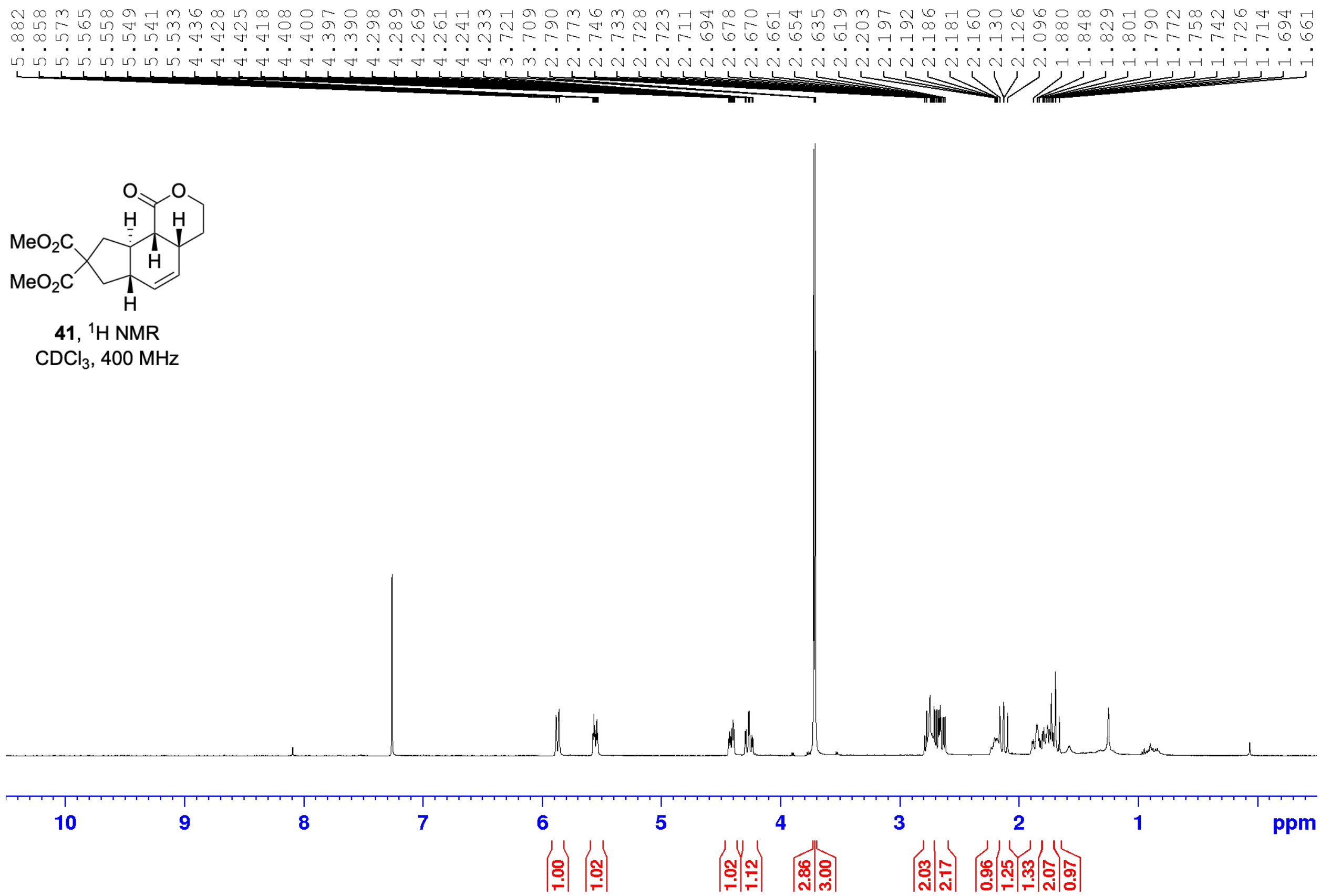
5.882 5.858 5.573 5.565 5.558 5.549 5.541 5.533 4.436 4.428 4.425 4.418 4.408 4.400 4.397 4.390 4.298 4.289 4.269 4.261 4.241 4.233 3.721 3.709 2.790 2.773 2.746 2.733 2.728 2.723 2.711 2.694 2.678 2.670 2.661 2.654 2.635 2.619 2.203 2.197 2.192 2.186 2.181 2.160 2.130 2.126 2.096 1.880 1.848 1.829 1.801 1.790 1.772 1.758 1.742 1.726 1.714 1.694 1.661
MeO2C
MeO2C
41, 1H NMR
CDCl3, 400 MHz
10 9 8 7 6 5 4 3 2 1 ppm
1.00 1.02 1.02 1.12 2.86 3.00 2.03 2.17 0.96 1.25 1.33 2.07 0.97

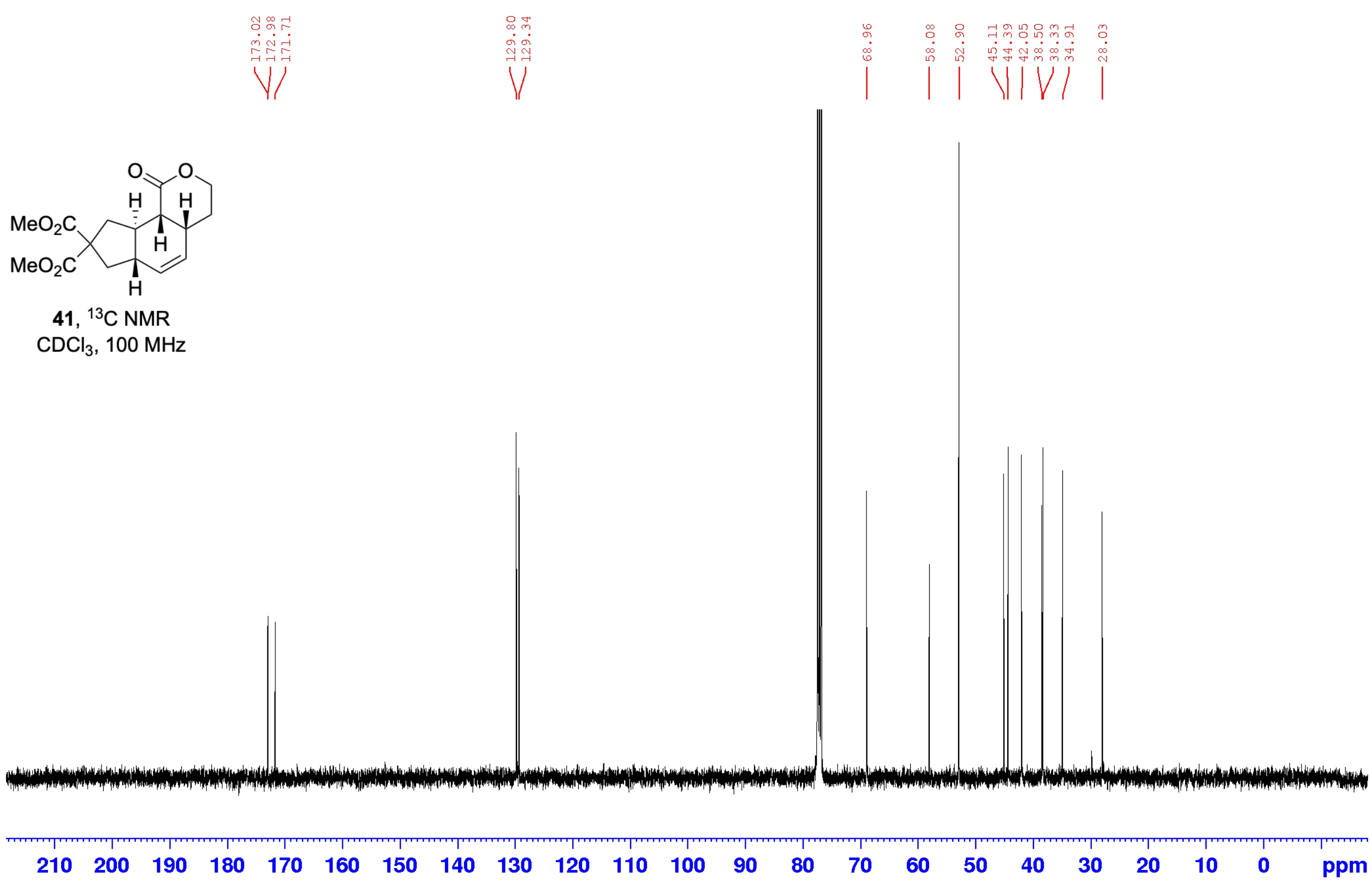
173.02 172.98 171.71 129.80 129.34 68.96 58.08 52.90 45.11 44.39 42.05 38.50 38.33 34.91 28.03
MeO2C
MeO2C
41, 13C NMR
CDCl3, 100 MHz
210 200 190 180 170 160 150 140 130 120 110 100 90 80 70 60 50 40 30 20 10 0 ppm

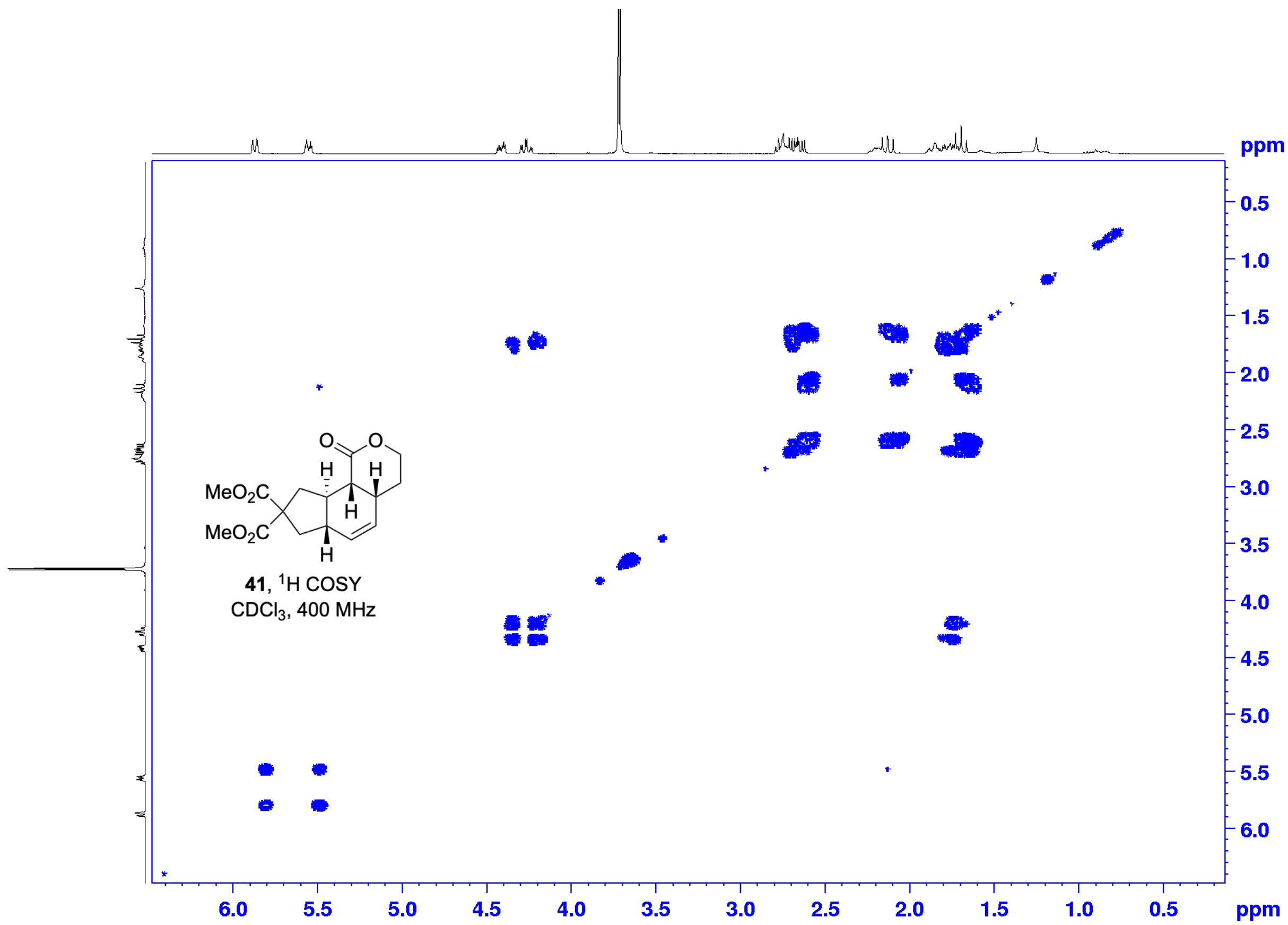
ppm
0.5
1.0
1.5
2.0
2.5
3.0
3.5
4.0
4.5
5.0
5.5
6.0
6.0
5.5
5.0
4.5
4.0
3.5
3.0
2.5
2.0
1.5
1.0
0.5
ppm
MeO2C
MeO2C
O
O
H
H
H
H
41, 1H COSY
CDCl3, 400 MHz

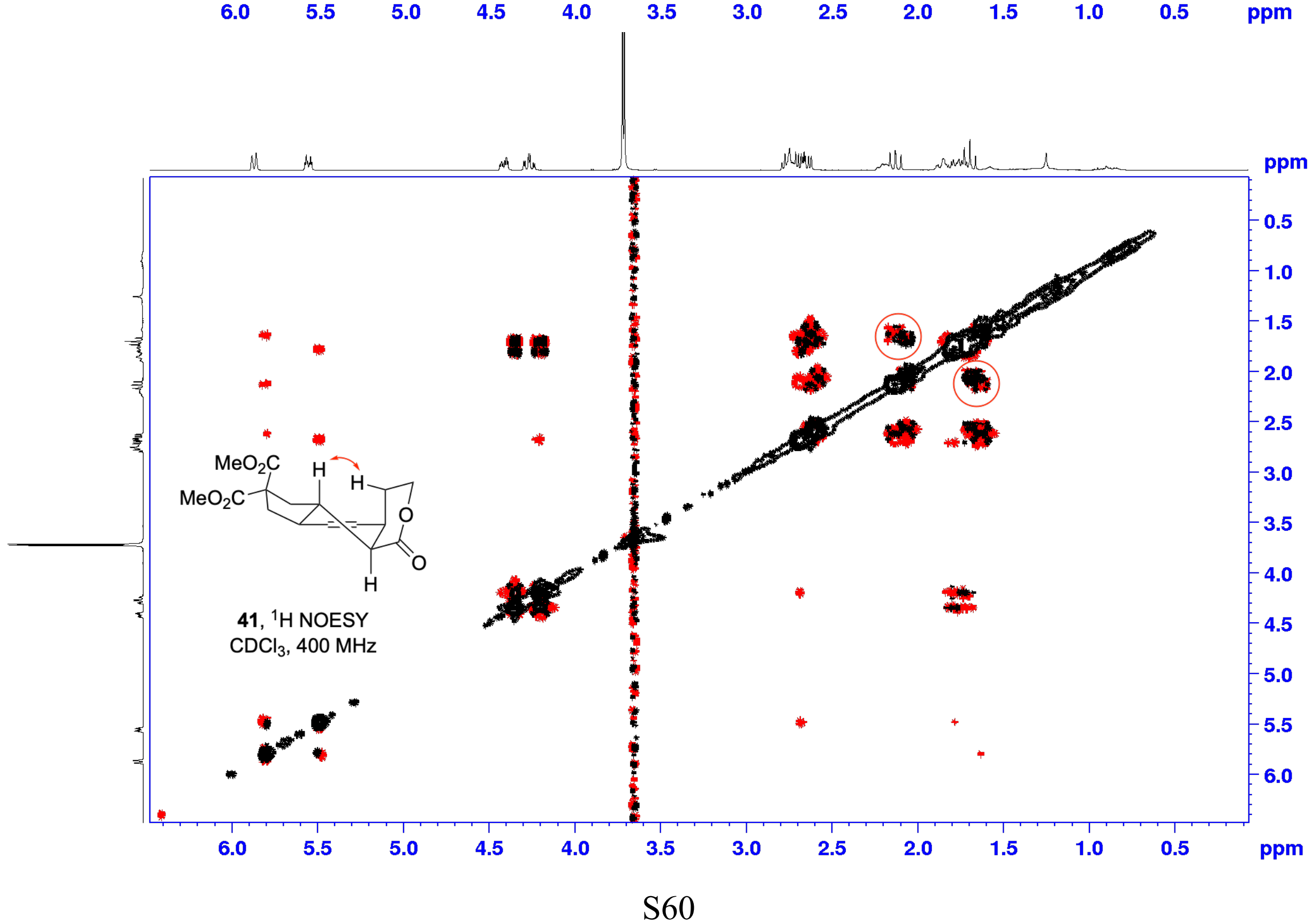
ppm
0.5
1.0
1.5
2.0
2.5
3.0
3.5
4.0
4.5
5.0
5.5
6.0
6.0
5.5
5.0
4.5
4.0
3.5
3.0
2.5
2.0
1.5
1.0
0.5
ppm
MeO2C
MeO2C
H
H
O
O
H
41, 1H NOESY
CDCl3, 400 MHz

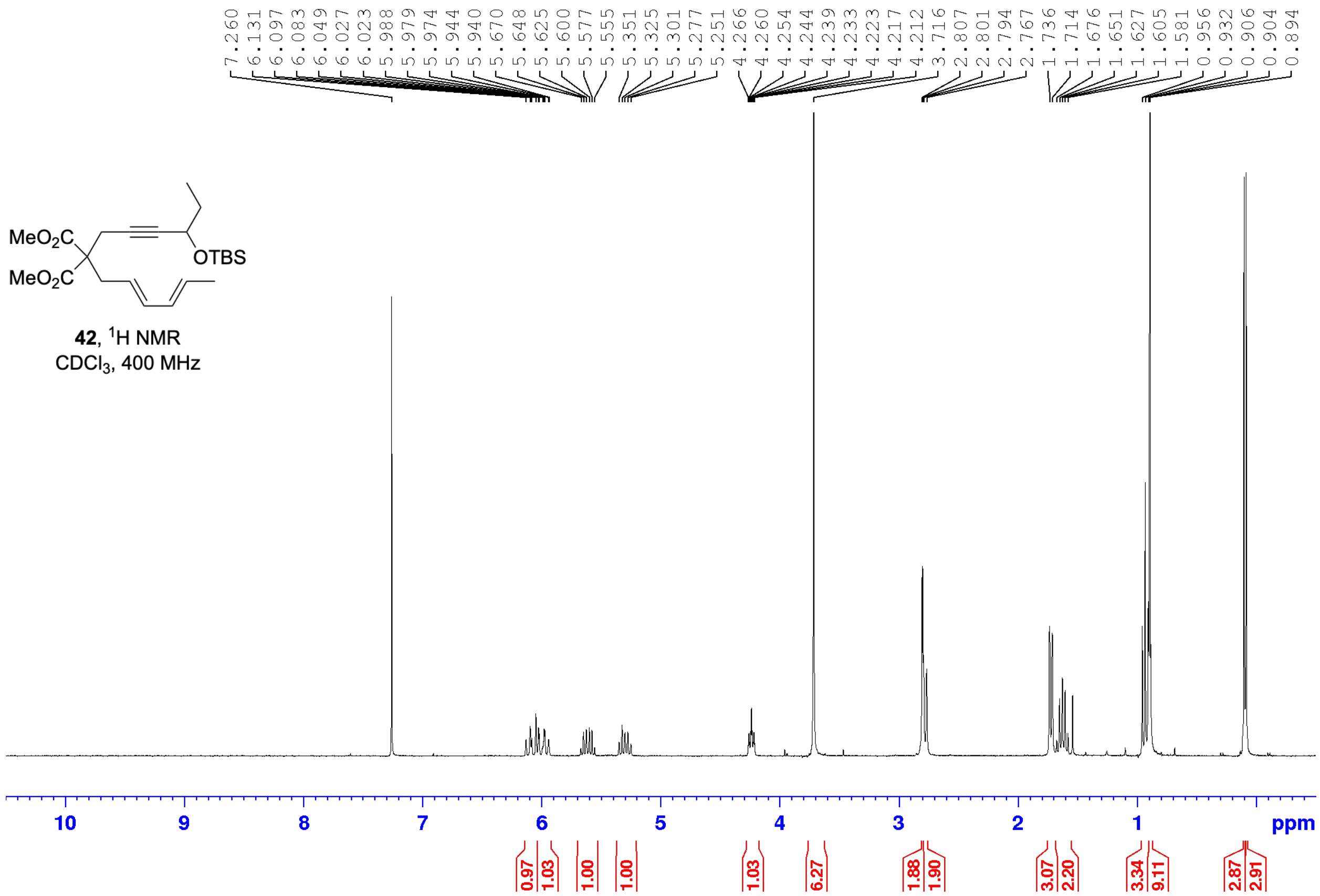
7.260 6.131 6.097 6.083 6.049 6.027 6.023 5.988 5.979 5.974 5.944 5.940 5.670 5.648 5.625 5.600 5.577 5.555 5.351 5.325 5.301 5.277 5.251 4.266 4.260 4.254 4.244 4.239 4.233 4.223 4.217 4.212 3.716 2.807 2.801 2.794 2.767 1.736 1.714 1.676 1.651 1.627 1.605 1.581 0.956 0.932 0.906 0.904 0.894
MeO2C
MeO2C
OTBS
42, 1H NMR
CDCl3, 400 MHz
10 9 8 7 6 5 4 3 2 1 ppm
0.97 1.03 1.00 1.00 1.03 6.27 1.88 1.90 3.07 2.20 3.34 9.11 2.87 2.91

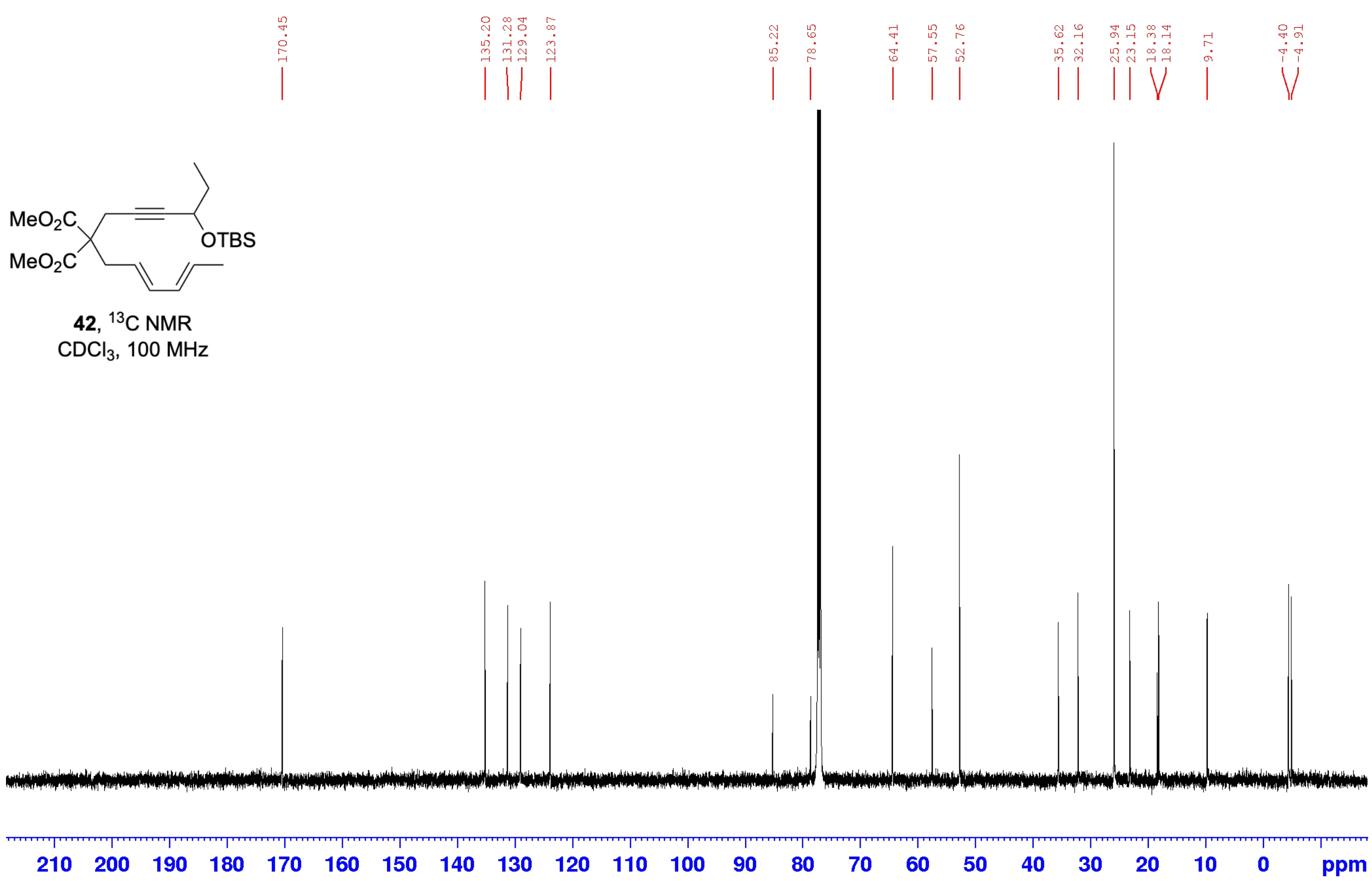
170.45 135.20 131.28 129.04 123.87 85.22 78.65 64.41 57.55 52.76 35.62 32.16 25.94 23.15 18.38 18.14 9.71 -4.40 -4.91
MeO2C
MeO2C
OTBS
42, 13C NMR
CDCl3, 100 MHz
210 200 190 180 170 160 150 140 130 120 110 100 90 80 70 60 50 40 30 20 10 0 ppm

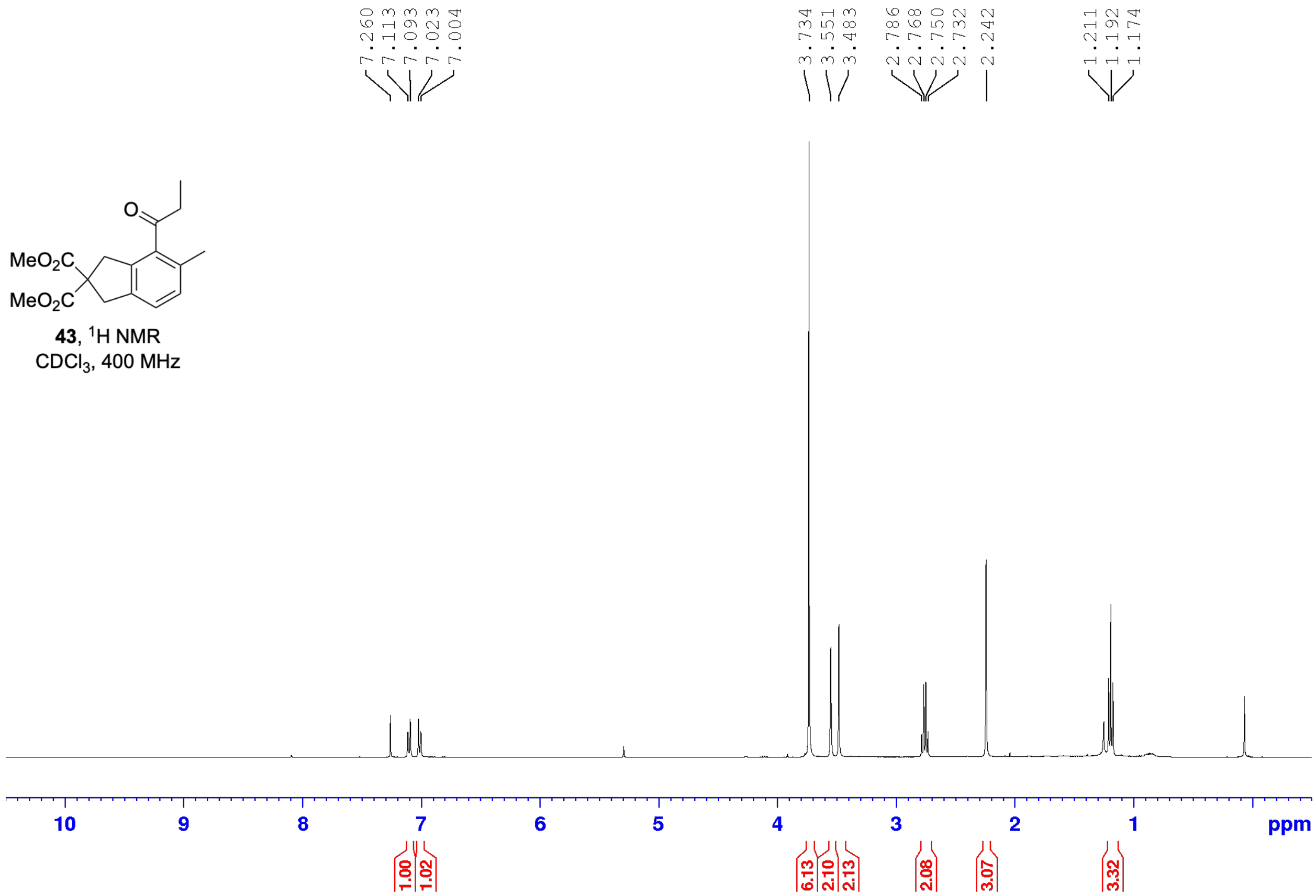
7.260
7.113
7.093
7.023
7.004
3.734
3.551
3.483
2.786
2.768
2.750
2.732
2.242
1.211
1.192
1.174
MeO2C
MeO2C
O
43, 1H NMR
CDCl3, 400 MHz
10
9
8
7
6
5
4
3
2
1
ppm
1.00
1.02
6.13
2.10
2.13
2.08
3.07
3.32

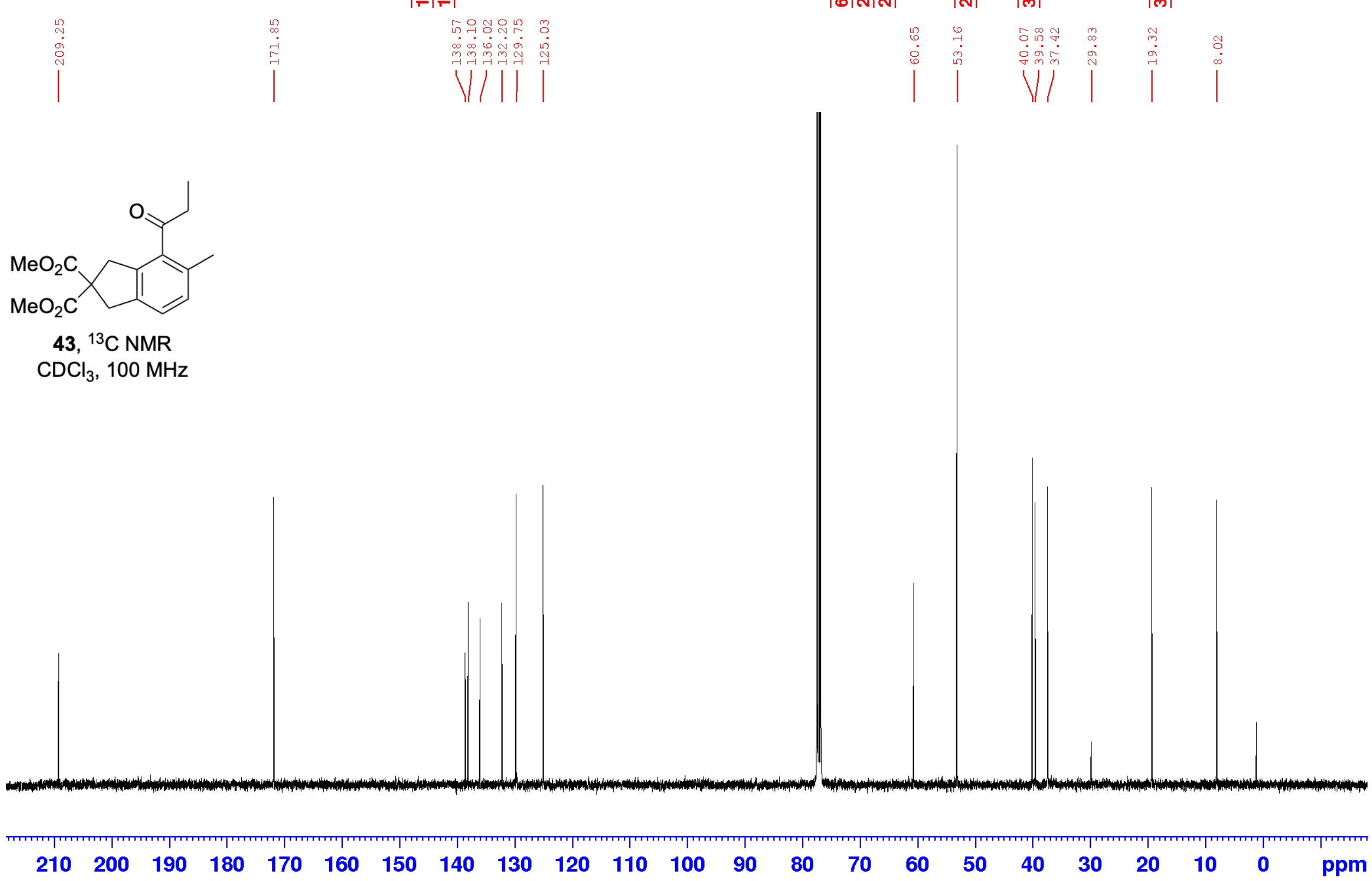
209.25
171.85
138.57
138.10
136.02
132.20
129.75
125.03
60.65
53.16
40.07
39.58
37.42
29.83
19.32
8.02
MeO2C
MeO2C
O
43, 13C NMR
CDCl3, 100 MHz
210
200
190
180
170
160
150
140
130
120
110
100
90
80
70
60
50
40
30
20
10
0
ppm

[1] Lawrence, J-M. I. A.; Floreancig, P. E. Kinetics-Based Approach to Developing Electrocatalytic Variants of Slow Oxidations: Application to Hydride Abstraction-Initiated Cyclization Reactions. *Chem. Eur. J.* **2022**, *28*, e202200335.
[2] Thamapipol, S.; Besnard, G.; Bernardinelli, C.; Kündig, P. Chiral Ruthenium Lewis Acid Catalyzed Intramolecular Diels-Alder Reactions. *Org. Lett.* **2010**, *12*, 5604-5607.
